# Mitigating Molecular Aggregation in Drug Discovery With Predictive Insights From Explainable AI

**DOI:** 10.1002/anie.202503259

**Published:** 2025-06-01

**Authors:** Hunter Sturm, Jonas Teufel, Kaitlin A. Isfeld, Pascal Friederich, Rebecca L. Davis

**Affiliations:** ^1^ Department of Chemistry University of Manitoba Winnipeg Canada; ^2^ Institute of Theoretical Informatics Karlsruhe Institute of Technology Karlsruhe Germany; ^3^ Institute of Nanotechnology Karlsruhe Institute of Technology Karlsruhe Germany

**Keywords:** Aggregation, Chemoinformatics, Computational chemistry, Explainable AI, Graph neural network

## Abstract

Herein, we present the application of multi‐channel graph attention network (MEGAN), our explainable AI (xAI) model, for the identification of small colloidally aggregating molecules (SCAMs). This work offers solutions to the long‐standing problem of false positives caused by SCAMs in high‐throughput screening for drug discovery and demonstrates the power of xAI in the classification of molecular properties that are not chemically intuitive based on our current understanding. We leverage xAI insights and molecular counterfactuals to design alternatives to problematic compounds in drug screening libraries. Additionally, we experimentally validate the MEGAN prediction classification for one of the counterfactuals and demonstrate the utility of counterfactuals for altering the aggregation properties of a compound through minor structural modifications. The integration of this method in high‐throughput screening approaches will help combat and circumvent false positives, providing better lead molecules more rapidly and thus accelerating drug discovery cycles.

## Graphical abstract

In this work, MEGAN, our explainable AI model, is applied to predict small colloidally aggregating molecules (SCAMs), a significant source of false positives in drug discovery. MEGAN provides interpretable predictions and proposes structurally similar, non‐aggregating alternative molecules. Experimental validation of one such alternative demonstrates MEGAN's potential to guide molecular design and improve high‐throughput screening reliability.

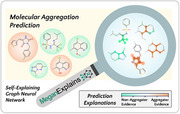

## Introduction

Interest in the application of machine learning (ML) in lead discovery has grown substantially in recent years, driven by academic and industrial initiatives to apply ML methods during early‐stage drug discovery.^[^ ^1^, ^2^ ^]^ This trend is largely attributed to the availability of extensive datasets containing activity data generated through high‐throughput screening (HTS) campaigns. The activity data produced by HTS has long been essential for hit identification in early‐stage drug discovery and is becoming even more critical with the growing interest in ML approaches for predicting lead compounds.

A persistent challenge in HTS‐based hit identification is the prevalence of false hits. Although large‐scale HTS campaigns typically generate numerous initial hits, only a small proportion represents the desired interactions between compounds and their target biomolecules. Many screening libraries contain a significant number of false positive and negative data points, with up to 80%–95% of the hits from initial screening representing artifacts.^[^ ^3^, ^4^ ^]^ This long‐standing challenge in medicinal chemistry now extends to ML‐based approaches for drug discovery. Models trained on datasets containing large numbers of false hits are prone to predicting compounds that are not viable as leads. Addressing these issues in data quality is essential to enhance the hit discovery efforts of medicinal chemists as well as the predictive prowess of ML models.

Colloidal aggregation represents a significant source of false positives in HTS.^[^ ^5^ ^]^ Aggregation occurs when molecules form supramolecular complexes, or colloids, at or above a critical aggregation concentration.^[^ ^6^ ^]^ Small colloidally aggregating molecules (SCAMs) can interact nonspecifically with proteins, leading to local unfolding and functional disruption, or they can interfere through mechanisms such as aggregation‐induced emission, where self‐assembled molecules fluoresce upon reaching their critical aggregation concentration.^[^ ^6^, ^7^ ^]^ Estimates suggest that 15%–20% of small molecules in public chemogenomic databases aggregate under standard screening conditions, underscoring the need for accurate prediction of aggregation to mitigate its impact on drug discovery.^[^ ^8^ ^]^

**Figure 1:**
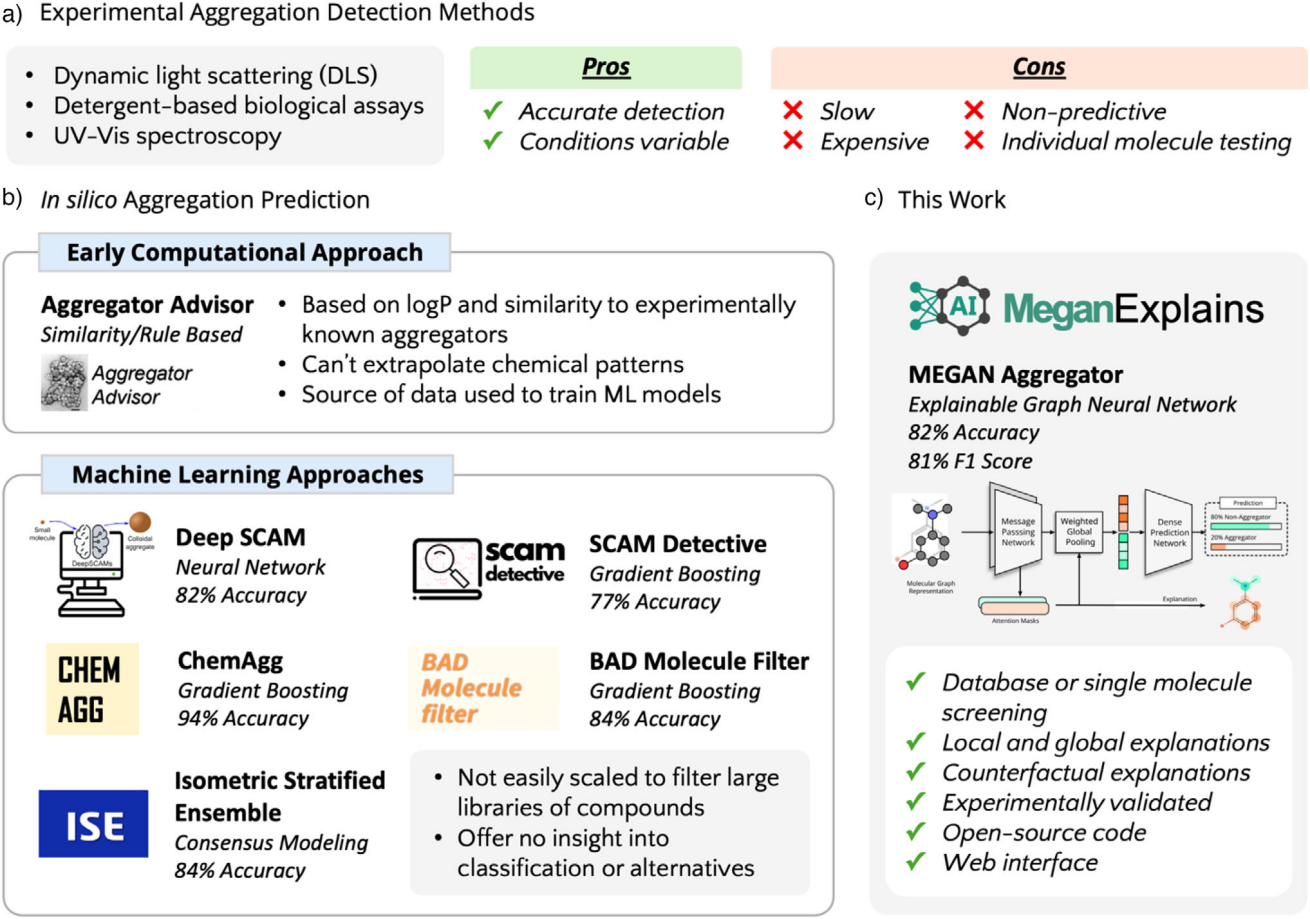
Summary of a) experimental and b) in silico approaches to aggregation determination c) compared with our work. Accuracies are those reported in the respective publications (refs. [8, 9, 10, 11, 12, 13]) and are thus not directly comparable due to differences in training and testing datasets.

The experimental detection of SCAMs is both expensive and time‐consuming, which has led to numerous in silico methods being developed to screen aggregating compounds from HTS datasets (Figure 1).^[^ ^8^, ^9^, ^10^, ^11^, ^12^, ^13^, ^14^, ^15^ ^]^ One of the earliest tools, Aggregator Advisor, evaluates molecules represented as SMILES strings and determines their similarity to known aggregators based on LogP and Tanimoto similarity (Figure 1b).^[^ ^9^ ^]^ In addition to providing a rule‐based in silico method for aggregation screening, Aggregator Advisor has also provided the field with a valuable database of experimentally validated aggregators.^[^ ^9^ ^]^ This dataset has been pivotal in the development and training of most of the ML models for aggregation prediction, enabling these models to classify small molecules as aggregators with greater accuracy and reliability (Figure 1b).^[^ ^8^, ^10^, ^11^, ^12^, ^13^ ^]^

ML‐based methods for the classification of aggregation have demonstrated accuracies exceeding 80% in predicting aggregators. However, these methods often do not provide scalable implementations or open‐source code that allows for the filtering of large molecular libraries.^[^ ^11^ ^]^ Scalable approaches, such as SCAM Detective and DeepSCAM, typically achieve accuracies in the range of 70%–80%.^[^ ^8^, ^10^ ^]^ Consequently, there remains a critical need for an accurate, scalable, and interpretable method capable of efficiently screening large libraries of compounds.

Aggregation is a complex phenomenon that is influenced by many variables, including concentration, pH, temperature, buffer, and solvent.^[^ ^16^, ^17^ ^]^ Although numerous studies have explored the molecular features that contribute to small molecule aggregation, no clear consensus has been reached on the key factors driving this phenomenon.^[^ ^18^ ^]^ Features such as logP, the number of hydroxyl groups, the number of sulfur atoms, and the number of aromatic rings are often proposed to contribute to aggregate formation; however, the complexity of molecular aggregation has led to difficulty in identifying reliable, universal trends distinguishing aggregating and non‐aggregating compounds.^[^ ^8^, ^9^, ^11^ ^]^ Given the absence of generalizable trends for aggregation prediction, there is a growing need for predictive models that can learn complex, nonlinear relationships. In this context, ML offers a powerful framework for the prediction of molecular aggregation and revealing insights into the structural patterns found and used by the ML models.

This study addresses the need for an accurate and scalable model capable of detecting SCAMs while providing interpretable explanations and the ability to create non‐aggregating counterfactuals (Figure 1c). The explainable AI (xAI) model employed in this study, a multi‐channel graph attention network (MEGAN), achieves an accuracy of 82% in predicting SCAMs and is suitable for screening both large molecular libraries and individual compounds.^[^ ^19^ ^]^ Furthermore, the model generates explanations for its classifications, offering insights into why a compound is predicted as a SCAM or non‐SCAM. The accompanying web server allows users to screen individual molecules and provides a user‐specified number of counterfactual explanations. These counterfactuals are structurally similar to the query molecule but possess flipped classification labels (e.g., counterfactuals for a molecule predicted to aggregate are structurally similar molecules predicted to be non‐aggregating, and vice versa). To validate our model and its application of counterfactuals, we synthesized and experimentally tested a non‐aggregating derivative of clioquinol—an established aggregator—proposed by the model. The experimental results confirm the model's prediction and demonstrate the experimental relevance of our model in the informed design of molecules with tailored aggregation properties.

## Results and Discussion

### The MEGAN Model

**Figure 2:**
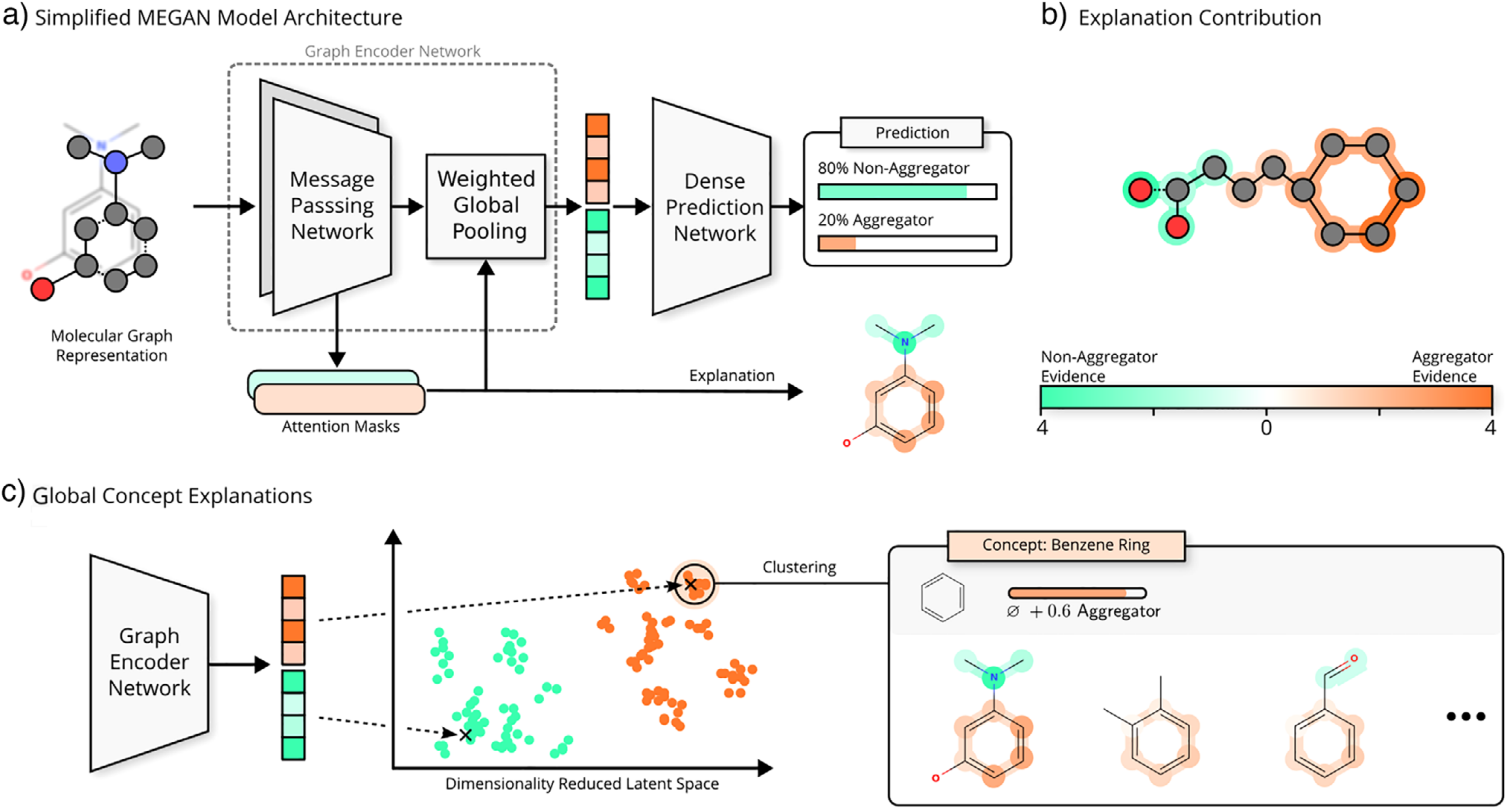
MEGAN model overview. a) Molecular graph structures are used as input to multiple attention‐based message‐passing layers. Node representations are aggregated and passed to a fully connected network to output the predicted class. Explanation masks are derived from the internal attention values. b) Attention‐based explanations are explicitly split into separate channels for each possible output class. c) Overarching structural explanations can be found by identifying clusters in the latent space of subgraph embeddings. Analyzing all members of a concept cluster yields general trends associated with certain structural motifs. Explicit explanation masks and values are constructed for illustration purposes.

To develop an accurate and scalable model for detecting SCAMs and elucidating the structure‐property relationships underlying molecular aggregation, we leverage xAI techniques applied to predictions generated by graph neural network models. To apply a graph neural network to the task of chemical property prediction, each molecule is first converted into a molecular graph where atoms are represented as nodes and bonds are represented as edges. Based on this graph‐structured input information, the graph neural network is trained on the dataset to predict the binary classification label of a given molecule as either an aggregator or a non‐aggregator (Figure 2).

In this work, we apply the multi‐explanation graph attention network (MEGAN) to the task of aggregation prediction.^[^ ^19^ ^]^ MEGAN is a self‐explaining graph neural network model architecture for which node and edge attributional explanations are directly derived from the model's internal attention and masking mechanism. These attributional explanations assign an importance value between 0 and 1 to each node and edge of a given graph to indicate which substructures of a given graph are especially influential for the predicted outcome. The MEGAN model specifically generates one such attributional explanation for each of the possible classification outcomes—one explanation highlighting the structural evidence in favor of an aggregator classification (orange) and the other explanation highlighting substructures associated with the non‐aggregator class (green) (Figure 2b).

### Prediction Accuracy and Benchmarking

We trained a MEGAN model on a dataset comprised of 12338 aggregating and 177048 non‐aggregating molecules (see Methods in Supporting Information). For a quantitative evaluation of our trained model, we used a separate test set of 1500 aggregators and 1500 non‐aggregators. The dataset was largely derived from a single experimental screen conducted under consistent conditions (e.g., phosphate buffer, pH = 7). To align molecular representations with the experimental conditions, protonation states were assigned based on physiological pH. Similar to most previously reported ML models, we achieved an accuracy of 82%. However, as we are using a training set with a class imbalance, accuracy can be misleading, as it may achieve high accuracy simply by predicting the majority class. To further evaluate the MEGAN model's performance, we employed the F1 score, which, even on balanced test sets, offers a perspective on the balance between precision and recall beyond the accuracy metric. The MEGAN model achieved an F1 score of 81%, indicating that the model has a good balance between precision and recall. The similarity in the accuracy (82%) and F1 score (81%) also suggests that the model's performance is well‐balanced across both classes (aggregators and non‐aggregators). The performance of the MEGAN model was compared against the XGboost model of Yang et al. (ChemAgg), using our balanced test set. Attempts to compare to many of the other ML models listed in Figure 1 were unsuccessful due to the inaccessibility of the code used for these models. It was found that the MEGAN model provided a higher accuracy and F1 score than the ChemAgg model (acc = 73%, F1 = 74%), demonstrating the superior performance of graph neural networks in capturing molecular structures (see Supporting Information for details).

### Sensitivity of the Model to Small Structural Modifications

**Figure 3:**
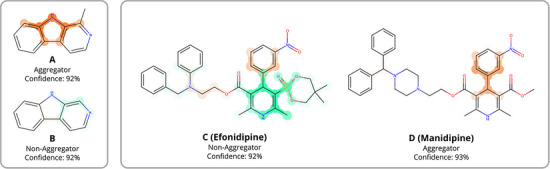
MEGAN predictions and explanation masks for two pairs of structurally similar molecules that exhibit experimentally contrasting aggregation behavior.

When evaluating the performance of the MEGAN model in predicting molecular aggregation, its predictions were observed to exhibit a high degree of sensitivity to subtle modifications in molecular structure. To illustrate this sensitivity, two groups of compounds with experimentally validated aggregation behavior are presented: one comprising molecules from the training dataset (**A**, **B**) and the other consisting of molecules external to the training dataset (**C**, **D**) (Figure 3).

Experimental studies reported in the literature have previously identified azacarbazole **A** as an aggregator and azacarbazole **B** as a non‐aggregator, despite the two compounds differing by only a methyl group. The MEGAN model correctly predicted **A** as an aggregating and **B** as a non‐aggregating compound with high confidence, likely due to their inclusion in the training dataset. However, more notable are the distinct explanation masks generated by the model for each compound. Examination of the explanation mask for **A** indicates that the methyl group does not significantly influence the model's prediction. Instead, the presence of the methyl group appears to cause the model to discriminate between different parts of the shared heterocyclic core of **A** and **B** in its classifications of each compound. The fact that the addition of a methyl group to the azacarbazole results in aggregation is not chemically intuitive, suggesting that we cannot directly derive trends in aggregation from the structures. This example shows the need for models like MEGAN to find edge cases like this and provide insight that can be further explored where traditional chemical intuition fails.

In comparing efonidipine (**C**) and manidipine (**D**), we again observe the MEGAN model's ability to correctly classify compounds based on changes in substructure. In both **C** and **D**, the model highlights the aromatic ring of the nitrobenzyl group as evidence for aggregation. In **C**, the bulky phosphonate group is identified as providing evidence against aggregation. From a chemical perspective, the explanation masks for **C** seems reasonable, as the phosphonate group likely reduces the molecule's capacity to form closely packed arrangements or stable intermolecular interactions that promote aggregate formation. In contrast to **C**, the methyl ester group of **D** decreases the attributional explanations for non‐aggregation resulting from the dihydropyridine. This would suggest that the methyl ester is less disruptive of favorable intermolecular interactions that induce aggregation in **D**. These findings suggest that the model effectively identifies key structural features that either promote or inhibit aggregation, highlighting the potential for targeted modifications to reduce aggregation behavior.

### DFT Assessment of Physical Relevance for MEGAN Model Structural Sensitivity

**Figure 4:**
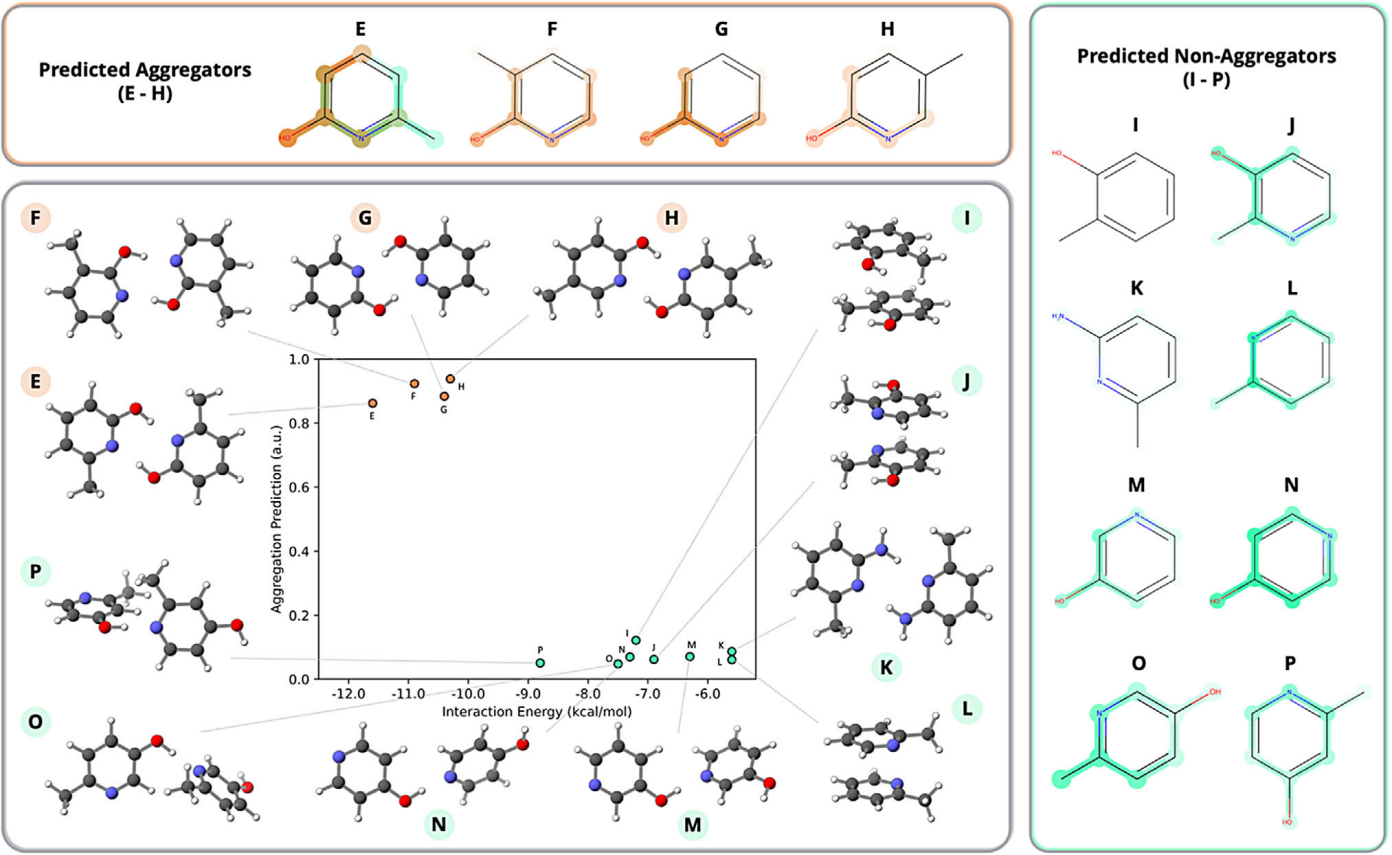
MEGAN predictions compared with DFT interaction energies of dimers for compounds **E** – **P**. Predictions are reported on a scale from 0 to 1, with 0 indicating a prediction of non‐aggregator and 1 indicating a prediction of aggregator. Also shown are the 3D structures of the optimized dimer geometries and MEGAN explanation masks for each molecule. The light‐colored explanation masks for **I** indicate that the MEGAN model detects no pattern systematically associated with either class.

To examine the physical relevance of the MEGAN model's sensitivity to small changes in molecular structure, quantum chemical modeling of a group of structurally similar compounds having different prediction labels was performed. As a significant portion of the molecules in the training dataset contain aromatic heterocycles, pyridine derivatives were selected as the primary focus of this study. This scaffold serves both as a simple example of an aromatic heterocycle and is easily modified to examine the effects of different intermolecular interactions. Although the 12 selected pyridine derivatives have not been experimentally evaluated for aggregation, their analysis provides insights into the relationship between aggregation prediction confidence and interaction energies. Based on the MEGAN model's prediction accuracy of 82%, approximately 2 of the 12 pyridine derivatives may be expected to be mislabeled; however, the functional groups that appear in the explanations are potentially still meaningful and likely trend with the actual aggregation‐enhancing or aggregation‐inhibiting behavior of these groups. The reason for that is that it is easier for the model to identify which groups trend with aggregation than to quantify the subtle relative influences of these groups on the final aggregation prediction. Therefore, even when the MEGAN model fails to predict the correct final label, it is very likely that the explanation masks are still correctly identifying and labeling relevant groups, but the model misjudges their exact relative influence on the overall aggregation likelihood. Interaction energies for the pyridine derivatives were calculated using density functional theory (DFT) and compared with the MEGAN predictions and explanation masks (Figure 4).^[^ ^20^, ^21^, ^22^ ^]^

The intermolecular interactions of pyridine derivatives predicted with high confidence as aggregators (**E**–**H**) were calculated to be at least 1.5 kcal mol^−1^ more energetically favorable than those of compounds predicted with high confidence as non‐aggregators (**I**–**P**). All 2‐hydroxypyridine derivatives were classified as aggregators with high confidence. Conformational searches of the dimers revealed that the strongest interactions between these molecules involved the formation of two intermolecular hydrogen bonds. The only other compound to form two intermolecular hydrogen bonds was 2‐aminopyridine (**K**), which was classified as a non‐aggregator. However, its interaction energy was only −6 kcal mol^−1^, likely due to the relatively weaker nature of N─H─N hydrogen bonds compared to O─H─N hydrogen bonds. Hydroxypyridines **M**, **N**, **O**, and **P** were classified as non‐aggregators and were capable of forming only one hydrogen bond, leading to weak interaction energies of greater than −9 kcal mol^−1^. The remaining compounds, **I**, **J**, and **L**, classified as non‐aggregators by the MEGAN model, all preferred conformations that promoted π–π interactions and had interaction energies of greater than −8 kcal mol^−1^. To further investigate the electronic nature of the interactions studied, energy decomposition analysis was completed using Psi4's implementation of symmetry‐adapted perturbation theory (SAPT) on the dimers of **E‐P**.^[^ ^23^, ^24^ ^]^ The SAPT results demonstrate trends that are consistent with the DFT interaction energies and show a clear distinction between the strengths of the interactions in the predicted aggregating and non‐aggregating molecules (see Supporting Information for details). Overall, this demonstrates the ability of the model to discriminate based on intermolecular interaction strength.

In examining the MEGAN model's explanation masks for the hydroxypyridines, the model is found to distinguish between the various OH substitution patterns. For the predicted aggregators (**O**‐**R**), the MEGAN model finds the HO─C─N substructure, which is responsible for the hydrogen bonding interactions in the dimers, as the key contributor to the aggregator classification (Figure 4). In contrast, for the isomers with weaker interaction energies (**J**, **M**, **N**, **O**, **P**), the model identifies the hydroxyl group as contributing to the classification of these compounds as non‐aggregators, demonstrating that the model's prediction is influenced by the substitution pattern. The MEGAN model is also observed to be sensitive to the strength of the hydrogen‐bond donating abilities of the substituents. Although the strongly hydrogen‐bonding hydroxyl group of 2‐hydroxypyridine (**G**) is highlighted as contributing to the aggregation prediction, the slightly weaker hydrogen‐bonding amino group of 2‐aminopyridine (**K**) is highlighted as contributing to a non‐aggregator prediction. Therefore, not only do the interaction energies correlate with the MEGAN predictions, but the MEGAN model is able to identify the structural features of these molecules, which may be contributing to intermolecular interactions and aggregation.

### Global Explanations

To investigate the molecular substructures commonly contributing to the MEGAN model's prediction of molecules as aggregators or non‐aggregators, a global concept extraction was performed.^[^ ^25^ ^]^ In this process, the pooled graph explanations for each channel undergo dimensionality reduction and clustering to provide clusters of molecules that share structural explanations (Figure 2c). Each cluster represents one specific molecular substructure, which occurs as an important explanation in many individual samples in the training data. All clusters from the concept extraction are presented in the Supporting Information. The concept extraction produced 159 clusters, with 25 associated with the non‐aggregator channel and the remaining 134 clusters associated with the aggregator channel.

Visual analysis of the clustered concepts suggests that flexible molecules and molecules containing groups that have the potential to disrupt π‐stacking through sterics often contribute to the non‐aggregation prediction. Alternatively, flat and rigid molecules, as well as molecules with functional groups that can act as both hydrogen bond donors and acceptors, contribute to the prediction of a molecule as an aggregator. Many of the trends that were identified in our clustering analysis are consistent with those that have been identified previously in the literature for classifying molecular aggregation potential in small molecules.^[^ ^8^, ^9^, ^10^, ^11^, ^12^, ^13^ ^]^

One notable trend identified in our cluster analysis, not previously reported in the literature, is the influence of thioureas and ureas on the classification of molecular aggregation. The model frequently associates ureas with aggregation, whereas the thiourea moiety is strongly linked to non‐aggregator labels. Interestingly, while the model has identified the urea and thiourea substructures as important to the classification of molecules as aggregating or non‐aggregating, there is no notable difference in the relative frequency of the urea and thiourea substructures between the aggregating and non‐aggregating datasets used for model training (relative frequencies provided in the Supporting Information (SVII and SXVI)).

It was further identified that when thioureas are adjacent to an electron‐withdrawing group, such as a carbonyl, they instead contribute to the aggregator label. This indicates that the local electronic environment of the urea and thiourea substructures plays a decisive role in the aggregation tendencies of molecules containing these functional groups. Traditional fingerprint‐based feature attribution methods would miss these subtleties, as they focus on substructure presence or absence. In contrast, MEGAN explanations reveal how specific atom environments and functional group contexts affect predictions. The insight provided by analysis of the global explanations has practical implications as it identifies new functional groups that correlate with aggregation but also highlights the critical importance of the local electronic and steric environment in controlling aggregation tendencies. This emphasizes the need for the MEGAN model and its explanations to capture the complexity of molecular aggregation.

To analyze whether the structural explanations generated based on the MEGAN model can be analyzed and interpreted in terms of physicochemical concepts in an automated way, we used an approach based on the prior chemical knowledge and the pattern‐recognition abilities of large language models, specifically GPT‐4o (see Supporting Information (SIX) for prompts and results). The objective was to connect structural graph explanations to broader chemical concepts and human‐understandable chemical trends in order to stimulate further ideas for more detailed analysis by experts and to potentially derive design rules.

When prompted to explain why molecular motifs derived from the global explanation analysis trend with aggregation, the large language model (LLM) outputs referred to relevant concepts such as potential interactions with water molecules and dimer interactions. Specifically, the output of the GPT‐4o model referred to hydrogen bonding effects, planar structures that promote π‐stacking, as well as steric hindrance of π‐stacking—effects that are commonly associated with aggregation and thus true but not novel.

However, in a subsequent blind test, we prompted the GPT‐4o model with all structural motifs identified by the MEGAN model through the global explanation analysis, but did not reveal the context of aggregation or the specific role of the motifs in enhancing or reducing aggregation, in order to reduce the bias of the model to just repeat already known prior knowledge of aggregation. The results agreed surprisingly well with the results of the previous text, with hydrogen bonding, π‐stacking, and sterics being important characteristics that separate the two groups of motifs. Some physicochemical characteristics and thus possible explanations were additionally mentioned that we did not consider before as relevant descriptors, e.g., electron‐donating effects, which lead to increased electron density in the π‐system of an aryl ring and facilitate solvation over intermolecular π–π stacking interactions. This provides insight that can be validated and quantified in further experiments. Overall, this demonstrates how specialized models, such as MEGAN, can potentially be interfaced with general‐purpose models such as LLMs to provide insight for the analysis of complex and not‐well‐understood datasets.^[^ ^26^ ^]^

### Counterfactuals

**Figure 5:**
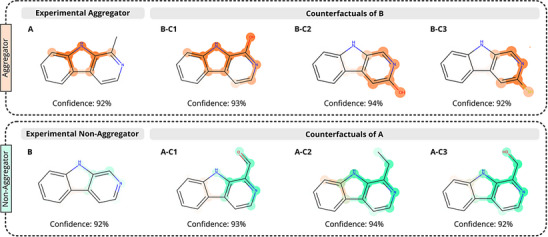
Counterfactuals of experimentally validated aggregator **A** and non‐aggregator **B** with MEGAN model's prediction confidence and explanation masks.

In addition to MEGAN's attributional explanations, we also employ counterfactual explanations to gain further insights into the model's behavior and decision‐making process. For a given original input molecule, we define a counterfactual as a molecule with a minimal structural change from the original molecule, which causes the greatest deviation in the model's prediction. Counterfactuals explain which kinds of local perturbations to chemical structure the model, and by extension, the underlying aggregation property, is most sensitive to. To probe some of the structural features and modifications influencing the model's classification predictions, counterfactuals were generated for azacarbazoles **A** and **B** (Figure 5). Recalling from the previous discussion, the model correctly classifies **A** as aggregating and **B** as non‐aggregating despite their subtle structural differences (Figure 3).

In general, the counterfactuals generated for aggregator **A** include molecules with electron‐withdrawing groups added to the pyridine ring of the azacarbazole as well as molecules with additional steric bulk added to the carbon of the methyl group at the C1 position of the azacarbazole. The addition of an electron‐withdrawing group, such as an aldehyde (**A‐C1**), to the pyridine ring was found to switch the classification label from aggregator to non‐aggregator. Based on this observation, we suggest that electron‐withdrawing groups could be capable of disrupting or altering the π‐stacking arrangements that may produce aggregation in **A** by modifying the electronics of the azacarbazole ring system. Additionally, steric bulk added to the methyl group of the azacarbazole (**A‐C1**, **A‐C2**, **A‐C3**) results in larger, more flexible substituents, which sit out of the plane of the ring system, potentially disrupting favorable π‐stacking arrangements and preventing aggregation. The non‐aggregator channel's explanation masks for the counterfactuals of **A** are nearly identical to those of the non‐aggregator **B**, with the only substantial difference being that the additional substituent is also highlighted in the counterfactuals.

The counterfactuals generated for non‐aggregator **B** included molecules containing either a hydroxyl (**B‐C1**, **B‐C2**) or thiol group (**B‐C3**) adjacent to the nitrogen atom of the pyridine ring. These analogous substructures, which are ideally suited for forming hydrogen bonding interactions that may contribute to aggregation, are frequently observed in molecules classified by the model as aggregators. These findings are consistent with trends observed in the study of pyridine derivatives (**E**‐**H**), where the HO─C─N substructure was found to be critical for aggregation.

The explanation masks for each of the counterfactuals of **B** (**B‐C1**, **B‐C2**, **B‐C3**) highlight the hydroxy/thiol pyridine substructures in red, indicating that these substructures provide the model with evidence for the aggregation class. The identification of these functional groups is also consistent with the results of other studies, in which thiols and hydroxyl groups have been identified as functional groups commonly inducing aggregation.^[^ ^8^, ^9^, ^10^, ^11^, ^12^, ^13^ ^]^ Interestingly, compound **A‐C3**, which contains a hydroxyl group not directly bound to the pyridine, is predicted as non‐aggregating, suggesting that the hydroxyl group alone is not a predictor of aggregation for these compounds and further emphasizing the importance of the hydroxy/thio pyridine substructure.

### Experimental Validation of Predictions and Counterfactuals

In addition to serving as a means for identifying the structural features of molecules contributing to their classification as aggregators or non‐aggregators by the MEGAN model, counterfactuals also allow for the prediction of new molecules highly similar to an input molecule with alternate aggregation properties. Applying the model in such a manner provides a method for the design of new compounds that maintain the desired structural aspects of a molecule, which may be crucial to forming necessary interactions within a biological target while modifying its aggregation tendencies. The following example provides experimental validation of the MEGAN prediction classification for one of the counterfactuals and demonstrates the utility of counterfactuals for altering the aggregation properties of a compound through minor structural modifications.

**Figure 6:**
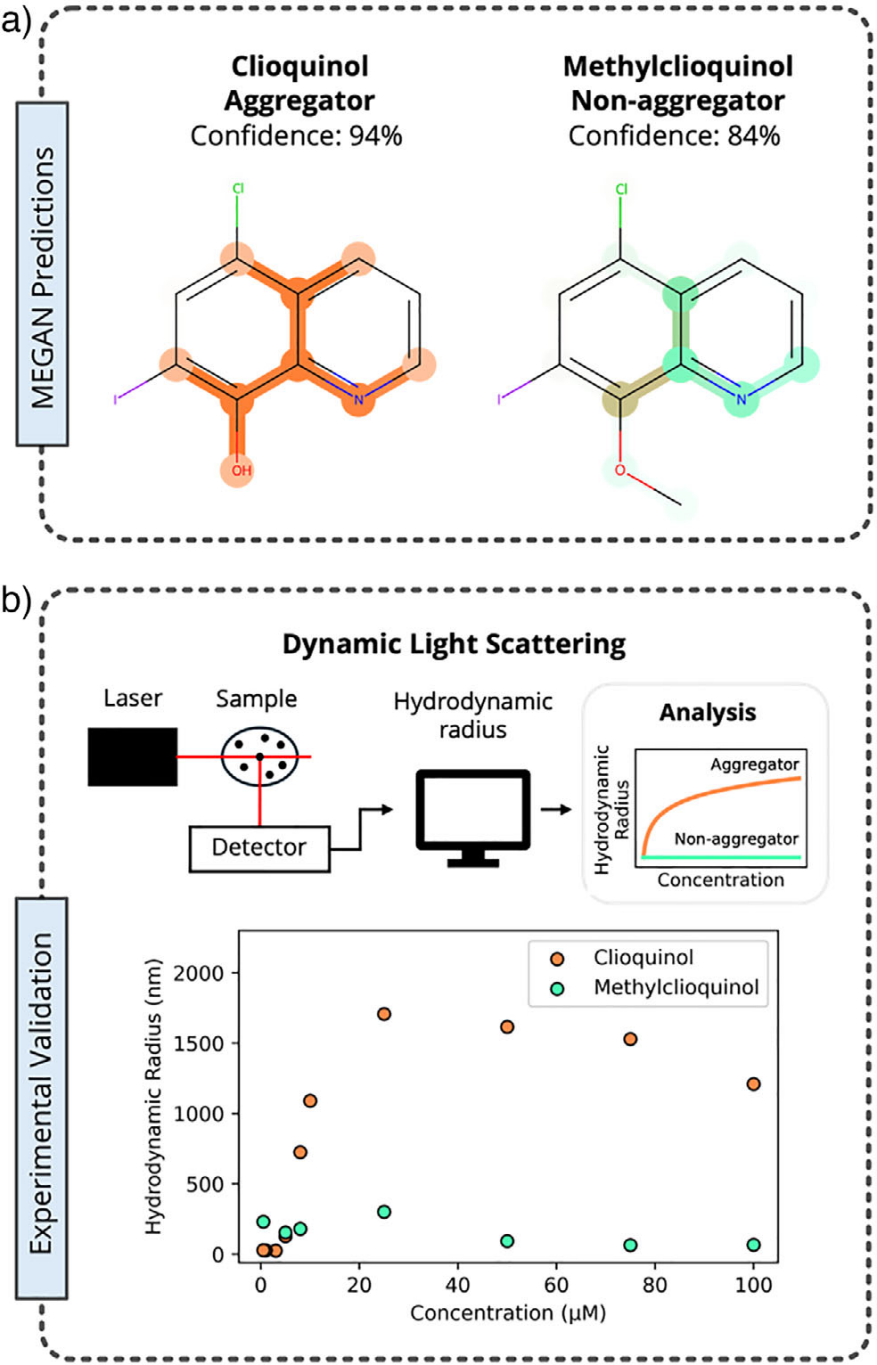
a) MEGAN model's prediction confidence and explanation masks for clioquinol and methylclioquinol. b) Overview of DLS method and experimental DLS results for clioquinol (orange) and methylclioquinol (green).

We investigated clioquinol, which has been previously reported to aggregate and is correctly predicted by the MEGAN model, and its flipped label counterfactual, methylclioquinol, which has never previously been experimentally examined for its aggregation properties (Figure 6). To compare the aggregation behavior of these two molecules, dynamic light scattering (DLS) experiments were performed for each compound. For the DLS experiments, solutions of methylclioquonol and clioquinol at concentrations of 100, 75, 50, 25, 10, 8, 5, 3, 1 and 0.5 µM from were prepared by dilution of a 1 mM stock solution of the respective compound in DMSO with a 40 mM sodium phosphate buffer at pH 7.4.

Consistent with previous reports, clioquinol was found to aggregate, as demonstrated by a significant increase in its hydrodynamic radius at higher concentrations. In contrast, methylclioquinol exhibited minimal fluctuation in its hydrodynamic radius, indicating that aggregation does not occur in the concentration range studied. These findings highlight the potential power of the MEGAN model's ability to aid in the rational design of molecules with tailored aggregation properties, offering a versatile approach for optimizing compound behavior in biological systems while preserving critical structural features.

To understand the model's rationale for suggesting methylclioquinol as a counterfactual, the explanation masks for both molecules were examined. The explanation masks for clioquinol highlight the majority of the quinoline scaffold, with a focus on the area containing the nitrogen and alcohol, as providing evidence for the aggregator class. In contrast, for methylclioquinol, the methoxy group as well as the quinolin scaffold are both highlighted, albeit to a lesser extent, as providing evidence for the non‐aggregator class. The methylation of clioquinol at the oxygen atom eliminates its ability to engage in hydrogen bonding interactions. These findings suggest that the model recognizes hydrogen bonding motifs and the steric bulk introduced by substituents as key factors influencing aggregation.

### External Validation

To further validate the predictive accuracy of the MEGAN model, a dataset of 58 structurally diverse drugs that have been experimentally characterized as aggregators or non‐aggregators was compiled and assessed using the model. This external validation dataset was sourced from two literature references, where the compounds had been experimentally screened to evaluate the impact of colloidal aggregation on SARS‐CoV‐2 drug repurposing efforts.^[^ ^27^, ^28^ ^]^ The dataset includes 30 aggregating and 28 non‐aggregating compounds, none of which were present in the training or test sets for the MEGAN model, ensuring that they were previously unseen by the model.

The MEGAN model was capable of correctly classifying 15 of the 30 aggregating compounds and 24 of the 28 non‐aggregating compounds, resulting in an accuracy of 68%. For comparison, we evaluated the performance of CHEMAgg, a widely recognized model for aggregation prediction, using the same validation dataset. CHEMAgg achieved an accuracy of only 50%, significantly lower than the MEGAN model. Although CHEMAgg accurately identified 27 of the 28 non‐aggregating compounds, it only correctly classified 2 of the 30 aggregating compounds. CHEMAgg's classification of 55 of the 58 molecules as non‐aggregators suggests a strong bias in this model toward non‐aggregator classification. In contrast, the MEGAN model provides a more balanced classification and demonstrates superior performance in accurately predicting molecular aggregation. This result highlights the ability of our model to outperform existing state‐of‐the‐art models, particularly in identifying aggregating compounds, which is a critical capability for accurately screening datasets for promiscuous molecules.

## Conclusion

The MEGAN model presented in this study demonstrates a high level of accuracy for predicting SCAMs, achieving 82% on a balanced in‐distribution test dataset and similar performance on a smaller external dataset of drug molecules. Although commonly accepted features such as logP values and the number of aromatic rings are relevant for detecting SCAMs, the model's high accuracy and explanations indicate that it learns more complex relationships between molecular structure and aggregating behavior.

Through local and global model explanations and counterfactual analysis, we identified and systematically examined specific molecular motifs associated with aggregation. By combining expert knowledge, quantum mechanical modeling, and automated interpretations using large language models, we provide deeper insights into molecular aggregation. The MEGAN model not only highlights molecular motifs strongly linked to aggregation but also identifies small structural modifications that can significantly alter aggregating behavior, providing valuable tools for molecular design.

The MEGAN model's accuracy, combined with the accessibility and extended functionality provided through a publicly available web interface, enables improved detection of SCAMs. Furthermore, the use of molecular counterfactuals offers a practical approach for designing alternatives to problematic compounds. This capability facilitates the filtering of HTS libraries, helping to reduce false positives in drug discovery databases and improving overall screening reliability.

## Supporting Information

The authors have cited additional references within the Supporting Information.^[^ ^29^, ^30^, ^31^, ^32^, ^33^, ^34^, ^35^, ^36^, ^37^, ^38^, ^39^, ^40^, ^41^, ^42^, ^43^, ^44^, ^45^, ^46^, ^47^ ^]^

## Conflict of Interests

The authors declare no conflict of interest.

## Supporting information



Supporting Information

## Data Availability

The data that support the findings of this study are openly available in GitHub at https://github.com/DavisGroup/MEGAN‐aggregation‐data. The full code availability statement can be found in the Supporting Information.
