# Supplementary material for: Mitigating Molecular Aggregation in Drug Discovery With Predictive Insights From Explainable AI
Source: Angew Chem Int Ed Engl. 2025 Jun 1;64(29):e202503259. doi: 10.1002/anie.202503259 (PMC12258689; doi:10.1002/anie.202503259)
Supplement: Supplementary file 1 — Supporting Information [file ANIE-64-e202503259-s001.pdf]

Supporting Information

Mitigating Molecular Aggregation in Drug Discovery with Predictive Insights from Explainable AI

Hunter Sturm, Jonas Teufel, Kaitlin A. Isfeld, Pascal Friederich, Rebecca L. Davis

SI. Methods **2**
SII. Model performance comparison **6**
SIII. Global concept extraction report **7**
SIV. Examples of correct model predictions for molecule pairs with small structural changes **8**
SV. Impact of training set imbalance and test set imbalance on accuracy, precision, and recall **9**
SVI. Energy decomposition analysis of dimers of E-P **11**
SVII. Determination of relative frequency of thiourea fragments for all compounds in the aggregator and non-aggregator datasets from SMARTS pattern **14**
SVIII. Correlation of explanation masks and atomic contributions of simple molecular descriptors **15**
SIX. Automated generation of explanations using large language models **18**
SX. Dataset structural feature and property analysis **20**
SXI. List of all RDKit molecular descriptors calculated for all compounds in the aggregator and non-aggregator datasets **23**
SXII. Histograms of normally distributed general molecular descriptors for the aggregator and non-aggregator datasets **24**
SXIII. Histograms of non-normally distributed general molecular descriptors for the aggregator and non-aggregator datasets **30**
SXIV. Histograms of fragment molecular descriptors for the aggregator and non-aggregator datasets **34**
SXV. p-values, means, standard deviations and Cohen's d values for the 15 statistically significant general molecular descriptors calculated for the aggregator and non-aggregator datasets **63**
SXVI. Comparison of the relative frequency of each of the fragments described by the fragment descriptors for the aggregator and non-aggregator molecules **64**
SXVII. DFT energies and coordinates for pyridine derivatives E-P, monomers **107**
SXVIII. DFT energies and coordinates for pyridine derivatives E-P, Dimers **119**
SXIX. 1H and 13C NMR spectra for methylclioquinol **131**

SI. Methods

Dataset curation and preparation

For model training, a dataset of molecules that have been experimentally validated as either aggregating or non-aggregating was curated. In order to do so, data from the Shoichet laboratory utilized for the development of their Aggregator Advisor tool was used.[9] For the development of Aggregator Advisor, Stoichet et al. identified over 12,600 likely aggregators from their previously published experimental screens for colloidal aggregation. These compounds were used as our dataset of experimentally validated aggregators. To curate our dataset of non-aggregating molecules, one of the libraries screened for aggregation by Stoichet consisting of approximately 198,000 compounds was filtered to exclude any molecules in our dataset of aggregating molecules.[29] As the 198,000 compound dataset was utilized to populate the 12,600 aggregators in Aggregator Advisor, any compound in the dataset that was not identified by Stoichet as an aggregator has been experimentally validated as a non-aggregator.

Upon data curation, the resulting datasets consisted of 12,607 aggregating and 197,846 non-aggregating molecules represented as SMILES strings. In order to clean the data for use in training our machine learning model, the SMILES strings were canonicalized using RdKit.[30] If multiple fragments were present in the SMILES strings, the longest one was kept and the others (generally counterions or solvents) were removed. If there was more than one long (>12 characters) fragment, the data point was discarded. All duplicate molecules and molecules producing RdKit errors were also discarded from the datasets. Additionally, for the non-aggregating dataset, molecules containing metal atoms and inorganic molecules (i.e. containing zero carbon atoms were discarded. After filtering the molecules, the remaining datasets consisted of 12,338 aggregating and 177,048 non-aggregating molecules. Molecular descriptors were analyzed to examine the differences in structural features and properties of the compounds in the aggregating and non-aggregating datasets (see section SX of Supporting Information).

For the training of the graph neural network, the dataset was first pre-processed using the publicly available protonation software library DimorphiteDL to extract all possible protonation states at physiological pH for each molecule.[31] A value of 7.4 was used for both the min_ph and max_ph parameters of the DimorphiteDL software. The protonated molecule variants were then converted into graph representations using RDKit to extract numeric node and edge attribute vectors.[30] Node features include a one-hot representation of the atom type, as well as additional information such as the number of connected hydrogen and local charge distribution. Similarly, the edge features contain a one-hot encoding of the bond type and additional information such as whether the bond is part of a ring structure.

In case users are interested in different conditions than the ones listed above, fine-tuning of the trained model depending on conditions can be achieved in multiple ways: If condition information is available for all data points in a new dataset, then the information can be used as global information in the GNN model or as a condition on the prediction. In that way, during test time, condition-dependent prediction can be generated by the model. Alternatively, if conditions are only known for a subset of the datapoints, a general base-model can be trained with all datapoints and then fine-tuned for the subset of datapoints with desired target conditions.

Explainable graph neural networks, MEGAN

The Multi-Explanation Graph Attention Network (MEGAN) is a graph neural network designed to make predictions while also explaining how it arrived at those predictions. Unlike many other models, MEGAN can provide multiple explanations for each prediction, such as highlighting features that contribute positively or negatively. It uses attention mechanisms to focus on important parts of the graph and a self-consistent, masking-based training process to ensure that its explanations align with the expected meanings. This makes MEGAN especially useful for tasks like predicting molecular properties, where understanding the role of specific substructures is critical.

Soley relying on attention masks to generate explanations has been demonstrated to lead to misleading results. For that reason, the MEGAN model[19] additionally aggregates attention scores across all layers to perform an attention-weighted graph pooling. This means that the global pooling step before the final regression MLP is weighted using the aggregated attention scores, which in this way receive additional training signal from the graph labels. We do this to ensure the faithfulness of the generated explanations: Only information from nodes with high attention values is used for the final prediction outcome. More information can be found in Teufel et al.[19] To ensure an interpretable alignment of the generated attention masks with human expectations, the training process also explicitly implements a semi-supervised explanation loss. This training loss promotes the model to approximately solve the primary classification task using only the explanation masks themselves. In this case, the "Aggregator" explanation channel, for example, is trained to highlight explanations only for true "Aggregator" elements. This effectively forces the model to recognize and highlight those substructures which occur more often in elements belonging to one class versus the other.

As demonstrated in Teufel et al.[19], the MEGAN model beats common graph explanation methods such as GNNExplainer and GNES in explanation accuracy, sparsity, and fidelity when trained on dedicated benchmark datasets with ground truth explanations. At the same time, the model matches the performance of other graph neural networks such as GAT, GIN, Schnet, and PAiNN in molecular regression and classification tasks. Thus, the MEGAN model is currently on the Pareto front of accuracy and explainability.

More details on the concrete implementation and benchmarks can be found in Teufel et al.[19]. An interactive interface to query the MEGAN model for aggregation prediction is available at https://megan.aimat.science/predict/megan_aggregator. Code and data are available on GitHub (See Data and Code Availability).

Graph Representation

To obtain a prediction for a given molecule using the aforementioned MEGAN graph neural network, the molecule first has to be converted into a graph structure. In this graph structure, each node (atom) is represented by a numeric node feature vector and each edge is represented by a numeric edge feature vector. We construct the node feature vector by concatenating the following information:

- A one-hot representation of 15 common atom types, including carbon, nitrogen, oxygen, sulfur, chlorine, fluorine as well as a special encoding for “other” atom types taht are not explicitly included.
- Separate one-hot encoding of atom’s hybridization state, its total degree and the total number of attached hydrogens.
- A single binary flag to indicate whether or not the atom is part of an aromatic ring.
- Continuous values for the atom’s mass and it’s formal charge.
- Individual atom contributions of the molecular descriptors LogP, TPSA and LabuteASA.

Likewise, we construct the edge feature vector by concatenating the following information:

- A one-hot encoding of the bond type and the stereo state of the bond.
- A single binary flag to indicate whether or not the bond is part of aromatic ring.

The conversion as well as the computation of the node and edge features is implemented using the RDKit software package which creates the molecular graph from its corresponding SMILES representation. The pre-processing and feature computation takes approximately 0.78±0.16 milliseconds (averaged over 1000 elements) and can therefore easily be considered real-time.

Counterfactuals

Counterfactuals are generated by exploring the immediate graph neighborhood of each original molecule and then selecting those that result in the highest prediction difference relative to the original prediction. We generate the local neighborhood of the molecular graphs with a procedure inspired by Riley et al. in which all chemically feasible atom and bond insertions and deletions are applied to the molecule recursively.[32] Depending on the recursion depth, this procedure typically produces 100-1000 perturbed graphs out of which we present the 10 graphs with the highest prediction difference as counterfactual explanations.

Fingerprint and descriptor-based ensemble methods

To compare the accuracy of our model to the aggregation prediction model reported by Yang et al., we re-implemented their computational approach and trained it on the dataset presented in this work. We used three types of descriptors, namely fingerprint features (circular fingerprints as well as ECFP4/Morgan fingerprints as implemented in RdKit), MACCS features as implemented in RdKit, and CATS features as implemented in https://github.com/alexarnimueller/cats-descriptor.[33-35] Within each set of descriptors, we used a feature importance analysis based on a Random Forest classifier to determine how many features are required to achieve the highest accuracy on the validation set. This resulted in a total selection of 33 features. After concatenation and hyperparameter optimization, we found the optimal hyperparameters of the Random Forest classifier to be an ensemble size of 500, with no limit on depth, and entropy as an impurity measure; of the Gradient Boosting classifier to be an ensemble size of 500, a maximum depth of 9, and a learning rate of 0.5; and of the xGBoost model to be an ensemble size of 500, a maximum depth of 8, and a learning rate of 0.1.

DFT modeling of interaction energies

DFT modeling of pyridine derivatives **E - P** was performed to assess the energetic favorability of the intermolecular interactions formed between compounds as well as the favored geometries of the dimers. To locate the lowest energy geometries of the monomers and dimers, initial geometries were constructed, and conformer ensembles were generated using CREST based on GFN2-xTB calculations.[36,37] The lowest energy monomer and dimer geometries were then optimized using wB97xD/def2-TZVP with the SMD solvent model (H_2O) in Gaussian 16.[38-41] Vibrational analysis performed at 273.15 K confirmed that all optimized geometries corresponded to minima on the potential energy surfaces, as indicated by the absence of imaginary frequencies. See section SXVII and SXVIII of the Supporting Information for coordinates and energies of optimized geometries. The interaction energies were calculated following Eq. S1 using the sum of the electronic energy (E) and zero-point correction to the electronic energy (E_{ZPE}). In all cases, the most energetically favorable geometry of the dimer that could be located was used for the calculation of the interaction energy.

$$\text{Interaction Energy} = (E_{dimer} + E_{ZPE,\ dimer}) - 2(E_{monomer} + E_{ZPE,\ monomer}) \quad \text{(Eq. S1)}$$

Dynamic light scattering experiments

DLS was used as an aggregation detection method for the examination of clioquinol and methylclioquinol. The instrument used in this study was a Nanotemper Prometheus Panta and standard 10 µL capillaries were used. Clioquinol was purchased from TCI chemicals via Fisher Scientific, and methylclioquinol was prepared and purified following standard methylation conditions using iodomethane (see section SXIX of the Supporting Information for NMR spectra).[42] Stock solutions of 1 mM were prepared for each compound in DMSO. The stock solutions underwent serial dilution using a 40 mM sodium phosphate buffer at pH 7.4 to prepare solutions of 100, 75, 50, 25, 10, 8, 5, 5, 3, 1, and 0.5 µM. The concentrations tested were chosen based on the concentrations that standard HTS screens are run at. Each solution was run in triplicate on the instrument, and aggregation was determined based on hydrodynamic radius.

Data and code availability

Our code for model training and counterfactual analysis can be found on Github https://github.com/aimat-lab/megan_aggregators. In our repository, we also include a persistent representation of the already trained model, which can be used directly. The full dataset can be downloaded at https://bwsyncandshare.kit.edu/s/4r9kgyCFQL6PTcF. The cleaned aggregator and non-aggregator datasets, external validation dataset and ^{1}H and ^{13}C NMR spectra can be downloaded at https://github.com/DavisGroup/MEGAN-aggregation-data. Furthermore, we provide an interactive web interface for the manual prediction of the aggregation behavior of single molecules at https://megan.aimat.science/predict/megan_aggregator. Given a SMILES representation of a molecule, the interface shows the predicted classification, visualization of local explanations, and the top counterfactuals.

SII. Model performance comparison

The performance of the MEGAN model was compared against the XGboost model of Yang et al. (ChemAgg), using our balanced test set.[11] The results in **Table S1**, in particular a comparison of Entries 1 and 2, show that the dataset presented here (or at least the balanced test split chosen here) is more difficult to predict than the dataset presented in Yang et al.[11] However, when comparing the performance of the feature based XGBoost model used in Yang et al. (Entry 2) with the MEGAN model (Entry 3), we see a clear advantage of the graph neural network. This can be due to the fact that the model input for the graph neural network is "complete" in a sense that the full molecular structure is modeled, which is not the case for the fingerprint and feature representations, or it can be related to the higher complexity and expressiveness of graph neural networks. Similar trends of superior performance of graph neural networks compared to classical machine learning models can also be seen with many other datasets of molecules and materials of similar size.[43]

Table S1. Accuracy and F1 score of aggregation classifiers from literature (Yang et al.[11]), compared to our data and our MEGAN model.

Entry	Model	Data	Accuracy (test)	F1 score (test)
1	Yang et al.	Yang et al.[1]	0.937	0.899
2	Yang et al.[2]	Ours (balanced test set)	0.733	0.735
3	Ours (MEGAN)	Ours (balanced test set)	0.818	0.807

[1] The dataset used in Yang et al. is not published, which is why we could not reproduce the experiments with our model on their dataset.

[2] The code used in Yang et al. is not published, so we used a re-implementation based on the methodology described in Yang et al. However, not all 5 sets of descriptors could be re-implemented, which is why we used a subset of 3 sets of descriptors (see Methods in section SI of Supporting Information). The best performance was achieved with a XGBoost model but the performance of random forest models and gradient boosting models was almost identical.

SIII. Global concept extraction report

Global concept extraction report is located at https://github.com/DavisGroup/MEGAN-aggregation-data

SIV. Examples of correct model predictions for molecule pairs with small structural changes

Additional examples of aggregation cliffs (i.e., where small structural changes flip the label from aggregator to non-aggregator or vice versa) in the training/test data have been identified and select examples are provided below. However, it should be noted that these represent a small sample of hand-picked aggregation cliffs out of the training and test set and it is highly likely given the oversampling method used for the aggregator class during model training that the model will miss many aggregation cliffs. We were unable to further validate our model's sensitivity on unseen sets of molecules exhibiting aggregation cliffs as no additional examples could be found in the literature.

**Experimentally Known Non-Aggregators**	**Experimentally Known Aggregators**
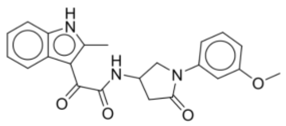 Predicted Non-Aggregator: 0.93 Confidence	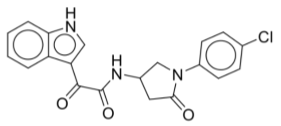 Predicted Aggregator: 0.60 Confidence
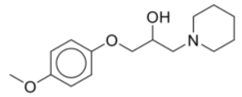 Predicted Non-Aggregator: 0.84 Confidence	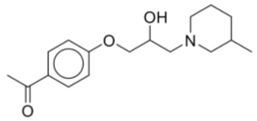 Predicted Aggregator: 0.91 Confidence
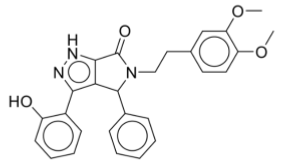 Predicted Non-Aggregator: 0.69 Confidence	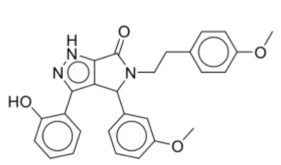 Predicted Aggregator: 0.92 Confidence
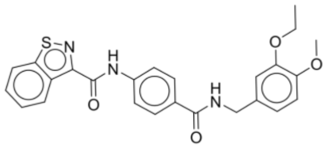 Predicted Non-Aggregator: 0.63 Confidence	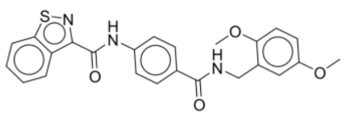 Predicted Aggregator: 0.87 Confidence

SV. Impact of training set imbalance and test set imbalance on accuracy, precision, and recall

As the dataset is highly imbalanced, we explicitly used oversampling to address the class imbalance, which is a common practice in such cases. This means that in each training epoch, we reused some of the non-aggregating molecules multiple times in order to have the same number of training instances with both label types.
For the evaluation of our model, we specifically constructed a balanced test set consisting of 500 randomly sampled aggregators and 500 randomly sampled non-aggregators that were not used during training. On this balanced test set, we report an accuracy of approximately 82%. However, we can still observe a slight imbalance in the prediction performance. The confusion matrix below shows that the model more often mispredicts true aggregators as non-aggregators than vice versa. This indicates that it is generally more biased to predict non-aggregators, which is most likely a consequence of the (training) dataset imbalance.

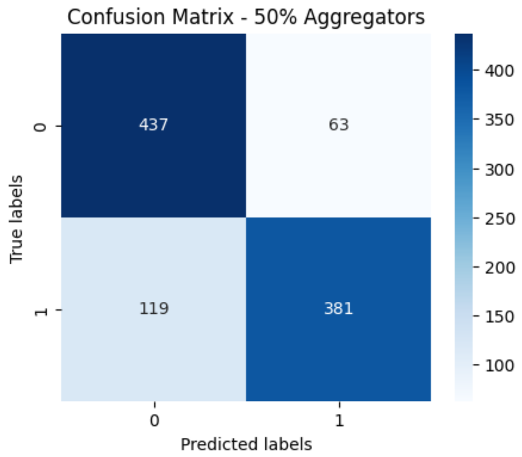

The plot below shows the confusion matrix when artificially reducing the ratio of aggregators by subsampling, creating a test set with high label imbalance (5 aggregators and 500 non-aggregators).

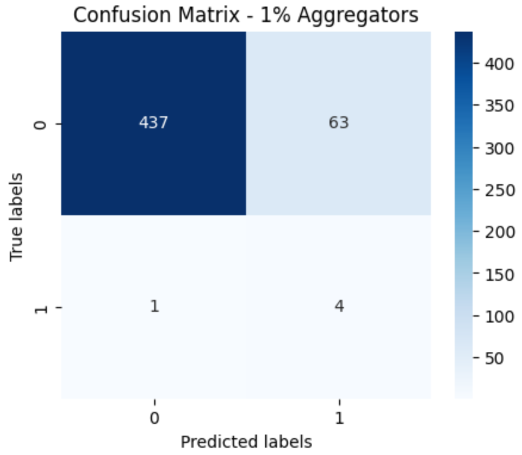

This case illustrates that (with the given threshold) the model has high recall, meaning a high capability of detecting (true) positives. Out of 5 true aggregators in the imbalanced test set, 4 are correctly identified. However, the model has a rather low precision, so there are many false positives as well, which is to be expected even for a well-trained model when tested on a highly imbalanced dataset. The Bayesian probability that a molecule actually is an aggregator, given that the train model predicts that it is an aggregator, i.e. the precision of the model, is dominated by the low overall probability of the aggregator label, so even a very well-trained model with high accuracy and recall have a low precision due to the high intrinsic ground truth label imbalance resulting in a low value of P(agg):

$$precision = P(agg \mid model\ predicts\ agg) = \frac{P(model\ predicts\ agg \mid agg) * P(agg)}{P(model\ predicts\ agg)} \propto recall * P(agg)$$

In agreement with the equation above, the explicit dependence of accuracy, precision, and recall as a function of test set (im)balance can be found below. While the accuracy slightly increases with increasing test set imbalance, the precision linearly drops (at a nearly constant recall).

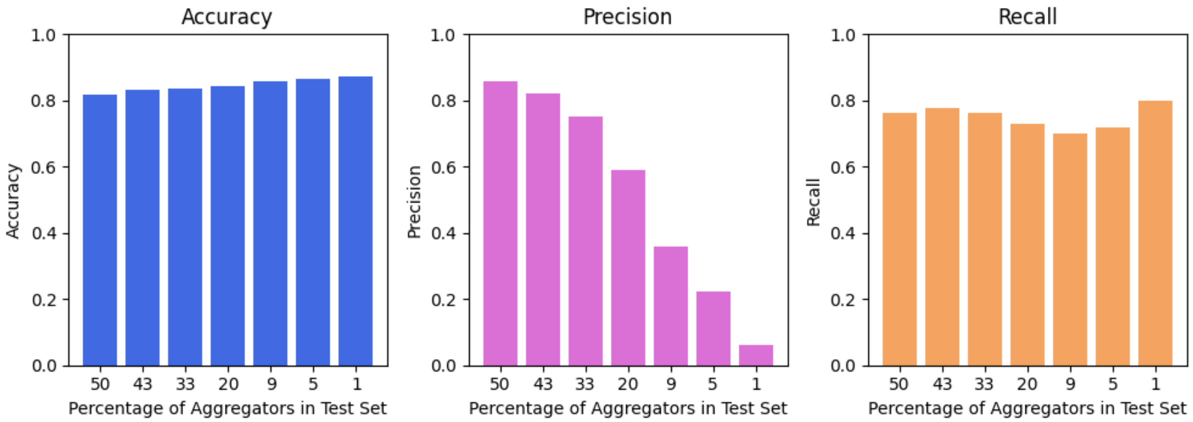

SVI. Energy decomposition analysis of dimers of E-P

For pyridine derivatives **E – P**, energy decomposition analysis (EDA) was completed to further evaluate the trend between aggregation prediction confidence and the dimer interaction energies (computational methods below). For the EDA, SAPT0 calculations were performed using Psi4 to decompose the dimers interaction energies (E_{int}) into the sum of four physically relevant energy terms: electrostatics (E_{elec}), exchange (E_{exch}), induction (E_{ind}), and dispersion (E_{disp}) (Eq. S2).

$$E_{int} = E_{elec} + E_{exch} + E_{ind} + E_{disp} \qquad \text{(Eq. S2)}$$

Each of these energy terms and the non-covalent interactions they represent are described in greater detail in the literature. [23, 24, 44] However, the terms can be briefly summarized as follows: the electrostatic interaction term describes the energy of interactions between permanent multipoles on each molecule. The exchange interaction term (sometimes called exchange repulsion) describes the energy needed to be overcome for tight molecular packing, and is often destabilizing. The induction term describes the energy of the induced electrostatic moments of one molecule interacting with the permanent electrostatic moments of another. Finally, the dispersion term describes the energy of interactions between induced dipoles.

The results of the EDA analysis for the pyridine derivative dimers are reported in Figure S1 and Table S2. These results show the total SAPT0 energy (i.e. the interaction energy of the two molecules forming the dimer (E_{int}) decomposed into its four contributing energy terms. For all dimers, E_{exch} was found to be destabilizing, whereas E_{elec}, E_{ind} and E_{disp} were stabilizing.

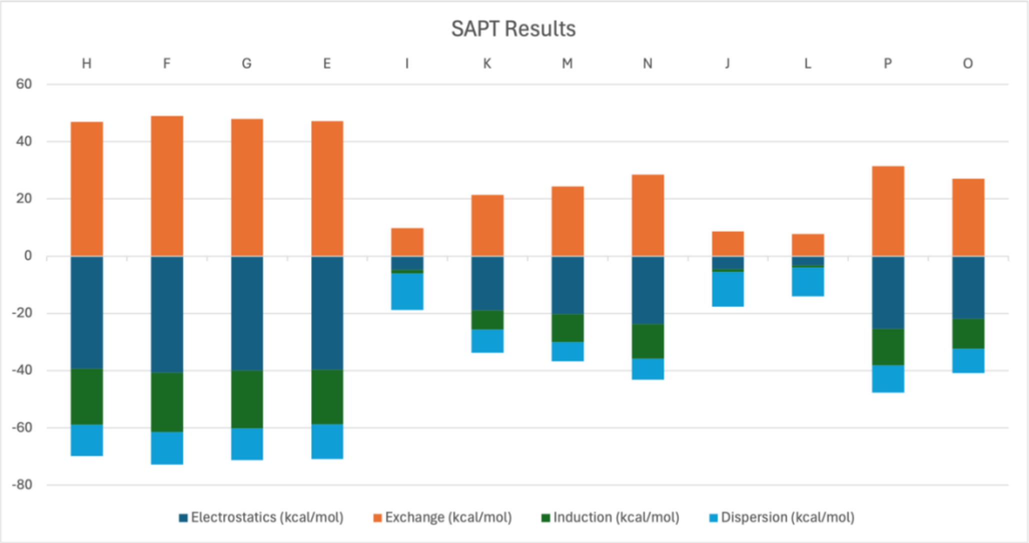

Figure S1. SAPT0 energy decomposition analysis results for dimers of **E - P**. Sorted from left to right based on highest to lowest aggregator prediction confidence.

Table S2. SAPT0 energy decomposition analysis results for dimers of **E** - **P**, sorted by aggregation prediction confidence. All energies are reported in kcal/mol.

Dimer	Aggregation Prediction	E_{elec}	E_{exch}	E_{ind}	E_{disp}	E_{int}
H	0.94	-39.29	46.87	-19.56	-10.99	-22.96
F	0.92	-40.72	48.99	-20.75	-11.36	-23.84
G	0.88	-40.00	48.02	-20.23	-11.03	-23.23
E	0.86	-39.67	47.17	-19.10	-12.07	-23.67
I	0.12	-4.82	9.76	-1.29	-12.67	-9.02
K	0.09	-18.89	21.38	-6.75	-8.11	-12.36
M	0.07	-20.25	24.36	-9.78	-6.75	-12.41
N	0.07	-23.64	28.44	-12.16	-7.32	-14.69
J	0.06	-4.52	8.61	-1.00	-12.07	-8.97
L	0.06	-3.15	7.72	-0.81	-10.09	-6.32
P	0.05	-25.35	31.47	-12.80	-9.47	-16.15
O	0.05	-21.84	27.08	-10.43	-8.55	-13.74

Analysis of the magnitude of E_{elec}, E_{exch}, E_{ind} and E_{disp} for each dimer revealed three distinct groups. Group 1 consists of the dimers of all of the predicted aggregators (**E**, **F**, **G** and **H**), group 2 consists of the dimers of predicted non-aggregators **K**, **M**, **N**, **O** and **P**, and group 3 consists of the dimers of predicted non-aggregators **I**, **J** and **L**. Interestingly, the molecules of group 1 each form two hydrogen bonds in their dimers, the molecules of group 2 each form one hydrogen bond in their dimer with the exception of the dimer of **K** which forms 2 hydrogen bonds, and molecules of group 3 are those exhibiting pi-stacking interactions.

For group 1 (dimers of the predicted aggregators **E**, **F**, **G** and **H**) the destabilizing exchange energy (E_{exch}) had the largest magnitude out of the four energy terms. Of the three stabilizing interactions, E_{elec} had the largest magnitude. This term would encompass the two favourable hydrogen bonding interactions present in each of the dimers of this group. The second most stabilizing energy term for dimers of molecules of this group was E_{ind}, followed by E_{disp}.

Group 2 (dimers of the predicted non-aggregators **K**, **M**, **N**, **O** and **P**) demonstrated the same energy trends as dimers in group 1. E_{exch} was destabilizing and had the largest magnitude while the three stabilizing interactions (from most to least favourable) were E_{elec}, E_{ind} and E_{disp}. Interestingly, for group 2, each of the four energy terms were roughly half the magnitude of the energies for dimers of group 1. This fits with the observation that dimers of both groups interact through hydrogen bonding, and that dimers of group 1 form two hydrogen bonds whereas dimers of group 2 form only one hydrogen bond (with the exception of the dimer of **K**). While **K** forms two hydrogen bonds in its dimer, the weaker nature of the N-H-N hydrogen bonding

interactions in this dimer, relative to the more polarized O-H-N hydrogen bonding interactions present in dimers of **E-H,** matches the observed trend. The decreased strength of the N-H-N hydrogen bonding can be seen in the smaller electrostatic and induction terms in **K** relative to **E-H**.

Group 3 (dimers of the predicted non-aggregators **I**, **J**, and **L**) was found to have the smallest SAPT0 interaction energies and showed a different trend compared to groups 1 and 2. In group 3, E_{disp} was found to have the greatest magnitude and for all three dimers of this group was larger than the destabilizing exchange energy term. For this group, E_{ind} was the least stabilizing energy term preceded by E_{elec}. This was an interesting, yet unsurprising trend as the dimers of **I**, **J**, and **L** were the only dimers in our study that did not participate in hydrogen bonding and instead participate in pi-stacking interactions, explaining why E_{disp} had the largest magnitude of the energy terms. Overall, the energetic decomposition of the interaction energies of the dimers of the pyridine derivatives showed interesting trends and were consistent with the trends provided by the DFT calculations reported in the main text.

<u>EDA Computational Methods:</u> For SAPT0 calculation setup with Psi4, the default flags were used in all cases, except for the basis flag which was set to def2-TZVP and the freeze_core flag which was set to TRUE. The coordinates of the DFT optimized pyridine dimer geometries were used for the EDA (see section SI of the Supporting Information for DFT methods).

SVII. Determination of relative frequency of thiourea fragments for all compounds in the aggregator and non-aggregator datasets from SMARTS pattern

In order to determine the number of thiourea fragments present in each compound in the aggregator and non-aggregator datasets, the SMILES strings for the molecules in each dataset were searched for the thiourea substructure using RDKit. The thiourea substructure was defined using the SMARTS pattern [NX3][CX3](=[SX1])[NX3] and the search was performed on the SMILES strings for the aggregator and non-aggregator datasets which had been protonated to protonated to the most energetically favourable ionization state at physiological pH (pH=7.4) using the FixpKa functionality of OpenEye's QUACPAC 2.1.2.1.[45]

In the non-aggregator dataset, 4,268 molecules were found to contain one thiourea group and 37 molecules were found to contain between two and four thiourea groups. In the aggregator dataset, 334 molecules contained one thiourea group and 1 molecule contained two thiourea groups. The relative frequencies of the thiourea substructure in each dataset is illustrated below. As the relative frequency of molecules containing more than one thiourea group is negligible, it has been excluded from the image below.

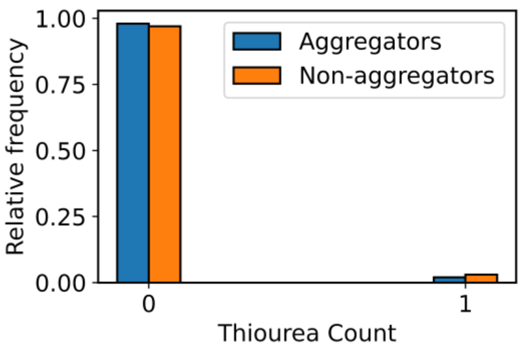

SVIII. Correlation of explanation masks and atomic contributions of simple molecular descriptors

We conducted a feature importance analysis for the given aggregation classification task and find that several classic molecular descriptors show a weak separability between the aggregator and non-aggregators classes, i.e. they have slightly different (shifted) distributions when comparing their values for the aggregating and the non-aggregating samples. Notably, we find MolLogP to be the most important feature, but also find MolMR (Wildman-Crippen molar refractivity value - accounting for molecular size and polarizability) and LabuteASA (Labute's Approximate Surface Area) to be important as well[1].

These findings are reinforced by Figure S2, which show the slightly differing distributions for the three previously mentioned properties between the two ground truth classes in the test dataset. However, neither of these simple descriptors alone is capable of sufficiently separating the classes on their own, as indicated by the high distributional overlap coefficients of >0.75.

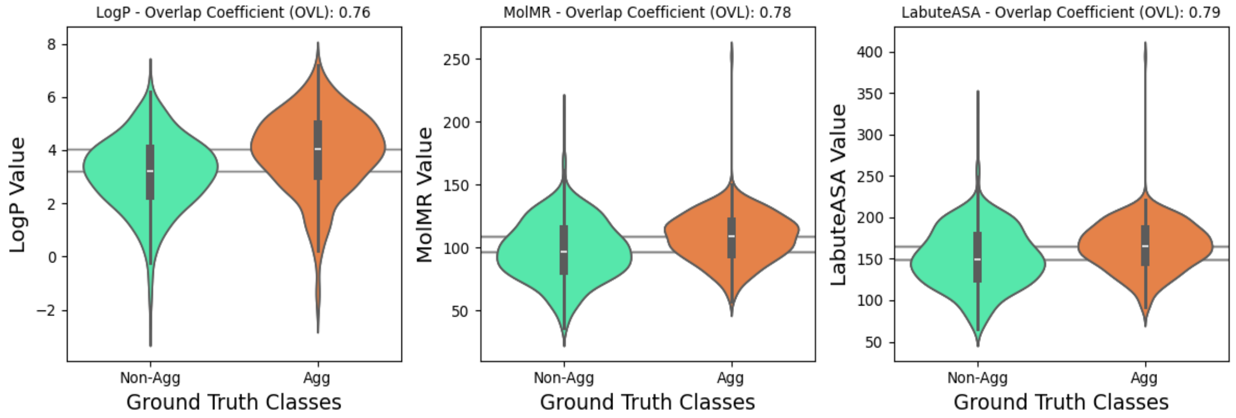

Figure S2. Class-separated distributions of three influential molecular descriptors for the 1000 elements of the test set. Non-Aggregator distributions are shown in green and Aggregator in orange. The three molecular descriptors LogP, MolMR and LabuteASA (left to right) have previously been identified as especially influential during a feature importance analysis. For each molecular descriptor, the overlap coefficient provides a measure of overlap between the two classe's distributions where higher values indicate lower separability.

In this context, it is important to emphasize that while simple molecular descriptors certainly provide some foundation for the model's explanations, they only contribute a small fraction to the overall class separability.

In contrast, as illustrated in Figure S3, the distribution of the MEGAN model's output logits shows a much higher capability of separating the two ground truth classes with a distributional overlap coefficient of only 0.34.

[1] We find TPSA to have little capability in distinguishing between aggregators and non-aggregators, hence we did not include it in this discussion.

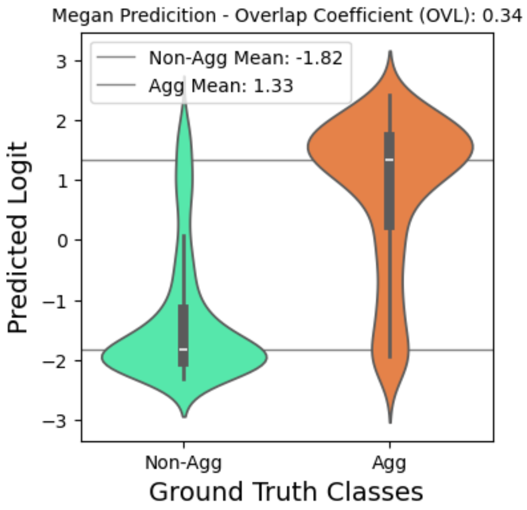

Figure S3. Class-separated distributions of the of MEGAN's prediction logits for the 1000 elements of the test set. Non-Aggregator distributions are shown in green and Aggregator in orange. The overlap coefficient provides a measure of overlap between the two classe's distributions where higher values indicate lower separability.

In light of this presumption, we have conducted a quantitative analysis to compare the MEGAN model's atom-contribution-based explanation masks with the decomposed atom contributions available for the LogP, MolMR, and LabuteASA properties. For each molecule in the test set, we compute the Spearman correlation coefficient between the explanation mask and the atom contributions. Figure S4 shows the average over these individual correlation coefficients for both of the model's explanation channels, respectively. We find that the "non-aggregator" explanations are anticorrelated with atomic logP contributions and that the "aggregator" explanations are correlated with the MolMR contributions. This means that the parts of molecules indicated by the model as non-aggregating have, on average, lower logP scores and thus higher water-solubilities, which makes intuitive sense. It also means that the parts of molecules indicated by the model as aggregating contribute more to the molecular polarizability, potentially related to aromatic systems, which have high polarizability values and at the same time promote stacking and aggregation. In terms of the LabuteASA contributions, we find both "aggregator" and "non-aggregator" explanations to be inversely correlated, albeit on different populations of molecules.

Ultimately, for this analysis, it is again important to emphasize that these are small correlation values with rather large standard deviations, indicative of relatively small effects. However, in continuation of the previous argument, no individual simple descriptor can be expected to fully explain aggregation considering their relatively small individual contributions to class separability.

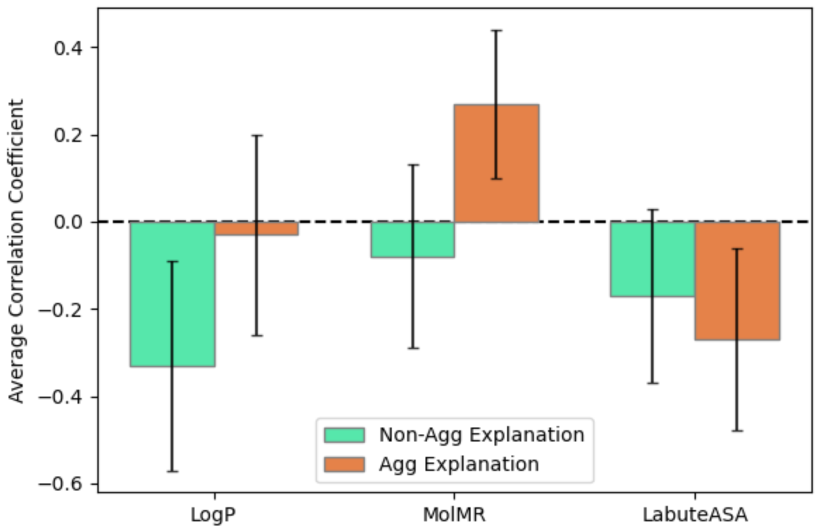

Figure S4. Distribution of spearman correlation coefficient of MEGAN's explanation masks and the atom contributions of the three molecular descriptors LogP, MolMR and LabuteASA. Non-Aggregator distributions are shown in green and Aggregator in orange. The extent of bar plots indicates the average spearman coefficient over the 1000 elements of the test set and error bars indicate the corresponding standard deviation.

SIX. Automated generation of explanations using large language models

To analyze whether the explanations generated based on the MEGAN model can be interpreted in terms of structural and physicochemical properties in an automated way, we experimented with large language models, specifically GPT-4o. The goal was to see whether the graph explanations can be automatically related to more broad chemical concepts and human-understandable explanations, in order to stimulate further ideas for more detailed analysis by experts and to potentially derive design rules in an automated way.

Prompts were created with the following template, which was adapted from OpenAI's examples:

> "[SYSTEM PROMPT]: You are a chemistry expert with the task of proposing possible hypotheses about the underlying structure-property relationships of molecular properties. You will be presented with some empirical evidence, which links a molecular fragment to a certain statistical impact on a given molecular property. You will create a hypothesis about the underlying physical and chemical mechanism that can explain why the given structure may have the observed effect.
>
> The property in question is the following: Molecular Aggregation
>
> Your answer should follow the structure below:
>
> Detailed Explanation: [Elaboration of the causal reasoning for the suggested substructure-property relationship]
>
> Hypothesis: [One sentence describing the structure and the linked property. Two sentences about the hypothesized causal explanation.]
>
> [USER PROMPT]: The structure is represented by the following scaffold in SMILES representation: {{ SMILES }}. This structure has been linked to {{ non-aggregating | aggregating }} behavior."

In contrast to previous large language models such as GPT-3.5, the GPT-4o model in most cases correctly interpreted the SMILES code and translated it to a description of the chemical structures as well as substructures contained in the explanatory motif. However, in most cases the connection to physicochemical properties did not reveal informative insights. In most cases where non-aggregating motifs were queried, the answer of the GPT-4o model referred to functional groups that enhanced hydrogen bonding with water molecules, and steric hindrance of π-stacking, which are potentially correct but also rather obvious explanations. In case of aggregating motifs, the answers frequently included functional groups that enhanced hydrogen bonding to form molecular networks, as well as planar structures that promote π-stacking. Few explanations provided further insight that might be validated and quantified in further experiments, e.g. electron-donating effects which can lead to increased electron density in the π system of the benzene ring, potentially facilitating better solvent interactions over intermolecular π-π stacking interactions.

In further tests, we used GPT-4o to analyse and summarize common structural and physicochemical characteristics in all explanation concepts, with and without revealing that the explained property is molecular aggregation. The detailed results including the prompts can be found below. One of the obtained summaries was the following: "Positive influence [on

aggregation]: High conjugation, electron-donating groups, structural rigidity, hydrophobicity, and bulky groups (especially with sulfur and nitrogen heterocycles). Negative influence [on aggregation]: Electron-withdrawing groups, polar substituents (carbonyl, amides), smaller or flexible structures, and reduced conjugation." While being highly aggregated and thus not very specific, those outputs can be used for fully automated feature engineering to train simpler subsymbolic models or even derive analytical models using methods such as symbolic regression.

Overall, we observe a drastic improvement of the usefulness of large language models in automatically interpreting results of explainable AI methods (from GPT-3.5 to GPT-4o), revealing basic insights into structure-property relations governing molecular aggregation. However, more specific insights that inspire more detailed analysis approaches or even lead to immediate understanding are still lacking.[26]

Detailed prompts:

> We trained an explainable AI methods based on graph neural networks to predict a molecular property, called PROP. We then analysed the model's explanations to identify relevant molecular motifs that increase and decrease property PROP.
>
> The following motifs increase PROP:
> [LIST OF SMILES CODES OF ALL MOTIFS]
> The following motifs increase PROP:
> [LIST OF SMILES CODES OF ALL MOTIFS]

SX. Dataset structural feature and property analysis

Numerous molecular descriptors were explored to evaluate the differences in structural features and properties of the compounds in the aggregating and non-aggregating datasets. Prior to the calculation of molecular descriptors, each compound from the cleaned datasets of aggregating and non-aggregating molecules was protonated to its most energetically favourable ionization state at physiological pH (pH=7.4) using the FixpKa functionality of OpenEye's QUACPAC 2.1.2.1.[45] Using RDKit, 111 molecular descriptors were then calculated for both the aggregator and non-aggregator compounds (full list of descriptors provided in section SXI of the Supporting Information). Of the 111 descriptors, 85 were fragment descriptors which indicate the number of occurrences of specific fragments (e.g., halogens, carboxylic acids, nitro groups) in a given molecule. The remaining 26 descriptors were for general features (e.g., molecular weight, logP, ring count, number of hydrogen bond donors) of the molecules.

Histograms of each feature were plotted for both the aggregator and non-aggregator molecules to assess the distribution of the data (all histograms presented in sections SXII-SXIV of the Supporting Information). Nearly all 85 fragment descriptors, as well as 10 of the 26 general descriptors, showed a non-normal distribution. As such, the fragment descriptors were analyzed separately from the general descriptors, and only the 16 general descriptors following a normal distribution were considered for further analysis.

To assess to statistical significance of each of the 16 general descriptors for the aggregating and non-aggregating molecules, p-values were calculated for each descriptor from a standard independent two sample t-test assuming equal population variances (calculated using scipy.stats.ttest_ind).[46] With the exception of the NumRotatableBonds descriptor, all p-values were determined to be less than 0.005, indicating a high statistical significance for these descriptors. As such, the effect size was computed for the 15 statistically significant descriptors as the magnitude of the Cohen's d value.

$$Cohen's\ d = \frac{\mu_1 - \mu_2}{\sqrt{\frac{1}{2}((\sigma_1)^2 + (\sigma_2)^2)}} \qquad \text{(Eq. S3)}$$

Where μ_1 and μ_2 are the means of the descriptor values for aggregators and non-aggregators, and σ_1 and σ_2 are the standard deviations. The p-values, means, standard deviations and Cohen's d values for the 15 statistically significant general molecular descriptors are presented in section SXV of the Supporting Information.

Out of the 16 normally distributed general molecular descriptors, 15 were calculated to have p-values of less than 0.005, indicating that they are highly statistically significant. For these 15 descriptors, the effect size was calculated in order to look for meaningful relationships between the properties of the molecules in the aggregating and non-aggregating datasets. As a measure of the difference in the mean of each descriptor of the two data sets, Cohen's d values were calculated (Figure S5). The generally accepted interpretation of Cohen's d values is that a value of less than 0.2 is a small effect size, values near 0.5 are a medium effect size, and values greater than 0.8 are a large effect size.[47] Cohen's d values indicate that the logP has a medium to large effect size. Further analysis of the histogram for this feature (Figure S6a) indicates that the molecules in the aggregator data set have a higher logP, on average, than those in the non-

aggregator data set. The number of aromatic rings (both NumAromaticCarbocycles and NumAromaticRings) and the fraction of sp3 hybridized carbon atoms (FractionCSP3) are found to have medium effect sizes, with the aggregators having higher values on average for the number of aromatic rings and lower values on average for the fraction of sp3 hybridized carbons. The observation that the aggregators have a smaller fraction of sp3 hybridized carbons is consistent with the greater aromaticity observed in the aggregators dataset. Consistent with many other reports on aggregation features, our data indicates that both logP and aromaticity are linked to aggregation. Alternatively, other features proposed in the literature to be important to small molecule aggregation (i.e. number of sulfur atoms and number of hydroxyl groups) were not found to vary significantly between the molecules in the aggregating and non-aggregating datasets (Figure S6d).[11]

Analysis of the fragment descriptors through comparison of the relative frequency of each of the fragments between the aggregator and non-aggregator molecules revealed that the fragments provide little insight into the difference in the chemical nature of the aggregator and non-aggregator molecules. Plots of the relative frequency of each of the fragment descriptors are presented in section SXVI of the Supporting Information.

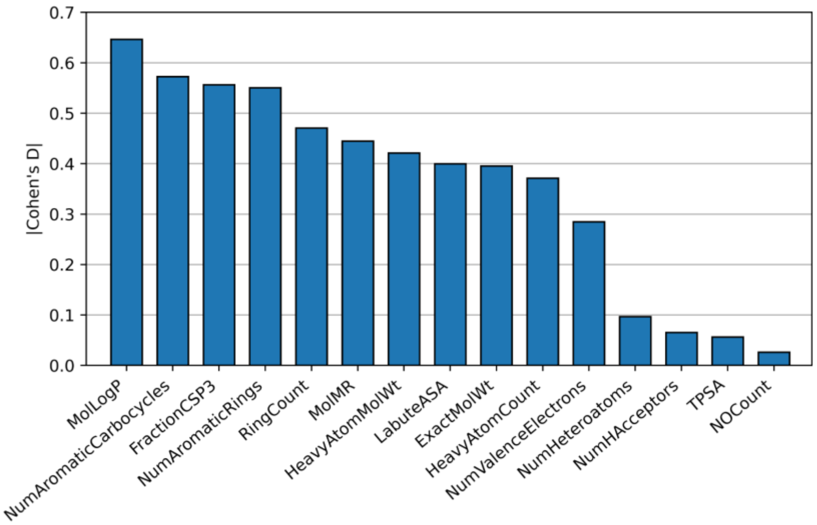

Figure S5. The absolute value of the Cohen's d for the 15 statistically significant general molecular descriptors.

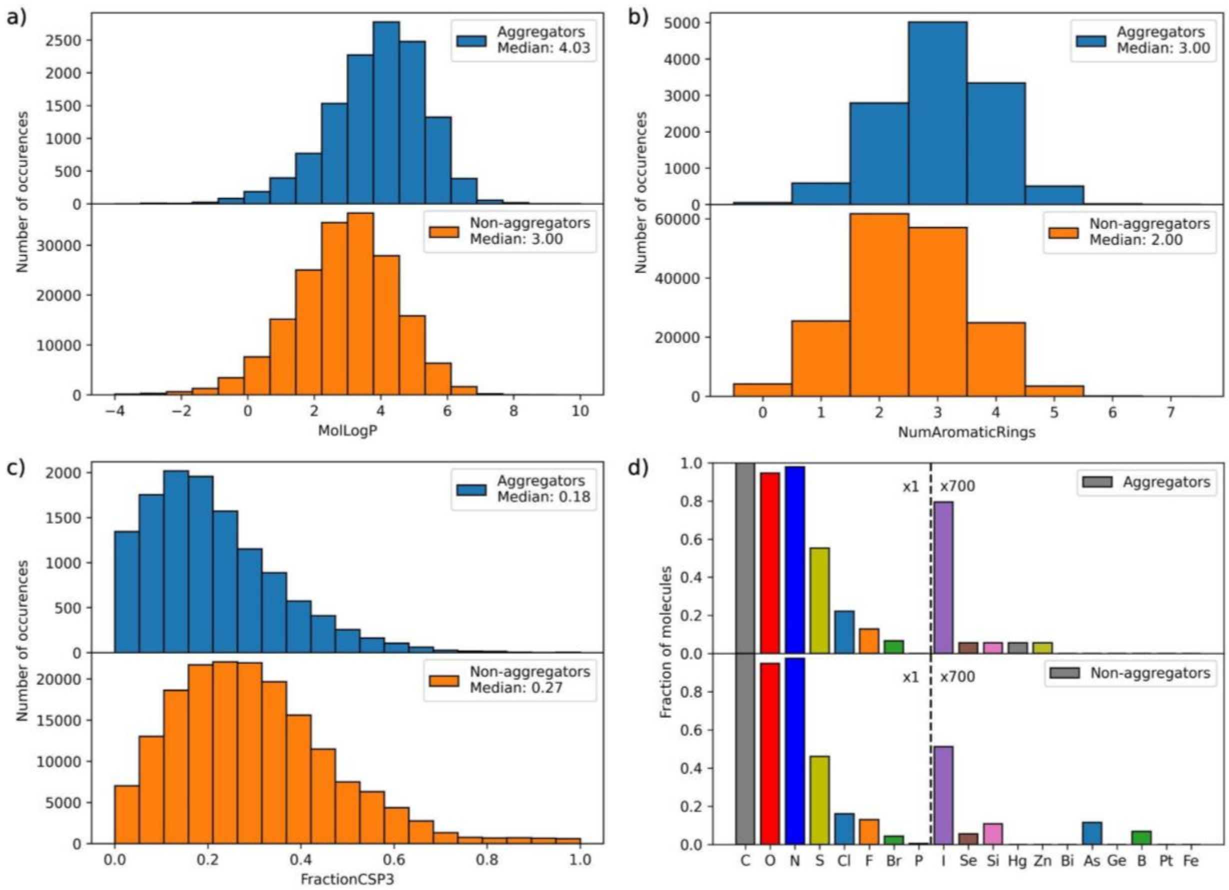

Figure S6. Histograms of a) MolLogP, b) Number of aromatic rings per molecule, c) Fraction of SP3 hybridized carbon, and d) element frequencies for the aggregator and non-aggregator datasets.

SXI. List of all RDKit molecular descriptors calculated for all compounds in the aggregator and non-aggregator datasets

ExactMolWt, FractionCSP3, HeavyAtomCount, HeavyAtomMolWt, LabuteASA, MolLogP, MolMR, NHOHCount, NOCount, NumAliphaticCarbocycles, NumAliphaticHeterocycles, NumAliphaticRings, NumAromaticCarbocycles, NumAromaticHeterocycles, NumAromaticRings, NumHAcceptors, NumHDonors, NumHeteroatoms, NumRadicalElectrons, NumRotatableBonds, NumSaturatedCarbocycles, NumSaturatedHeterocycles, NumSaturatedRings, NumValenceElectrons, RingCount, TPSA, fr_Al_COO, fr_Al_OH, fr_Al_OH_noTert, fr_ArN, fr_Ar_COO, fr_Ar_N, fr_Ar_NH, fr_Ar_OH, fr_COO, fr_COO2, fr_C_O, fr_C_O_noCOO, fr_C_S, fr_HOCCN, fr_Imine, fr_NH0, fr_NH1, fr_NH2, fr_N_O, fr_Ndealkylation1, fr_Ndealkylation2, fr_Nhpyrrole, fr_SH, fr_aldehyde, fr_alkyl_carbamate, fr_alkyl_halide, fr_allylic_oxid, fr_amide, fr_amidine, fr_aniline, fr_aryl_methyl, fr_azide, fr_azo, fr_barbitur, fr_benzene, fr_benzodiazepine, fr_bicyclic, fr_diazo, fr_dihydropyridine, fr_epoxide, fr_ester, fr_ether, fr_furan, fr_guanido, fr_halogen, fr_hdrzine, fr_hdrzone, fr_imidazole, fr_imide, fr_isocyan, fr_isothiocyan, fr_ketone, fr_ketone_Topliss, fr_lactam, fr_lactone, fr_methoxy, fr_morpholine, fr_nitrile, fr_nitro, fr_nitro_arom, fr_nitro_arom_nonortho, fr_nitroso, fr_oxazole, fr_oxime, fr_para_hydroxylation, fr_phenol, fr_phenol_noOrthoHbond, fr_phos_acid, fr_phos_ester, fr_piperdine, fr_piperzine, fr_priamide, fr_prisulfonamd, fr_pyridine, fr_quatN, fr_sulfide, fr_sulfonamd, fr_sulfone, fr_term_acetylene, fr_tetrazole, fr_thiazole, fr_thiocyan, fr_thiophene, fr_unbrch_alkane, fr_urea

SXII. Histograms of normally distributed general molecular descriptors for the aggregator and non-aggregator datasets

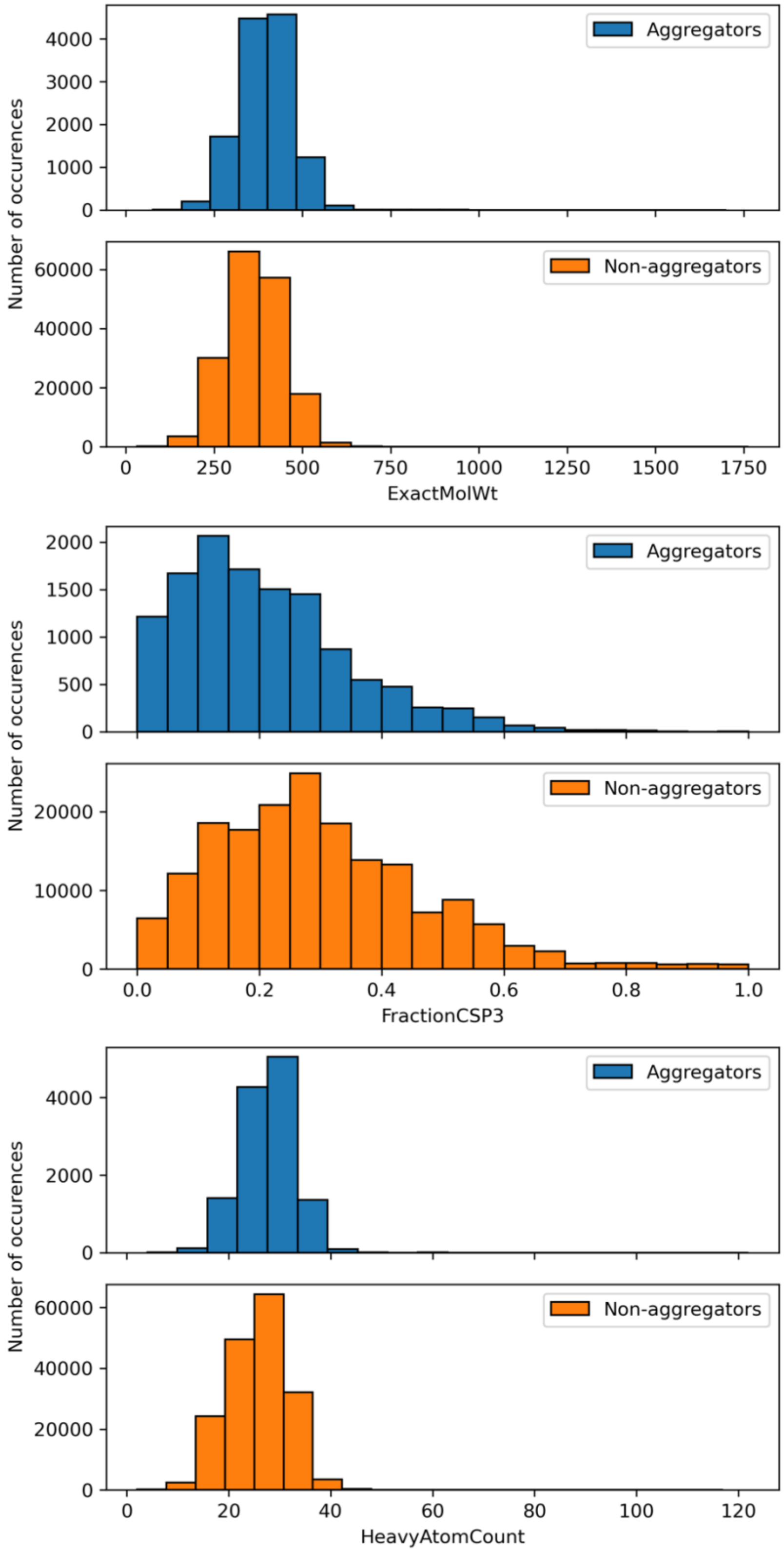

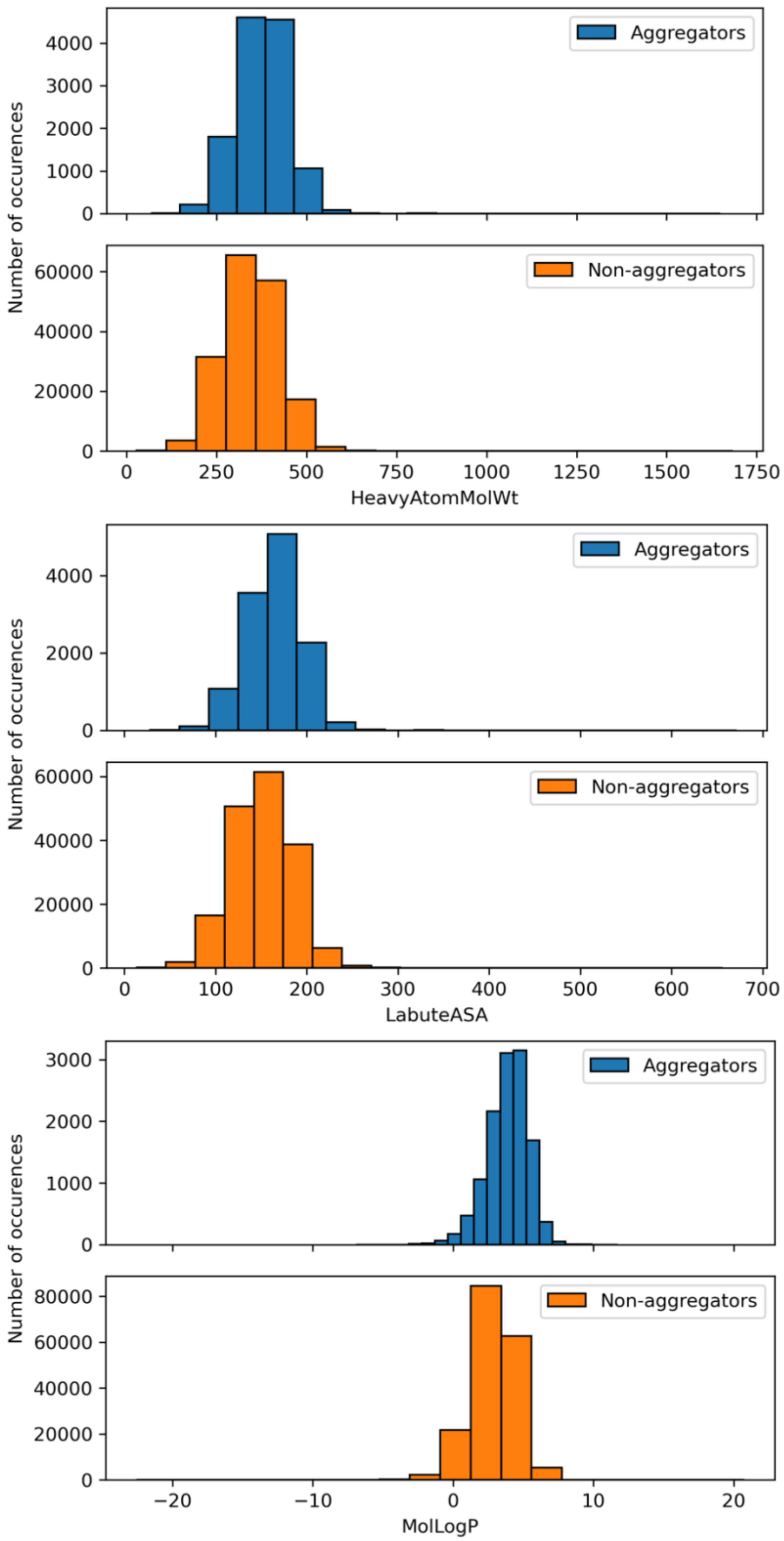
Aggregators
Non-aggregators
Number of occurences
HeavyAtomMolWt
0
250
500
750
1000
1250
1500
1750
0
1000
2000
3000
4000
0
20000
40000
60000
Aggregators
Non-aggregators
Number of occurences
LabuteASA
0
100
200
300
400
500
600
700
0
2000
4000
0
20000
40000
60000
Aggregators
Non-aggregators
Number of occurences
MolLogP
−20
−10
0
10
20
0
1000
2000
3000
0
20000
40000
60000
80000

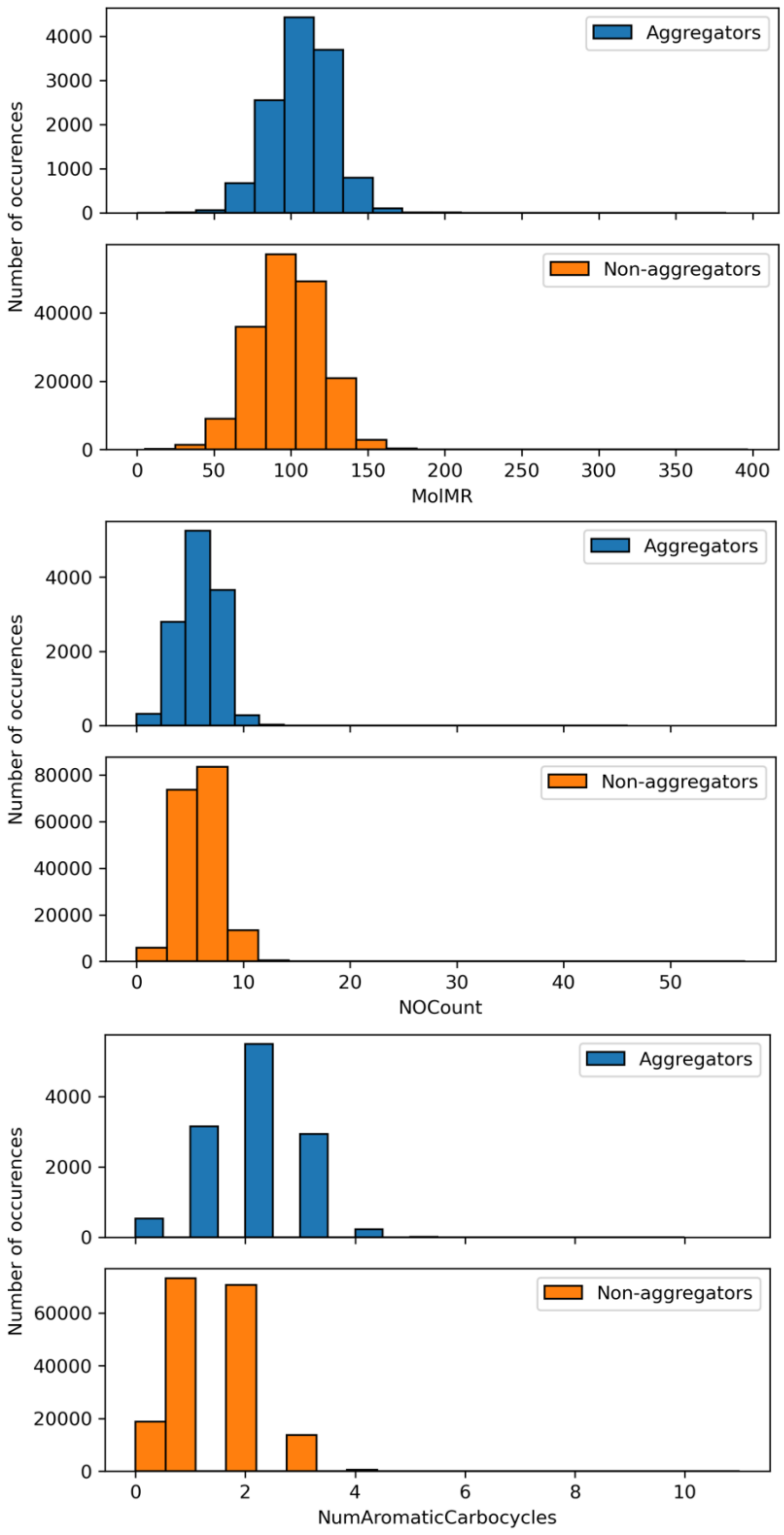
Aggregators
Non-aggregators
Number of occurences
MolMR
NOCount
NumAromaticCarbocycles

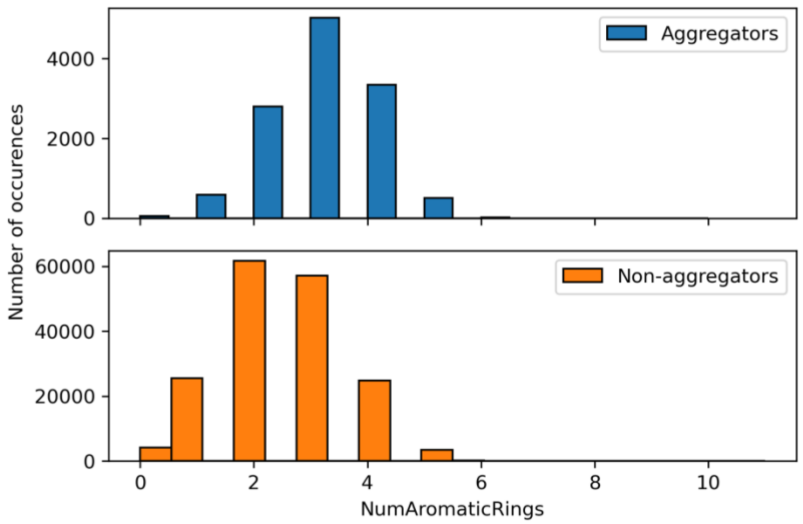
Aggregators
Non-aggregators
Number of occurences
NumAromaticRings

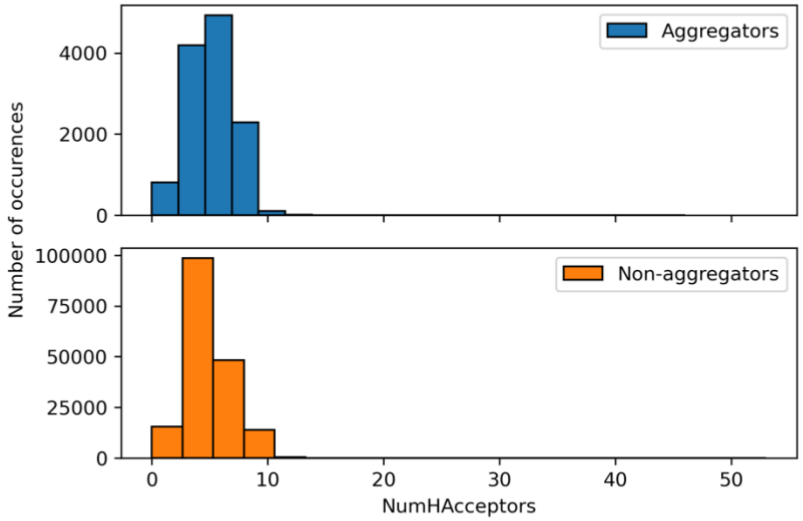
Aggregators
Non-aggregators
Number of occurences
NumHAcceptors

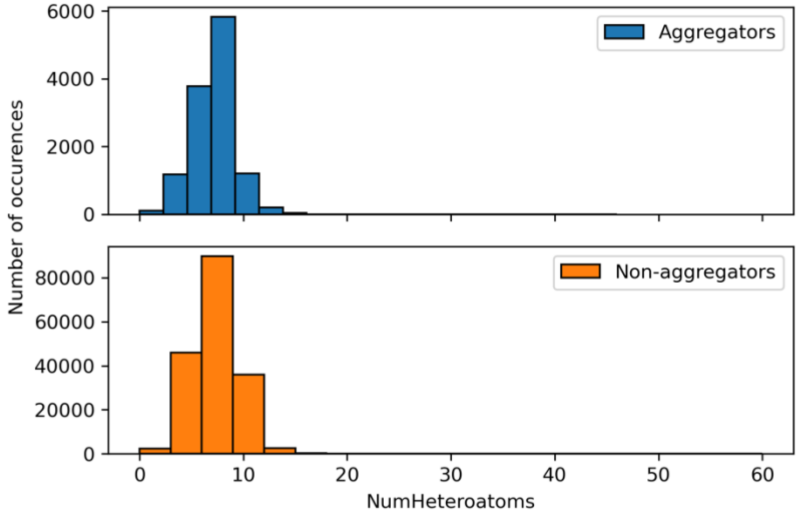
Aggregators
Non-aggregators
Number of occurences
NumHeteroatoms

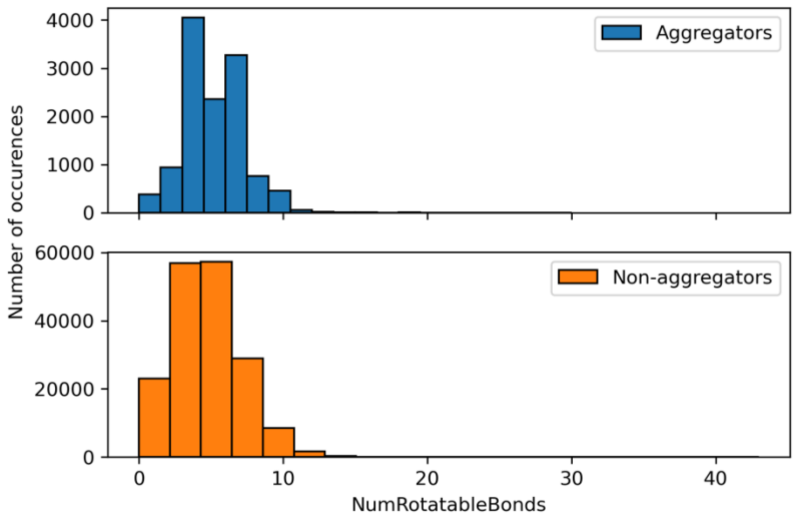
Aggregators
Non-aggregators
Number of occurences
NumRotatableBonds
4000
3000
2000
1000
0
60000
40000
20000
0
0
10
20
30
40

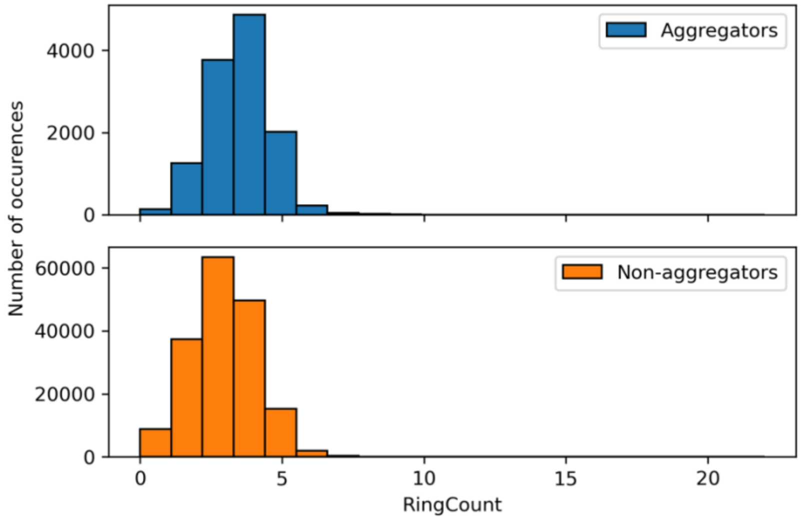
Aggregators
Non-aggregators
Number of occurences
RingCount
4000
2000
0
60000
40000
20000
0
0
5
10
15
20

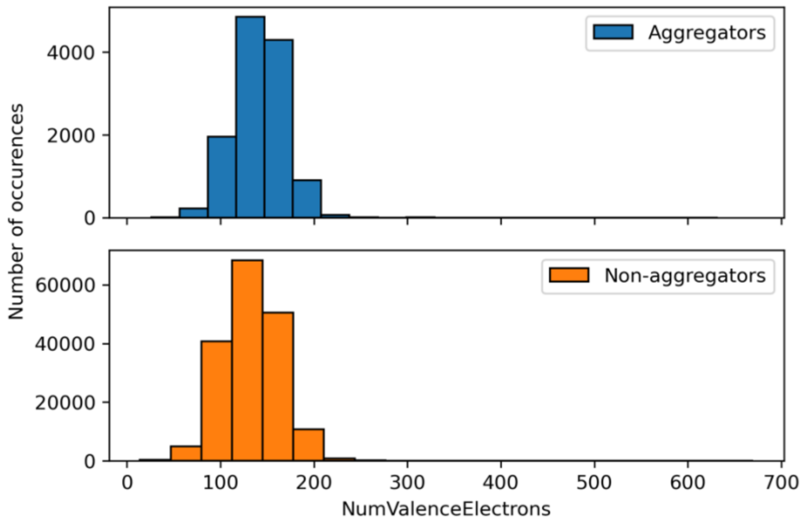
Aggregators
Non-aggregators
Number of occurences
NumValenceElectrons
4000
2000
0
60000
40000
20000
0
0
100
200
300
400
500
600
700

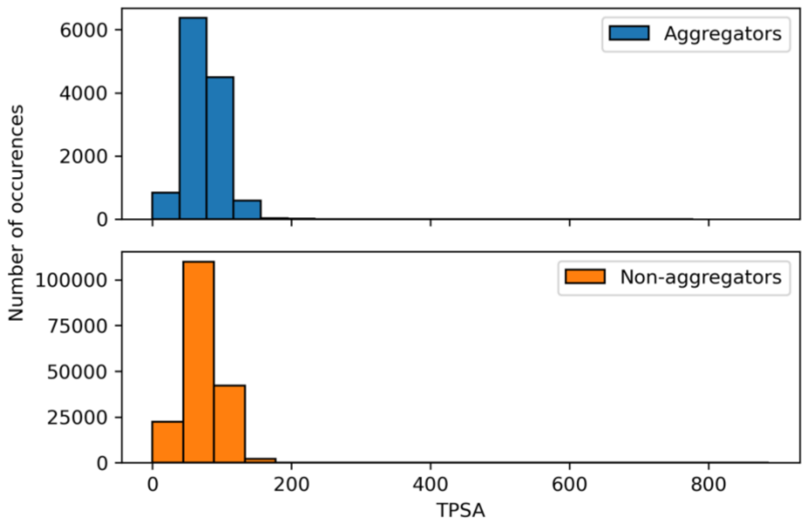

Aggregators
Non-aggregators
Number of occurences
6000
4000
2000
0
100000
75000
50000
25000
0
0
200
400
600
800
TPSA

SXIII. Histograms of non-normally distributed general molecular descriptors for the aggregator and non-aggregator datasets

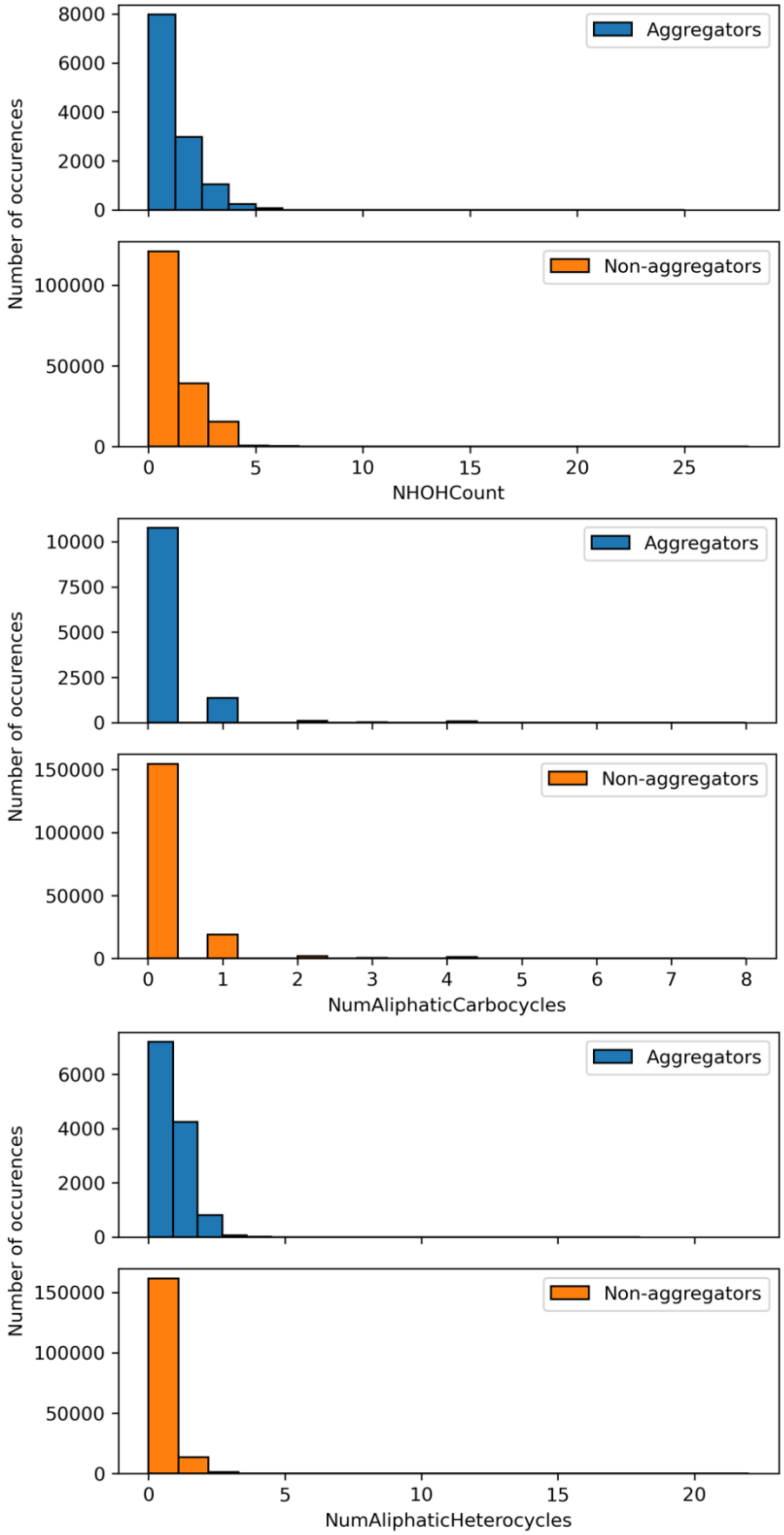

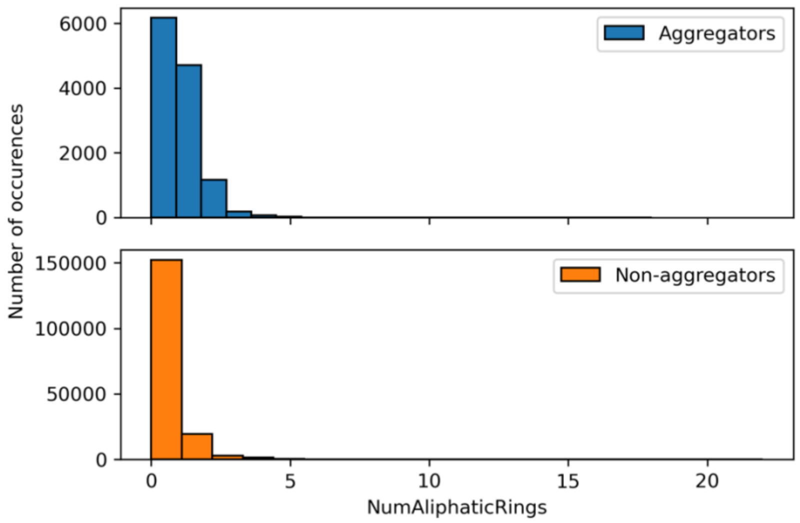

Aggregators
Non-aggregators
Number of occurences
NumAliphaticRings

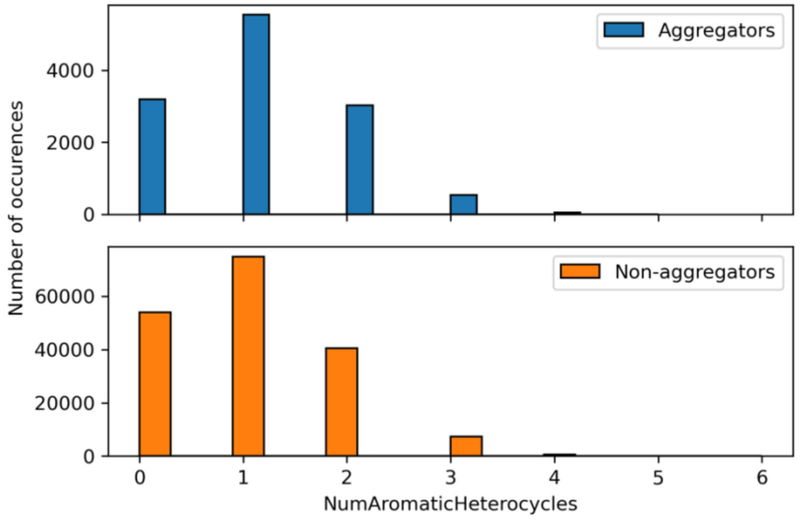

Aggregators
Non-aggregators
Number of occurences
NumAromaticHeterocycles

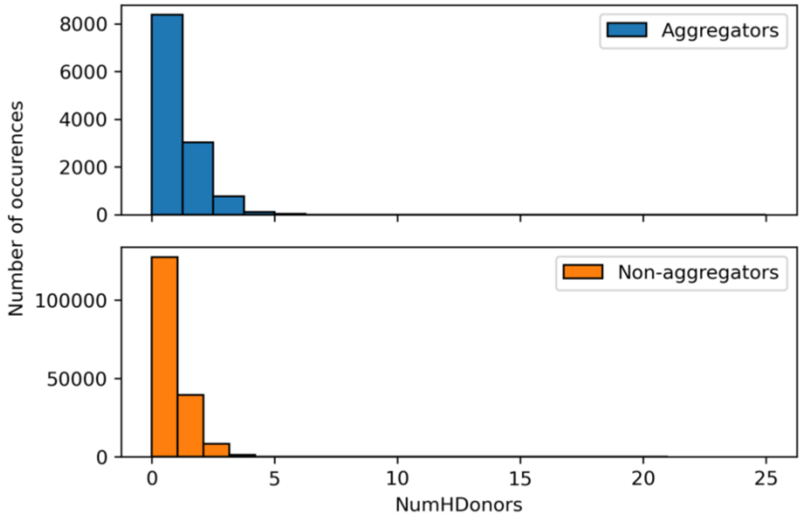

Aggregators
Non-aggregators
Number of occurences
NumHDonors

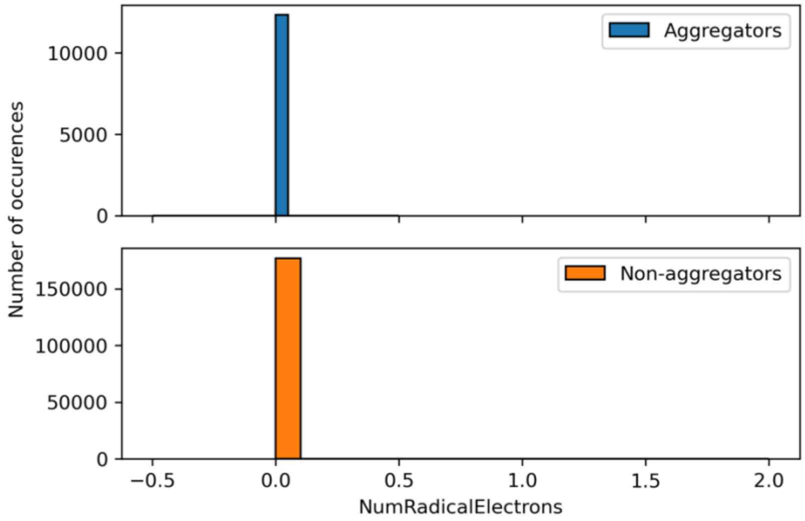
Aggregators
Non-aggregators
Number of occurences
NumRadicalElectrons

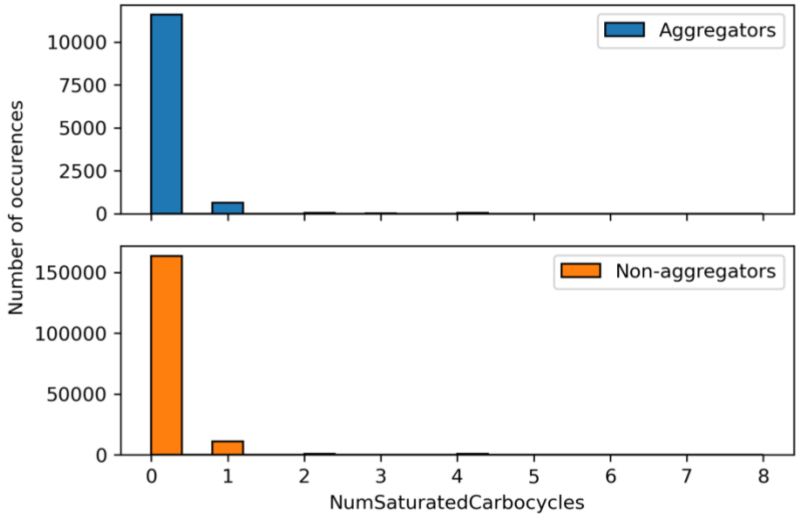
Aggregators
Non-aggregators
Number of occurences
NumSaturatedCarbocycles

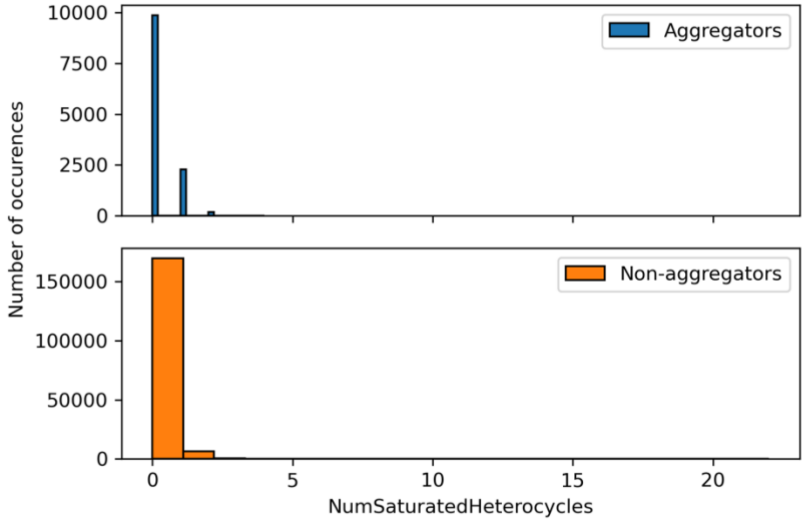
Aggregators
Non-aggregators
Number of occurences
NumSaturatedHeterocycles

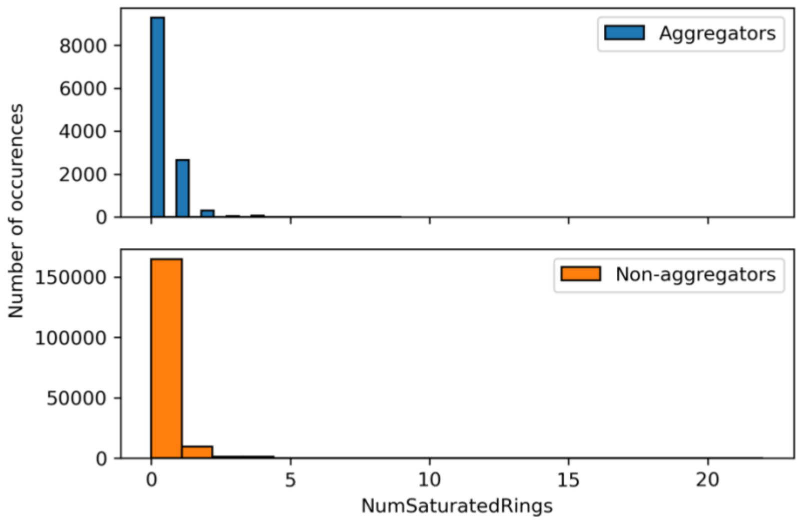

Aggregators
Non-aggregators
Number of occurences
8000
6000
4000
2000
0
150000
100000
50000
0
0
5
10
15
20
NumSaturatedRings

SXIV. Histograms of fragment molecular descriptors for the aggregator and non-aggregator datasets

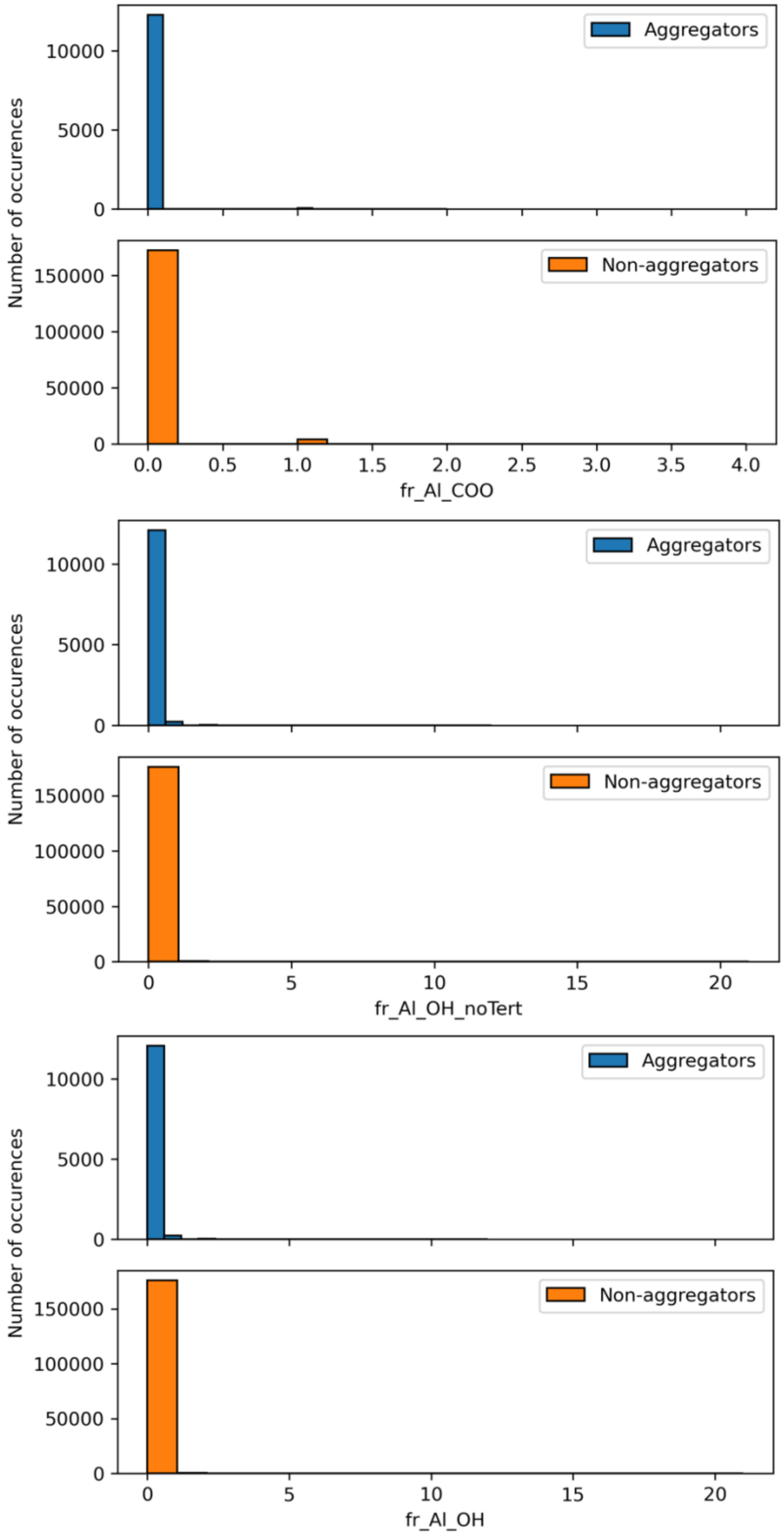

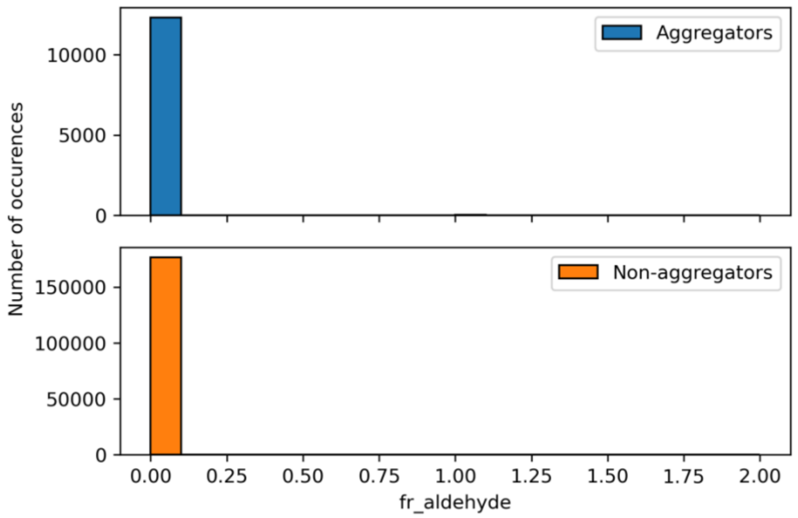
Aggregators
Non-aggregators
Number of occurences
fr_aldehyde

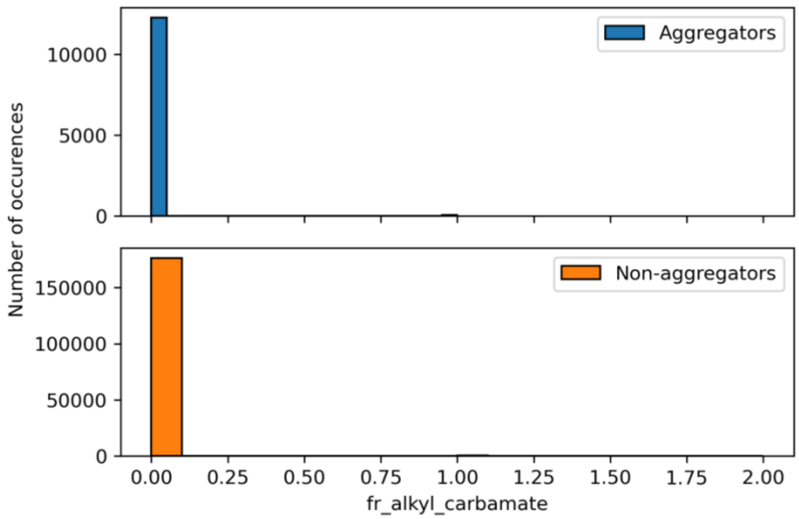
Aggregators
Non-aggregators
Number of occurences
fr_alkyl_carbamate

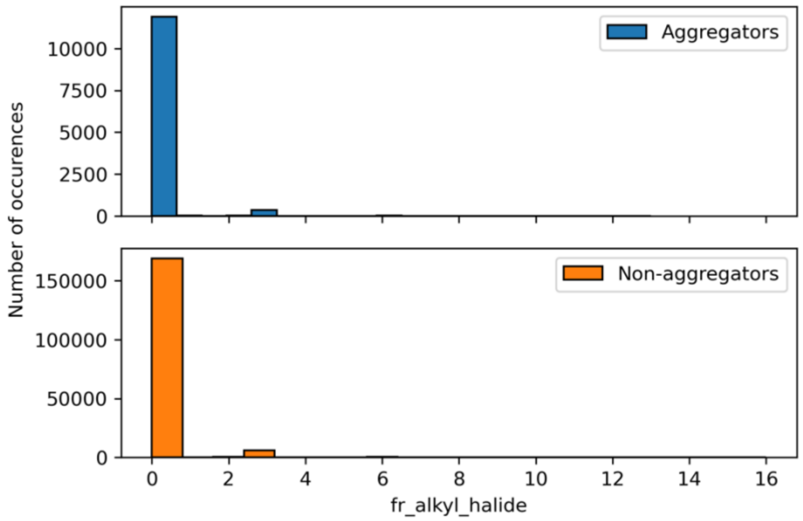
Aggregators
Non-aggregators
Number of occurences
fr_alkyl_halide

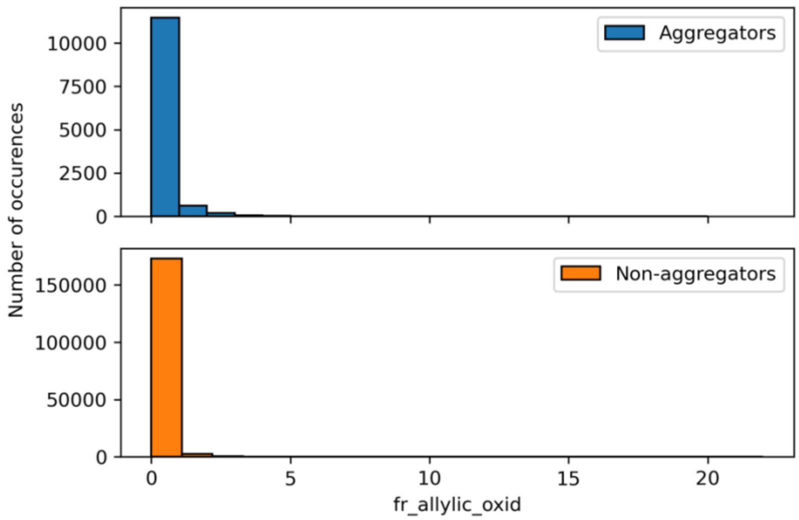
Aggregators
Non-aggregators
Number of occurences
fr_allylic_oxid

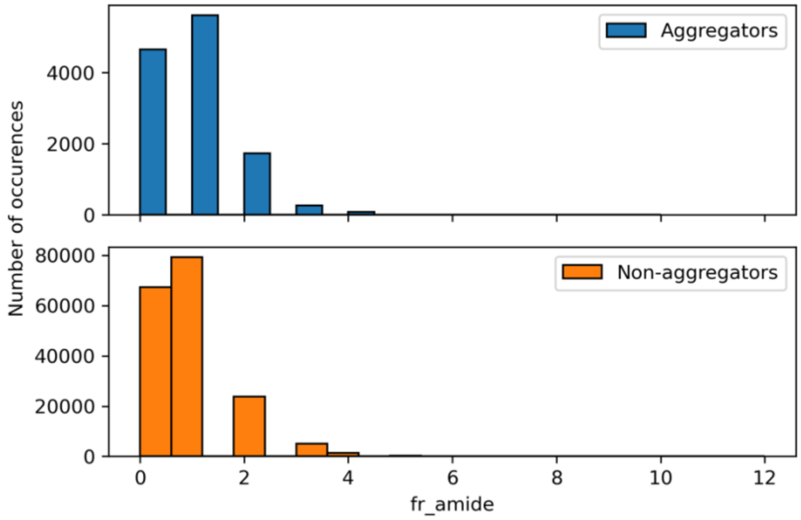
Aggregators
Non-aggregators
Number of occurences
fr_amide

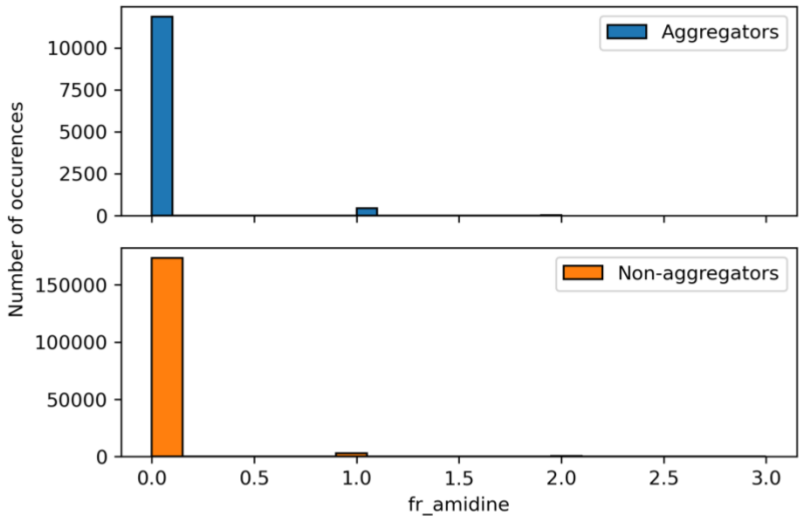
Aggregators
Non-aggregators
Number of occurences
fr_amidine

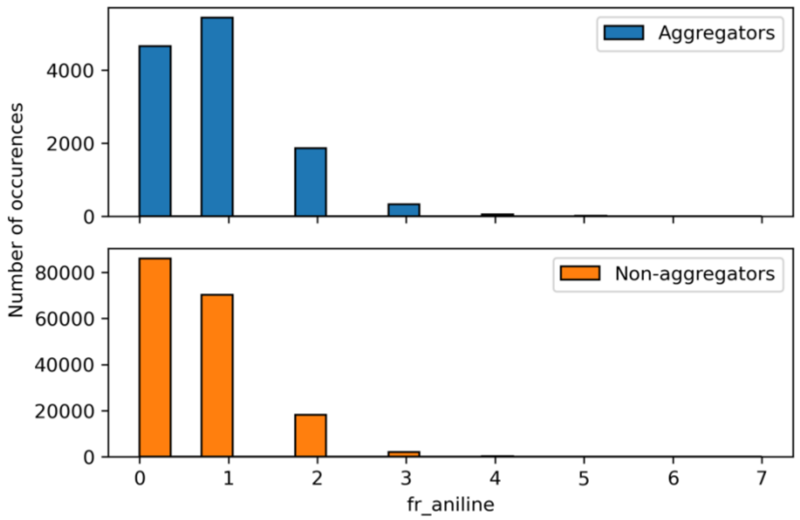
Aggregators
Non-aggregators
Number of occurences
fr_aniline

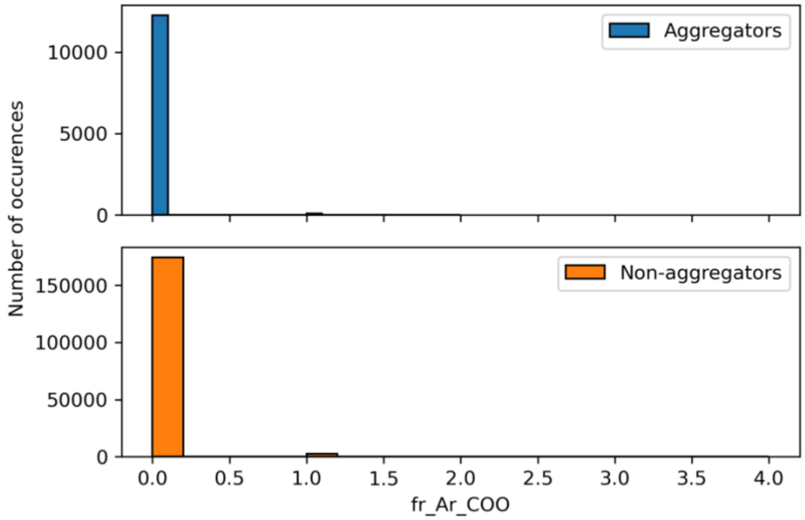
Aggregators
Non-aggregators
Number of occurences
fr_Ar_COO

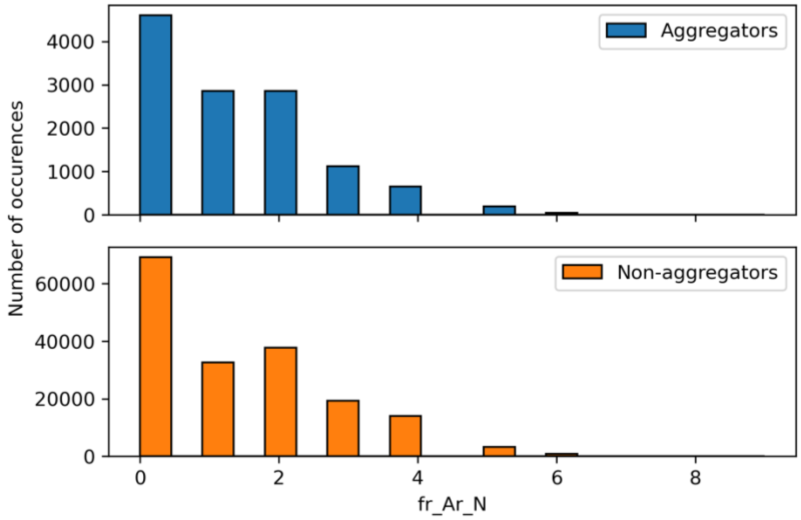
Aggregators
Non-aggregators
Number of occurences
fr_Ar_N

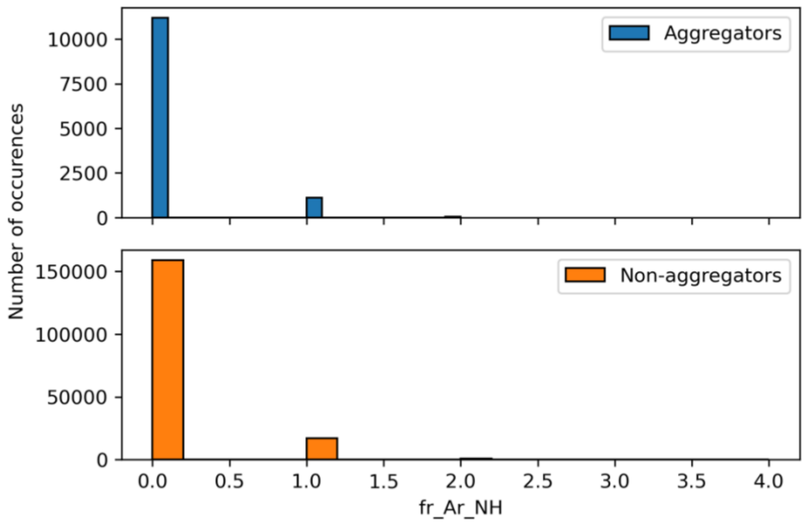

Aggregators
Non-aggregators
Number of occurences
10000
7500
5000
2500
0
150000
100000
50000
0.0 0.5 1.0 1.5 2.0 2.5 3.0 3.5 4.0
fr_Ar_NH

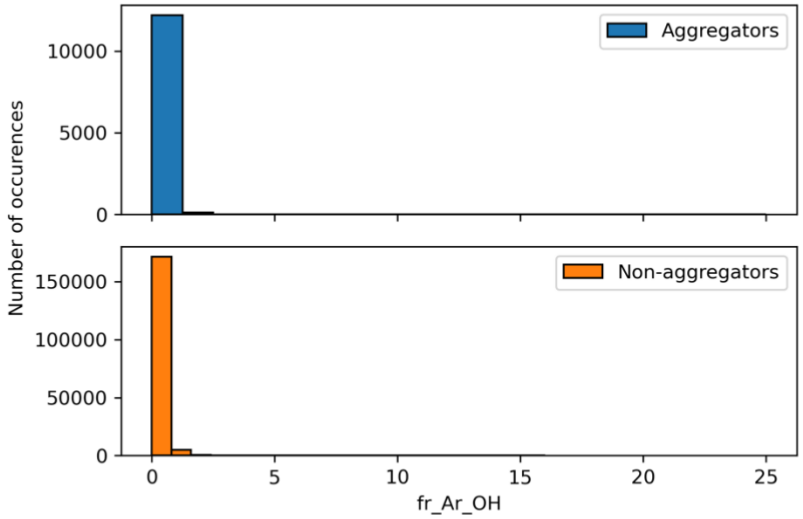

Aggregators
Non-aggregators
Number of occurences
10000
5000
0
150000
100000
50000
0 5 10 15 20 25
fr_Ar_OH

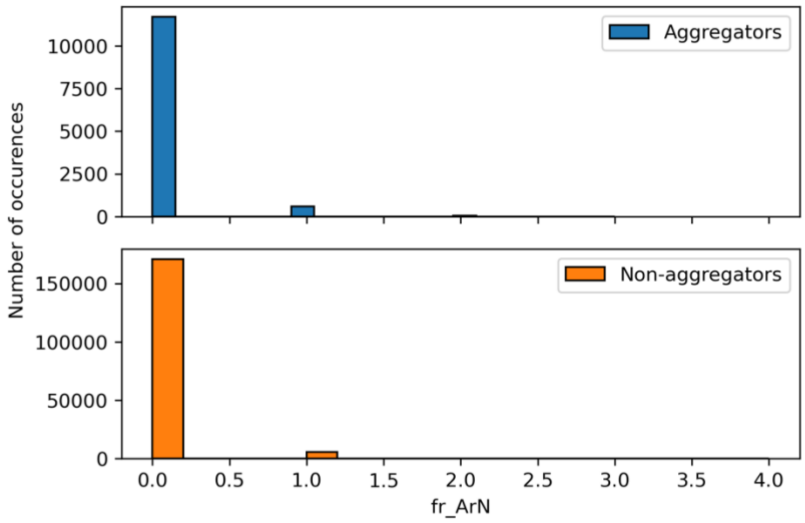

Aggregators
Non-aggregators
Number of occurences
10000
7500
5000
2500
0
150000
100000
50000
0.0 0.5 1.0 1.5 2.0 2.5 3.0 3.5 4.0
fr_ArN

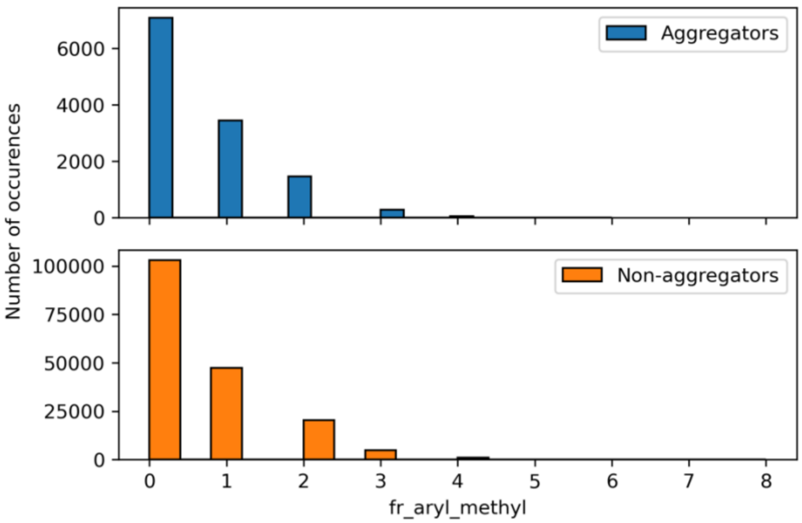
Aggregators
Non-aggregators
Number of occurences
fr_aryl_methyl

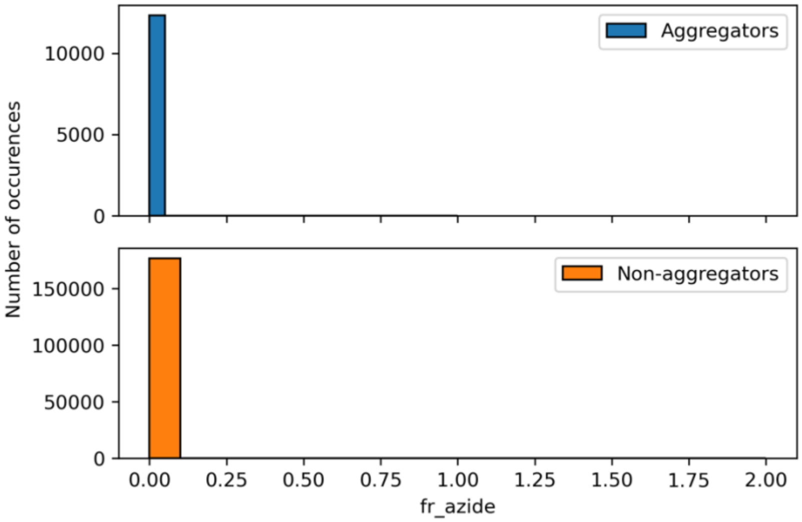
Aggregators
Non-aggregators
Number of occurences
fr_azide

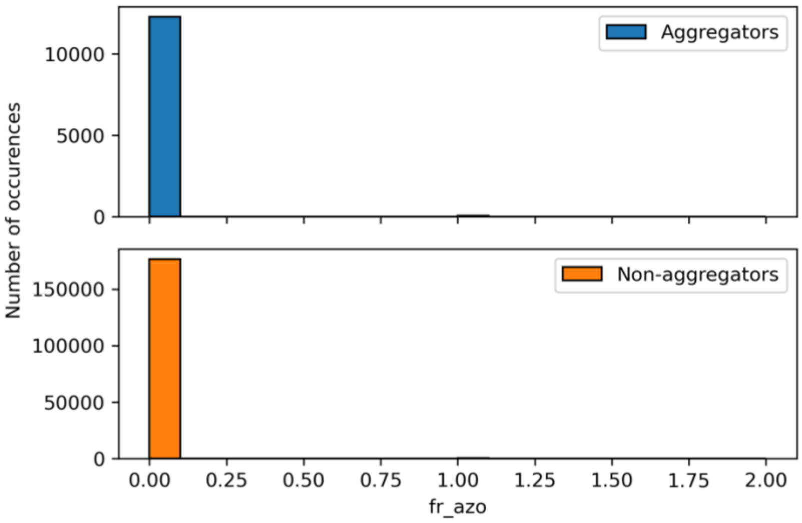
Aggregators
Non-aggregators
Number of occurences
fr_azo

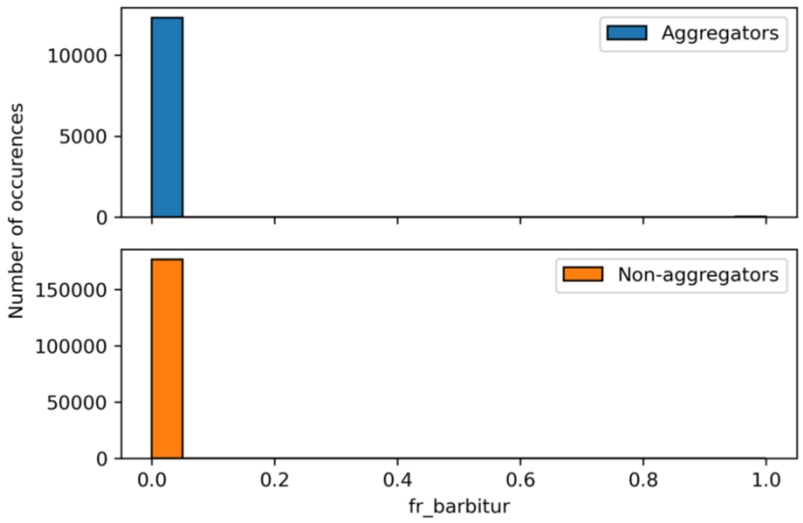

Aggregators
Non-aggregators
Number of occurences
10000
5000
0
150000
100000
50000
0
0.0
0.2
0.4
0.6
0.8
1.0
fr_barbitur

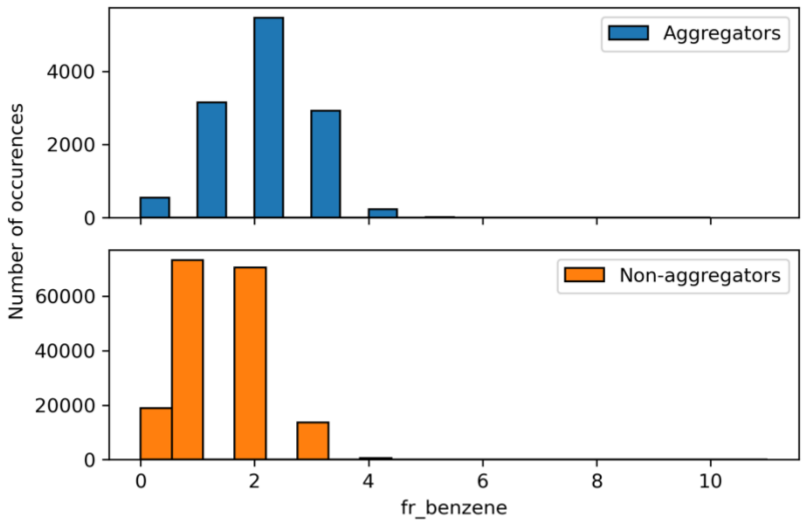

Aggregators
Non-aggregators
Number of occurences
4000
2000
0
60000
40000
20000
0
0
2
4
6
8
10
fr_benzene

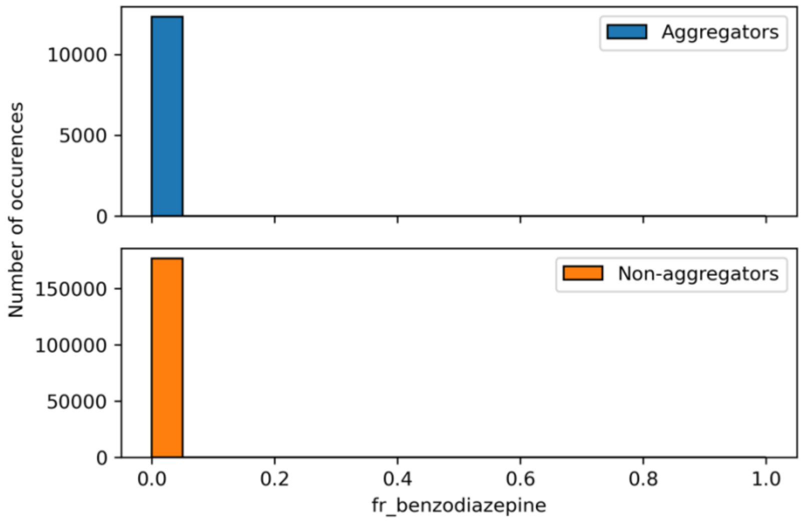

Aggregators
Non-aggregators
Number of occurences
10000
5000
0
150000
100000
50000
0
0.0
0.2
0.4
0.6
0.8
1.0
fr_benzodiazepine

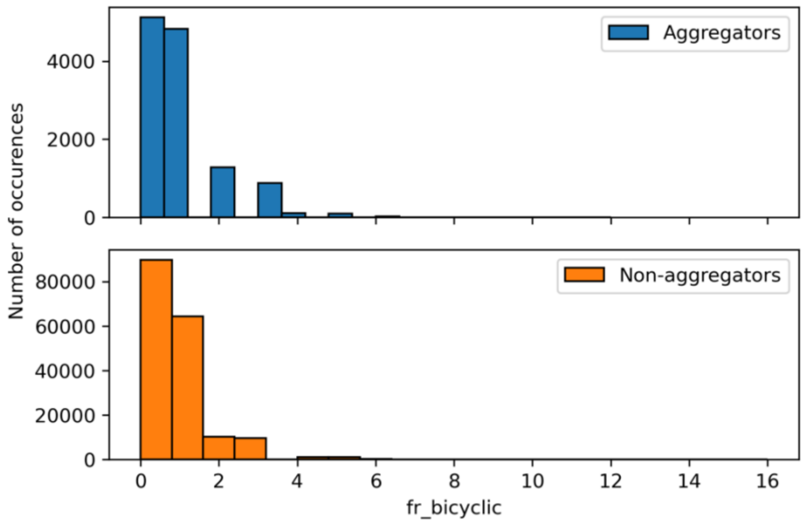
Aggregators
Non-aggregators
Number of occurences
fr_bicyclic

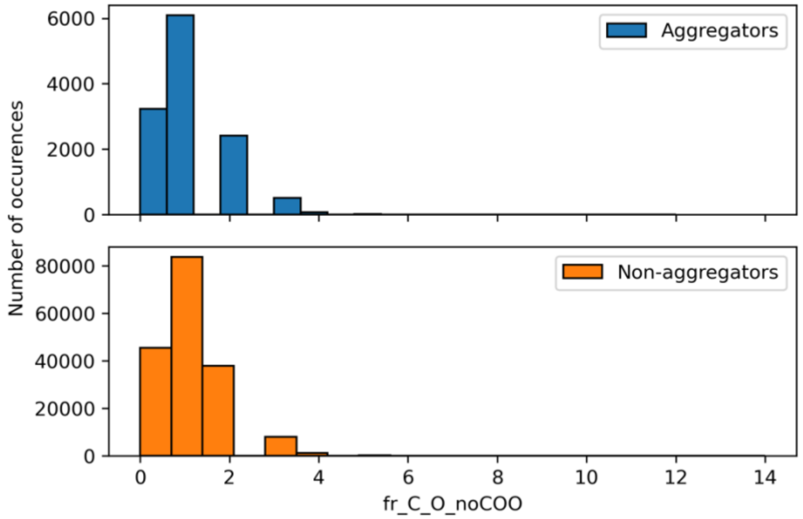
Aggregators
Non-aggregators
Number of occurences
fr_C_O_noCOO

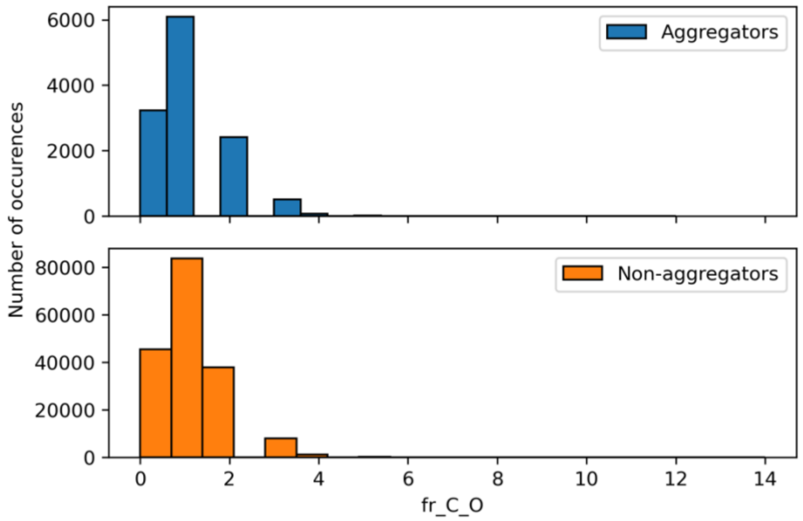
Aggregators
Non-aggregators
Number of occurences
fr_C_O

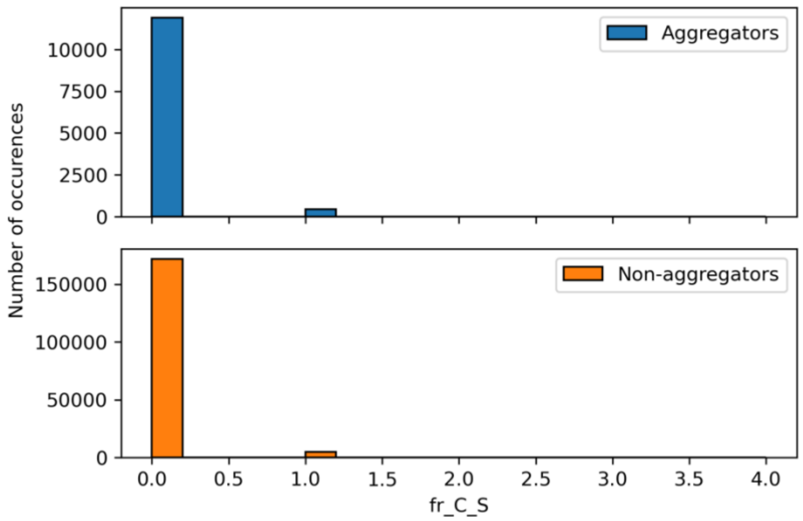
Aggregators
Non-aggregators
Number of occurences
10000
7500
5000
2500
0
150000
100000
50000
0
0.0
0.5
1.0
1.5
2.0
2.5
3.0
3.5
4.0
fr_C_S

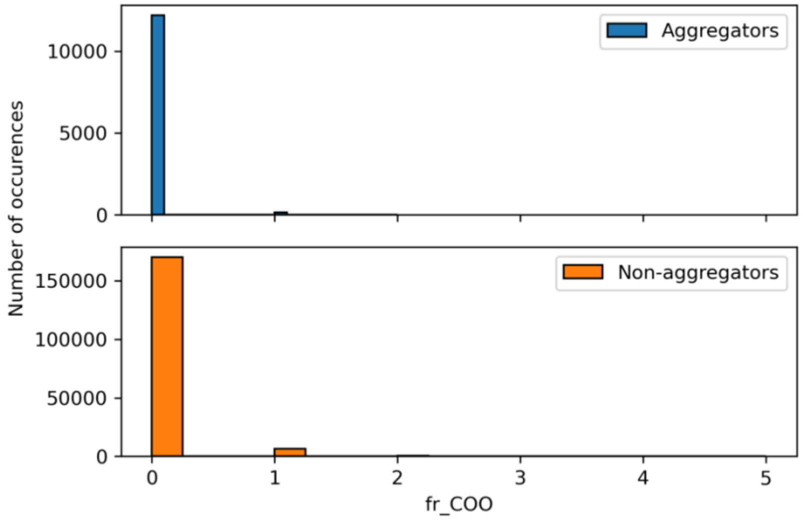
Aggregators
Non-aggregators
Number of occurences
10000
5000
0
150000
100000
50000
0
0
1
2
3
4
5
fr_COO

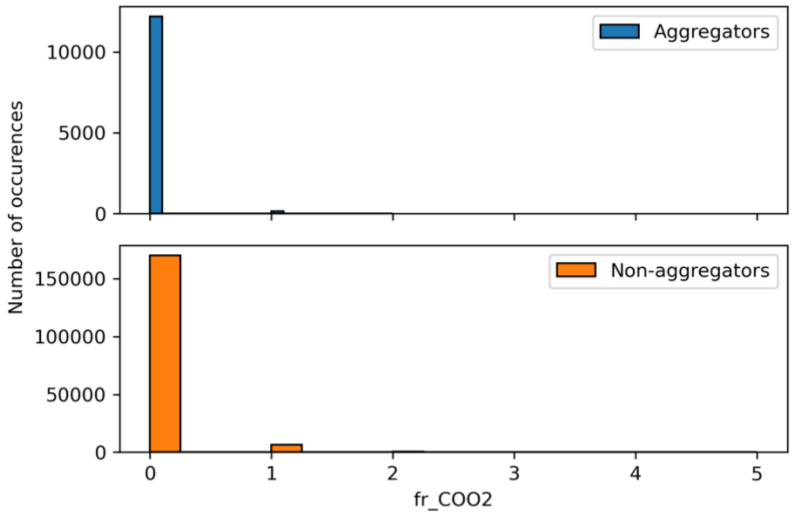
Aggregators
Non-aggregators
Number of occurences
10000
5000
0
150000
100000
50000
0
0
1
2
3
4
5
fr_COO2

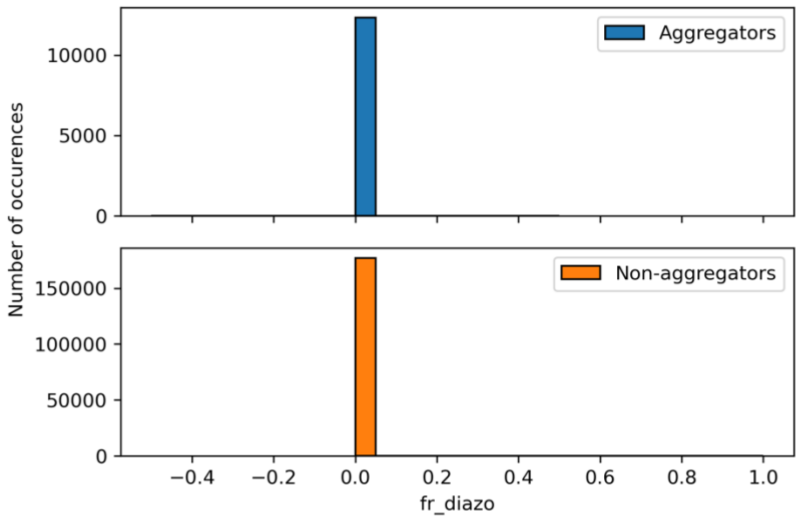
Aggregators
Non-aggregators
Number of occurences
fr_diazo

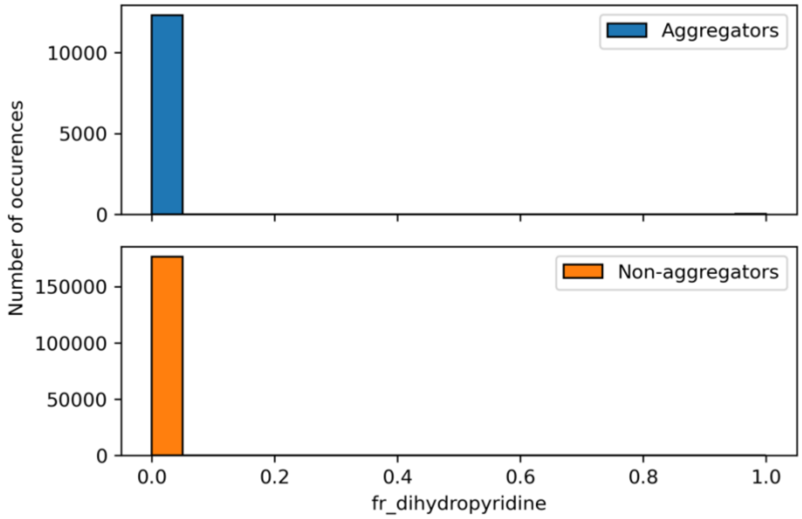
Aggregators
Non-aggregators
Number of occurences
fr_dihydropyridine

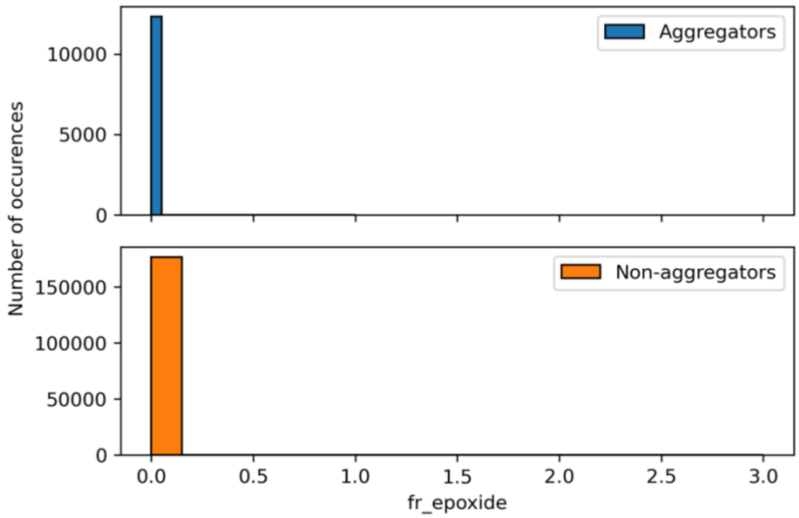
Aggregators
Non-aggregators
Number of occurences
fr_epoxide

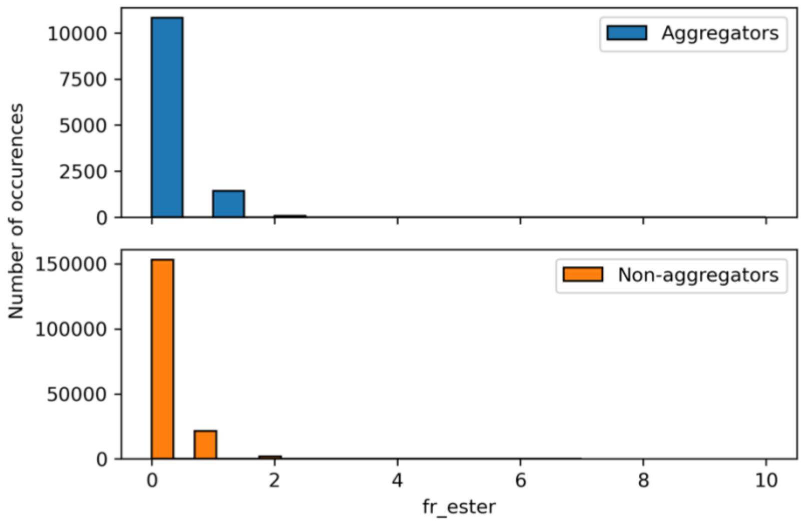

Aggregators
Non-aggregators
Number of occurences
fr_ester

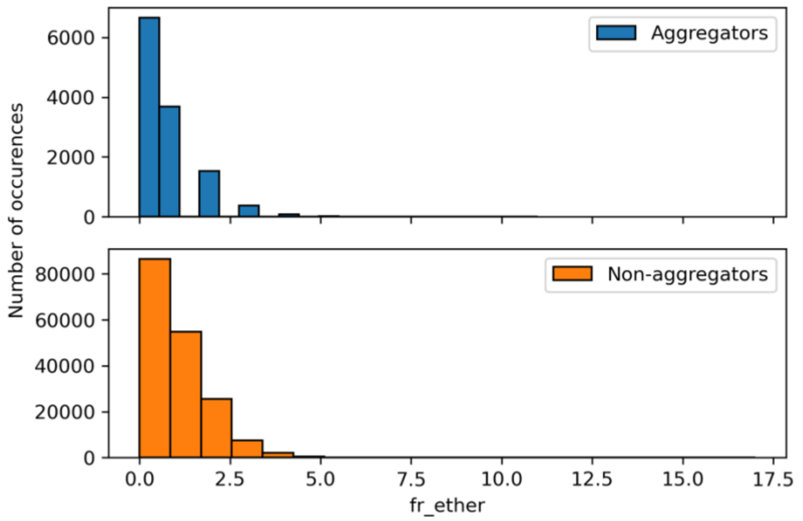

Aggregators
Non-aggregators
Number of occurences
fr_ether

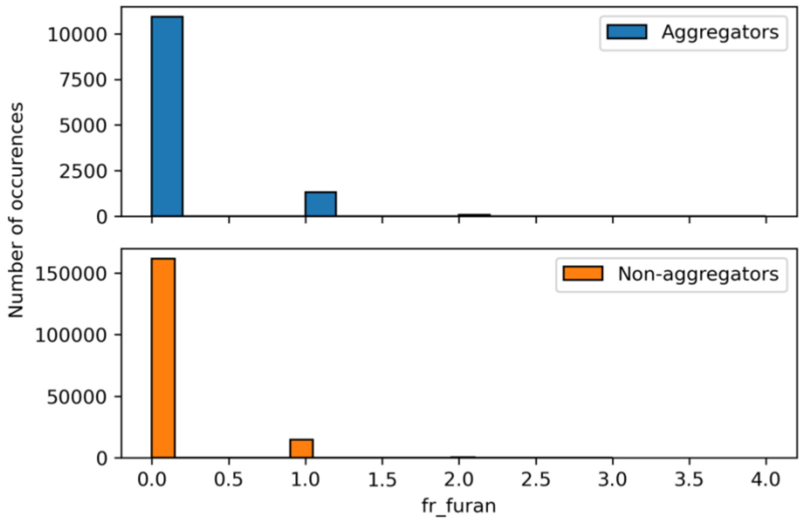

Aggregators
Non-aggregators
Number of occurences
fr_furan

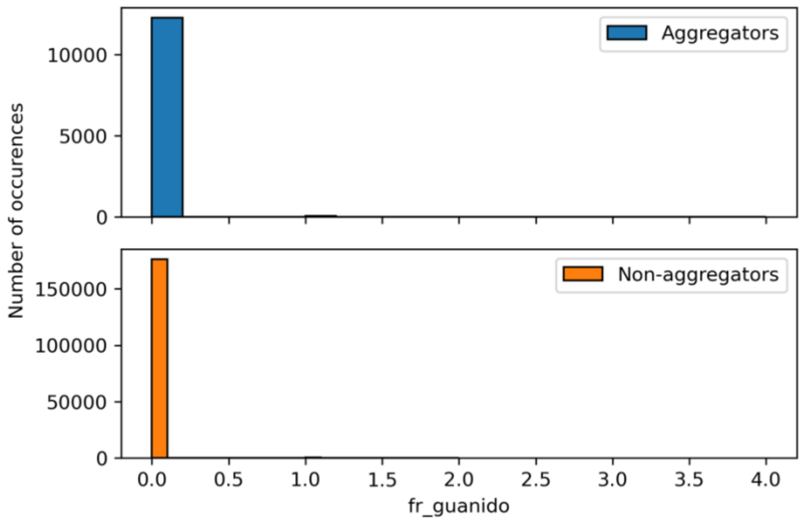
Aggregators
Non-aggregators
Number of occurences
10000
5000
0
150000
100000
50000
0
0.0
0.5
1.0
1.5
2.0
2.5
3.0
3.5
4.0
fr_guanido

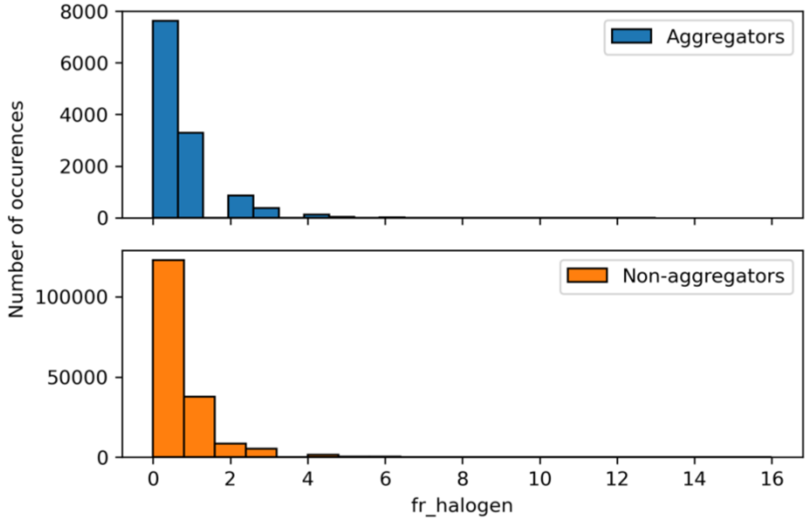
Aggregators
Non-aggregators
Number of occurences
8000
6000
4000
2000
0
100000
50000
0
0
2
4
6
8
10
12
14
16
fr_halogen

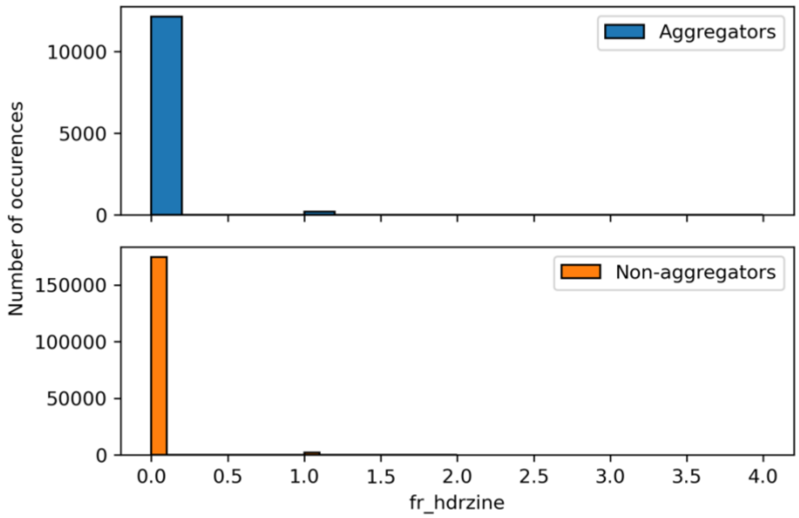
Aggregators
Non-aggregators
Number of occurences
10000
5000
0
150000
100000
50000
0
0.0
0.5
1.0
1.5
2.0
2.5
3.0
3.5
4.0
fr_hdrzine

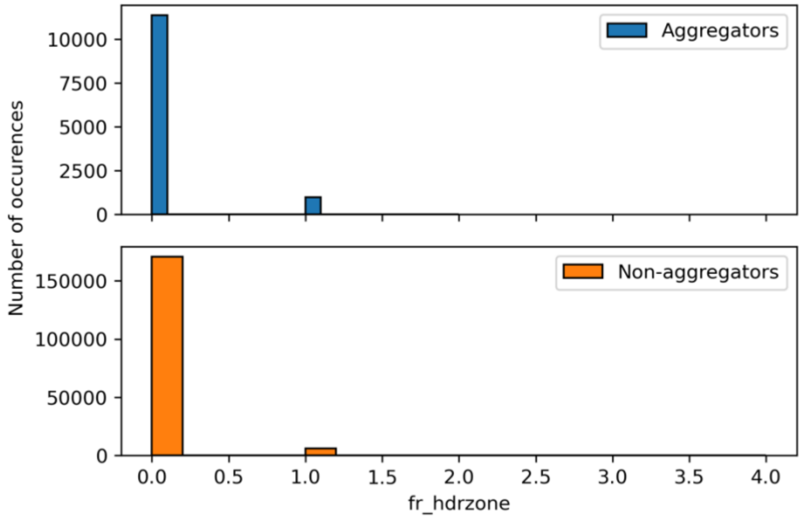
Aggregators
Non-aggregators
Number of occurences
10000
7500
5000
2500
0
150000
100000
50000
0
0.0 0.5 1.0 1.5 2.0 2.5 3.0 3.5 4.0
fr_hdrzone

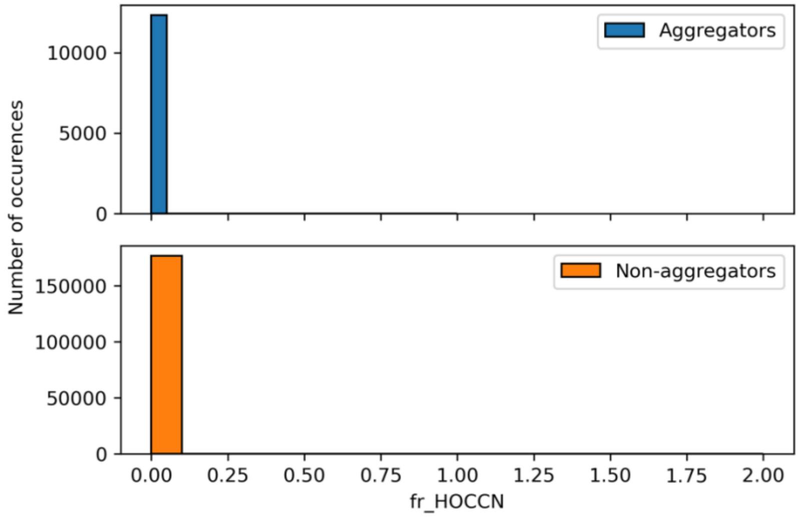
Aggregators
Non-aggregators
Number of occurences
10000
5000
0
150000
100000
50000
0
0.00 0.25 0.50 0.75 1.00 1.25 1.50 1.75 2.00
fr_HOCCN

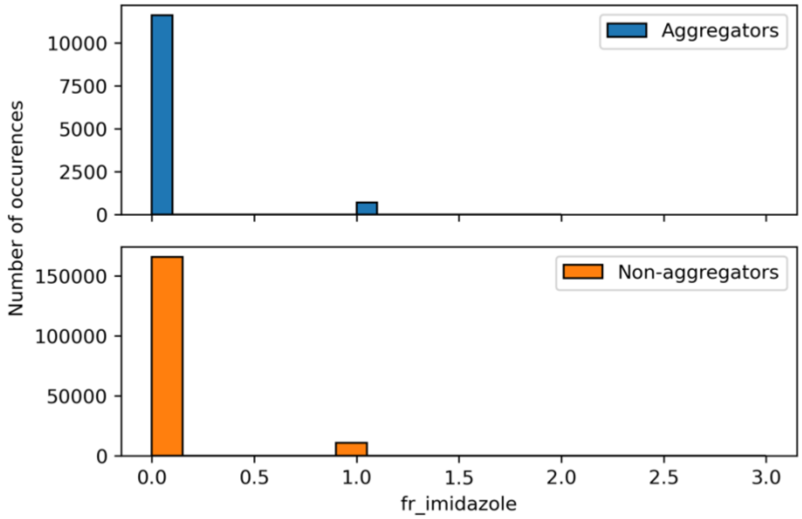
Aggregators
Non-aggregators
Number of occurences
10000
7500
5000
2500
0
150000
100000
50000
0
0.0 0.5 1.0 1.5 2.0 2.5 3.0
fr_imidazole

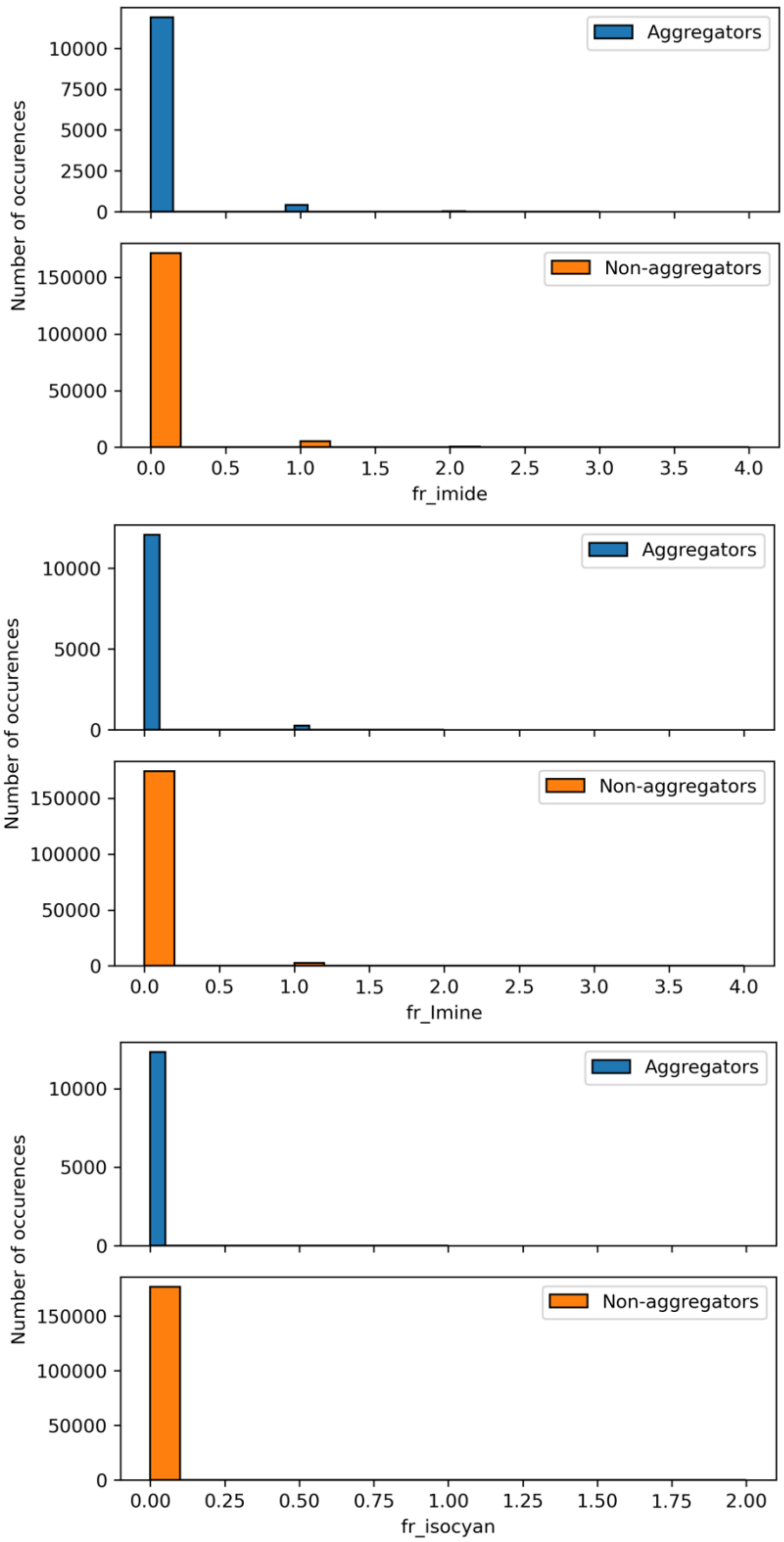

Aggregators
Non-aggregators
Number of occurences
fr_imide
fr_Imine
fr_isocyan

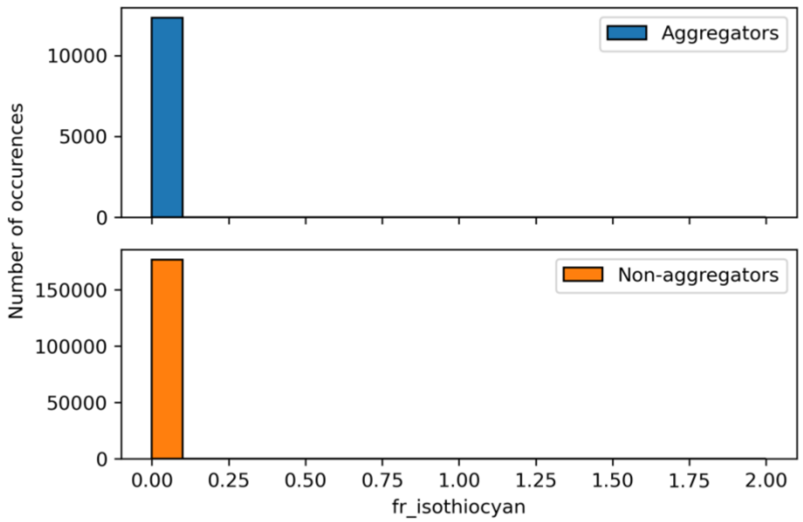
Aggregators
Non-aggregators
Number of occurences
10000
5000
0
150000
100000
50000
0
0.00
0.25
0.50
0.75
1.00
1.25
1.50
1.75
2.00
fr_isothiocyan

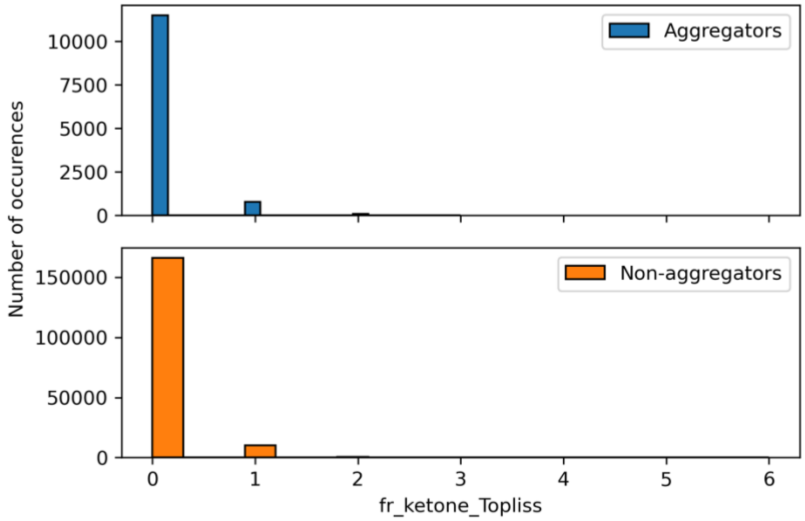
Aggregators
Non-aggregators
Number of occurences
10000
7500
5000
2500
0
150000
100000
50000
0
0
1
2
3
4
5
6
fr_ketone_Topliss

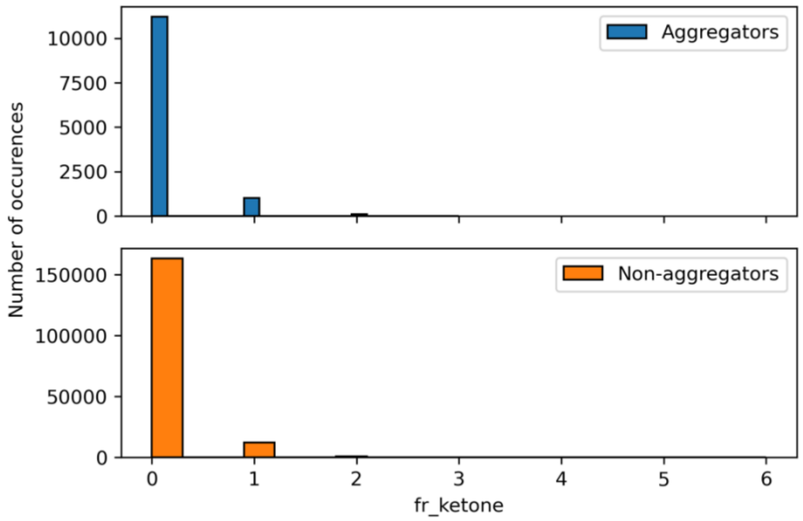
Aggregators
Non-aggregators
Number of occurences
10000
7500
5000
2500
0
150000
100000
50000
0
0
1
2
3
4
5
6
fr_ketone

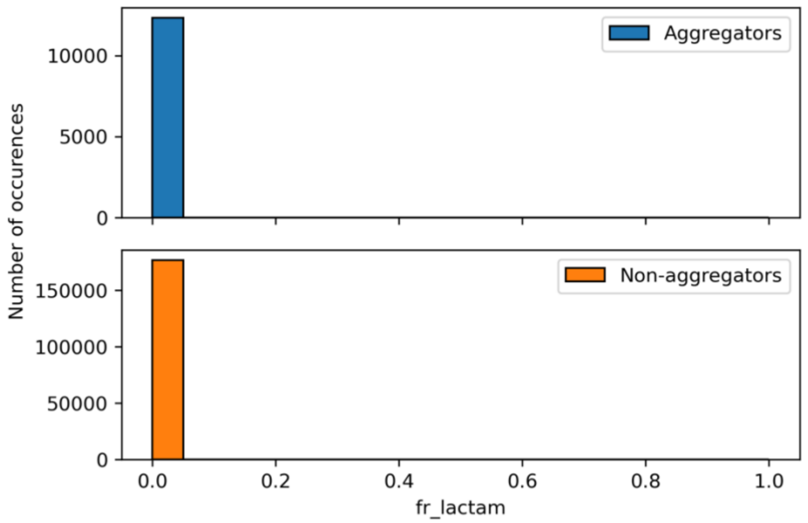
Aggregators
Non-aggregators
Number of occurences
10000
5000
0
150000
100000
50000
0
0.0
0.2
0.4
0.6
0.8
1.0
fr_lactam

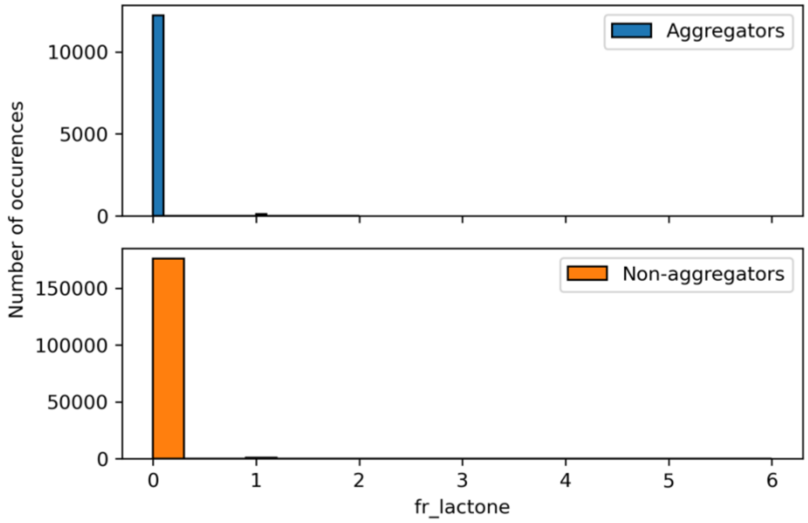
Aggregators
Non-aggregators
Number of occurences
10000
5000
0
150000
100000
50000
0
0
1
2
3
4
5
6
fr_lactone

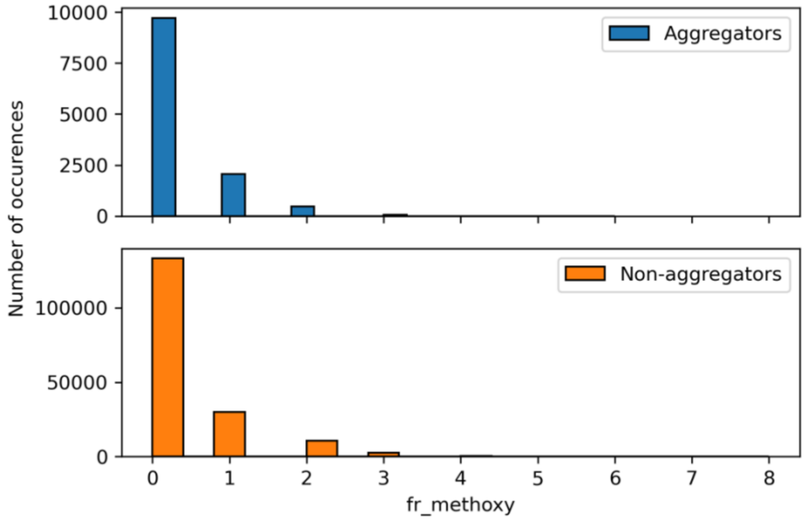
Aggregators
Non-aggregators
Number of occurences
10000
7500
5000
2500
0
100000
50000
0
0
1
2
3
4
5
6
7
8
fr_methoxy

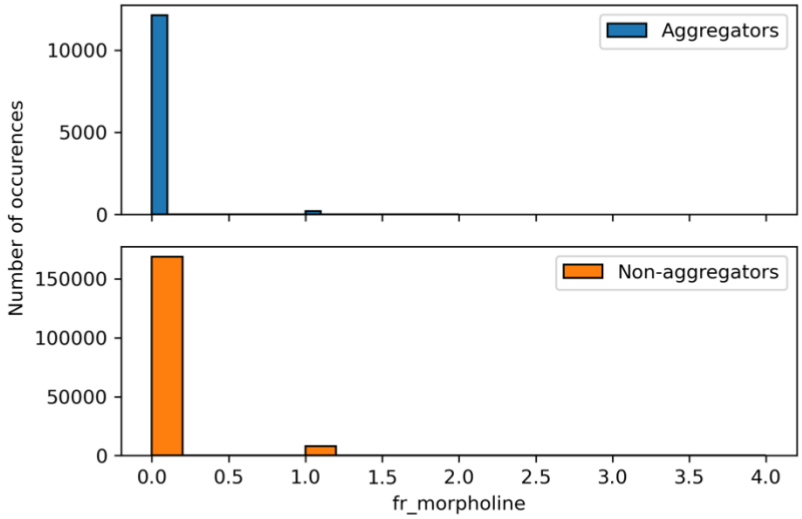

Aggregators
Non-aggregators
Number of occurences
10000
5000
0
150000
100000
50000
0
0.0 0.5 1.0 1.5 2.0 2.5 3.0 3.5 4.0
fr_morpholine

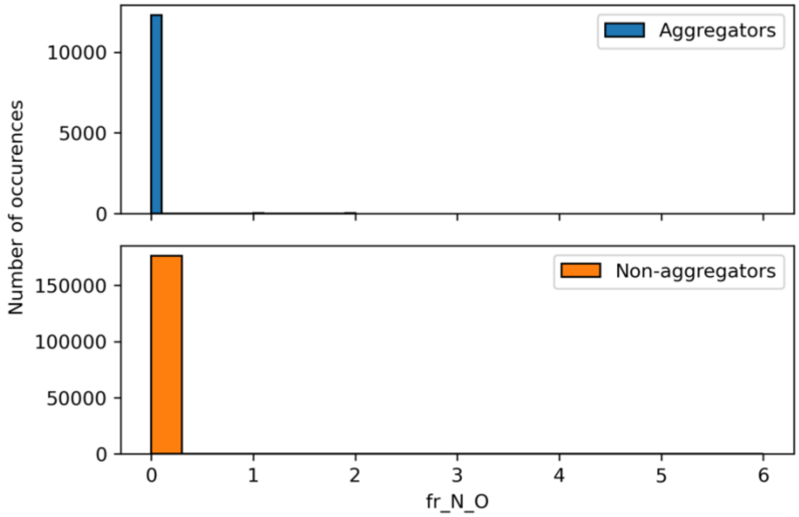

Aggregators
Non-aggregators
Number of occurences
10000
5000
0
150000
100000
50000
0
0 1 2 3 4 5 6
fr_N_O

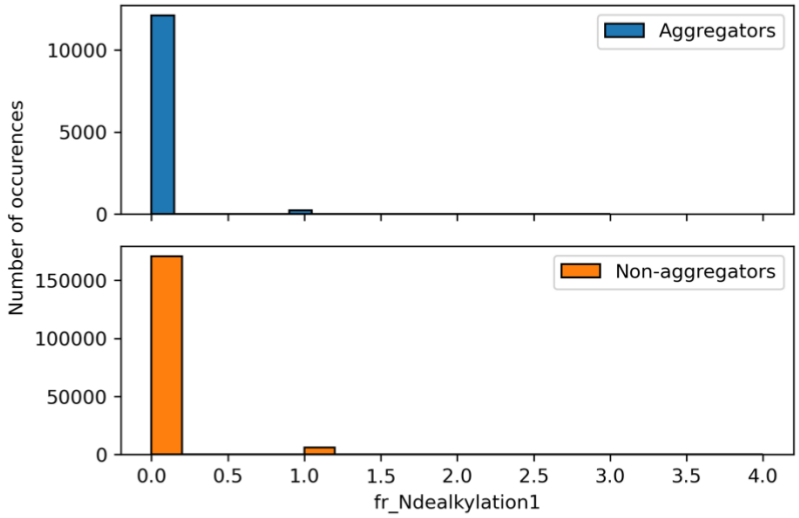

Aggregators
Non-aggregators
Number of occurences
10000
5000
0
150000
100000
50000
0
0.0 0.5 1.0 1.5 2.0 2.5 3.0 3.5 4.0
fr_Ndealkylation1

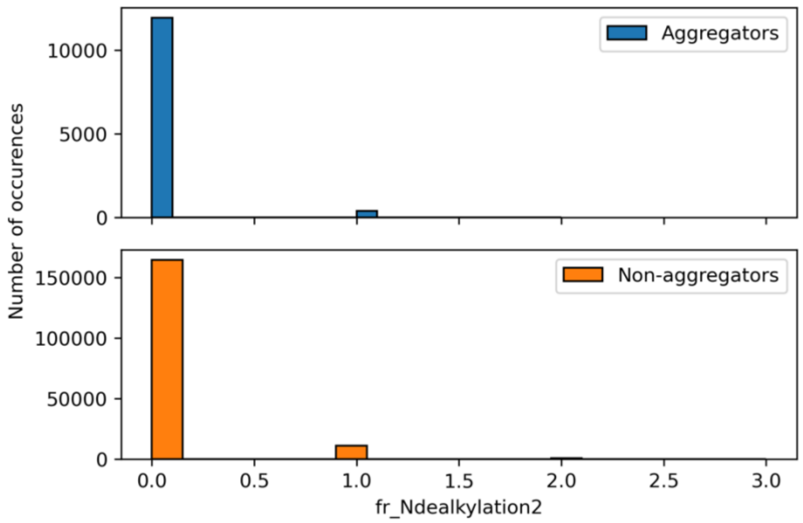

Aggregators
Non-aggregators
Number of occurences
fr_Ndealkylation2

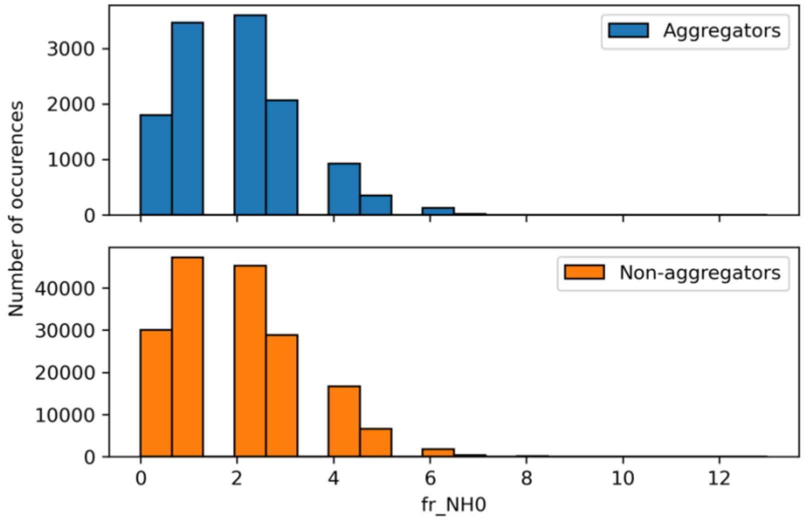

Aggregators
Non-aggregators
Number of occurences
fr_NH0

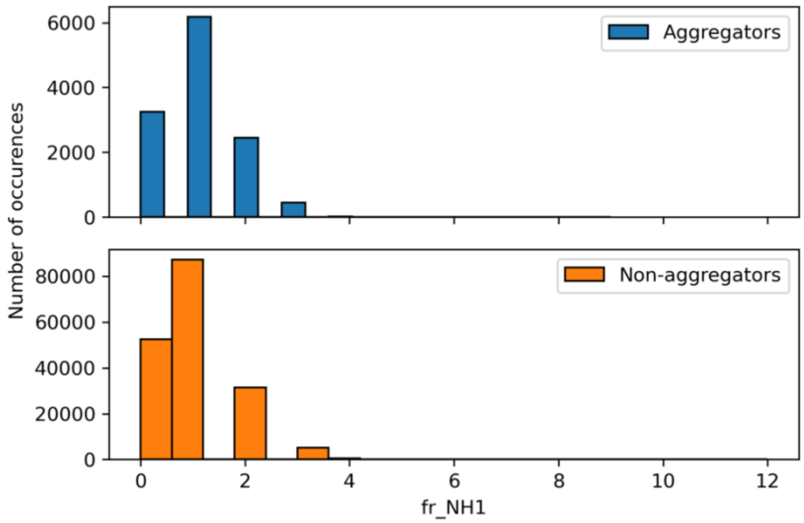

Aggregators
Non-aggregators
Number of occurences
fr_NH1

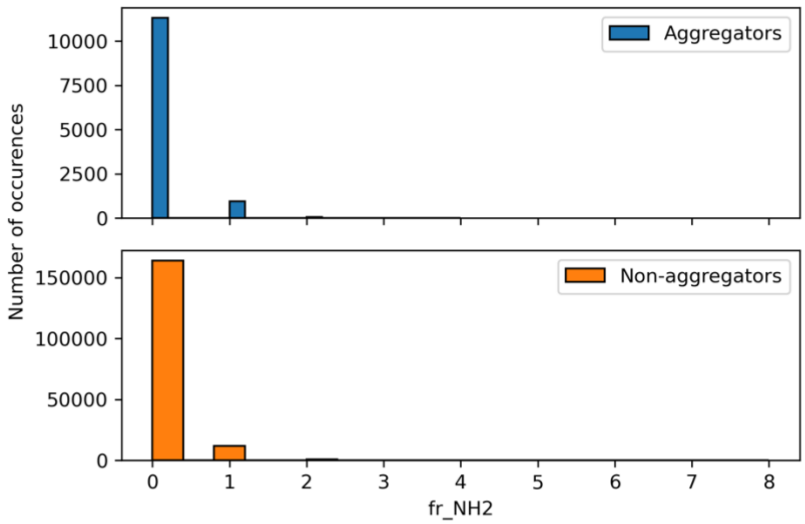
Aggregators
Non-aggregators
Number of occurences
fr_NH2

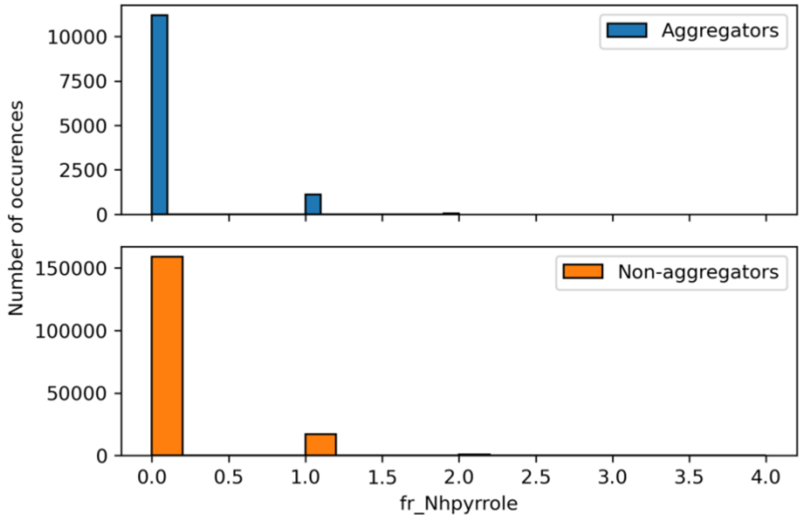
Aggregators
Non-aggregators
Number of occurences
fr_Nhpyrrole

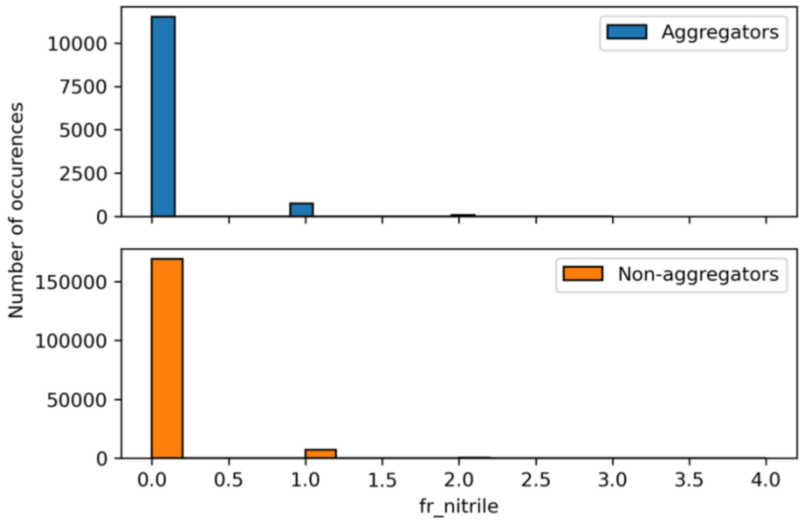
Aggregators
Non-aggregators
Number of occurences
fr_nitrile

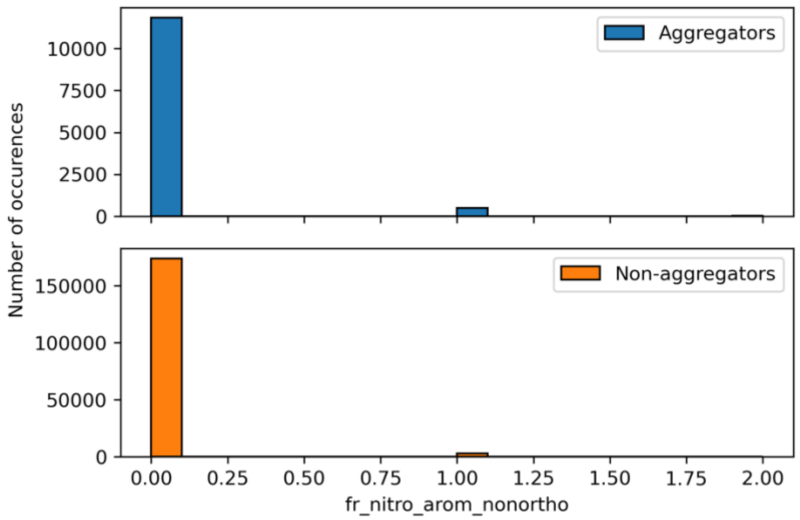
Aggregators
Non-aggregators
Number of occurences
fr_nitro_arom_nonortho
0.00
0.25
0.50
0.75
1.00
1.25
1.50
1.75
2.00
0
2500
5000
7500
10000
50000
100000
150000

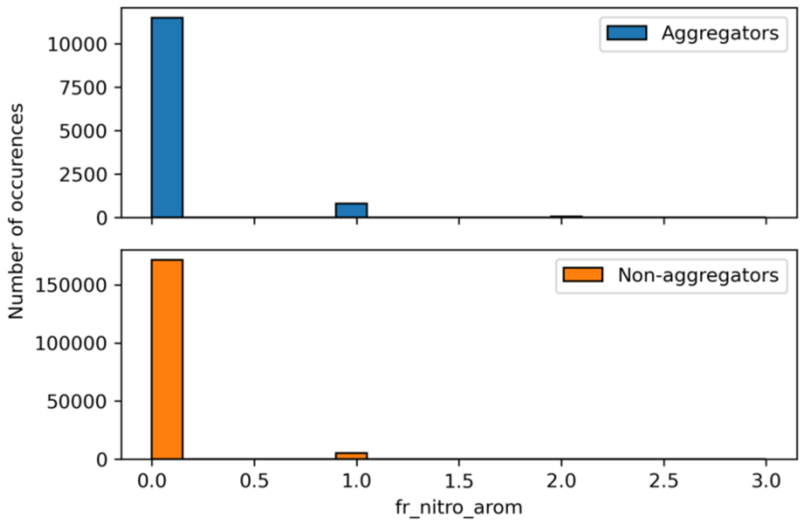
Aggregators
Non-aggregators
Number of occurences
fr_nitro_arom
0.0
0.5
1.0
1.5
2.0
2.5
3.0
0
2500
5000
7500
10000
50000
100000
150000

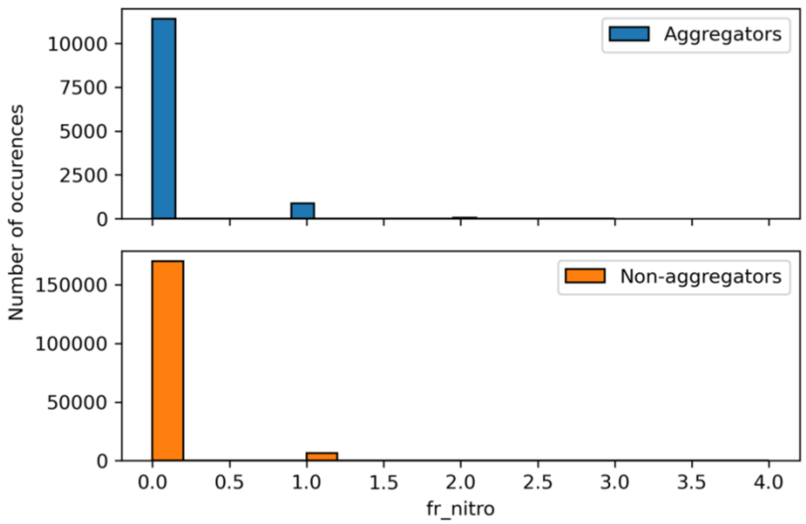
Aggregators
Non-aggregators
Number of occurences
fr_nitro
0.0
0.5
1.0
1.5
2.0
2.5
3.0
3.5
4.0
0
2500
5000
7500
10000
50000
100000
150000

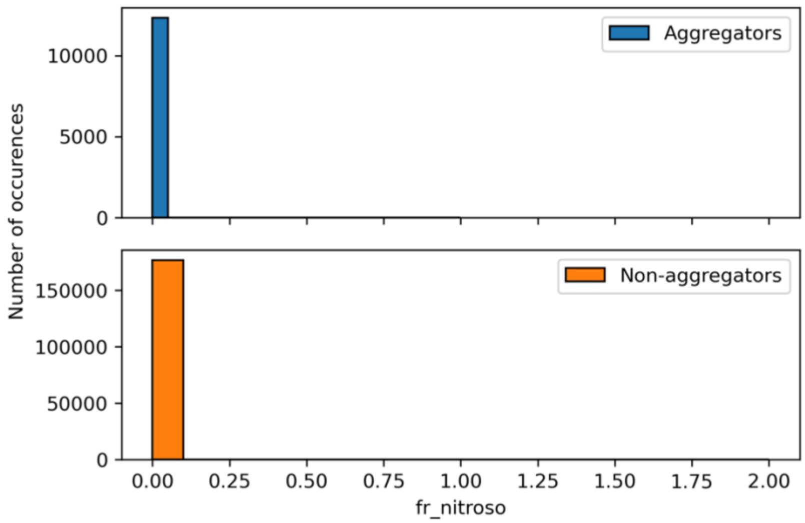
Aggregators
Non-aggregators
Number of occurences
0
5000
10000
0
50000
100000
150000
0.00
0.25
0.50
0.75
1.00
1.25
1.50
1.75
2.00
fr_nitroso

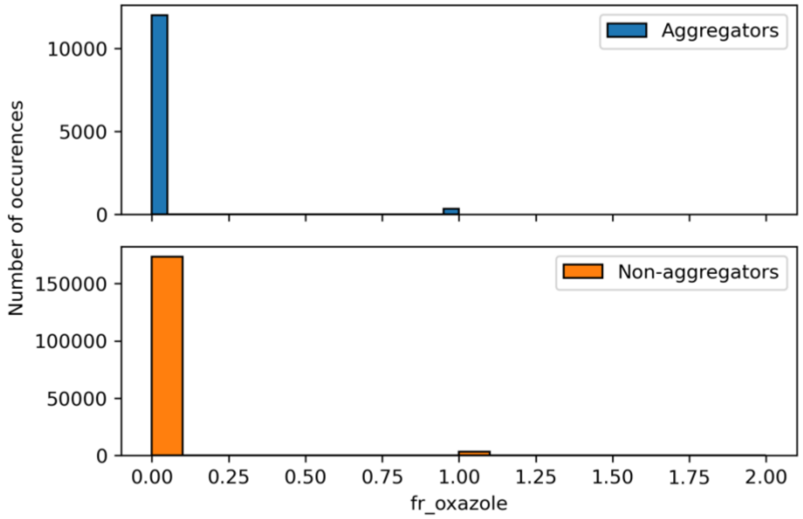
Aggregators
Non-aggregators
Number of occurences
0
5000
10000
0
50000
100000
150000
0.00
0.25
0.50
0.75
1.00
1.25
1.50
1.75
2.00
fr_oxazole

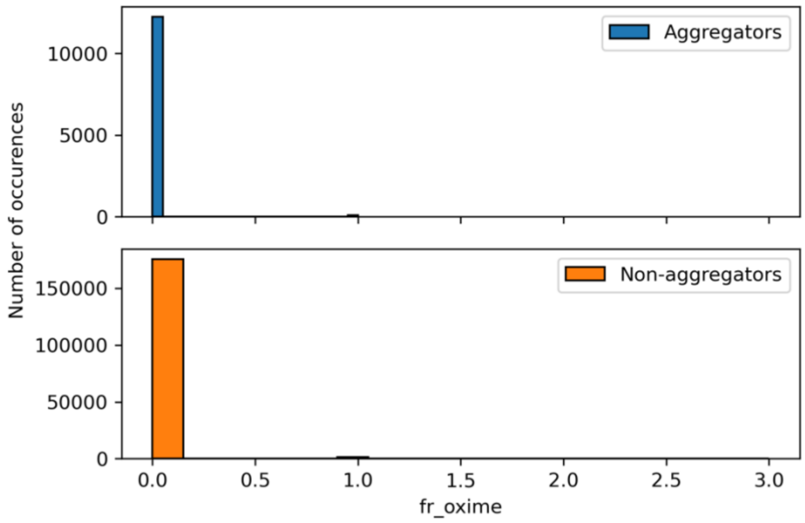
Aggregators
Non-aggregators
Number of occurences
0
5000
10000
0
50000
100000
150000
0.0
0.5
1.0
1.5
2.0
2.5
3.0
fr_oxime

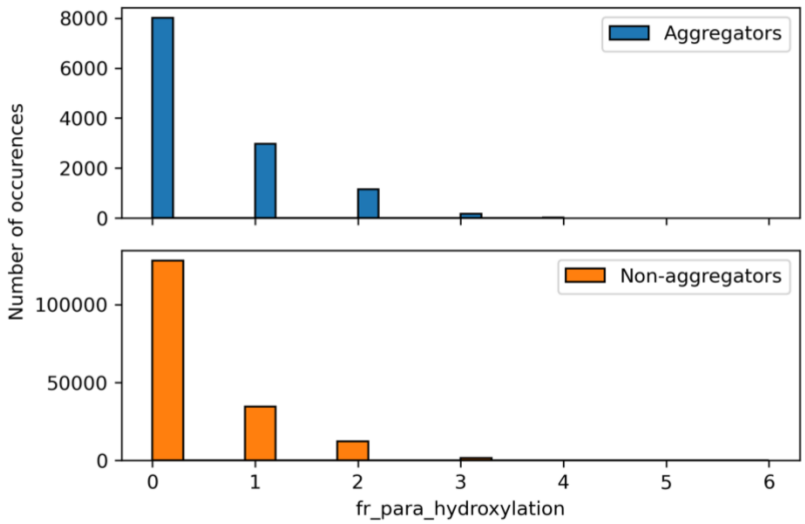
Aggregators
Non-aggregators
Number of occurences
8000
6000
4000
2000
0
100000
50000
0
0
1
2
3
4
5
6
fr_para_hydroxylation

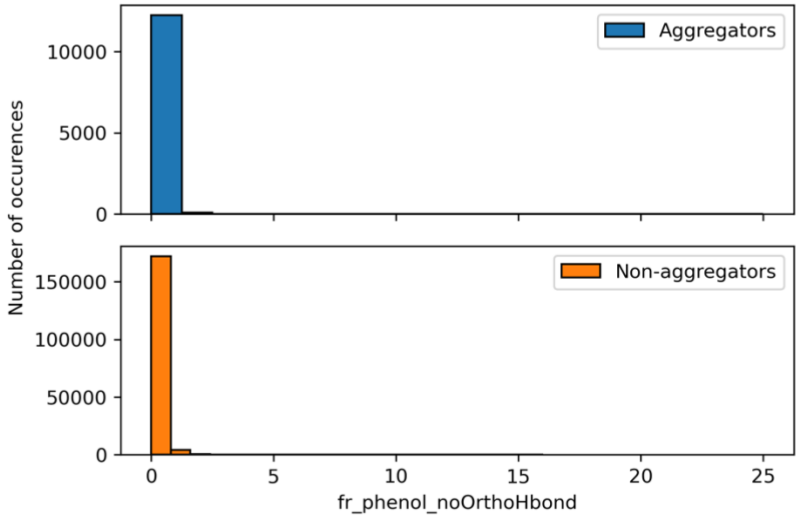
Aggregators
Non-aggregators
Number of occurences
10000
5000
0
150000
100000
50000
0
0
5
10
15
20
25
fr_phenol_noOrthoHbond

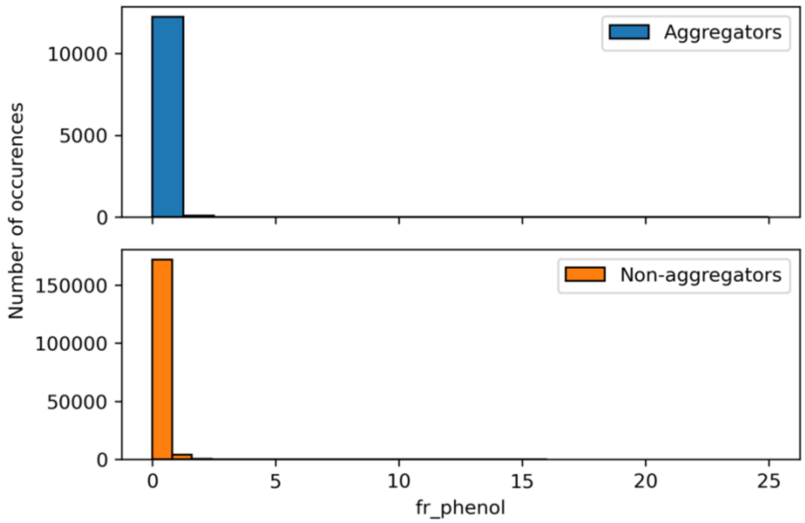
Aggregators
Non-aggregators
Number of occurences
10000
5000
0
150000
100000
50000
0
0
5
10
15
20
25
fr_phenol

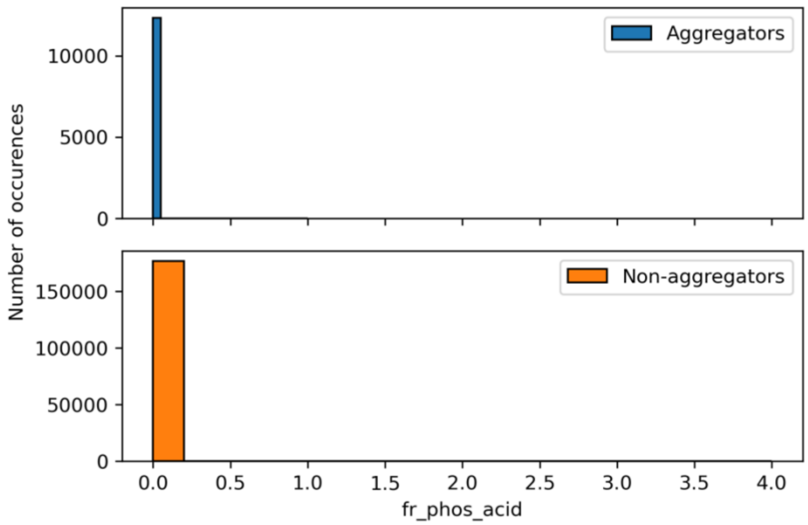

Aggregators
Non-aggregators
Number of occurences
fr_phos_acid

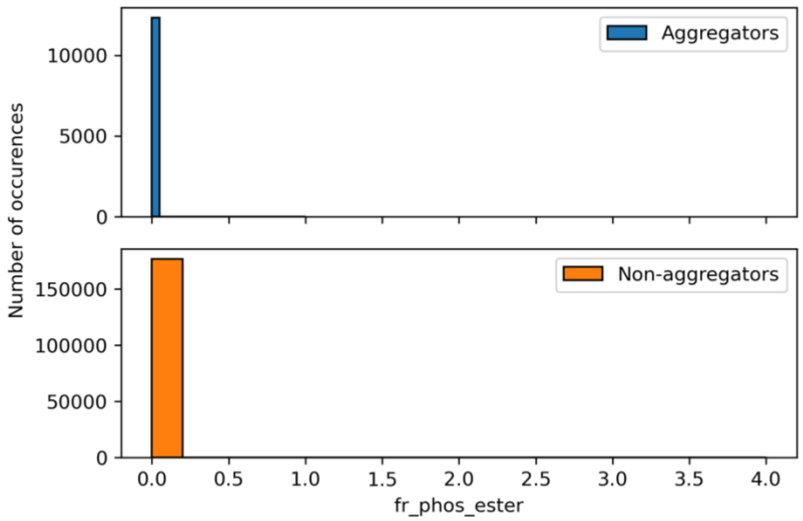

Aggregators
Non-aggregators
Number of occurences
fr_phos_ester

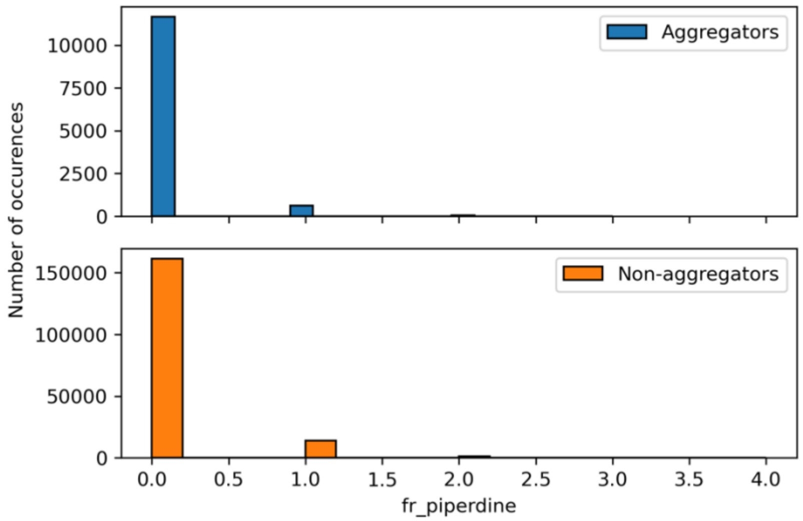

Aggregators
Non-aggregators
Number of occurences
fr_piperdine

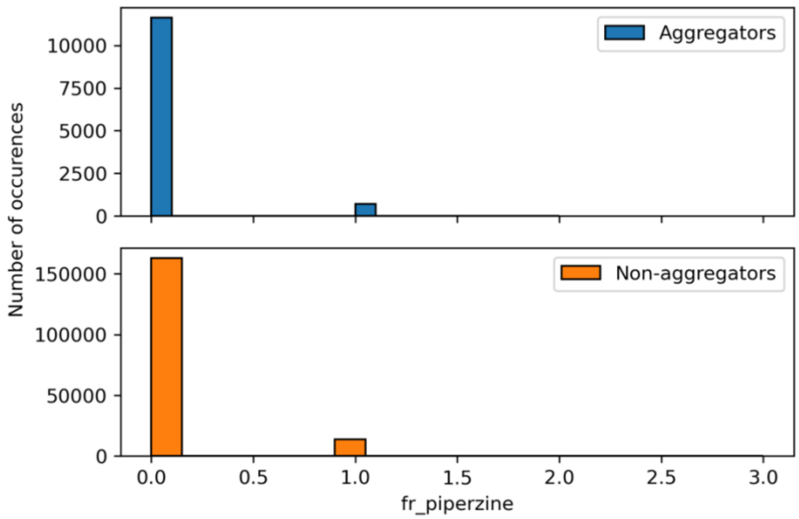

Aggregators
Non-aggregators
Number of occurences
fr_piperzine

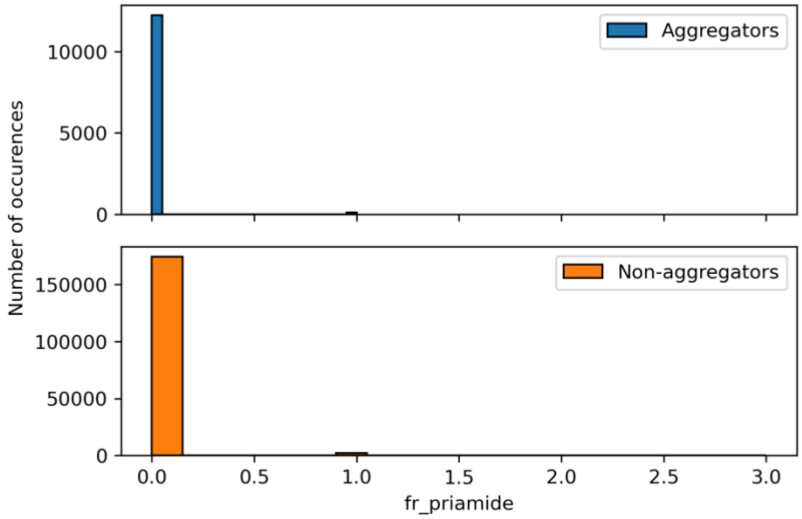

Aggregators
Non-aggregators
Number of occurences
fr_priamide

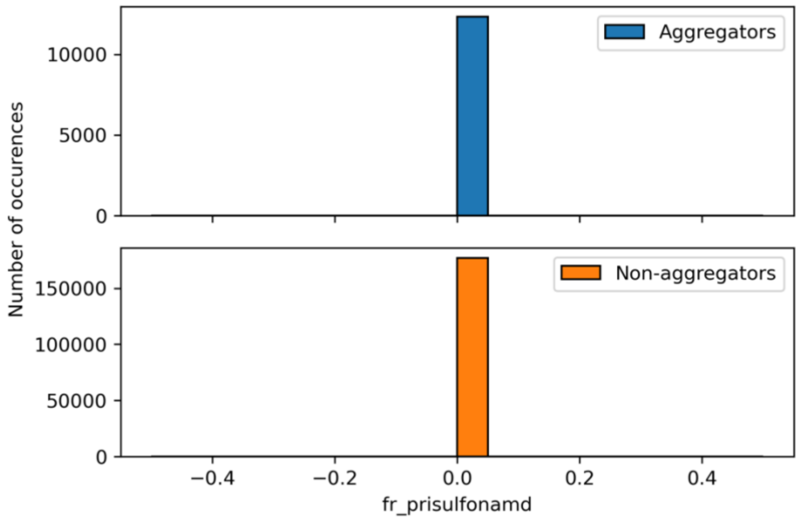

Aggregators
Non-aggregators
Number of occurences
fr_prisulfonamd

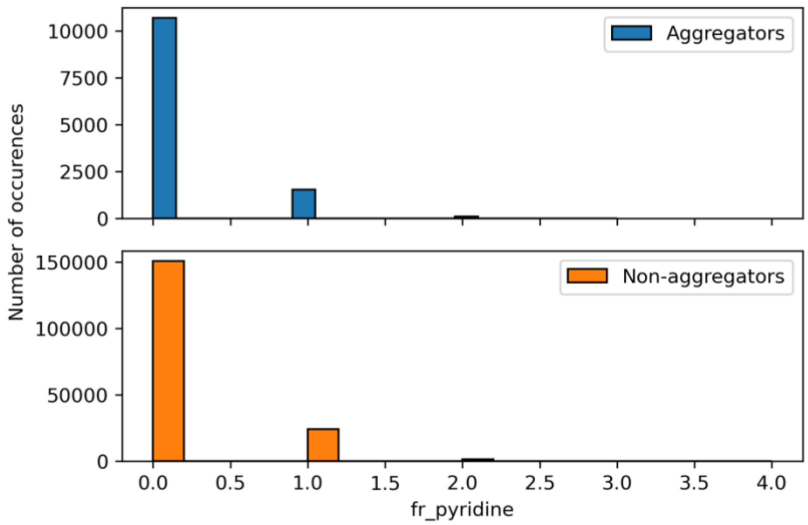
Aggregators
Non-aggregators
Number of occurences
10000
7500
5000
2500
0
150000
100000
50000
0
0.0
0.5
1.0
1.5
2.0
2.5
3.0
3.5
4.0
fr_pyridine

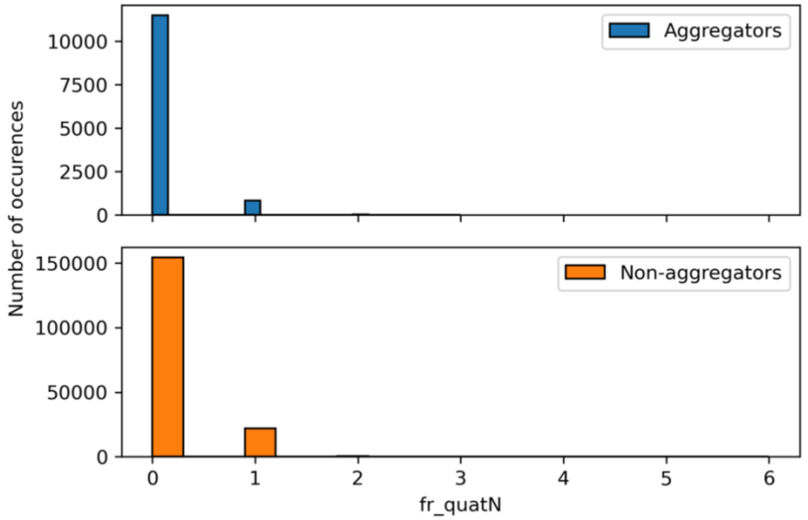
Aggregators
Non-aggregators
Number of occurences
10000
7500
5000
2500
0
150000
100000
50000
0
0
1
2
3
4
5
6
fr_quatN

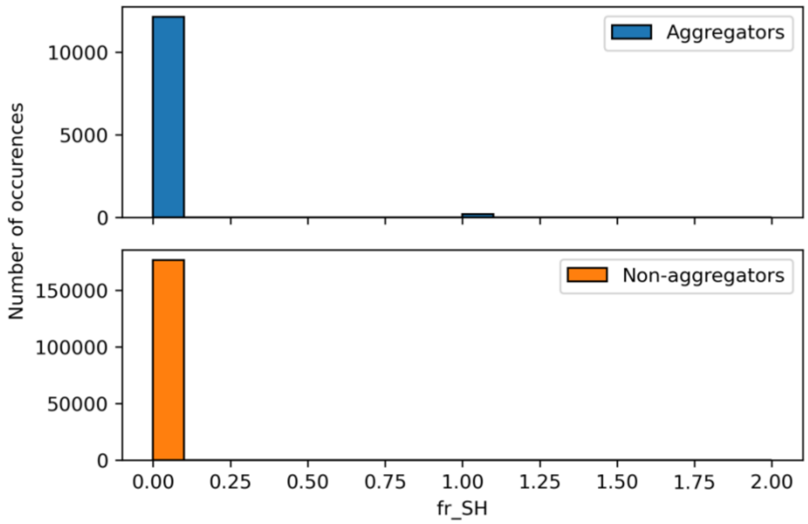
Aggregators
Non-aggregators
Number of occurences
10000
5000
0
150000
100000
50000
0
0.00
0.25
0.50
0.75
1.00
1.25
1.50
1.75
2.00
fr_SH

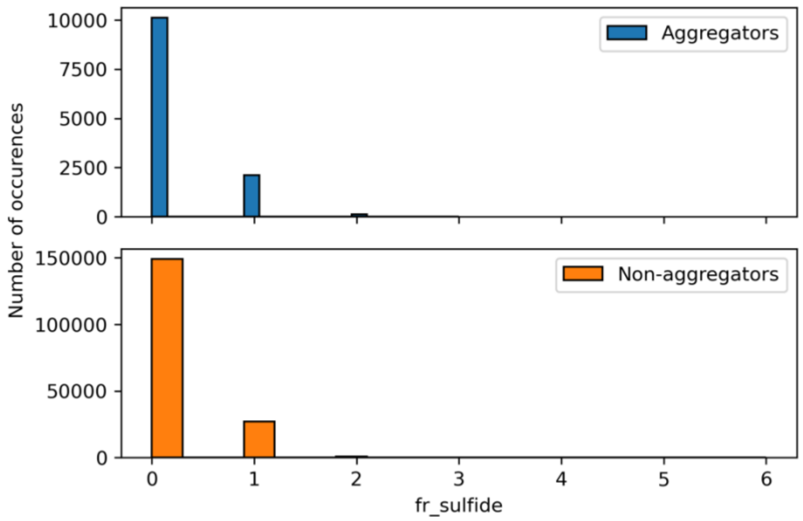
Aggregators
Non-aggregators
Number of occurences
fr_sulfide

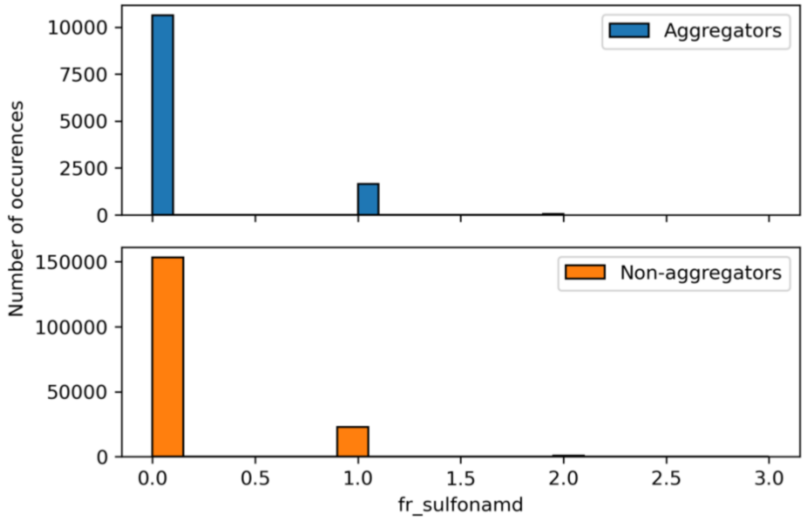
Aggregators
Non-aggregators
Number of occurences
fr_sulfonamd

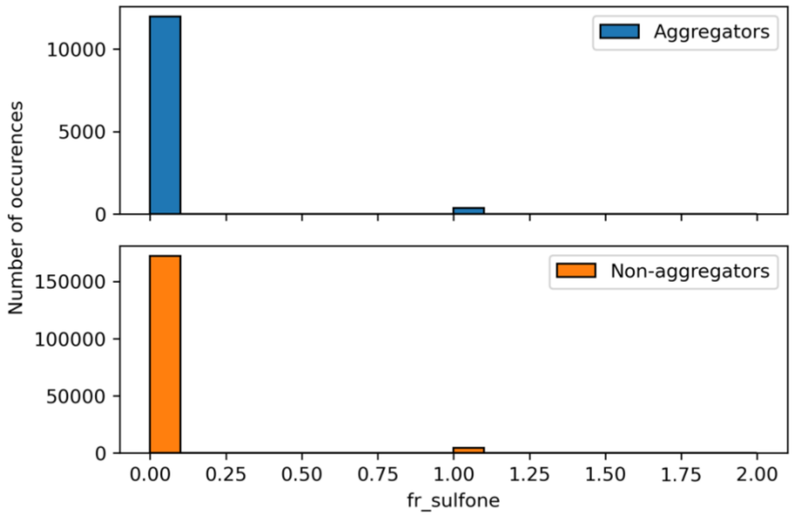
Aggregators
Non-aggregators
Number of occurences
fr_sulfone

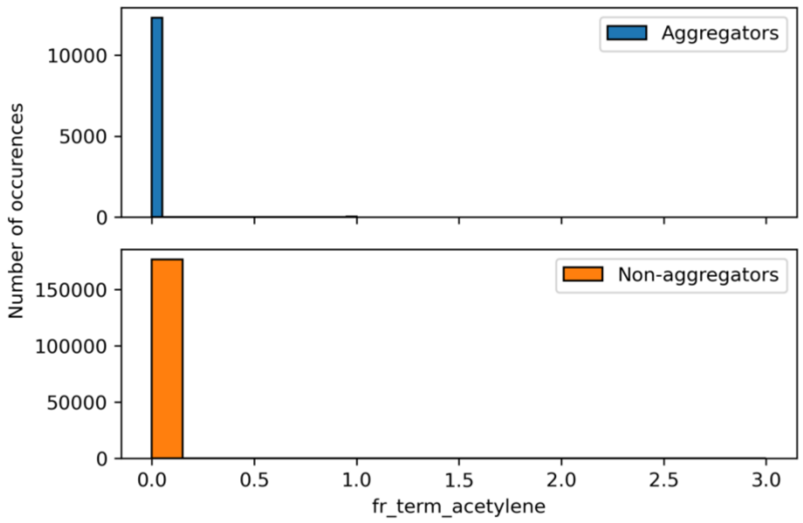

Aggregators
Non-aggregators
Number of occurences
fr_term_acetylene

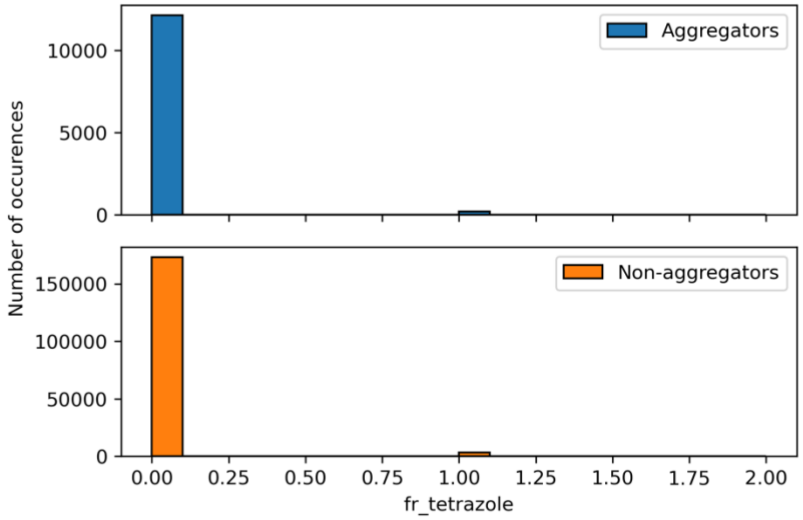

Aggregators
Non-aggregators
Number of occurences
fr_tetrazole

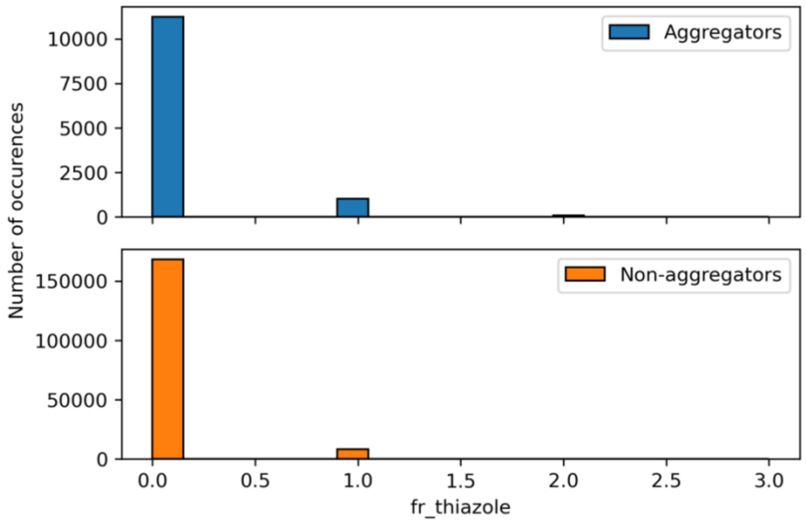

Aggregators
Non-aggregators
Number of occurences
fr_thiazole

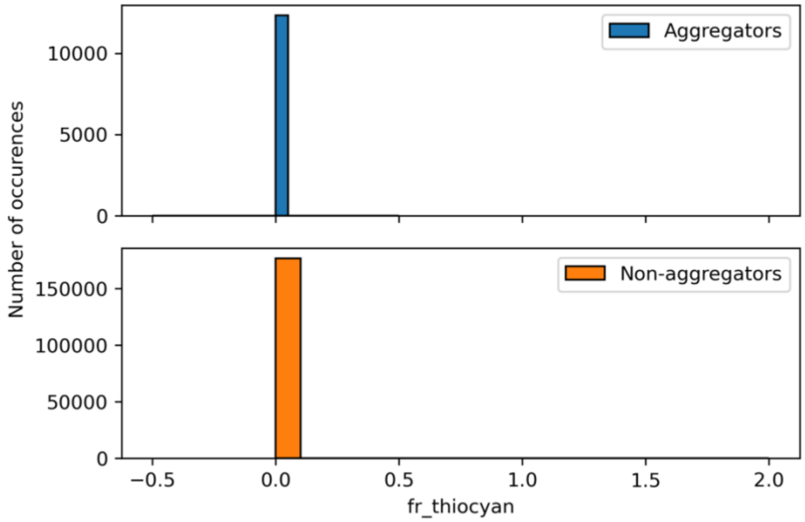
Aggregators
Non-aggregators
Number of occurences
fr_thiocyan

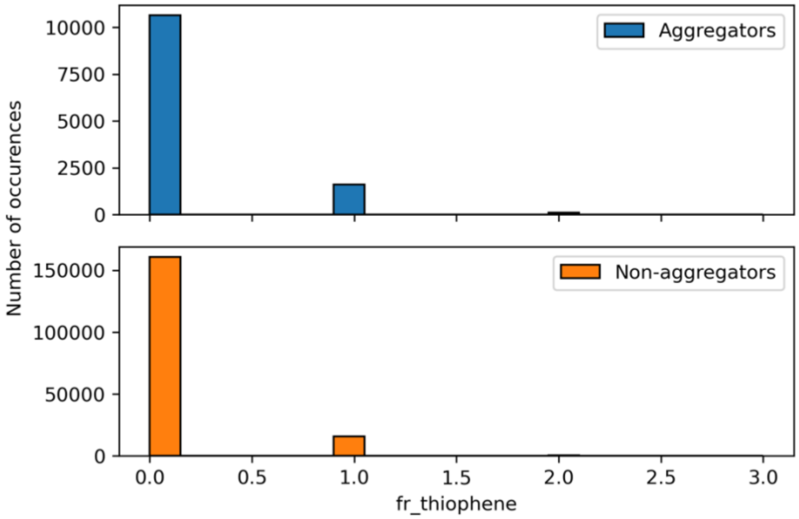
Aggregators
Non-aggregators
Number of occurences
fr_thiophene

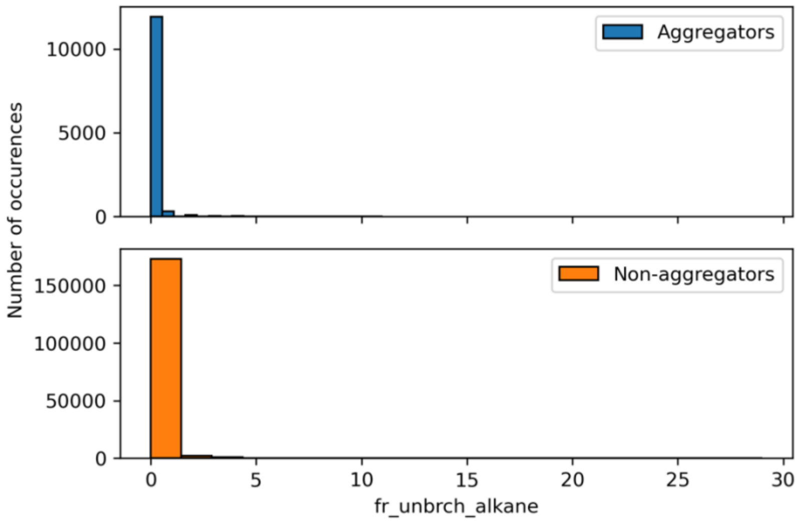
Aggregators
Non-aggregators
Number of occurences
fr_unbrch_alkane

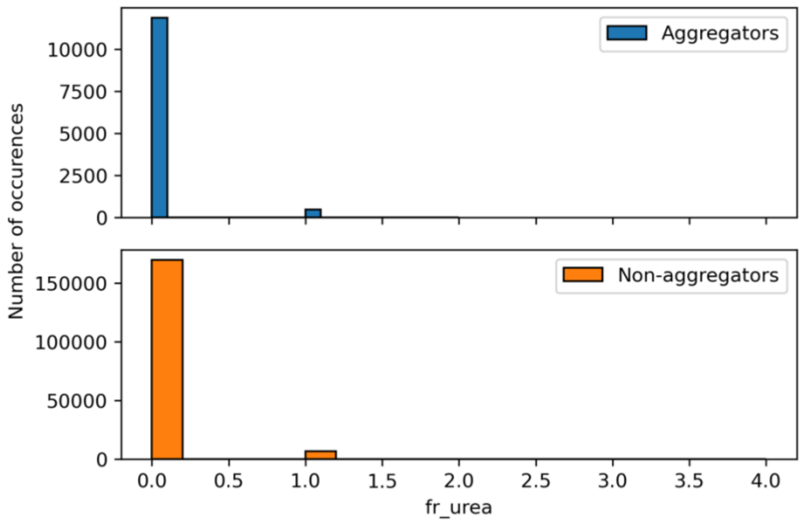
Aggregators
Non-aggregators
Number of occurences
fr_urea
10000
7500
5000
2500
0
150000
100000
50000
0
0.0
0.5
1.0
1.5
2.0
2.5
3.0
3.5
4.0

SXV. p-values, means, standard deviations and Cohen's d values for the 15 statistically significant general molecular descriptors calculated for the aggregator and non-aggregator datasets

Table S3. Statistics for the 15 statistically significant general molecular descriptors calculated for the aggregator and non-aggregator datasets.

Molecular Descriptor	p-Value	Aggregators Mean	Aggregators Std. Dev.	Non-aggregators Mean	Non-aggregators Std. Dev.	Cohen's D	|Cohen's_D|
MolLogP	0.000	3.888	1.455	2.906	1.581	0.647	0.647
NumAromaticCarbocycles	0.000	1.940	0.873	1.459	0.802	0.573	0.573
FractionCSP3	0.000	0.208	0.143	0.296	0.174	-0.557	0.557
NumAromaticRings	0.000	3.027	0.958	2.475	1.044	0.551	0.551
RingCount	0.000	3.681	1.029	3.182	1.092	0.471	0.471
MolMR	0.000	107.895	20.149	98.343	22.730	0.445	0.445
HeavyAtomMolWt	0.000	377.782	74.078	345.340	79.833	0.421	0.421
LabuteASA	0.000	164.944	30.714	151.925	34.314	0.400	0.400
ExactMolWt	0.000	396.802	76.906	365.012	83.778	0.395	0.395
HeavyAtomCount	0.000	27.697	5.375	25.592	5.952	0.371	0.371
NumValenceElectrons	0.000	141.820	27.812	133.437	31.001	0.285	0.285
NumHeteroatoms	0.000	7.074	2.168	6.863	2.206	0.097	0.097
NumHAcceptors	0.000	5.015	1.868	4.892	1.887	0.065	0.065
TPSA	0.000	74.603	27.422	73.071	27.139	0.056	0.056
NOCount	0.006	5.771	1.920	5.822	1.972	-0.026	0.026

SXVI. Comparison of the relative frequency of each of the fragments described by the fragment descriptors for the aggregator and non-aggregator molecules

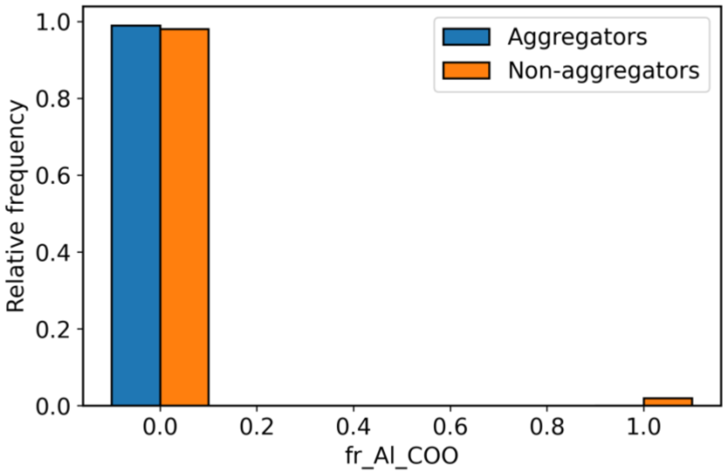

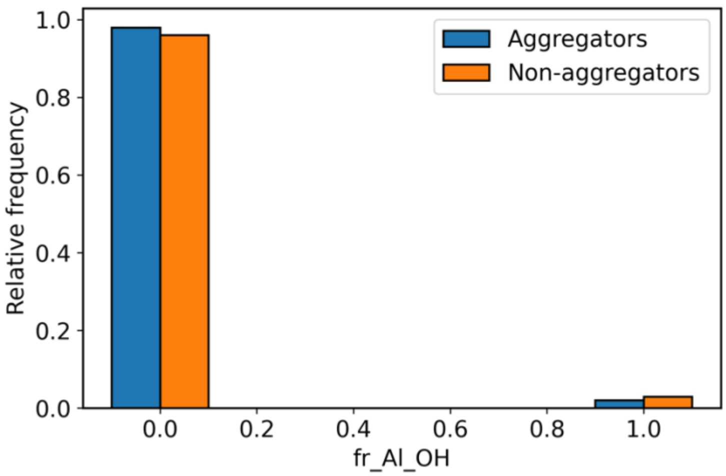

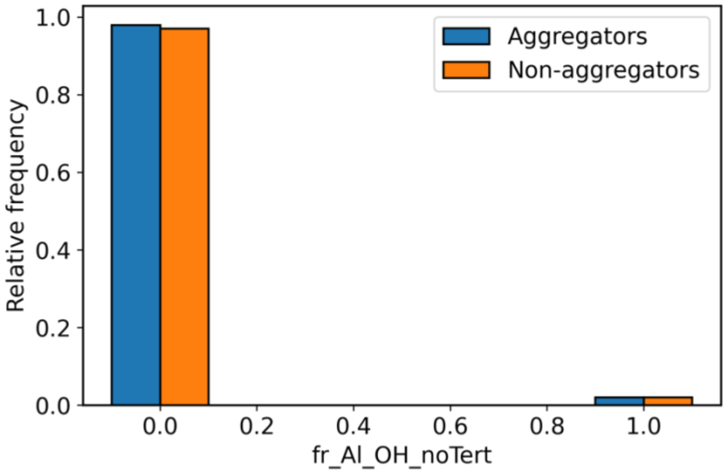

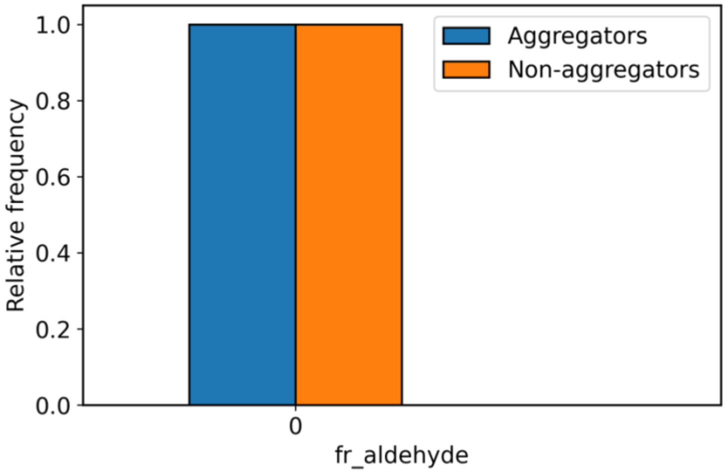

Aggregators
Non-aggregators
Relative frequency
1.0
0.8
0.6
0.4
0.2
0.0
0
fr_aldehyde

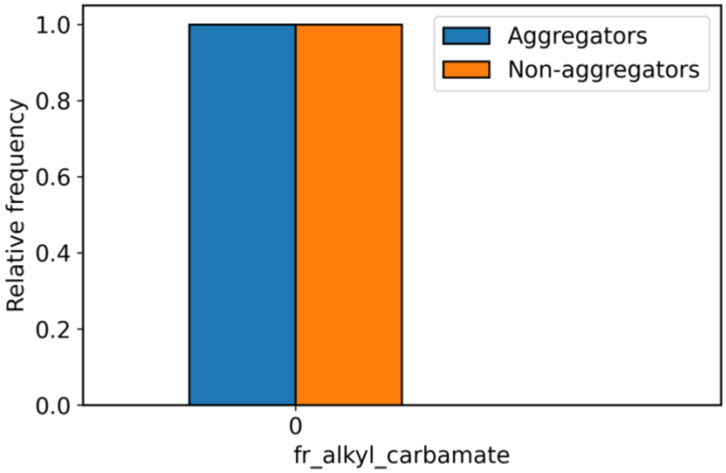

Aggregators
Non-aggregators
Relative frequency
1.0
0.8
0.6
0.4
0.2
0.0
0
fr_alkyl_carbamate

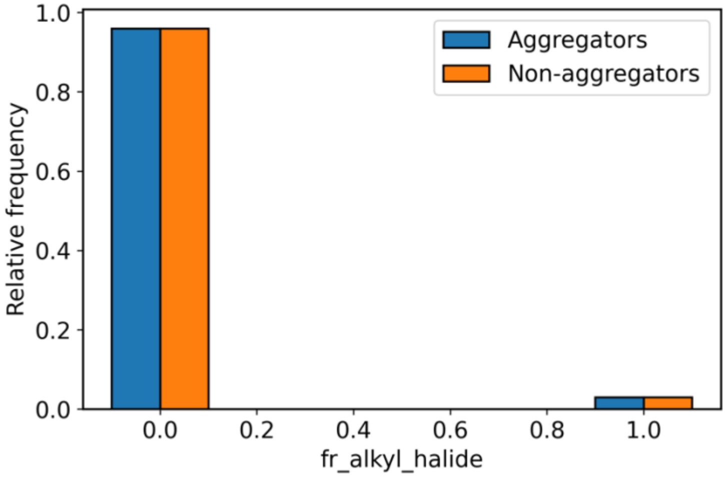

Aggregators
Non-aggregators
Relative frequency
1.0
0.8
0.6
0.4
0.2
0.0
0.0
0.2
0.4
0.6
0.8
1.0
fr_alkyl_halide

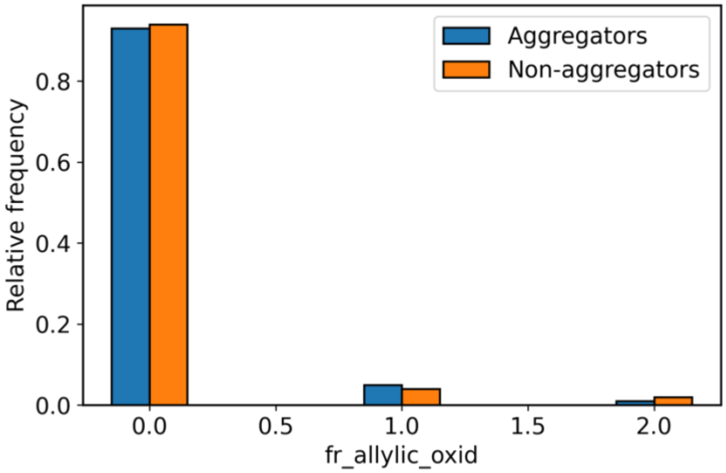

Aggregators
Non-aggregators
Relative frequency
0.0
0.2
0.4
0.6
0.8
0.0
0.5
1.0
1.5
2.0
fr_allylic_oxid

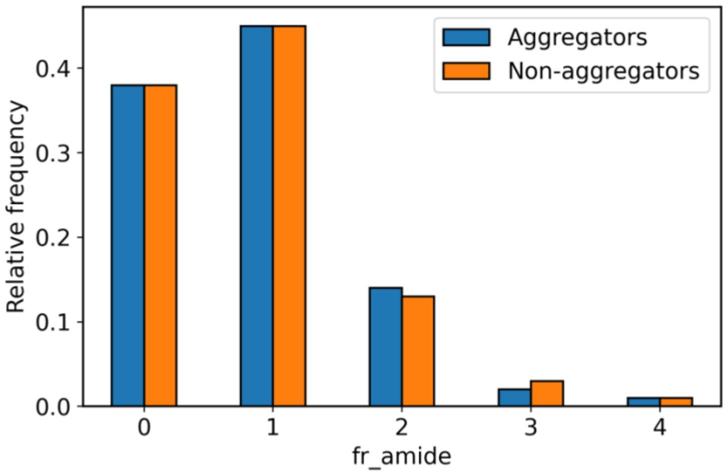

Aggregators
Non-aggregators
Relative frequency
0.0
0.1
0.2
0.3
0.4
0
1
2
3
4
fr_amide

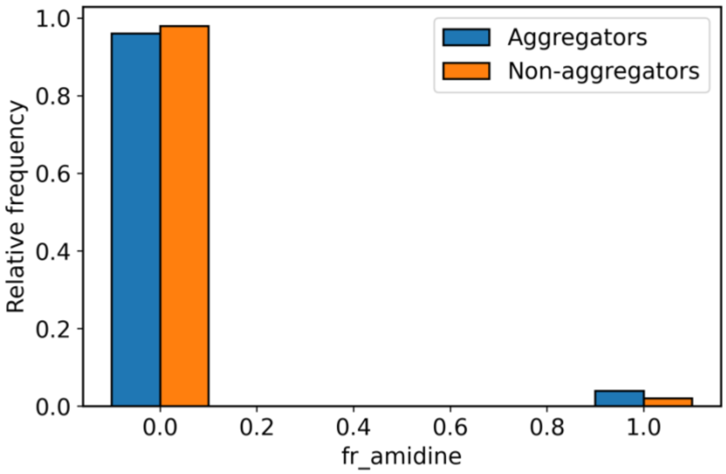

Aggregators
Non-aggregators
Relative frequency
0.0
0.2
0.4
0.6
0.8
1.0
0.0
0.2
0.4
0.6
0.8
1.0
fr_amidine

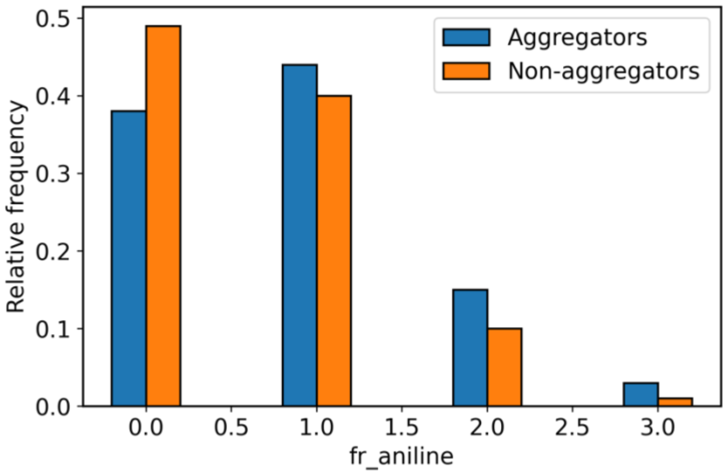
Aggregators
Non-aggregators
Relative frequency
0.0
0.1
0.2
0.3
0.4
0.5
0.0
0.5
1.0
1.5
2.0
2.5
3.0
fr_aniline

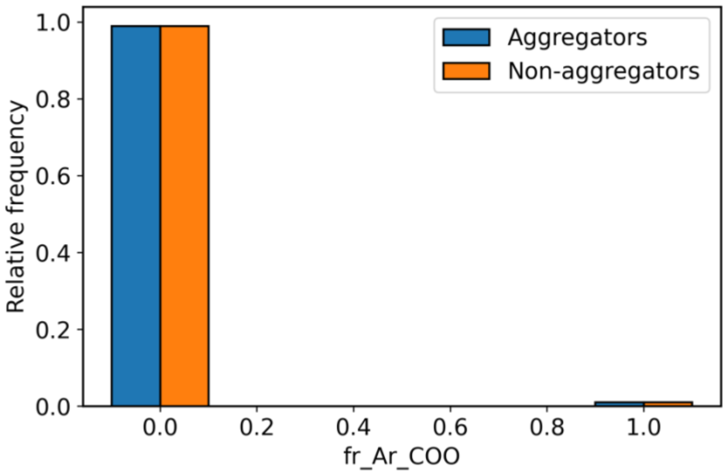
Aggregators
Non-aggregators
Relative frequency
0.0
0.2
0.4
0.6
0.8
1.0
0.0
0.2
0.4
0.6
0.8
1.0
fr_Ar_COO

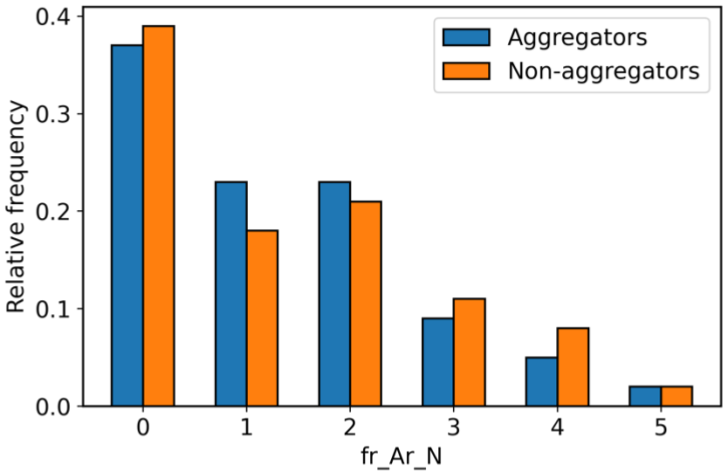
Aggregators
Non-aggregators
Relative frequency
0.0
0.1
0.2
0.3
0.4
0
1
2
3
4
5
fr_Ar_N

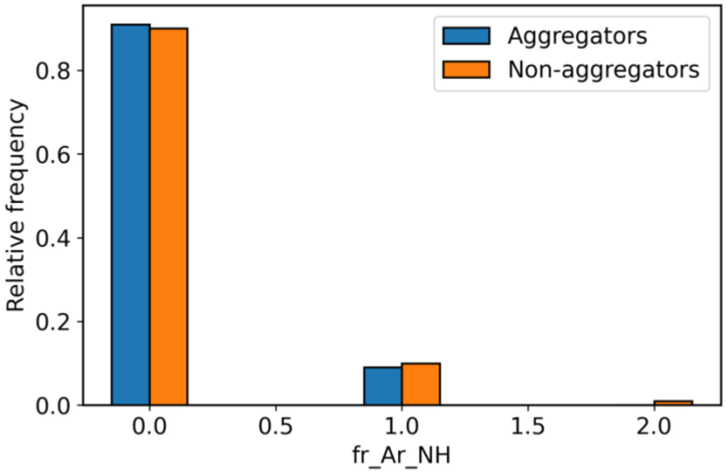

Aggregators
Non-aggregators
Relative frequency
0.0
0.2
0.4
0.6
0.8
0.0
0.5
1.0
1.5
2.0
fr_Ar_NH

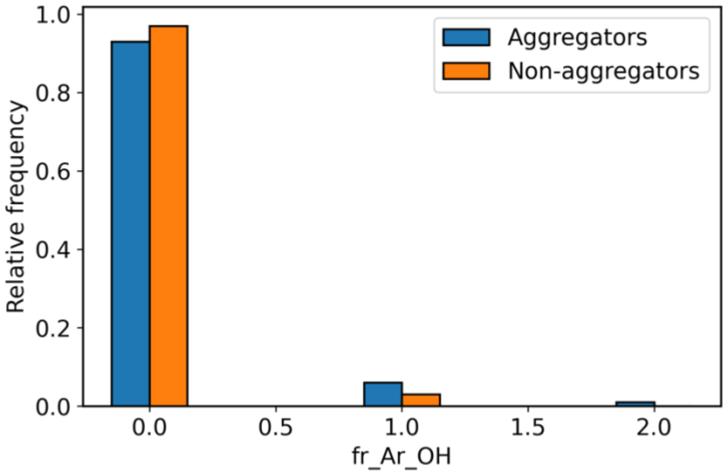

Aggregators
Non-aggregators
Relative frequency
0.0
0.2
0.4
0.6
0.8
1.0
0.0
0.5
1.0
1.5
2.0
fr_Ar_OH

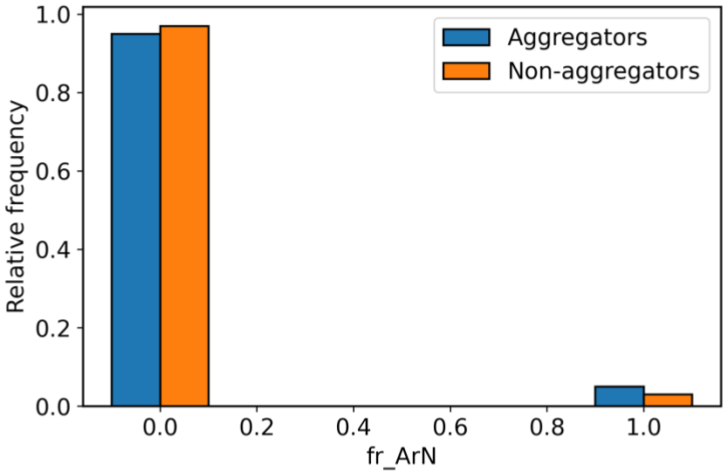

Aggregators
Non-aggregators
Relative frequency
0.0
0.2
0.4
0.6
0.8
1.0
0.0
0.2
0.4
0.6
0.8
1.0
fr_ArN

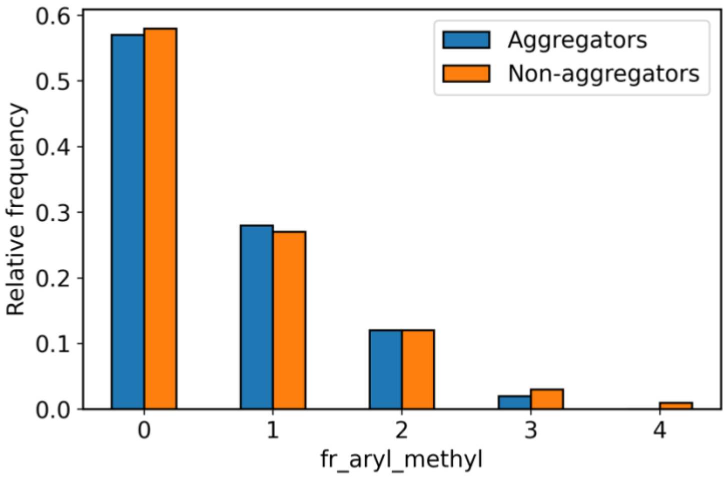
Aggregators
Non-aggregators
Relative frequency
0.0
0.1
0.2
0.3
0.4
0.5
0.6
0
1
2
3
4
fr_aryl_methyl

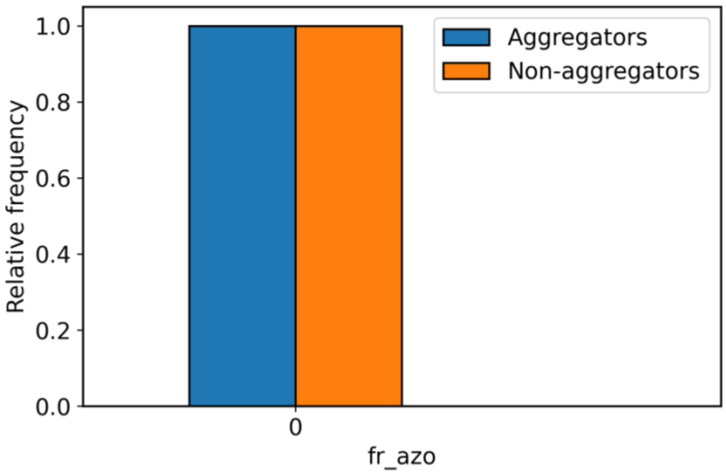
Aggregators
Non-aggregators
Relative frequency
0.0
0.2
0.4
0.6
0.8
1.0
0
fr_azo

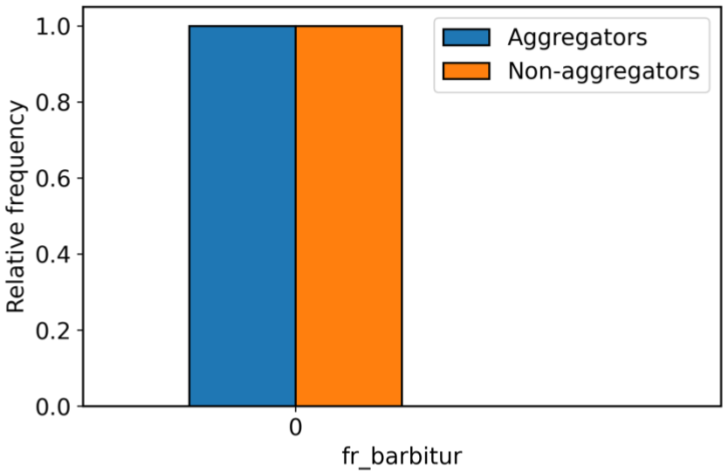
Aggregators
Non-aggregators
Relative frequency
0.0
0.2
0.4
0.6
0.8
1.0
0
fr_barbitur

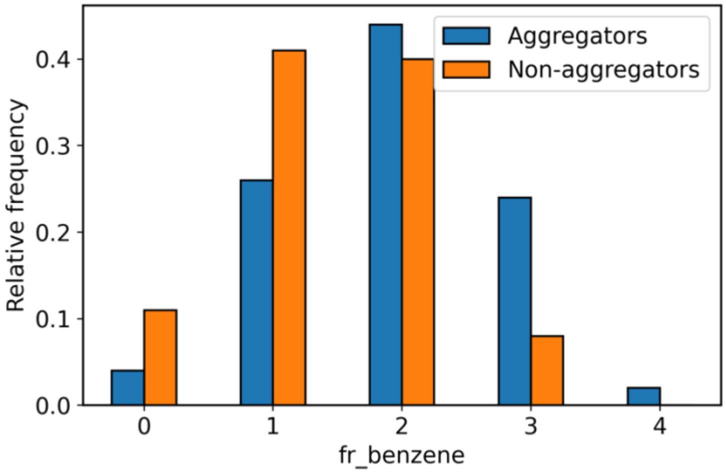
Aggregators
Non-aggregators
Relative frequency
fr_benzene

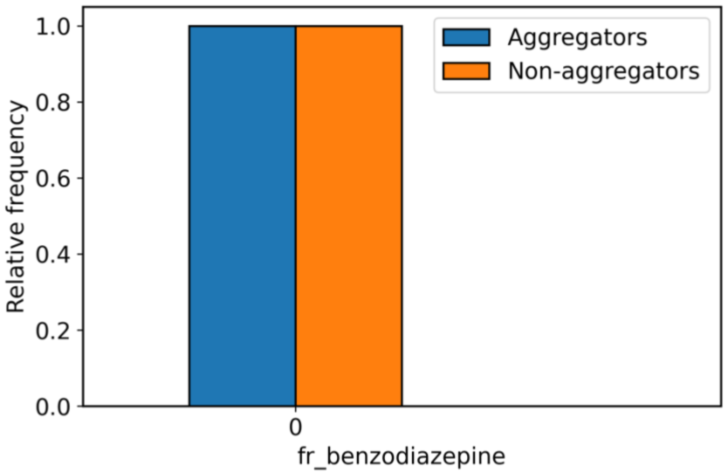
Aggregators
Non-aggregators
Relative frequency
fr_benzodiazepine

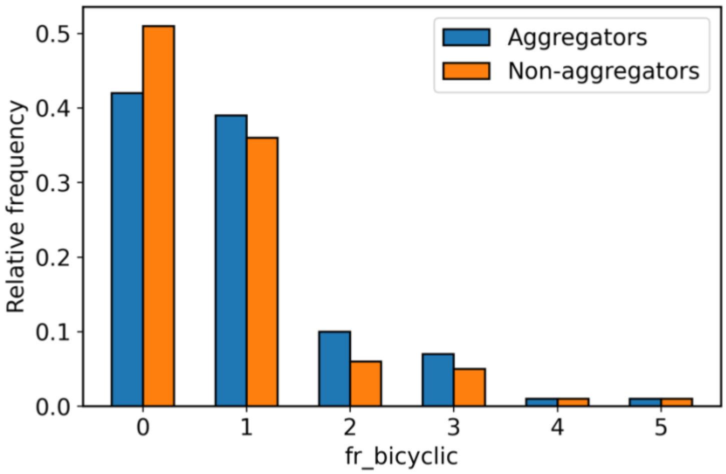
Aggregators
Non-aggregators
Relative frequency
fr_bicyclic

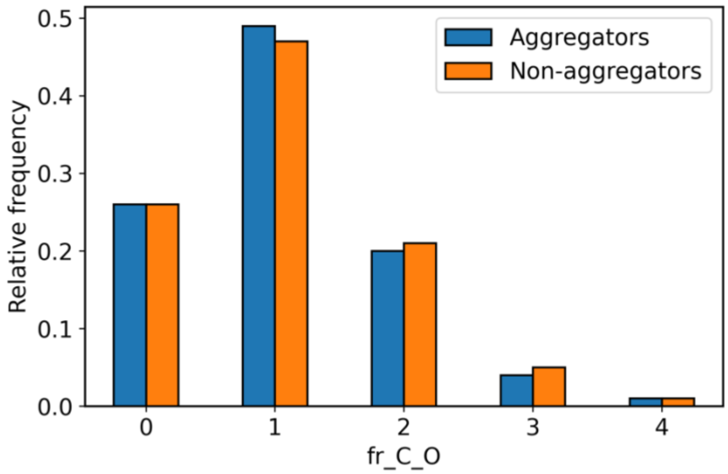
Aggregators
Non-aggregators
Relative frequency
fr_C_O

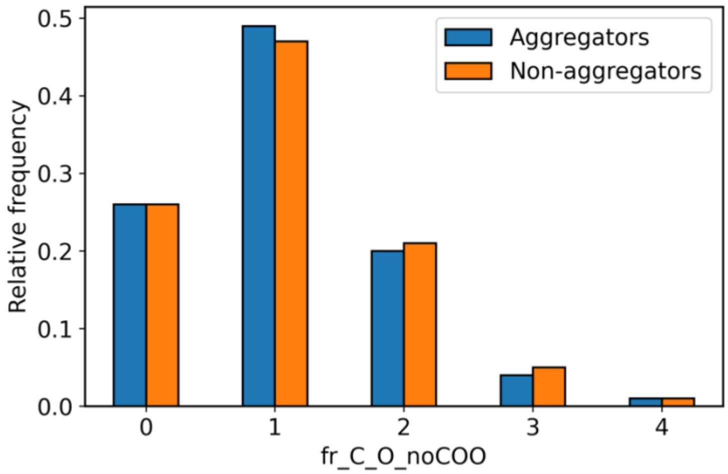
Aggregators
Non-aggregators
Relative frequency
fr_C_O_noCOO

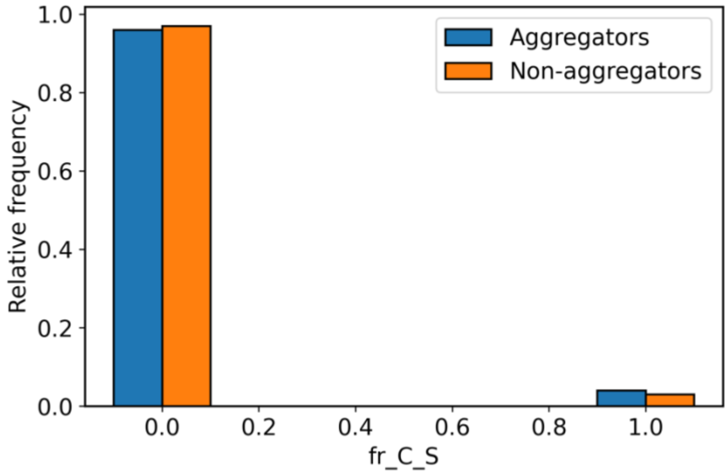
Aggregators
Non-aggregators
Relative frequency
fr_C_S

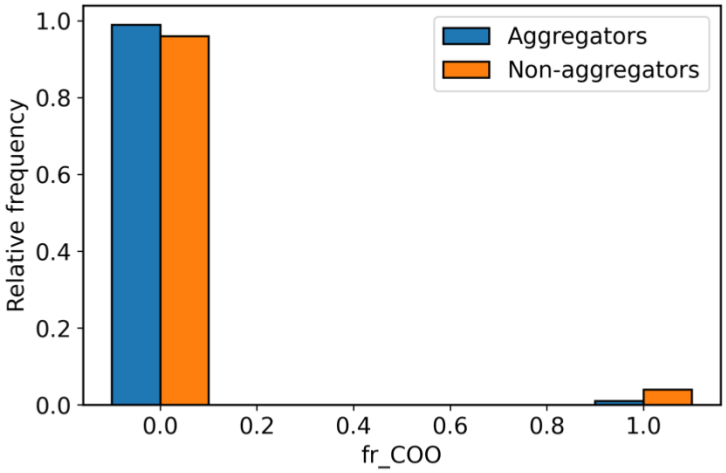

Aggregators
Non-aggregators
Relative frequency
1.0
0.8
0.6
0.4
0.2
0.0
0.0
0.2
0.4
0.6
0.8
1.0
fr_COO

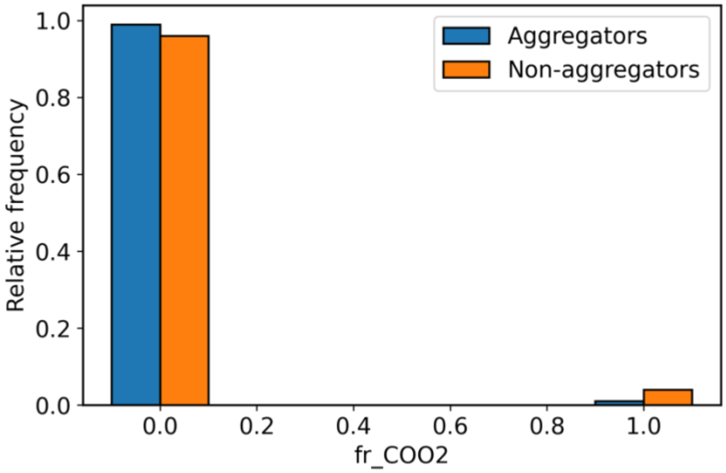

Aggregators
Non-aggregators
Relative frequency
1.0
0.8
0.6
0.4
0.2
0.0
0.0
0.2
0.4
0.6
0.8
1.0
fr_COO2

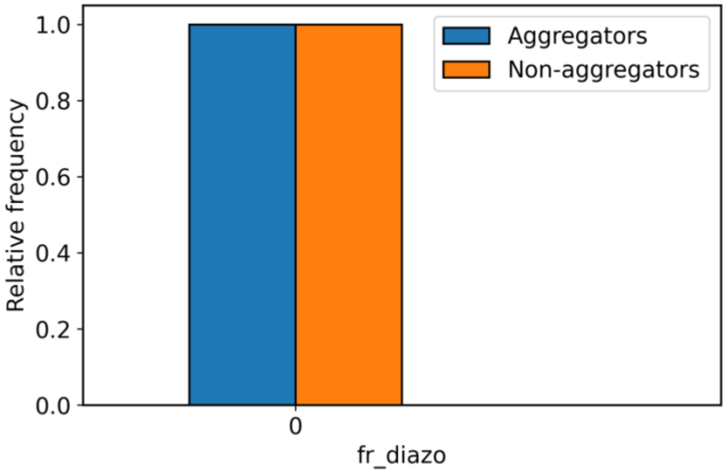

Aggregators
Non-aggregators
Relative frequency
1.0
0.8
0.6
0.4
0.2
0.0
0
fr_diazo

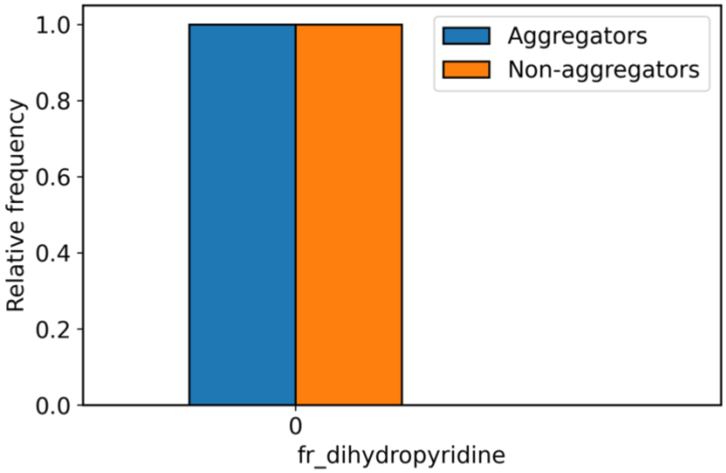
Aggregators
Non-aggregators
Relative frequency
1.0
0.8
0.6
0.4
0.2
0.0
0
fr_dihydropyridine

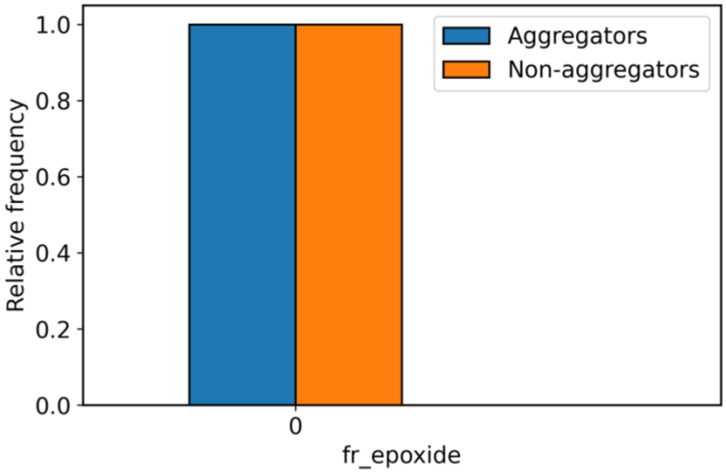
Aggregators
Non-aggregators
Relative frequency
1.0
0.8
0.6
0.4
0.2
0.0
0
fr_epoxide

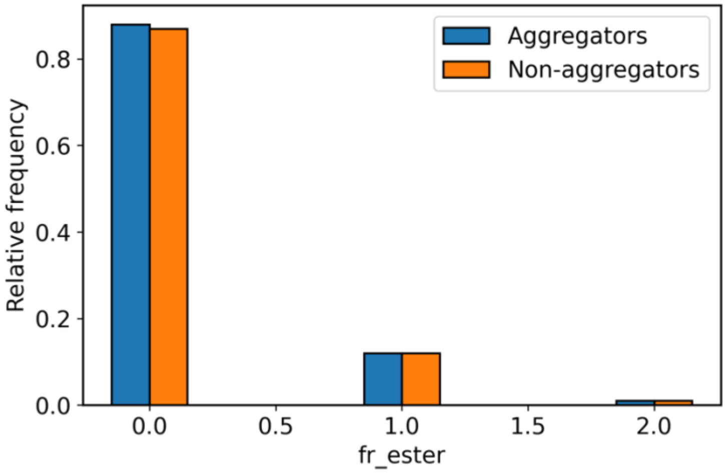
Aggregators
Non-aggregators
Relative frequency
0.8
0.6
0.4
0.2
0.0
0.0
0.5
1.0
1.5
2.0
fr_ester

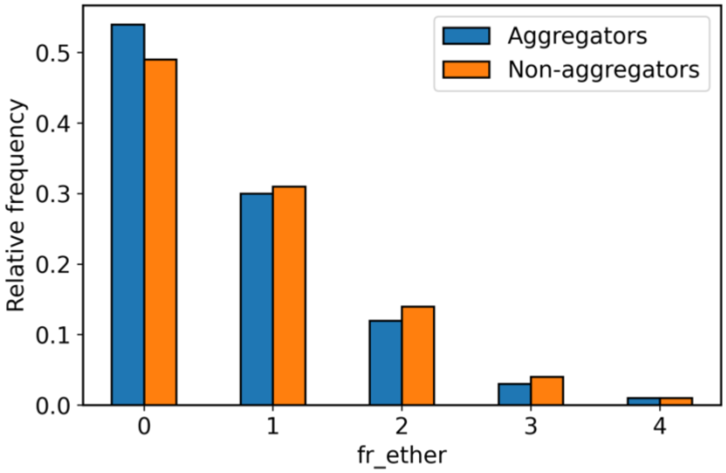
Aggregators
Non-aggregators
Relative frequency
0.0
0.1
0.2
0.3
0.4
0.5
0
1
2
3
4
fr_ether

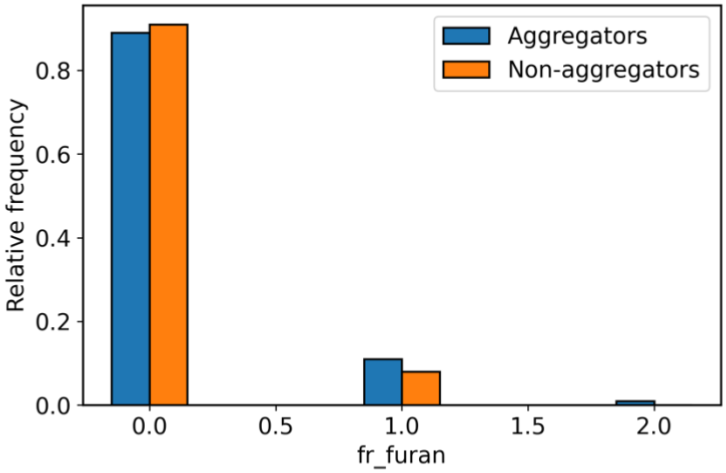
Aggregators
Non-aggregators
Relative frequency
0.0
0.2
0.4
0.6
0.8
0.0
0.5
1.0
1.5
2.0
fr_furan

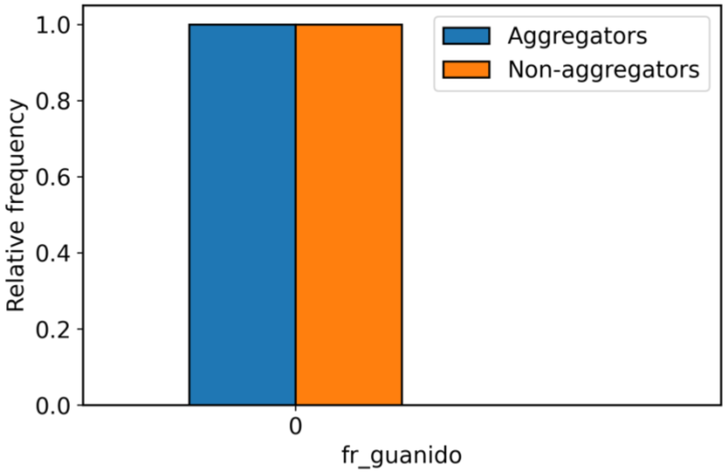
Aggregators
Non-aggregators
Relative frequency
0.0
0.2
0.4
0.6
0.8
1.0
0
fr_guanido

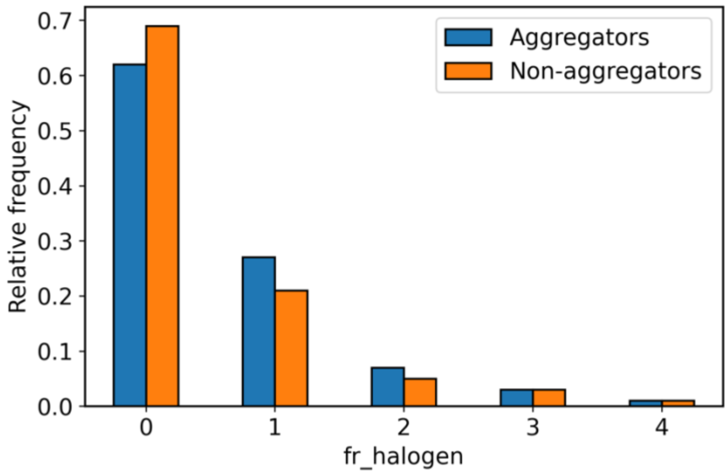

Aggregators
Non-aggregators
Relative frequency
fr_halogen

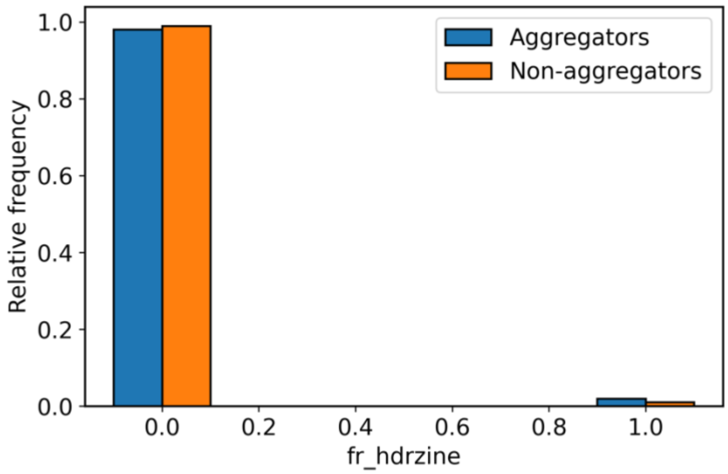

Aggregators
Non-aggregators
Relative frequency
fr_hdrzine

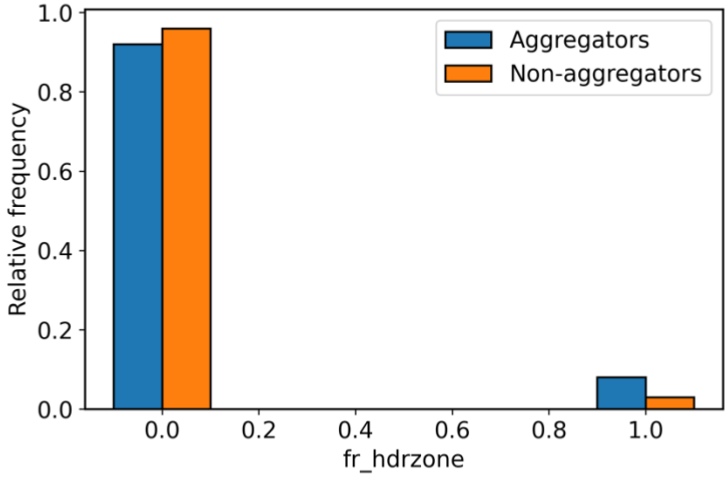

Aggregators
Non-aggregators
Relative frequency
fr_hdrzone

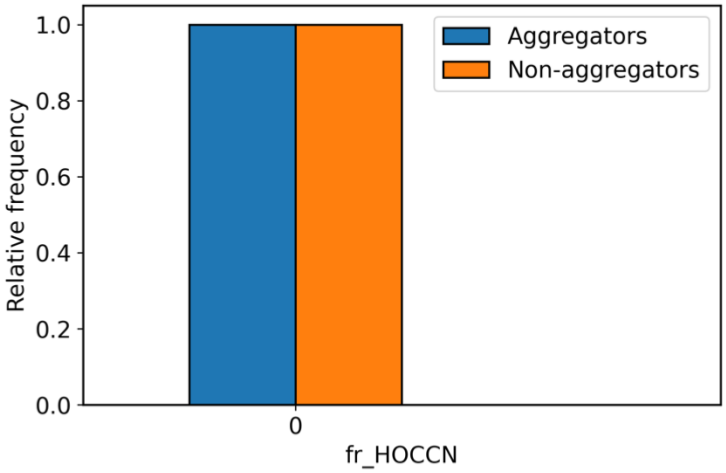
Relative frequency
fr_HOCCN
Aggregators
Non-aggregators
1.0
0.8
0.6
0.4
0.2
0.0
0

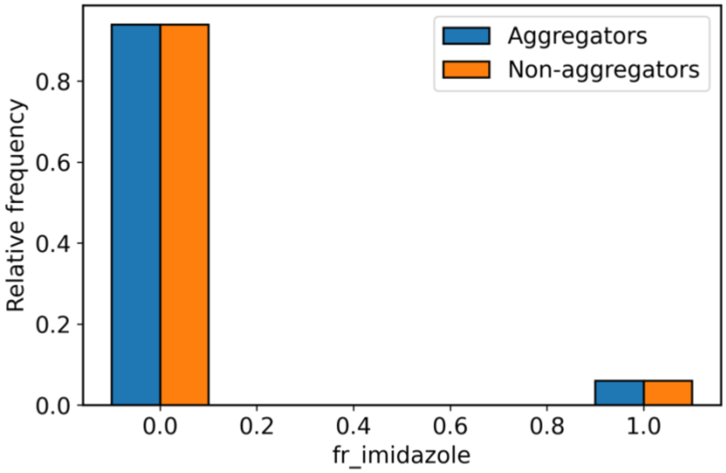
Relative frequency
fr_imidazole
Aggregators
Non-aggregators
0.8
0.6
0.4
0.2
0.0
0.0
0.2
0.4
0.6
0.8
1.0

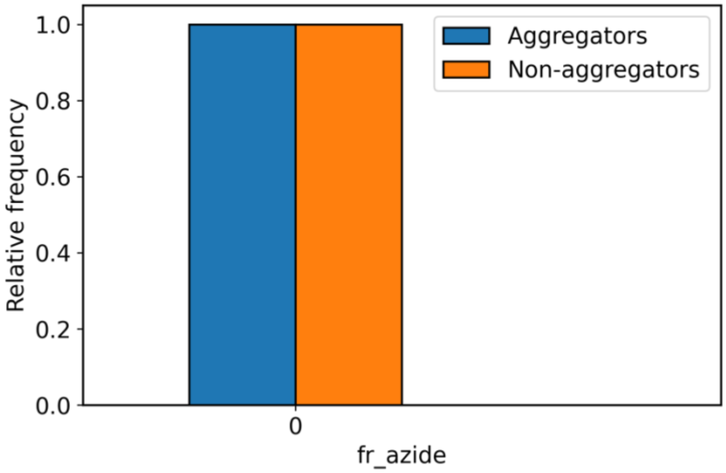
Relative frequency
fr_azide
Aggregators
Non-aggregators
1.0
0.8
0.6
0.4
0.2
0.0
0

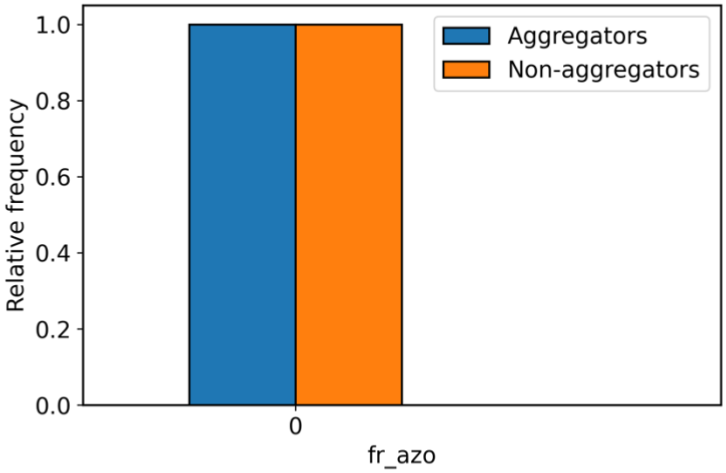
Aggregators
Non-aggregators
Relative frequency
1.0
0.8
0.6
0.4
0.2
0.0
0
fr_azo

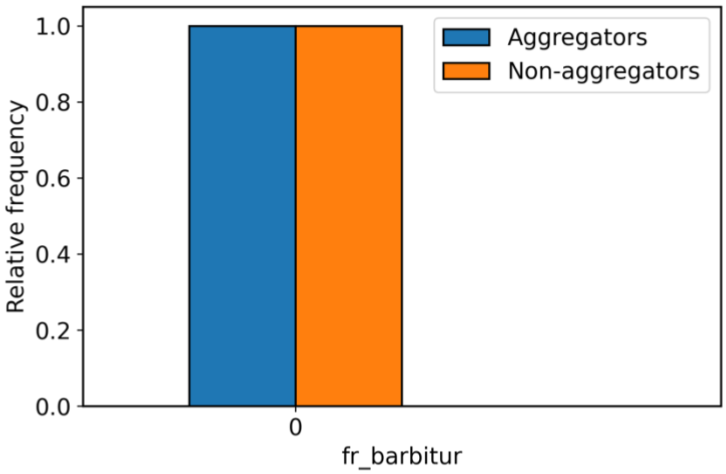
Aggregators
Non-aggregators
Relative frequency
1.0
0.8
0.6
0.4
0.2
0.0
0
fr_barbitur

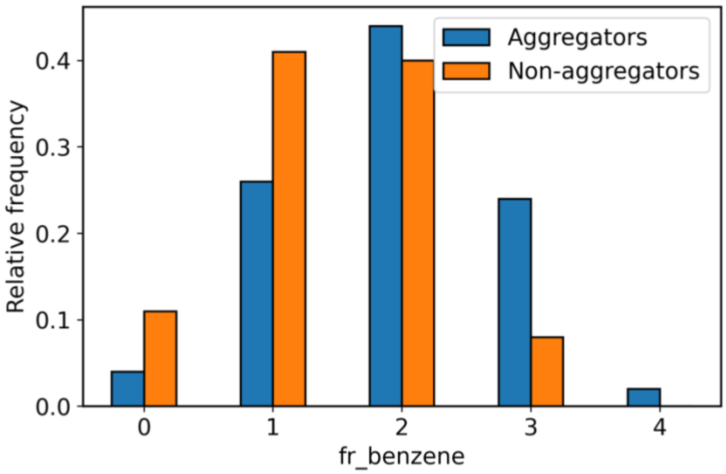
Aggregators
Non-aggregators
Relative frequency
0.4
0.3
0.2
0.1
0.0
0
1
2
3
4
fr_benzene

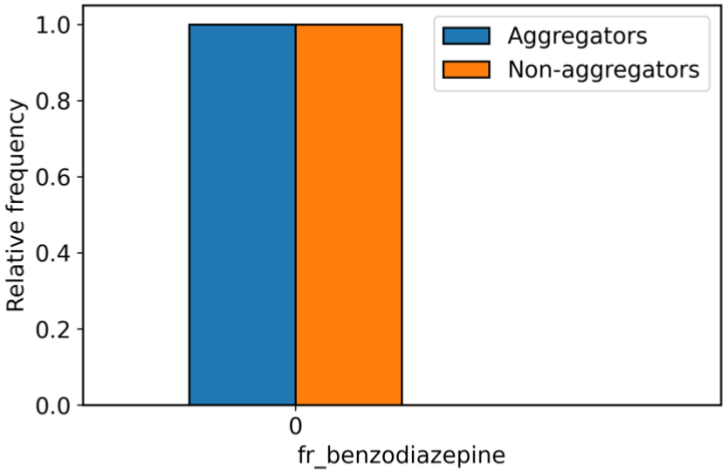
Aggregators
Non-aggregators
Relative frequency
1.0
0.8
0.6
0.4
0.2
0.0
0
fr_benzodiazepine

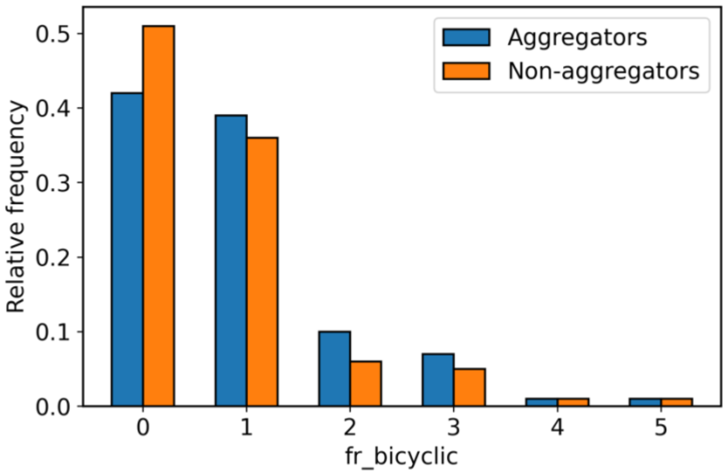
Aggregators
Non-aggregators
Relative frequency
0.5
0.4
0.3
0.2
0.1
0.0
0
1
2
3
4
5
fr_bicyclic

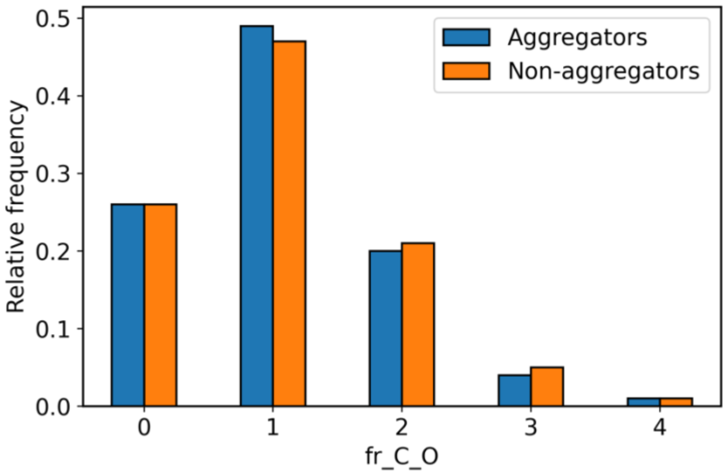
Aggregators
Non-aggregators
Relative frequency
0.5
0.4
0.3
0.2
0.1
0.0
0
1
2
3
4
fr_C_O

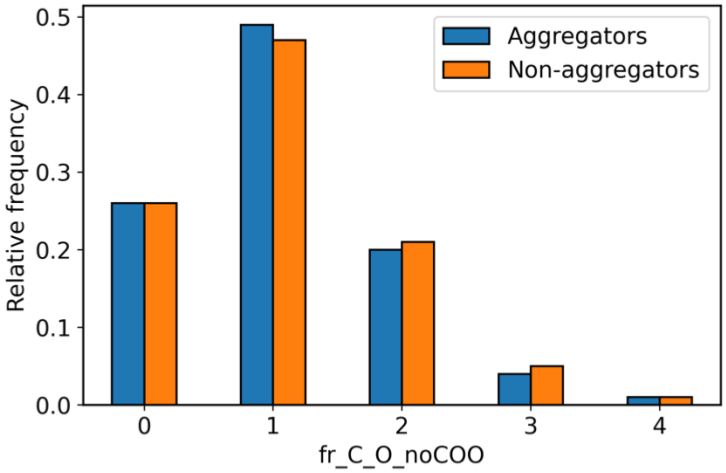
Aggregators
Non-aggregators
Relative frequency
fr_C_O_noCOO

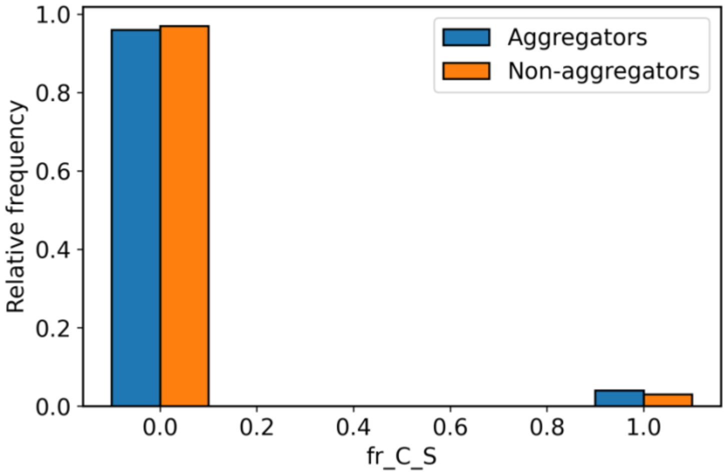
Aggregators
Non-aggregators
Relative frequency
fr_C_S

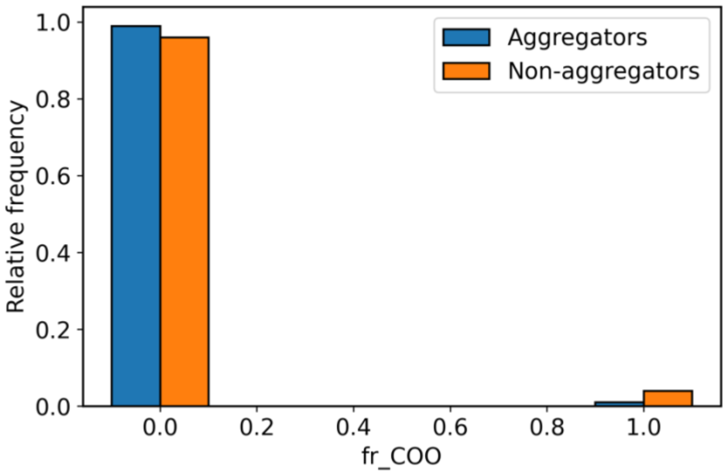
Aggregators
Non-aggregators
Relative frequency
fr_COO

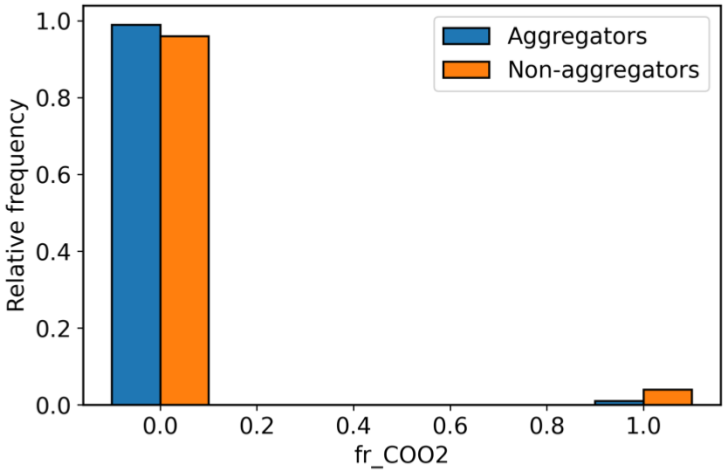
Aggregators
Non-aggregators
Relative frequency
fr_COO2
0.0
0.2
0.4
0.6
0.8
1.0

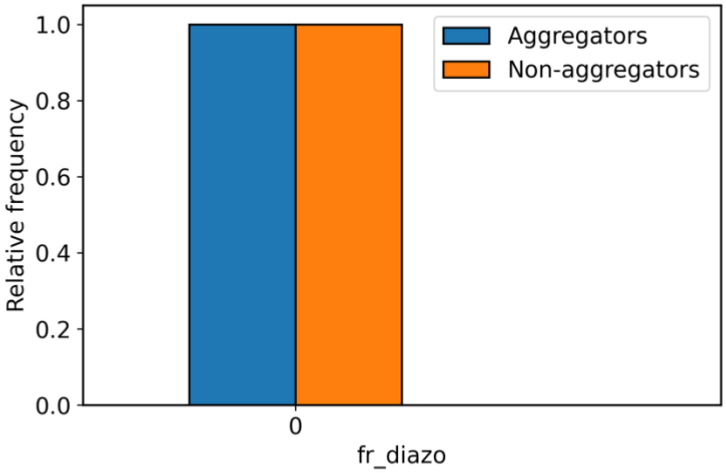
Aggregators
Non-aggregators
Relative frequency
fr_diazo
0

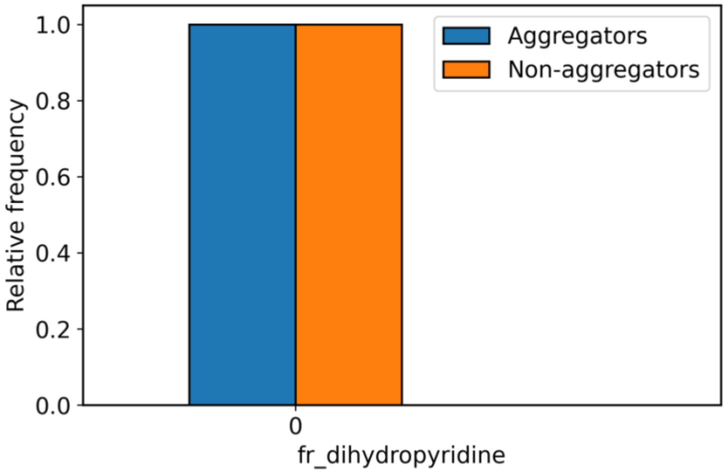
Aggregators
Non-aggregators
Relative frequency
fr_dihydropyridine
0

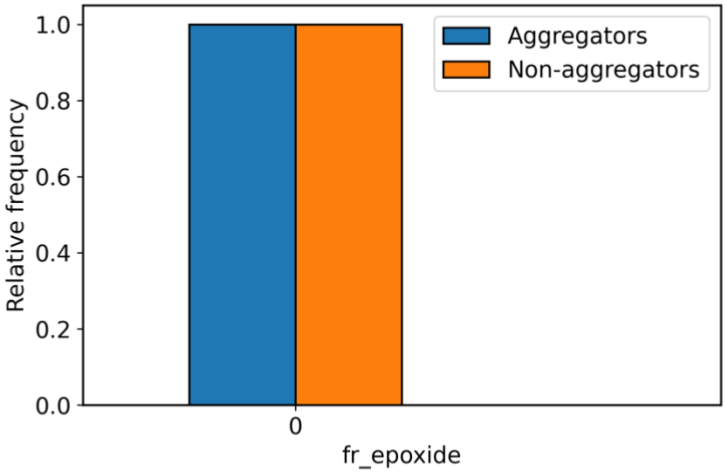
Aggregators
Non-aggregators
Relative frequency
1.0
0.8
0.6
0.4
0.2
0.0
0
fr_epoxide

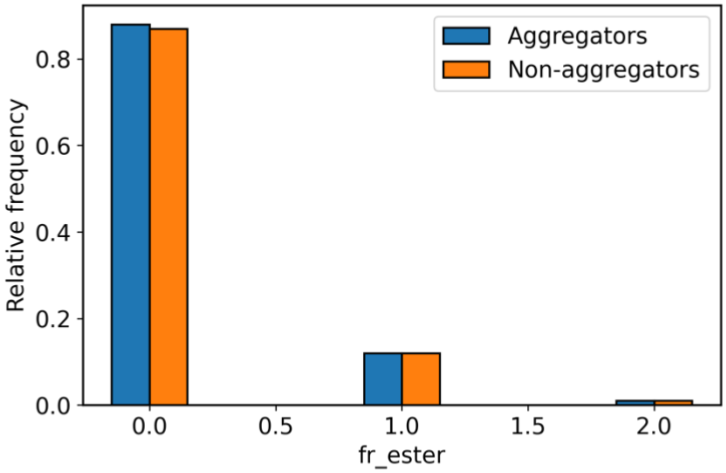
Aggregators
Non-aggregators
Relative frequency
0.8
0.6
0.4
0.2
0.0
0.0
0.5
1.0
1.5
2.0
fr_ester

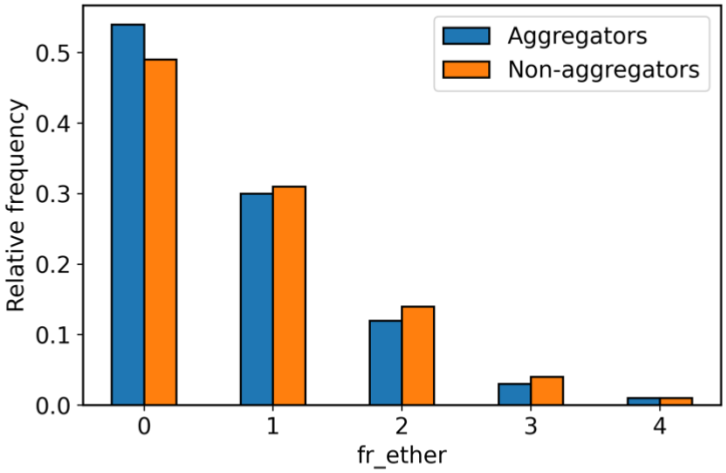
Aggregators
Non-aggregators
Relative frequency
0.5
0.4
0.3
0.2
0.1
0.0
0
1
2
3
4
fr_ether

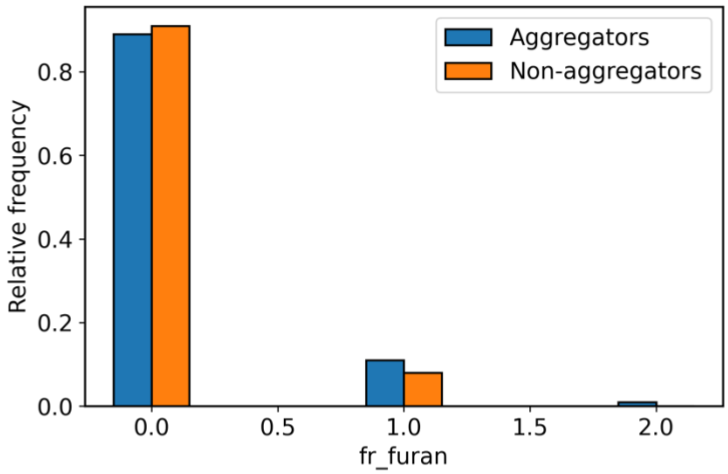
Aggregators
Non-aggregators
Relative frequency
fr_furan

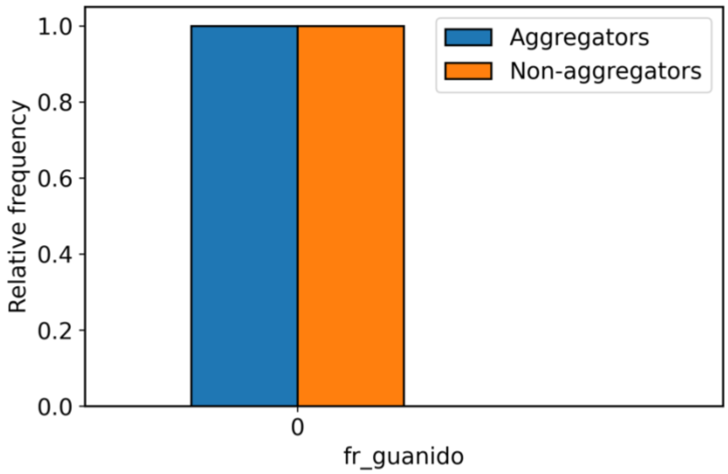
Aggregators
Non-aggregators
Relative frequency
fr_guanido

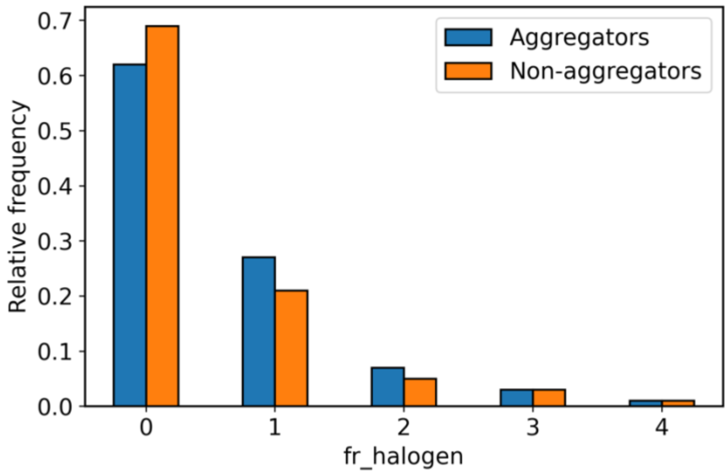
Aggregators
Non-aggregators
Relative frequency
fr_halogen

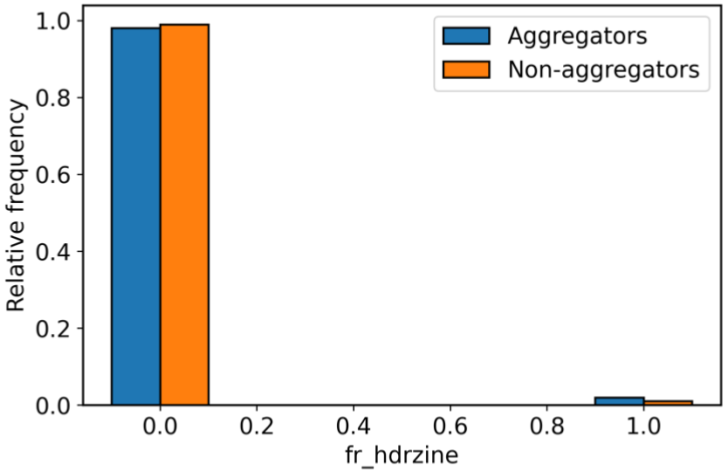

Aggregators
Non-aggregators
Relative frequency
1.0
0.8
0.6
0.4
0.2
0.0
0.0
0.2
0.4
0.6
0.8
1.0
fr_hdrzine

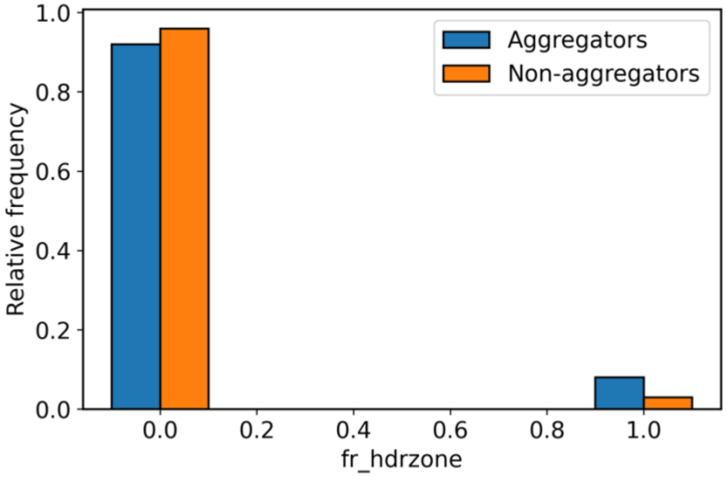

Aggregators
Non-aggregators
Relative frequency
1.0
0.8
0.6
0.4
0.2
0.0
0.0
0.2
0.4
0.6
0.8
1.0
fr_hdrzone

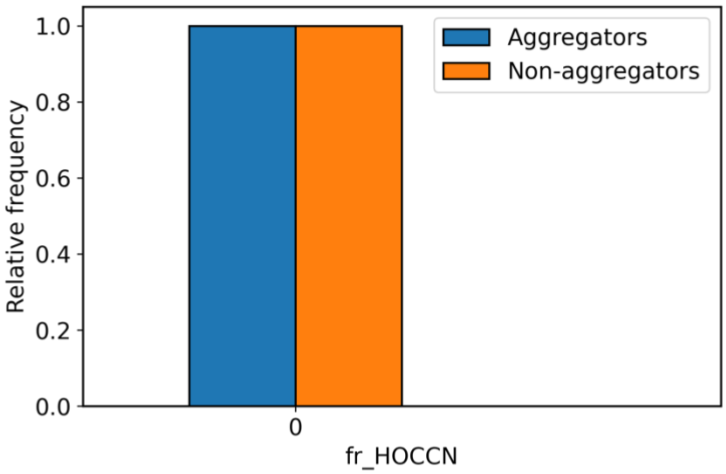

Aggregators
Non-aggregators
Relative frequency
1.0
0.8
0.6
0.4
0.2
0.0
0
fr_HOCCN

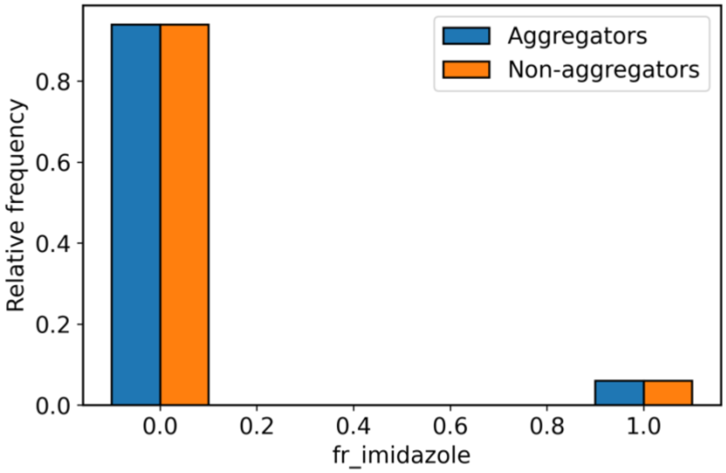

Aggregators
Non-aggregators
Relative frequency
0.0
0.2
0.4
0.6
0.8
0.0
0.2
0.4
0.6
0.8
1.0
fr_imidazole

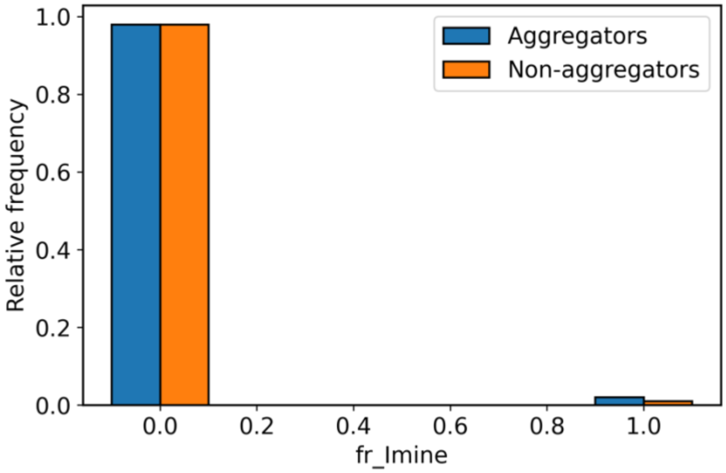

Aggregators
Non-aggregators
Relative frequency
0.0
0.2
0.4
0.6
0.8
1.0
0.0
0.2
0.4
0.6
0.8
1.0
fr_Imine

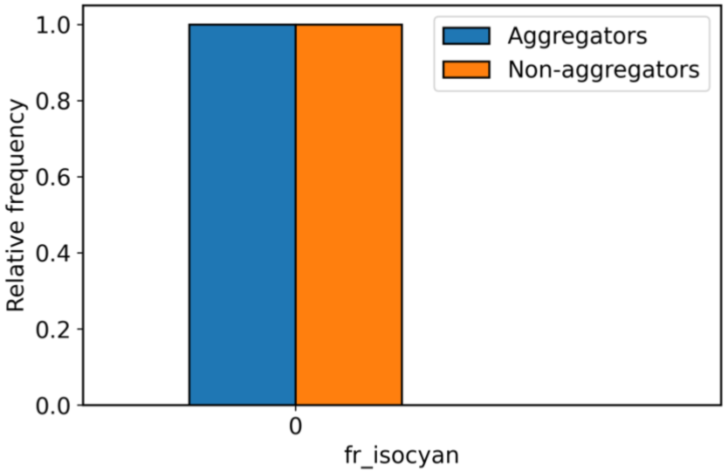

Aggregators
Non-aggregators
Relative frequency
0.0
0.2
0.4
0.6
0.8
1.0
0
fr_isocyan

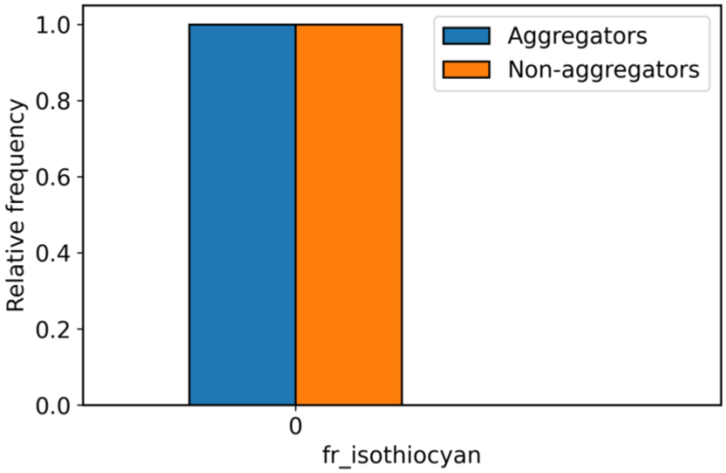
Aggregators
Non-aggregators
Relative frequency
1.0
0.8
0.6
0.4
0.2
0.0
0
fr_isothiocyan

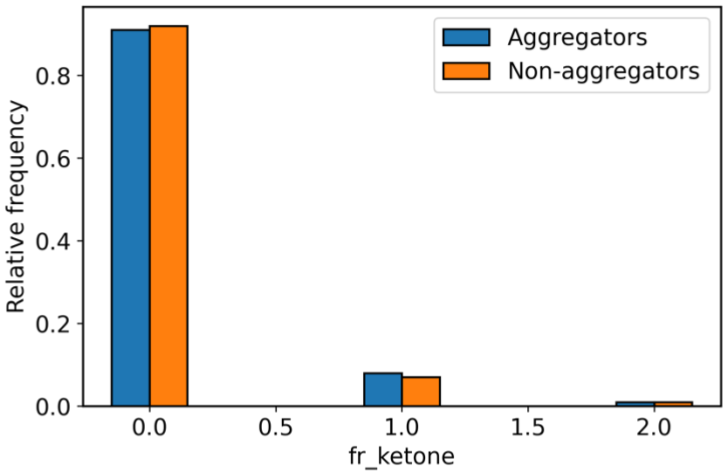
Aggregators
Non-aggregators
Relative frequency
0.8
0.6
0.4
0.2
0.0
0.0
0.5
1.0
1.5
2.0
fr_ketone

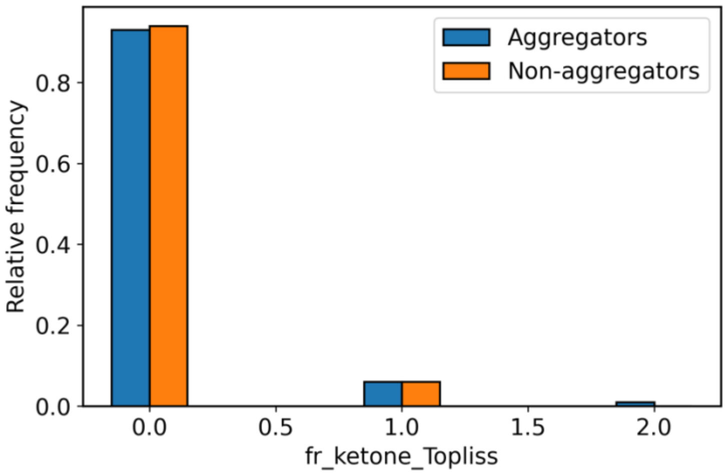
Aggregators
Non-aggregators
Relative frequency
0.8
0.6
0.4
0.2
0.0
0.0
0.5
1.0
1.5
2.0
fr_ketone_Topliss

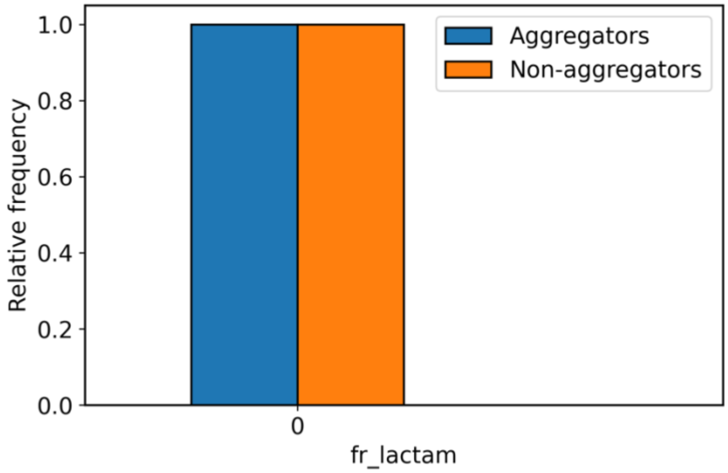
Aggregators
Non-aggregators
Relative frequency
1.0
0.8
0.6
0.4
0.2
0.0
0
fr_lactam

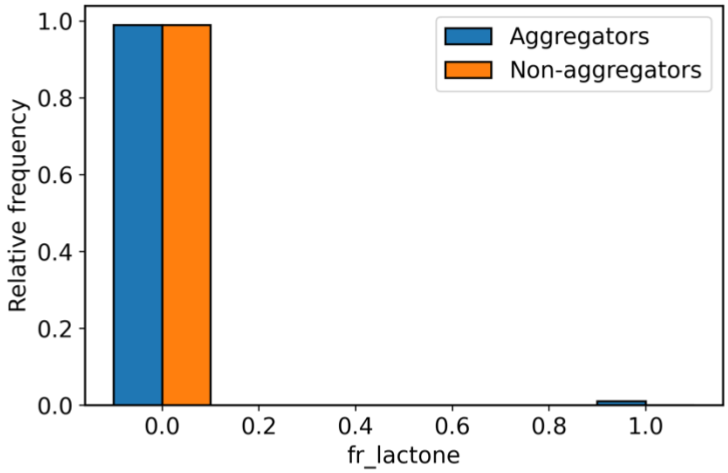
Aggregators
Non-aggregators
Relative frequency
1.0
0.8
0.6
0.4
0.2
0.0
0.0
0.2
0.4
0.6
0.8
1.0
fr_lactone

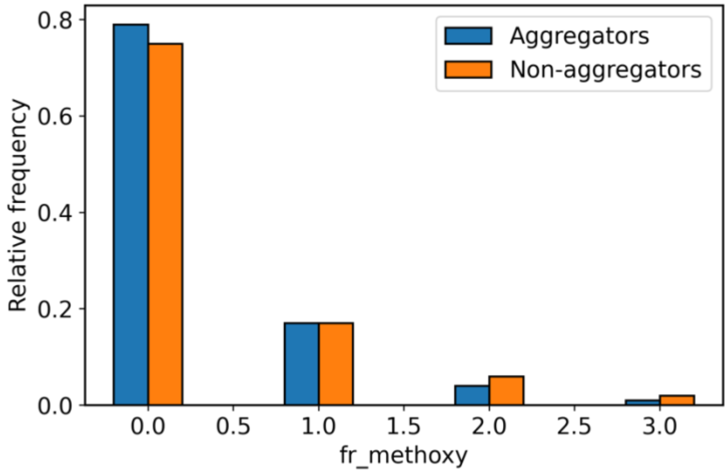
Aggregators
Non-aggregators
Relative frequency
0.8
0.6
0.4
0.2
0.0
0.0
0.5
1.0
1.5
2.0
2.5
3.0
fr_methoxy

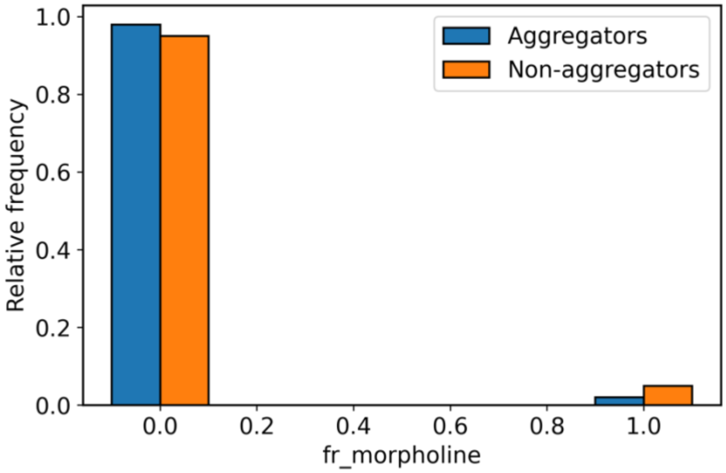
Aggregators
Non-aggregators
Relative frequency
fr_morpholine

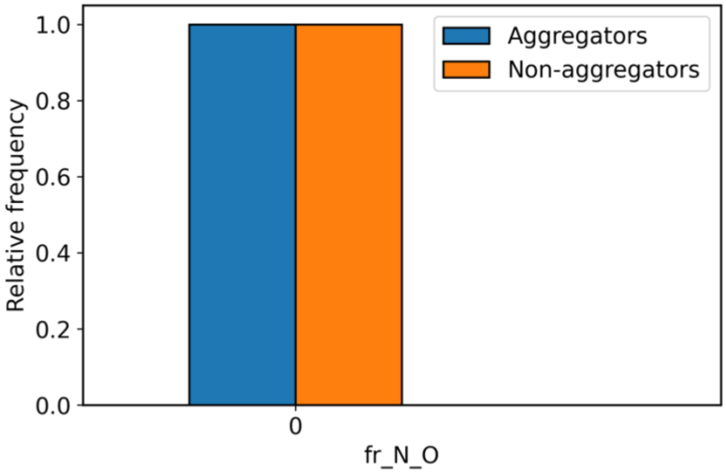
Aggregators
Non-aggregators
Relative frequency
fr_N_O

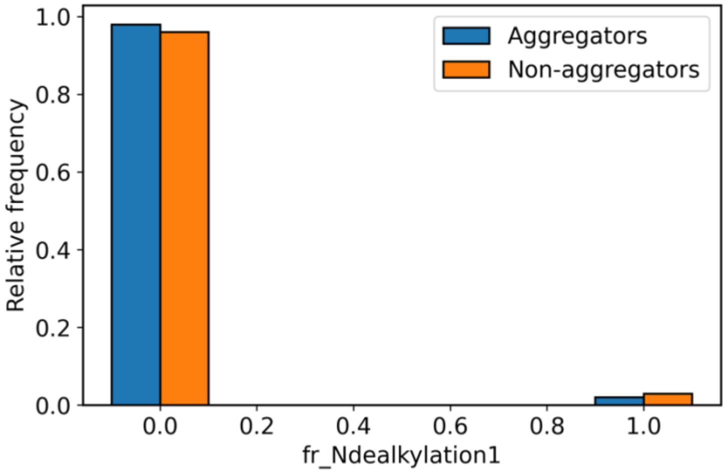
Aggregators
Non-aggregators
Relative frequency
fr_Ndealkylation1

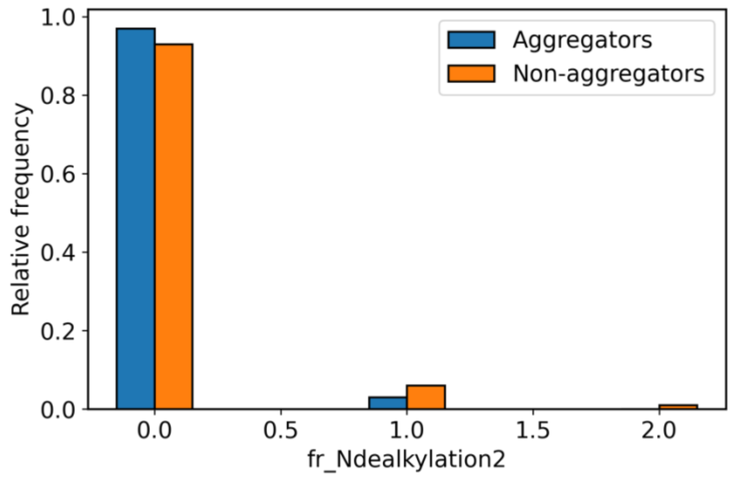
Aggregators
Non-aggregators
Relative frequency
fr_Ndealkylation2
0.0
0.2
0.4
0.6
0.8
1.0
0.5
1.5
2.0

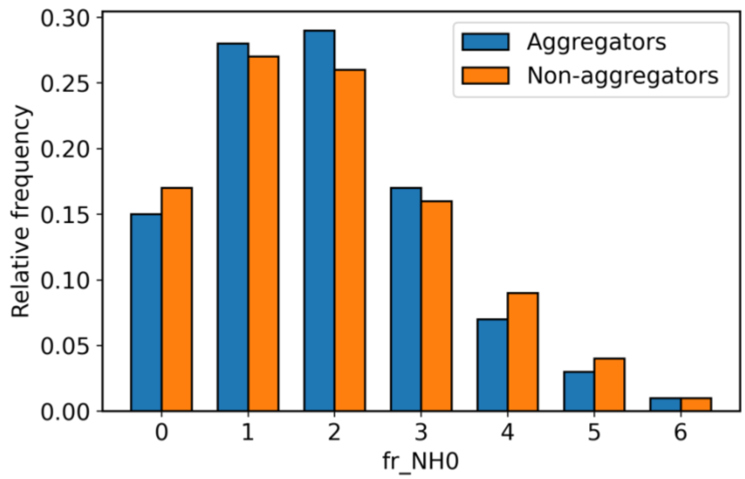
Aggregators
Non-aggregators
Relative frequency
fr_NH0
0.00
0.05
0.10
0.15
0.20
0.25
0.30
0
1
2
3
4
5
6

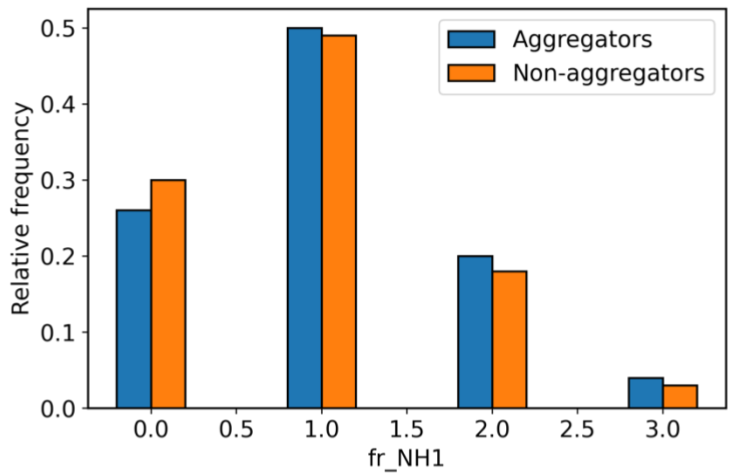
Aggregators
Non-aggregators
Relative frequency
fr_NH1
0.0
0.1
0.2
0.3
0.4
0.5
1.0
1.5
2.0
2.5
3.0

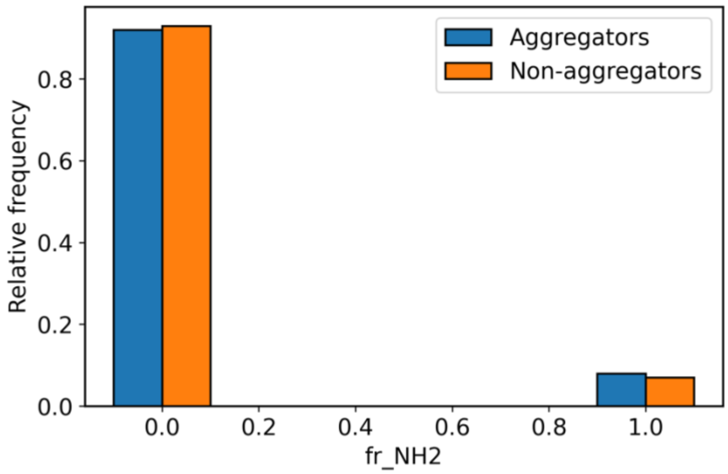
Aggregators
Non-aggregators
Relative frequency
fr_NH2
0.0
0.2
0.4
0.6
0.8
1.0

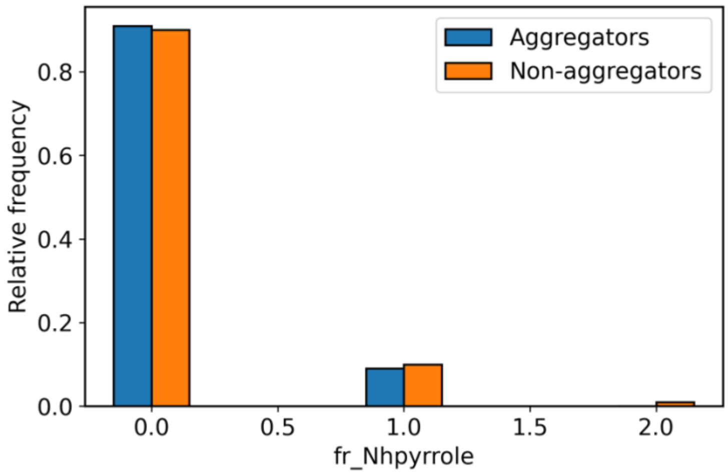
Aggregators
Non-aggregators
Relative frequency
fr_Nhpyrrole
0.0
0.2
0.4
0.6
0.8
0.5
1.0
1.5
2.0

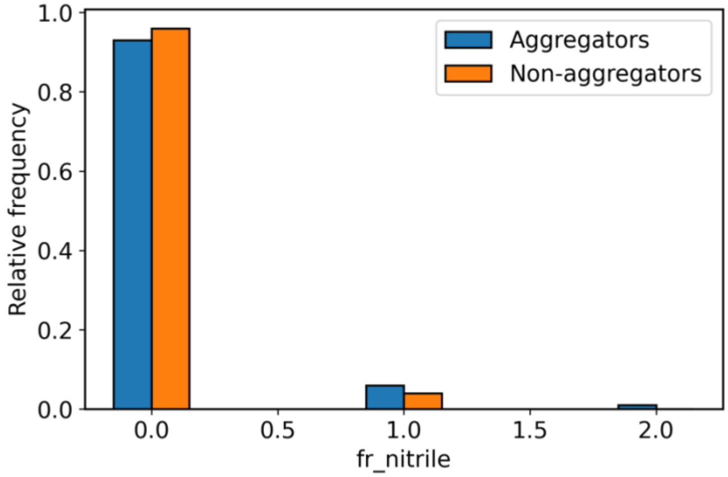
Aggregators
Non-aggregators
Relative frequency
fr_nitrile
0.0
0.2
0.4
0.6
0.8
1.0
0.5
1.5
2.0

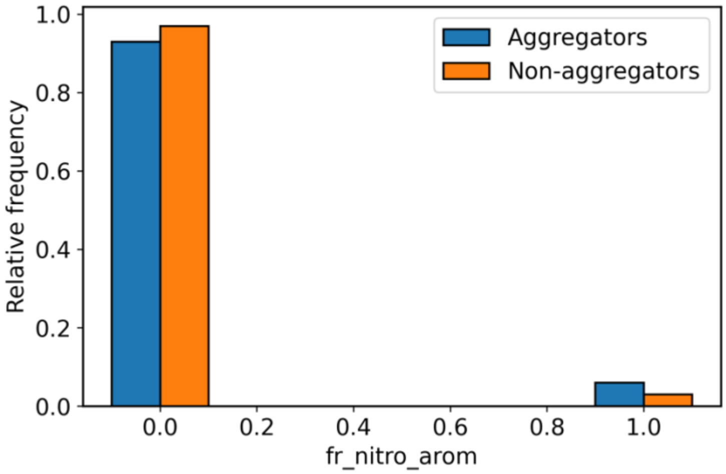
Aggregators
Non-aggregators
Relative frequency
fr_nitro_arom

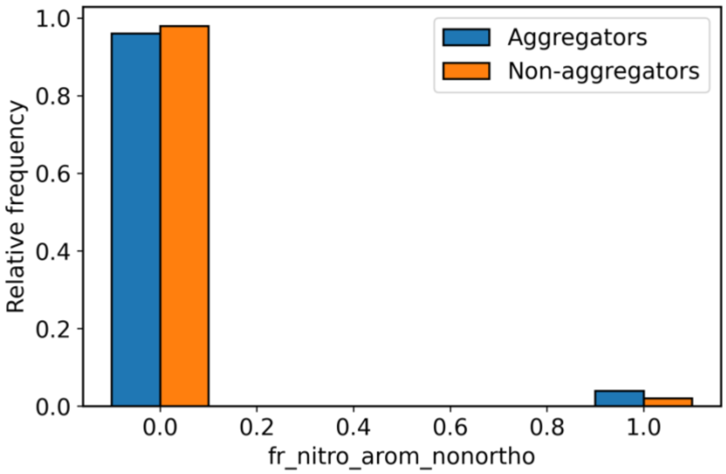
Aggregators
Non-aggregators
Relative frequency
fr_nitro_arom_nonortho

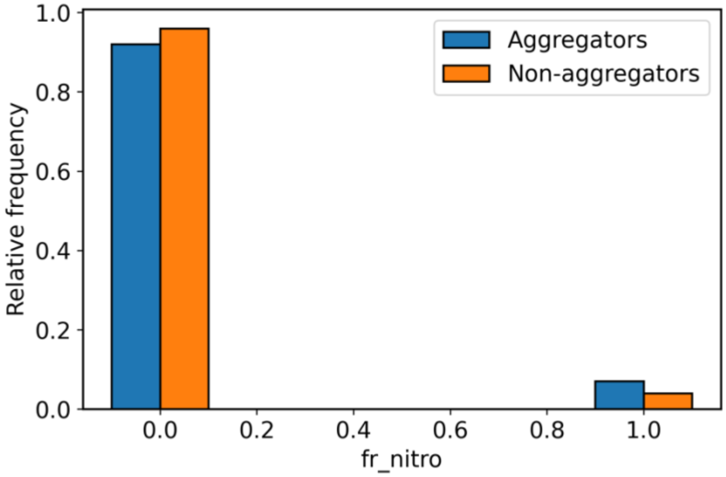
Aggregators
Non-aggregators
Relative frequency
fr_nitro

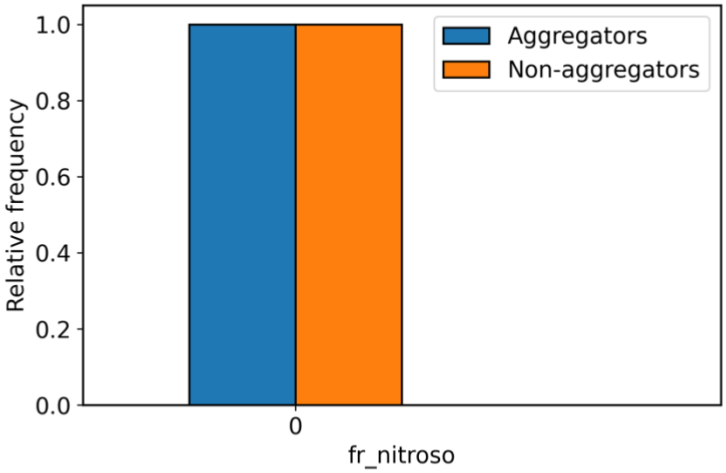
Aggregators
Non-aggregators
Relative frequency
1.0
0.8
0.6
0.4
0.2
0.0
0
fr_nitroso

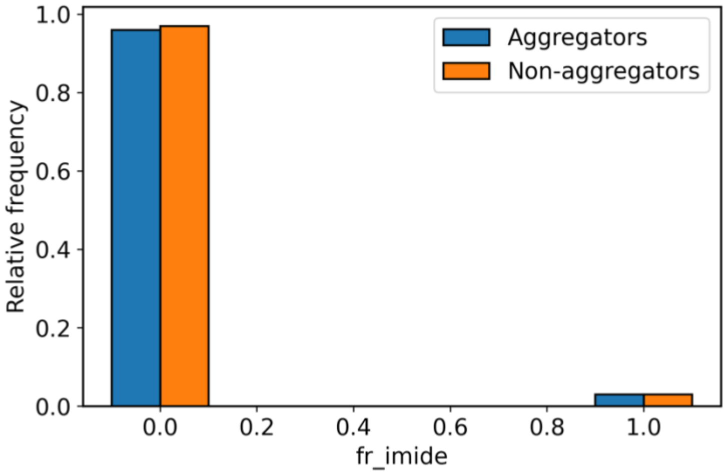
Aggregators
Non-aggregators
Relative frequency
1.0
0.8
0.6
0.4
0.2
0.0
0.0
0.2
0.4
0.6
0.8
1.0
fr_imide

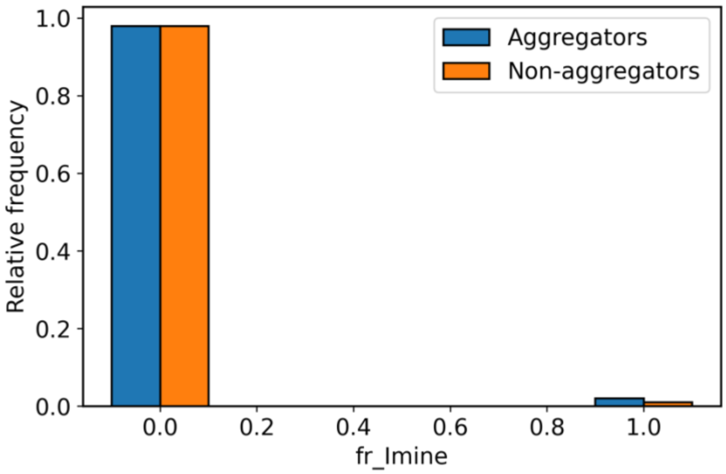
Aggregators
Non-aggregators
Relative frequency
1.0
0.8
0.6
0.4
0.2
0.0
0.0
0.2
0.4
0.6
0.8
1.0
fr_Imine

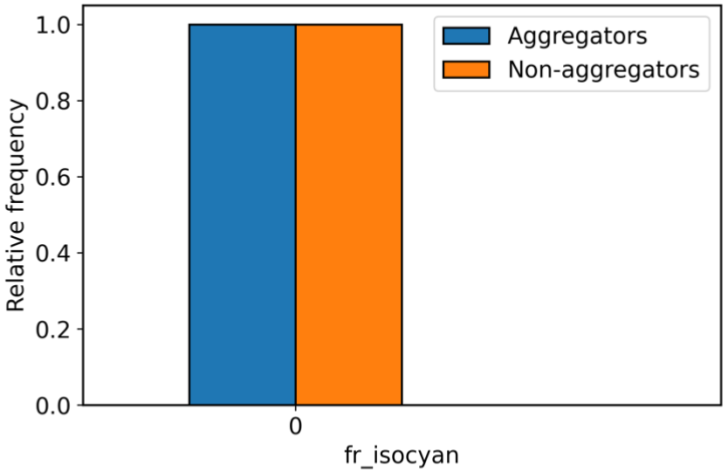
Aggregators
Non-aggregators
Relative frequency
1.0
0.8
0.6
0.4
0.2
0.0
0
fr_isocyan

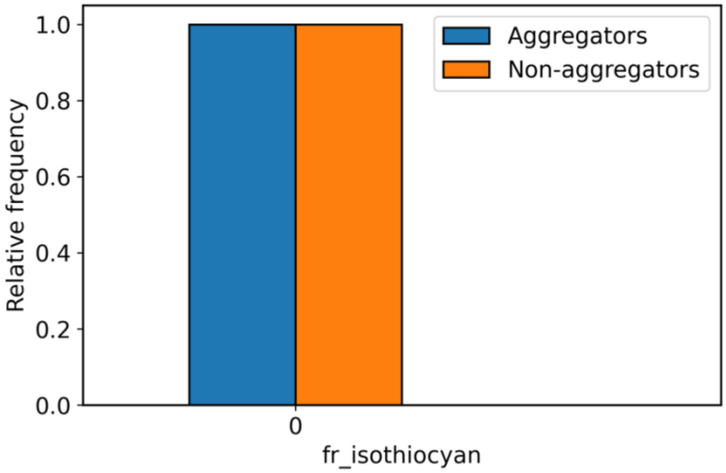
Aggregators
Non-aggregators
Relative frequency
1.0
0.8
0.6
0.4
0.2
0.0
0
fr_isothiocyan

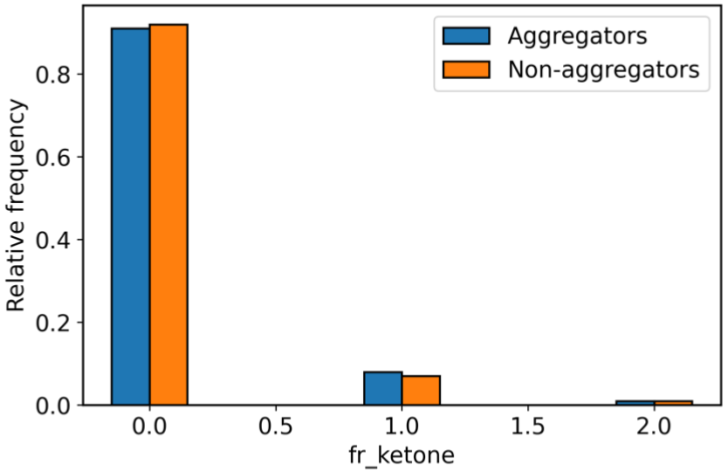
Aggregators
Non-aggregators
Relative frequency
0.8
0.6
0.4
0.2
0.0
0.0
0.5
1.0
1.5
2.0
fr_ketone

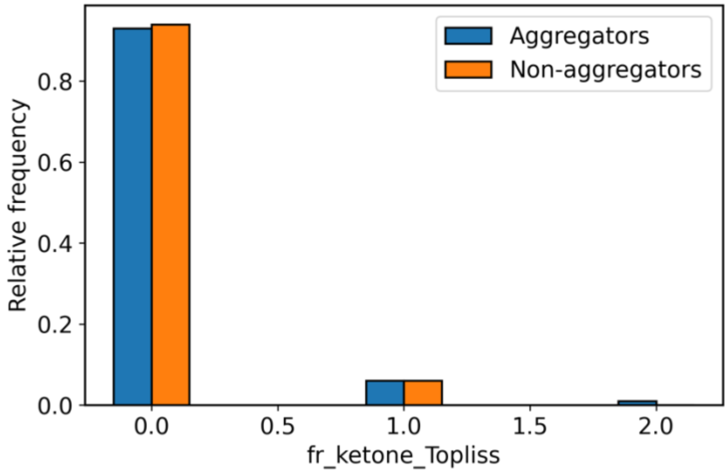
Aggregators
Non-aggregators
Relative frequency
0.0
0.2
0.4
0.6
0.8
0.0
0.5
1.0
1.5
2.0
fr_ketone_Topliss

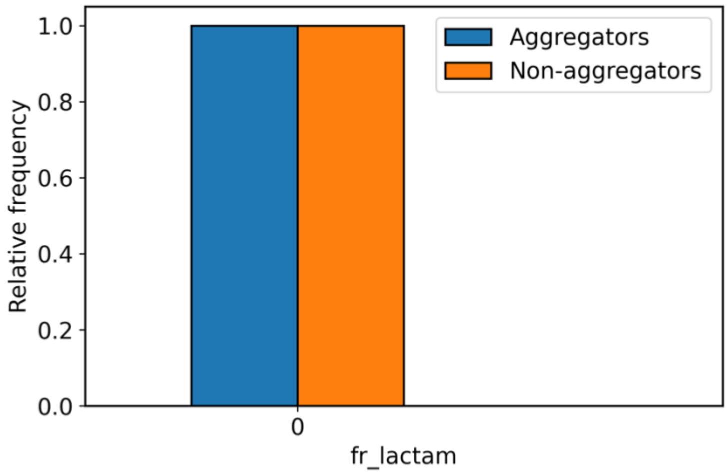
Aggregators
Non-aggregators
Relative frequency
0.0
0.2
0.4
0.6
0.8
1.0
0
fr_lactam

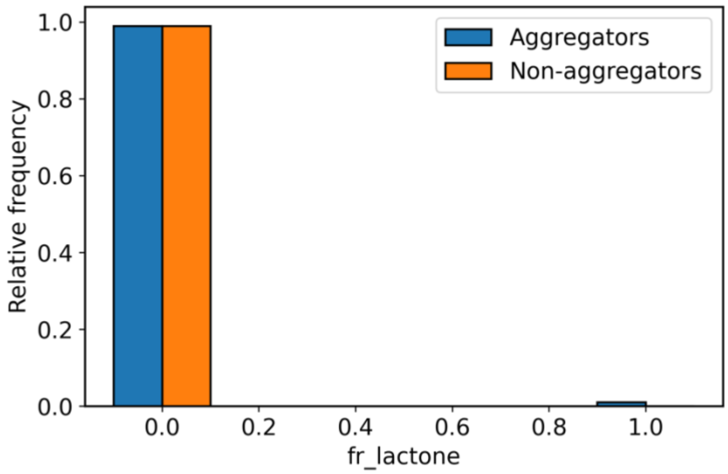
Aggregators
Non-aggregators
Relative frequency
0.0
0.2
0.4
0.6
0.8
1.0
0.0
0.2
0.4
0.6
0.8
1.0
fr_lactone

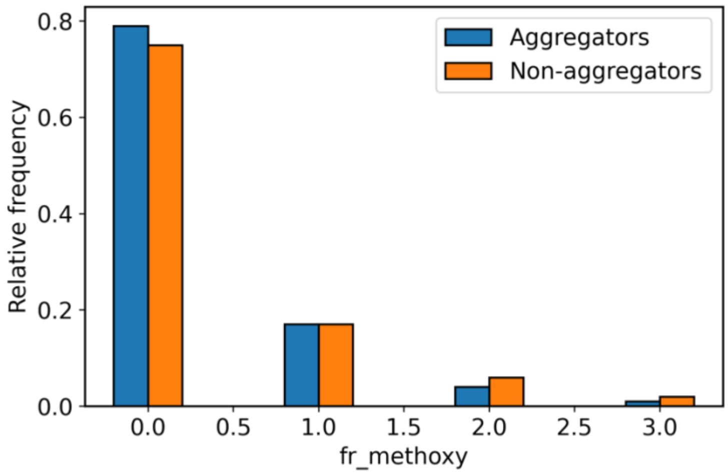
Aggregators
Non-aggregators
Relative frequency
fr_methoxy

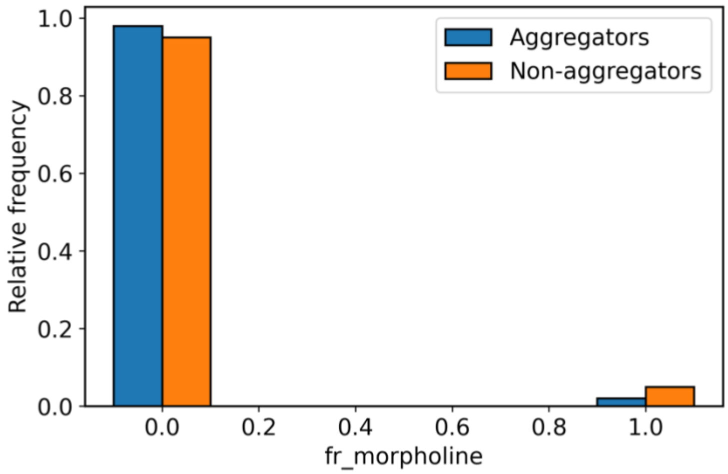
Aggregators
Non-aggregators
Relative frequency
fr_morpholine

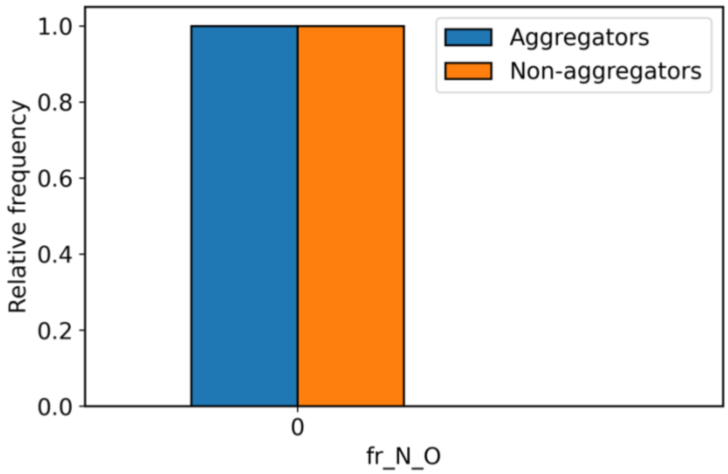
Aggregators
Non-aggregators
Relative frequency
fr_N_O

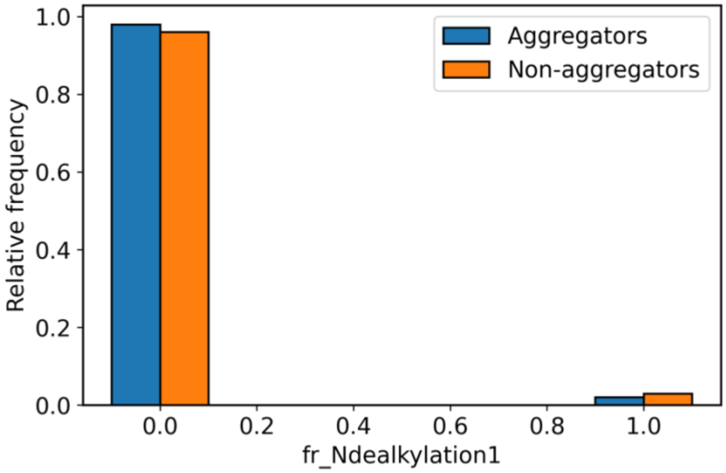

Aggregators
Non-aggregators
Relative frequency
fr_Ndealkylation1
1.0
0.8
0.6
0.4
0.2
0.0
0.0 0.2 0.4 0.6 0.8 1.0

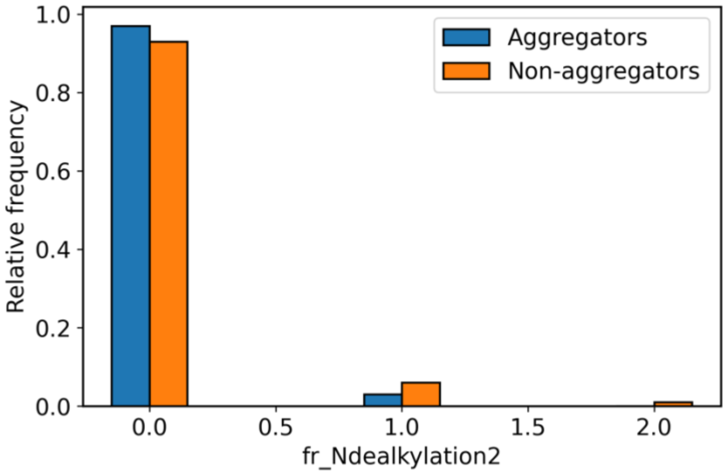

Aggregators
Non-aggregators
Relative frequency
fr_Ndealkylation2
1.0
0.8
0.6
0.4
0.2
0.0
0.0 0.5 1.0 1.5 2.0

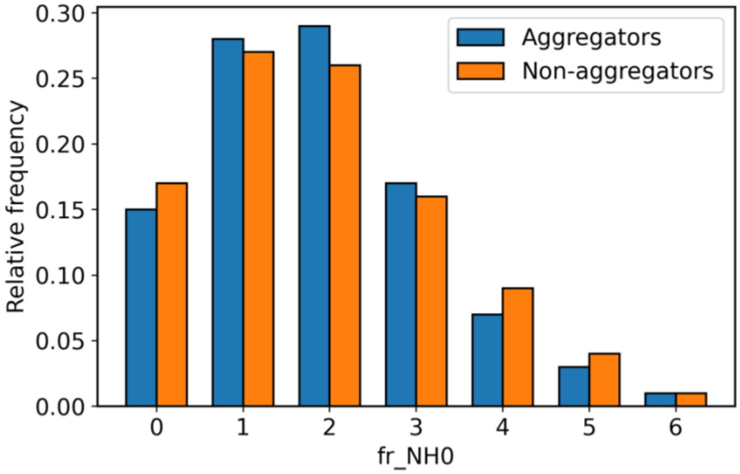

Aggregators
Non-aggregators
Relative frequency
fr_NH0
0.30
0.25
0.20
0.15
0.10
0.05
0.00
0 1 2 3 4 5 6

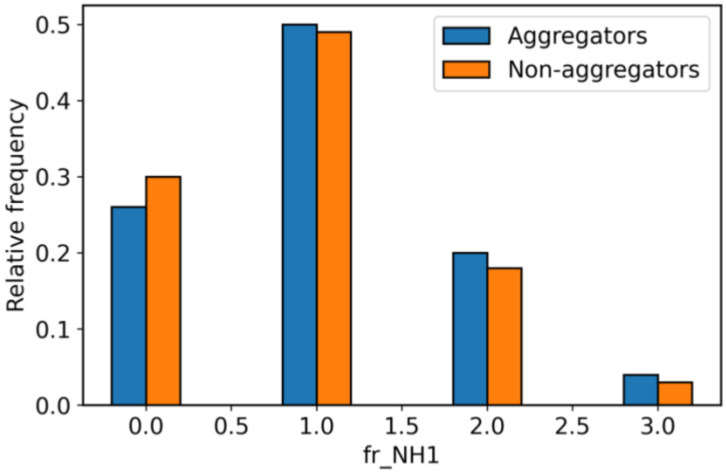
Aggregators
Non-aggregators
Relative frequency
fr_NH1

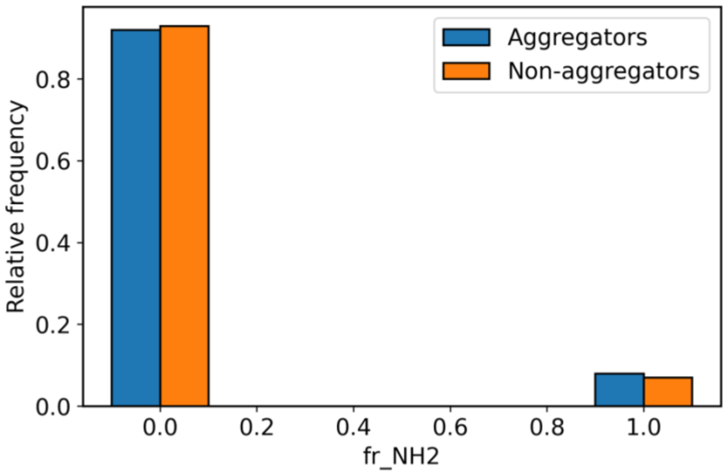
Aggregators
Non-aggregators
Relative frequency
fr_NH2

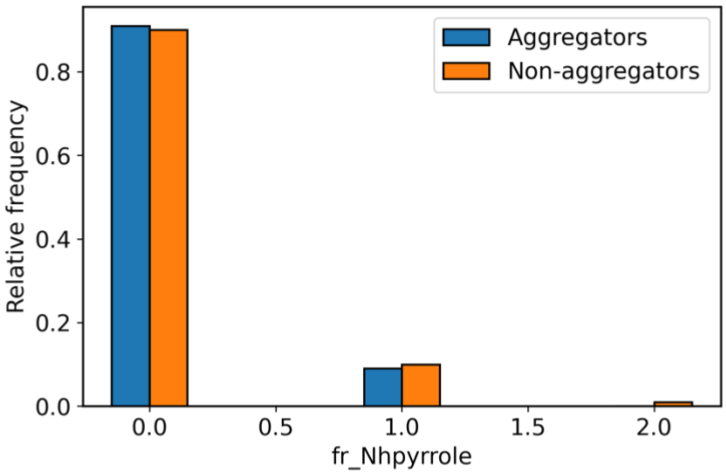
Aggregators
Non-aggregators
Relative frequency
fr_Nhpyrrole

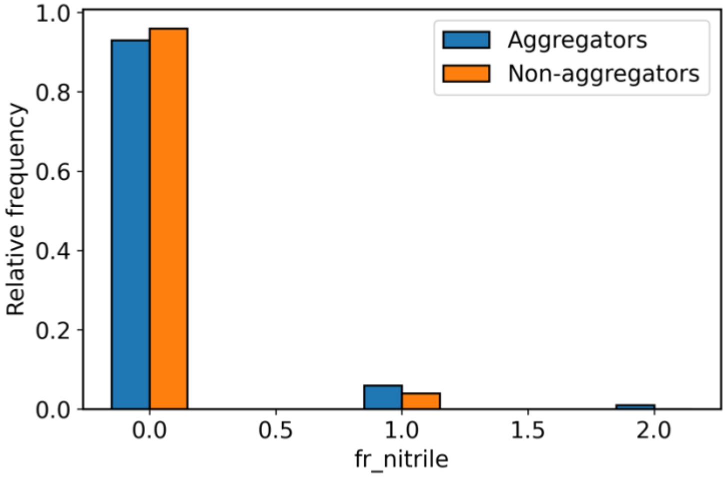
Aggregators
Non-aggregators
Relative frequency
fr_nitrile
0.0
0.2
0.4
0.6
0.8
1.0
0.0
0.5
1.0
1.5
2.0

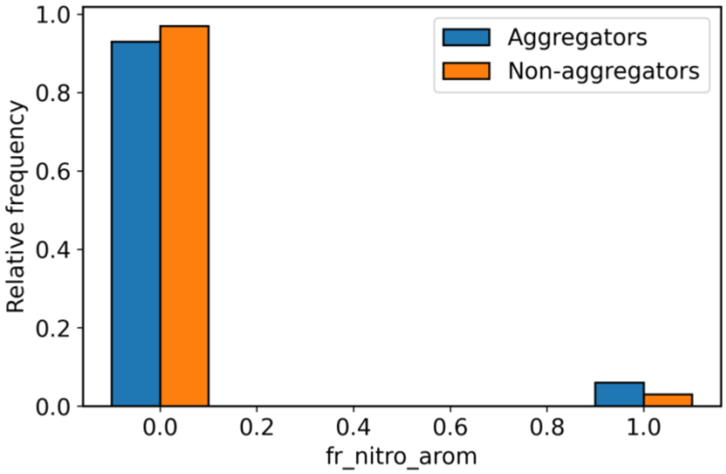
Aggregators
Non-aggregators
Relative frequency
fr_nitro_arom
0.0
0.2
0.4
0.6
0.8
1.0

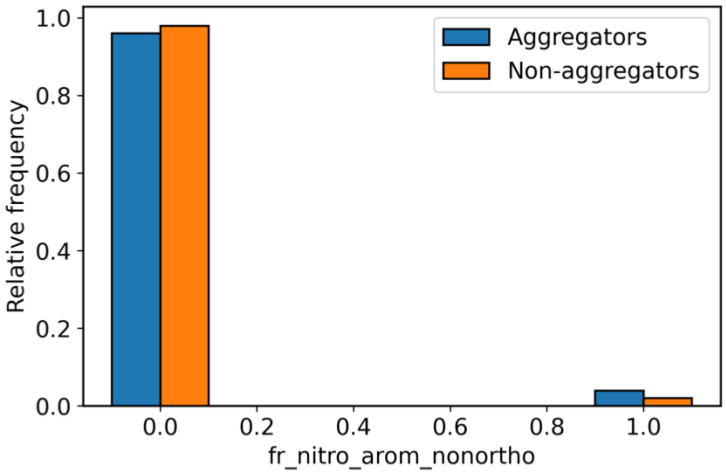
Aggregators
Non-aggregators
Relative frequency
fr_nitro_arom_nonortho
0.0
0.2
0.4
0.6
0.8
1.0

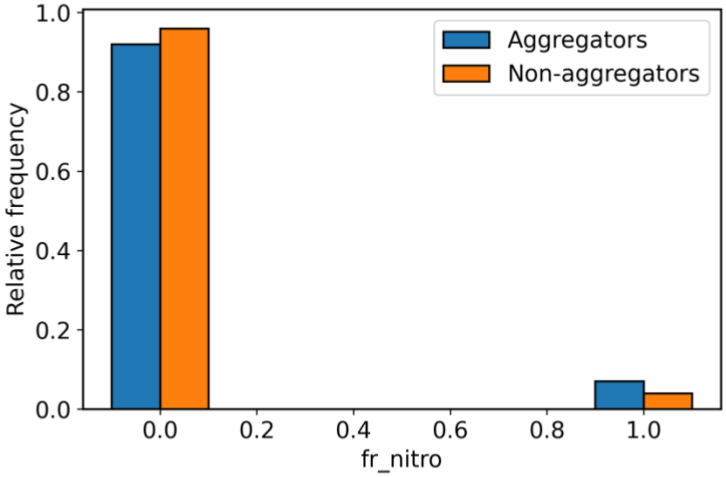

Aggregators
Non-aggregators
Relative frequency
fr_nitro

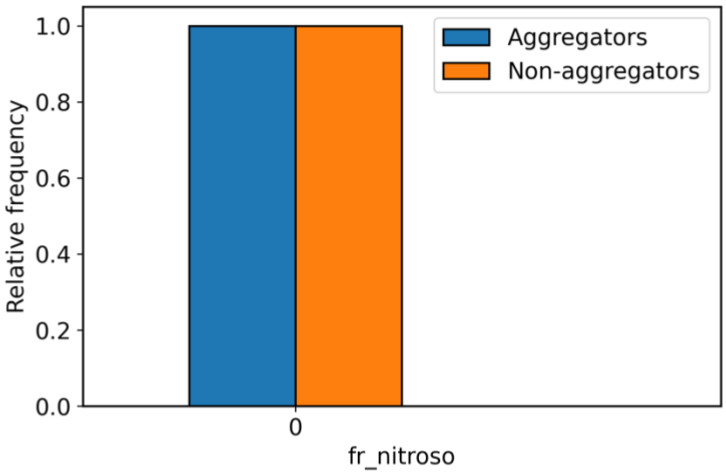

Aggregators
Non-aggregators
Relative frequency
fr_nitroso

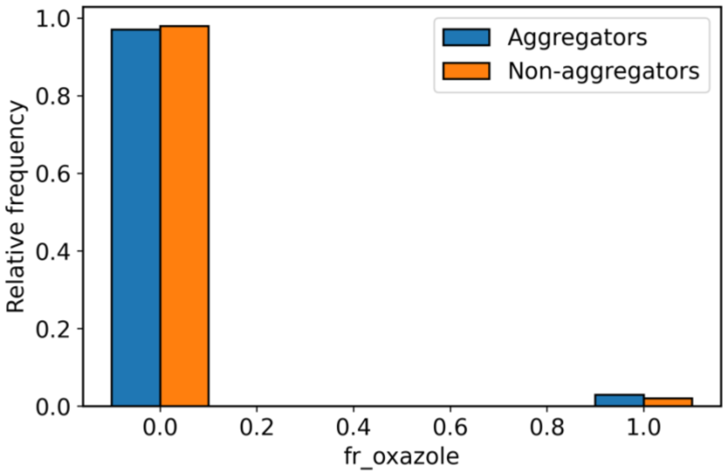

Aggregators
Non-aggregators
Relative frequency
fr_oxazole

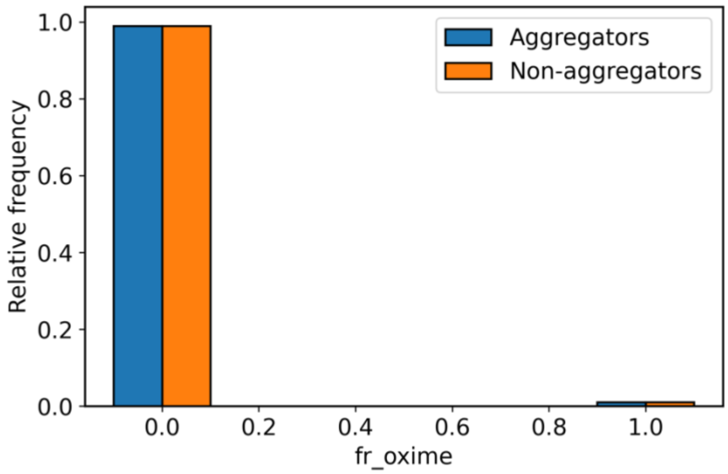
Aggregators
Non-aggregators
Relative frequency
fr_oxime
1.0
0.8
0.6
0.4
0.2
0.0
0.0
0.2
0.4
0.6
0.8
1.0

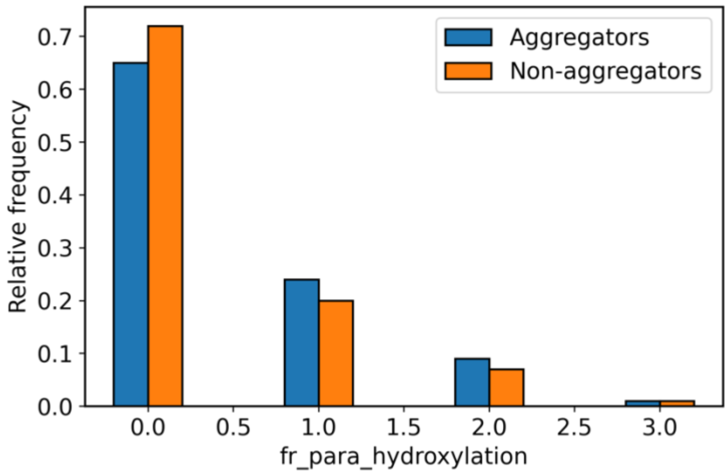
Aggregators
Non-aggregators
Relative frequency
fr_para_hydroxylation
0.7
0.6
0.5
0.4
0.3
0.2
0.1
0.0
0.0
0.5
1.0
1.5
2.0
2.5
3.0

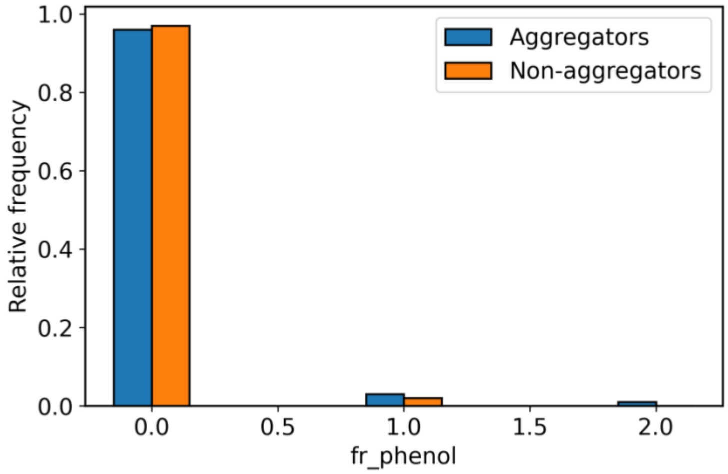
Aggregators
Non-aggregators
Relative frequency
fr_phenol
1.0
0.8
0.6
0.4
0.2
0.0
0.0
0.5
1.0
1.5
2.0

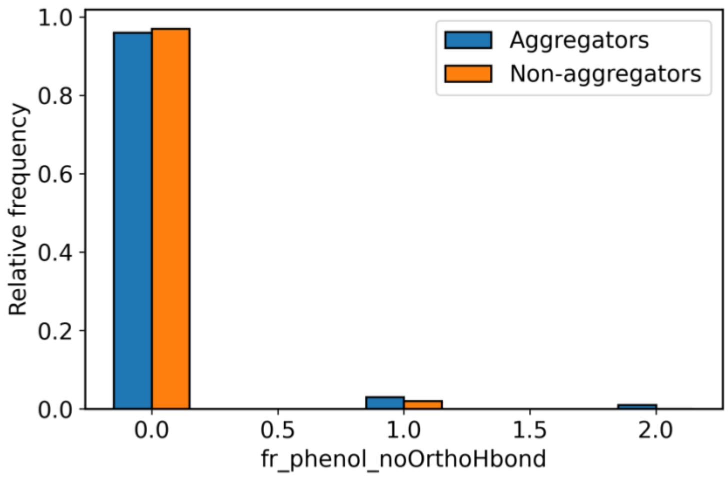

Aggregators
Non-aggregators
Relative frequency
fr_phenol_noOrthoHbond

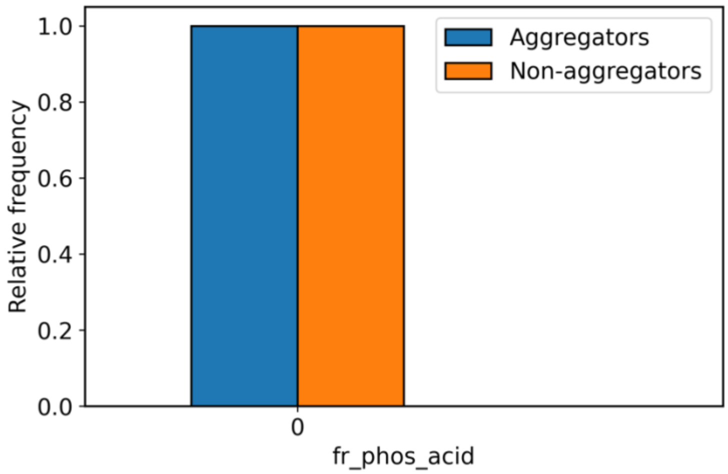

Aggregators
Non-aggregators
Relative frequency
fr_phos_acid

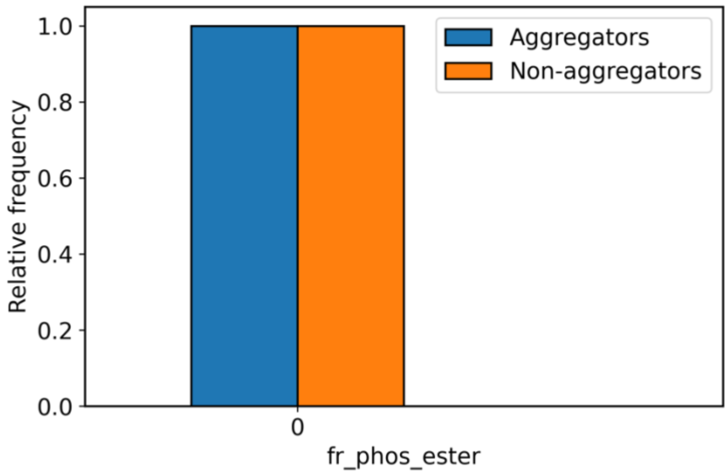

Aggregators
Non-aggregators
Relative frequency
fr_phos_ester

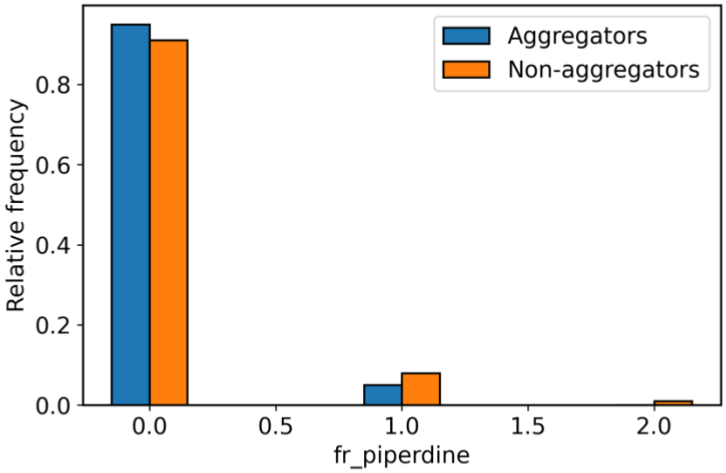
Aggregators
Non-aggregators
Relative frequency
0.0
0.2
0.4
0.6
0.8
0.0
0.5
1.0
1.5
2.0
fr_piperdine

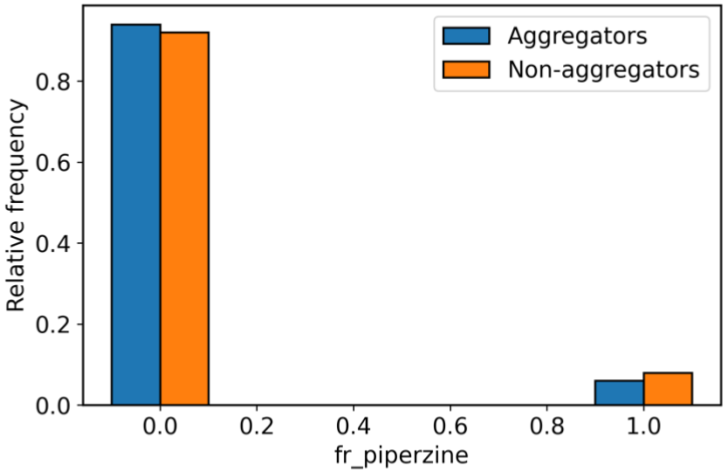
Aggregators
Non-aggregators
Relative frequency
0.0
0.2
0.4
0.6
0.8
0.0
0.2
0.4
0.6
0.8
1.0
fr_piperzine

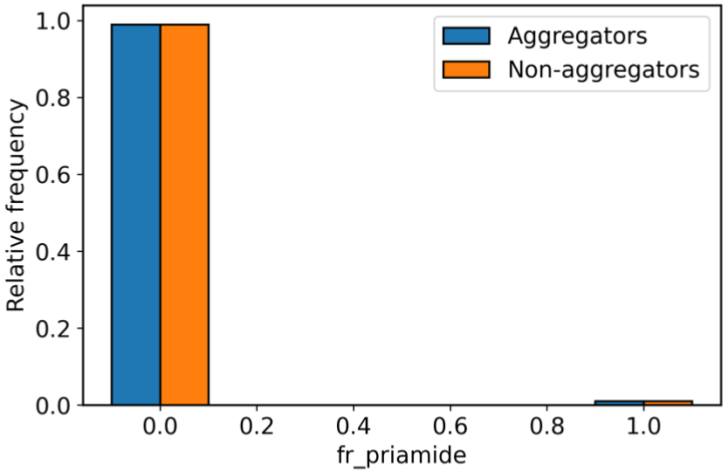
Aggregators
Non-aggregators
Relative frequency
0.0
0.2
0.4
0.6
0.8
1.0
0.0
0.2
0.4
0.6
0.8
1.0
fr_priamide

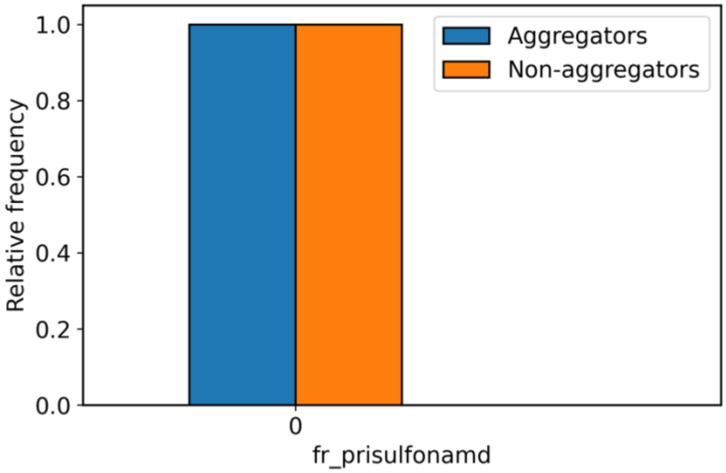

Aggregators
Non-aggregators
Relative frequency
1.0
0.8
0.6
0.4
0.2
0.0
0
fr_prisulfonamd

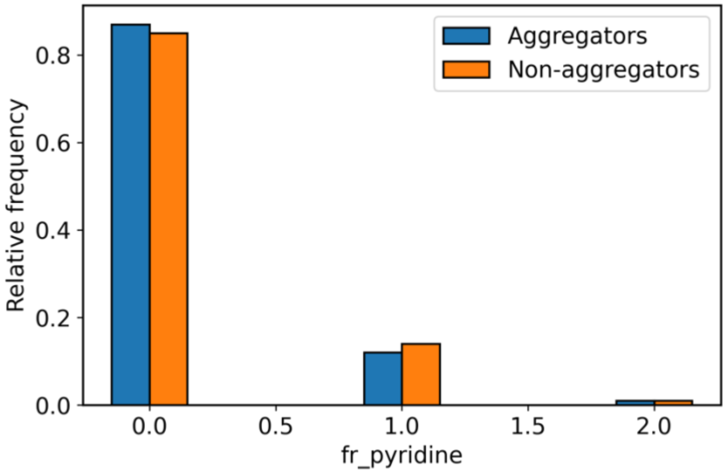

Aggregators
Non-aggregators
Relative frequency
0.8
0.6
0.4
0.2
0.0
0.0
0.5
1.0
1.5
2.0
fr_pyridine

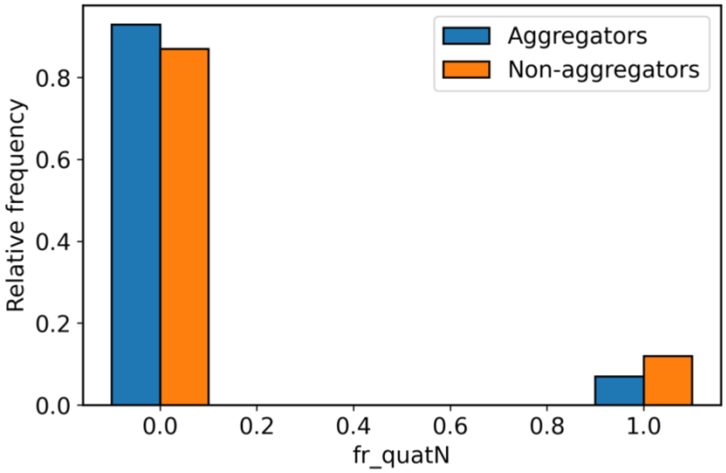

Aggregators
Non-aggregators
Relative frequency
0.8
0.6
0.4
0.2
0.0
0.0
0.2
0.4
0.6
0.8
1.0
fr_quatN

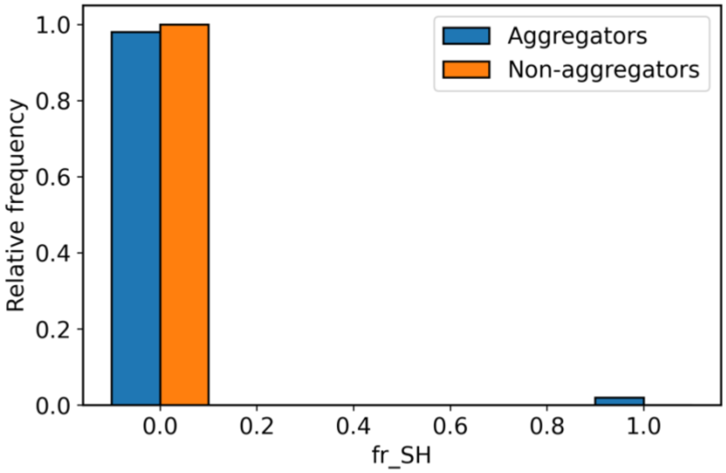

Aggregators
Non-aggregators
Relative frequency
1.0
0.8
0.6
0.4
0.2
0.0
0.0
0.2
0.4
0.6
0.8
1.0
fr_SH

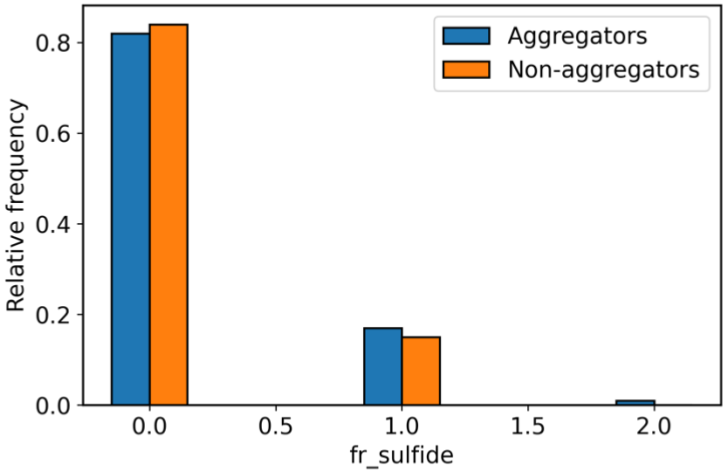

Aggregators
Non-aggregators
Relative frequency
0.8
0.6
0.4
0.2
0.0
0.0
0.5
1.0
1.5
2.0
fr_sulfide

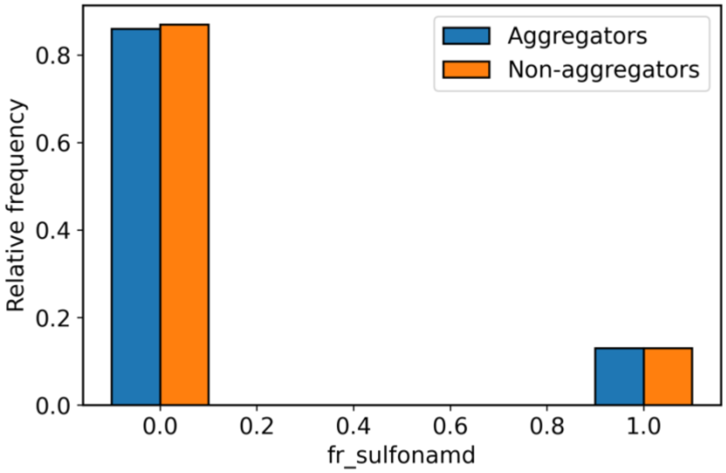

Aggregators
Non-aggregators
Relative frequency
0.8
0.6
0.4
0.2
0.0
0.0
0.2
0.4
0.6
0.8
1.0
fr_sulfonamd

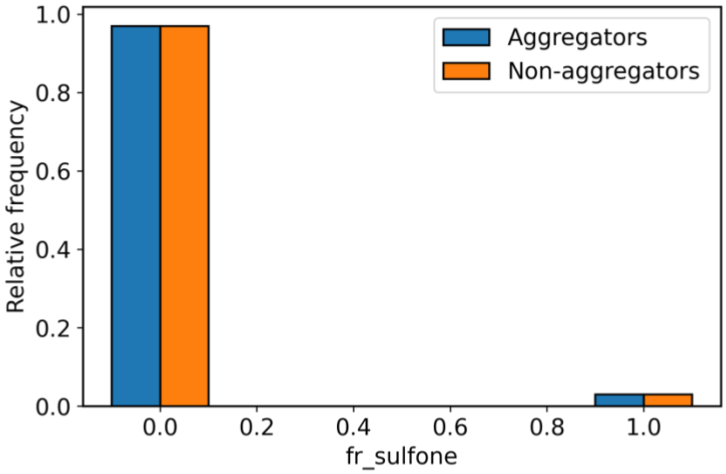

Aggregators
Non-aggregators
Relative frequency
fr_sulfone

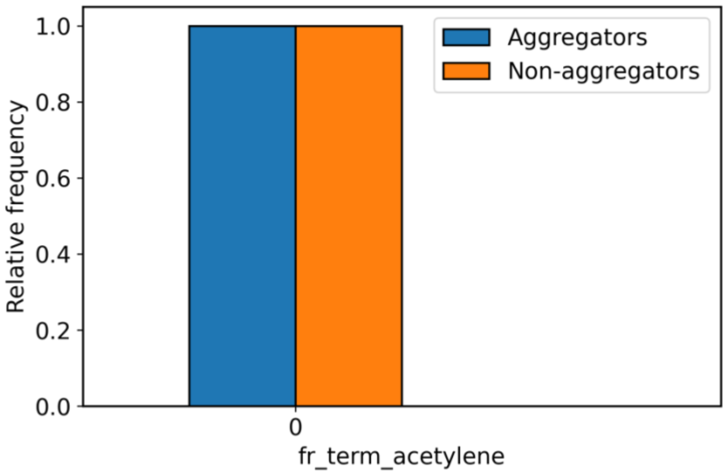

Aggregators
Non-aggregators
Relative frequency
fr_term_acetylene

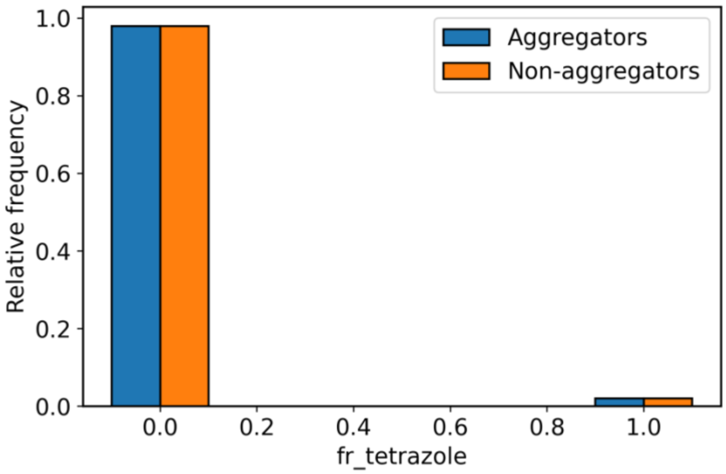

Aggregators
Non-aggregators
Relative frequency
fr_tetrazole

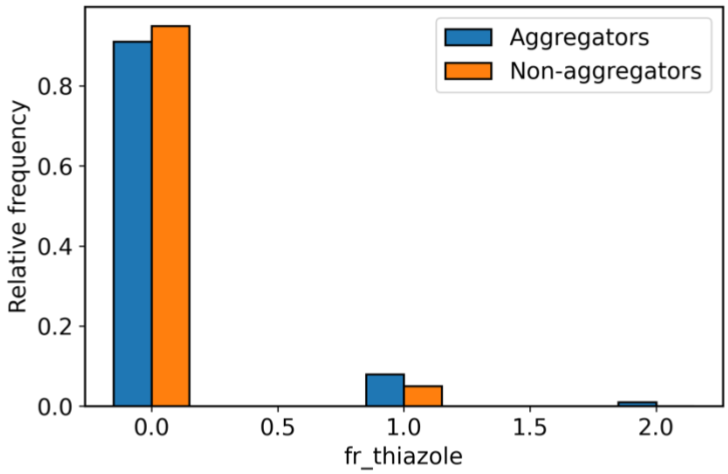
Aggregators
Non-aggregators
Relative frequency
0.0
0.2
0.4
0.6
0.8
0.0
0.5
1.0
1.5
2.0
fr_thiazole

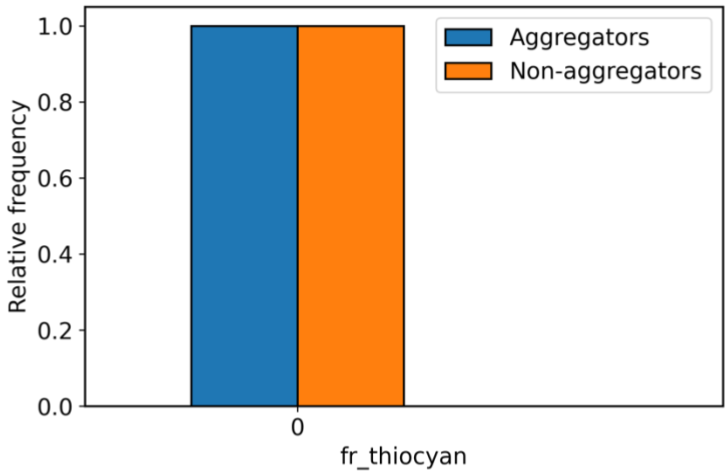
Aggregators
Non-aggregators
Relative frequency
0.0
0.2
0.4
0.6
0.8
1.0
0
fr_thiocyan

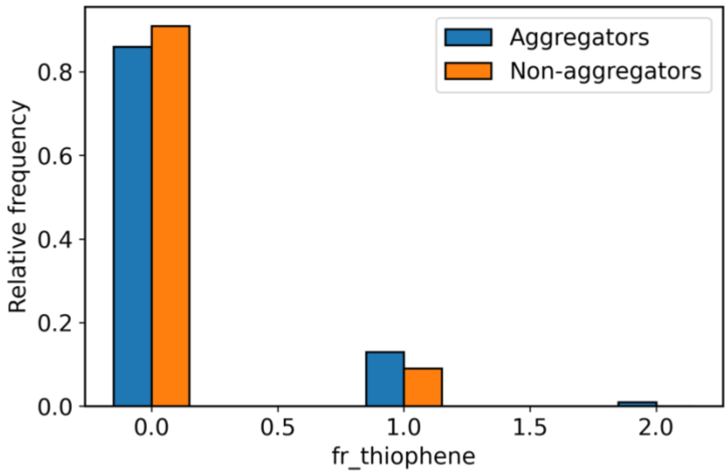
Aggregators
Non-aggregators
Relative frequency
0.0
0.2
0.4
0.6
0.8
0.0
0.5
1.0
1.5
2.0
fr_thiophene

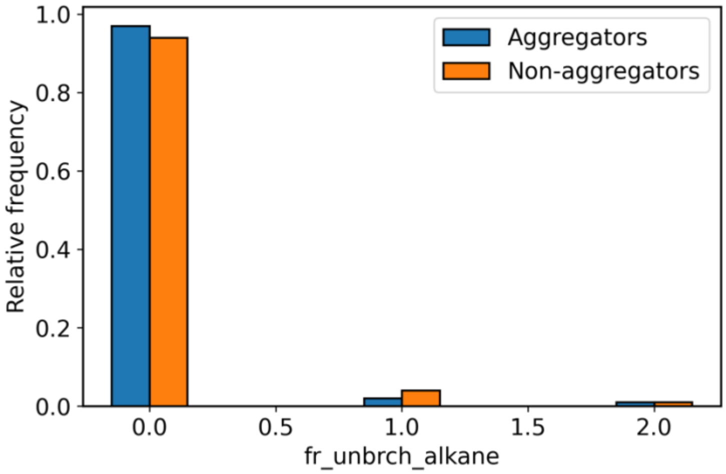
Aggregators
Non-aggregators
Relative frequency
fr_unbrch_alkane
1.0
0.8
0.6
0.4
0.2
0.0
0.0
0.5
1.0
1.5
2.0

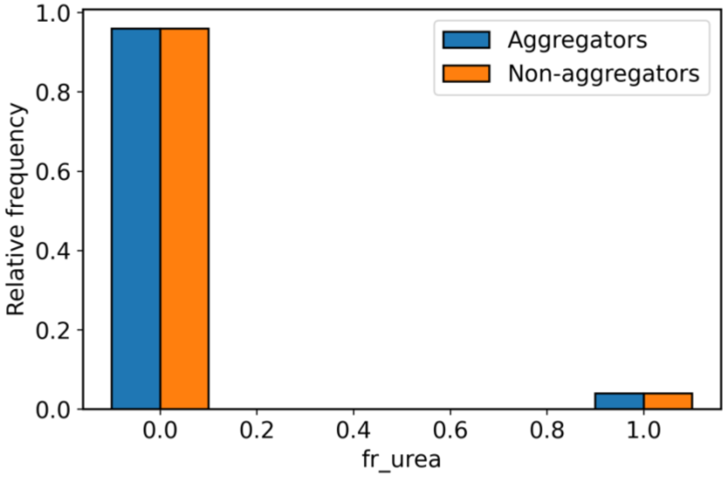
Aggregators
Non-aggregators
Relative frequency
fr_urea
1.0
0.8
0.6
0.4
0.2
0.0
0.0
0.2
0.4
0.6
0.8
1.0

SXVII. DFT energies and coordinates for pyridine derivatives E-P, monomers

Pyridine Derivative E (monomer)

HF = -362.8645756 hartrees
Zero-point correction= 0.121380 (Hartree/Particle)
Thermal correction to Gibbs Free Energy= 0.090214
Sum of electronic and zero-point Energies= -362.743195
Sum of electronic and thermal Free Energies= -362.774362

Standard orientation:

Center Number	Atomic Number	Atomic Type	Coordinates (Angstroms) X	Y	Z
1	6	0	1.074043	1.179809	-0.000217
2	6	0	1.135810	-0.202853	-0.000302
3	7	0	0.025020	-0.958332	-0.000250
4	6	0	-1.146969	-0.353964	-0.000010
5	6	0	-1.314992	1.028263	0.000198
6	6	0	-0.172323	1.797108	0.000070
7	6	0	2.441372	-0.933267	0.000202
8	8	0	-2.255757	-1.129714	0.000036
9	1	0	1.984328	1.763503	-0.000405
10	1	0	-2.305729	1.460347	0.000371
11	1	0	-0.246379	2.877455	0.000149
12	1	0	3.282285	-0.241361	-0.005866
13	1	0	2.514035	-1.580427	-0.876305
14	1	0	2.518742	-1.570020	0.883989
15	1	0	-1.978014	-2.054026	-0.000110

Pyridine Derivative F (monomer)

HF = -362.8622972 hartrees

Zero-point correction= 0.121878 (Hartree/Particle)

Thermal correction to Gibbs Free Energy= 0.091163

Sum of electronic and zero-point Energies= -362.740420

Sum of electronic and thermal Free Energies= -362.771134

Standard orientation:

Center Number	Atomic Number	Atomic Type	Coordinates (Angstroms) X	Y	Z
1	6	0	-0.535836	1.433915	0.000005
2	6	0	0.589768	0.635227	-0.000016
3	6	0	0.359069	-0.747592	-0.000032
4	7	0	-0.831818	-1.312778	-0.000018
5	6	0	-1.902224	-0.507789	0.000008
6	6	0	-1.806921	0.864458	0.000013
7	6	0	1.981712	1.185083	-0.000003
8	8	0	1.441034	-1.560332	0.000028
9	1	0	-0.420114	2.511552	0.000004
10	1	0	-2.868256	-0.998567	-0.000002
11	1	0	-2.697521	1.477406	0.000024
12	1	0	1.960944	2.274463	-0.000254
13	1	0	2.537439	0.850624	-0.878791
14	1	0	2.537252	0.851037	0.879065
15	1	0	1.131306	-2.474222	0.000013

Pyridine Derivative G (monomer)

HF = -323.5421013 hartrees

Zero-point correction= 0.094012 (Hartree/Particle)

Thermal correction to Gibbs Free Energy= 0.065222

Sum of electronic and zero-point Energies= -323.448089

Sum of electronic and thermal Free Energies= -323.476880

Standard orientation:

Center Number	Atomic Number	Atomic Type	Coordinates (Angstroms) X	Y	Z
1	6	0	1.187548	1.151260	-0.000010
2	6	0	-0.187921	1.222827	0.000011
3	6	0	-0.897108	0.023041	0.000074
4	7	0	-0.331427	-1.170086	0.000023
5	6	0	1.006844	-1.216660	-0.000025
6	6	0	1.807559	-0.095558	-0.000007
7	8	0	-2.247495	0.083992	-0.000038
8	1	0	1.777082	2.059338	-0.000025
9	1	0	-0.716273	2.166049	-0.000011
10	1	0	1.446286	-2.207289	-0.000011
11	1	0	2.884178	-0.191728	-0.000009
12	1	0	-2.592861	-0.817168	-0.000061

Pyridine Derivative H (monomer)

HF = -362.8610997 hartrees

Zero-point correction= 0.121393 (Hartree/Particle)

Thermal correction to Gibbs Free Energy= 0.090072

Sum of electronic and zero-point Energies= -362.739707

Sum of electronic and thermal Free Energies= -362.771028

Standard orientation:

Center Number	Atomic Number	Atomic Type	Coordinates (Angstroms) X	Y	Z
1	6	0	1.340844	-0.011829	-0.000206
2	6	0	0.569177	-1.156474	-0.000223
3	7	0	-0.771337	-1.165455	-0.000062
4	6	0	-1.387842	-0.001730	0.000077
5	6	0	-0.724111	1.223428	-0.000048
6	6	0	0.651394	1.204139	-0.000188
7	8	0	-2.742698	0.004197	0.000207
8	6	0	2.839239	-0.069977	0.000269
9	1	0	1.046957	-2.130463	-0.000451
10	1	0	-1.285375	2.147619	-0.000112
11	1	0	1.203015	2.137306	-0.000357
12	1	0	-3.047638	-0.911258	0.000115
13	1	0	3.190447	-1.102085	-0.006831
14	1	0	3.250115	0.422713	0.884055
15	1	0	3.251213	0.435440	-0.875728

Pyridine Derivative I (monomer)

HF = -346.8086801 hartrees

Zero-point correction= 0.132951 (Hartree/Particle)

Thermal correction to Gibbs Free Energy= 0.101973

Sum of electronic and zero-point Energies= -346.675729

Sum of electronic and thermal Free Energies= -346.706707

Standard orientation:

Center Number	Atomic Number	Atomic Type	Coordinates (Angstroms) X	Y	Z
1	6	0	-0.393047	0.765701	0.000003
2	6	0	-0.586779	-0.618718	0.000086
3	6	0	0.544534	-1.427814	0.000038
4	6	0	1.826001	-0.895117	-0.000024
5	6	0	1.991315	0.482034	-0.000050
6	6	0	0.882457	1.313265	-0.000011
7	6	0	-1.970117	-1.201501	-0.000034
8	8	0	-1.444611	1.642765	0.000148
9	1	0	0.409678	-2.503550	0.000091
10	1	0	2.686778	-1.551415	0.000077
11	1	0	2.983935	0.914841	-0.000118
12	1	0	0.992422	2.390674	-0.000040
13	1	0	-2.537985	-0.886963	0.880583
14	1	0	-1.927314	-2.289609	0.001300
15	1	0	-2.536700	-0.888947	-0.882178
16	1	0	-2.280107	1.165745	-0.000943

Pyridine Derivative J (monomer)

HF = -362.8520864 hartrees
Zero-point correction= 0.121082 (Hartree/Particle)
Thermal correction to Gibbs Free Energy= 0.090076
Sum of electronic and zero-point Energies= -362.731005
Sum of electronic and thermal Free Energies= -362.762010

Standard orientation:

Center Number	Atomic Number	Atomic Type	Coordinates (Angstroms) X	Y	Z
1	6	0	0.379007	0.768323	0.000009
2	6	0	0.550582	-0.621485	-0.000008
3	7	0	-0.499666	-1.441593	-0.000089
4	6	0	-1.729378	-0.926049	-0.000095
5	6	0	-1.975473	0.433416	0.000068
6	6	0	-0.897015	1.300603	0.000142
7	6	0	1.923985	-1.218113	0.000107
8	8	0	1.431419	1.635069	-0.000281
9	1	0	-2.549558	-1.634361	-0.000250
10	1	0	-2.990191	0.808159	0.000223
11	1	0	-1.031388	2.375060	0.000318
12	1	0	2.490816	-0.906377	0.882214
13	1	0	2.491094	-0.906382	-0.881809
14	1	0	1.858194	-2.303842	0.000081
15	1	0	2.267100	1.158175	0.000750

Pyridine Derivative K (monomer)

HF = -342.9931098 hartrees

Zero-point correction= 0.133682 (Hartree/Particle)

Thermal correction to Gibbs Free Energy= 0.102389

Sum of electronic and zero-point Energies= -342.859428

Sum of electronic and thermal Free Energies= -342.890721

Standard orientation:

Center Number	Atomic Number	Atomic Type	Coordinates (Angstroms) X	Y	Z
1	6	0	-1.123560	1.159906	0.003281
2	6	0	-1.147458	-0.223658	-0.000323
3	7	0	-0.026565	-0.960459	-0.004884
4	6	0	1.152729	-0.340465	-0.008966
5	6	0	1.267036	1.058025	-0.002368
6	6	0	0.112076	1.802118	0.004249
7	6	0	-2.439721	-0.981083	0.003454
8	7	0	2.273881	-1.129504	-0.065893
9	1	0	-2.046868	1.722931	0.008522
10	1	0	2.244379	1.522748	-0.001456
11	1	0	0.164807	2.884155	0.011720
12	1	0	-3.295394	-0.307087	0.014053
13	1	0	-2.496410	-1.631034	0.879237
14	1	0	-2.508681	-1.618473	-0.880705
15	1	0	3.133385	-0.706782	0.244452
16	1	0	2.146951	-2.085776	0.223657

Pyridine Derivative L (monomer)

HF = -287.6127912 hartrees

Zero-point correction= 0.117078 (Hartree/Particle)

Thermal correction to Gibbs Free Energy= 0.087096

Sum of electronic and zero-point Energies= -287.495713

Sum of electronic and thermal Free Energies= -287.525696

Standard orientation:

Center Number	Atomic Number	Atomic Type	Coordinates (Angstroms) X	Y	Z
1	7	0	-0.253353	-1.187072	0.000026
2	6	0	-0.874170	-0.001494	0.000029
3	6	0	-0.165744	1.195669	0.000025
4	6	0	1.218005	1.167855	-0.000005
5	6	0	1.860185	-0.059095	-0.000028
6	6	0	1.078760	-1.201045	0.000005
7	6	0	-2.371484	-0.018724	-0.000024
8	1	0	-0.700965	2.136309	0.000062
9	1	0	1.785605	2.090046	-0.000018
10	1	0	2.939080	-0.135200	-0.000049
11	1	0	1.546760	-2.179531	0.000000
12	1	0	-2.780490	0.990782	-0.000088
13	1	0	-2.744981	-0.545936	0.880225
14	1	0	-2.744847	-0.545962	-0.880326

Pyridine Derivative M (monomer)

HF = -323.5303203 hartrees

Zero-point correction= 0.093878 (Hartree/Particle)

Thermal correction to Gibbs Free Energy= 0.065012

Sum of electronic and zero-point Energies= -323.436442

Sum of electronic and thermal Free Energies= -323.465308

Standard orientation:

Center Number	Atomic Number	Atomic Type	Coordinates (Angstroms) X	Y	Z
1	6	0	-1.157173	1.159073	-0.000013
2	6	0	0.226419	1.200190	0.000019
3	6	0	0.914839	-0.002887	0.000080
4	6	0	0.190164	-1.189431	0.000165
5	7	0	-1.137993	-1.228003	0.000009
6	6	0	-1.796917	-0.068131	-0.000066
7	8	0	2.271079	-0.092432	-0.000369
8	1	0	-1.735221	2.073611	-0.000030
9	1	0	0.765872	2.140048	-0.000002
10	1	0	0.721045	-2.135175	0.000202
11	1	0	-2.878791	-0.123575	-0.000157
12	1	0	2.660415	0.787685	0.001759

Pyridine Derivative N (monomer)

HF = -323.5356016 hartrees
Zero-point correction= 0.094179 (Hartree/Particle)
Thermal correction to Gibbs Free Energy= 0.065366
Sum of electronic and zero-point Energies= -323.441422
Sum of electronic and thermal Free Energies= -323.470236

Standard orientation:

Center Number	Atomic Number	Atomic Type	Coordinates (Angstroms) X	Y	Z
1	6	0	1.187888	-1.115011	0.000060
2	6	0	-0.188948	-1.207232	0.000005
3	6	0	-0.926563	-0.029382	-0.000007
4	6	0	-0.244612	1.181668	0.000006
5	6	0	1.136599	1.152756	0.000072
6	7	0	1.866520	0.035787	-0.000054
7	8	0	-2.273433	-0.116151	0.000009
8	1	0	1.780065	-2.023220	-0.000134
9	1	0	-0.684174	-2.168882	-0.000066
10	1	0	-0.780960	2.122165	-0.000064
11	1	0	1.685633	2.087735	-0.000135
12	1	0	-2.664920	0.764101	-0.000106

Pyridine Derivative O (monomer)

HF = -362.8514983 hartrees
Zero-point correction= 0.121303 (Hartree/Particle)
Thermal correction to Gibbs Free Energy= 0.089963
Sum of electronic and zero-point Energies= -362.730196
Sum of electronic and thermal Free Energies= -362.761535

Standard orientation:

Center Number	Atomic Number	Atomic Type	Coordinates (Angstroms) X	Y	Z
1	6	0	-0.659984	1.196633	-0.000023
2	6	0	-1.331886	-0.021244	-0.000005
3	7	0	-0.663305	-1.178075	-0.000023
4	6	0	0.665806	-1.153972	-0.000012
5	6	0	1.404249	0.020837	0.000006
6	6	0	0.721993	1.225784	-0.000024
7	6	0	-2.828807	-0.091618	0.000030
8	8	0	2.767141	0.030299	0.000070
9	1	0	-1.221981	2.121579	-0.000059
10	1	0	1.180713	-2.110305	-0.000064
11	1	0	1.267264	2.161288	-0.000053
12	1	0	-3.187723	-0.628530	0.880647
13	1	0	-3.187796	-0.628596	-0.880501
14	1	0	-3.270817	0.904116	0.000017
15	1	0	3.098125	-0.873934	-0.000213

Pyridine Derivative P (monomer)

HF = -362.8578173 hartrees

Zero-point correction= 0.121853 (Hartree/Particle)

Thermal correction to Gibbs Free Energy= 0.090789

Sum of electronic and zero-point Energies= -362.735965

Sum of electronic and thermal Free Energies= -362.767028

Standard orientation:

Center Number	Atomic Number	Atomic Type	Coordinates (Angstroms) X	Y	Z
1	7	0	-1.133748	1.142852	0.000017
2	6	0	-1.177048	-0.197204	0.000074
3	6	0	-0.032333	-0.978461	0.000005
4	6	0	1.209329	-0.357415	-0.000020
5	6	0	1.264984	1.030250	-0.000008
6	6	0	0.067797	1.718222	-0.000020
7	6	0	-2.534432	-0.829657	-0.000029
8	8	0	2.314132	-1.135487	0.000002
9	1	0	-0.098577	-2.058539	-0.000005
10	1	0	2.211993	1.554512	-0.000014
11	1	0	0.080455	2.802824	-0.000006
12	1	0	-3.098213	-0.513476	-0.880016
13	1	0	-3.097999	-0.514213	0.880358
14	1	0	-2.468895	-1.916752	-0.000491
15	1	0	3.104638	-0.584836	0.000025

SXVIII. DFT energies and coordinates for pyridine derivatives E-P, Dimers

Pyridine Derivative E (dimer)

HF = -725.7489444 hartrees
Zero-point correction= 0.244125 (Hartree/Particle)
Thermal correction to Gibbs Free Energy= 0.200360
Sum of electronic and zero-point Energies= -725.504820
Sum of electronic and thermal Free Energies= -725.548584

Standard orientation:

Center Number	Atomic Number	Atomic Type	Coordinates (Angstroms) X	Y	Z
1	6	0	4.061995	0.321359	-0.000187
2	6	0	2.832251	0.949331	-0.000275
3	7	0	1.684963	0.246270	-0.000000
4	6	0	1.732458	-1.079565	0.000364
5	6	0	2.933579	-1.791618	0.000486
6	6	0	4.104306	-1.070476	0.000207
7	6	0	2.699732	2.439637	-0.000663
8	8	0	0.585232	-1.761317	0.000654
9	1	0	4.970030	0.908015	-0.000407
10	1	0	2.918593	-2.872463	0.000793
11	1	0	5.056750	-1.585788	0.000293
12	1	0	3.676762	2.919722	-0.000850
13	1	0	2.145636	2.771613	0.879649
14	1	0	2.145527	2.771148	-0.881083
15	1	0	-0.207398	-1.146647	0.000279
16	6	0	-4.062013	-0.321277	-0.000019
17	6	0	-2.832305	-0.949321	-0.000415
18	7	0	-1.684980	-0.246322	-0.000358
19	6	0	-1.732408	1.079521	0.000109
20	6	0	-2.933480	1.791641	0.000547
21	6	0	-4.104243	1.070559	0.000478
22	6	0	-2.699880	-2.439636	-0.000866
23	8	0	-0.585167	1.761234	0.000152
24	1	0	-4.970083	-0.907879	-0.000089
25	1	0	-2.918443	2.872487	0.000925
26	1	0	-5.056657	1.585925	0.000807
27	1	0	-3.676940	-2.919661	-0.001425
28	1	0	-2.146134	-2.771701	0.879637
29	1	0	-2.145367	-2.771128	-0.881095
30	1	0	0.207376	1.146464	0.000030

Pyridine Derivative F (dimer)

HF = -725.7426051 hartrees

Zero-point correction= 0.244454 (Hartree/Particle)

Thermal correction to Gibbs Free Energy= 0.200633

Sum of electronic and zero-point Energies= -725.498151

Sum of electronic and thermal Free Energies= -725.541972

Standard orientation:

Center Number	Atomic Number	Atomic Type	Coordinates (Angstroms) X	Y	Z
1	6	0	-4.184701	-0.527431	-0.000368
2	6	0	-3.383706	0.593863	0.000638
3	6	0	-1.994688	0.373487	0.000239
4	7	0	-1.450769	-0.835386	-0.001040
5	6	0	-2.255976	-1.907241	-0.002016
6	6	0	-3.624407	-1.804867	-0.001729
7	6	0	-3.929382	1.987111	0.002080
8	8	0	-1.186605	1.435581	0.001236
9	1	0	-5.261843	-0.406887	-0.000098
10	1	0	-1.760076	-2.870677	-0.003047
11	1	0	-4.242841	-2.691243	-0.002527
12	1	0	-5.018729	1.970028	0.002184
13	1	0	-3.592319	2.542296	-0.876095
14	1	0	-3.592121	2.540564	0.881271
15	1	0	-0.221928	1.150901	0.000396
16	6	0	4.184704	0.527392	-0.000172
17	6	0	3.383684	-0.593883	0.000787
18	6	0	1.994681	-0.373465	0.000258
19	7	0	1.450790	0.835424	-0.001100
20	6	0	2.256019	1.907261	-0.002025
21	6	0	3.624447	1.804846	-0.001610
22	6	0	3.929321	-1.987149	0.002310
23	8	0	1.186595	-1.435550	0.001186
24	1	0	5.261842	0.406817	0.000202
25	1	0	1.760144	2.870709	-0.003121
26	1	0	4.242908	2.691203	-0.002370
27	1	0	5.018670	-1.970099	0.002524
28	1	0	3.592334	-2.542341	-0.875889
29	1	0	3.591955	-2.540576	0.881477
30	1	0	0.221961	-1.150762	0.000362

Pyridine Derivative G (dimer)

HF = -647.10185 hartrees

Zero-point correction= 0.189083 (Hartree/Particle)

Thermal correction to Gibbs Free Energy= 0.148965

Sum of electronic and zero-point Energies= -646.912767

Sum of electronic and thermal Free Energies= -646.952885

Standard orientation:

Center Number	Atomic Number	Atomic Type	Coordinates (Angstroms) X	Y	Z
1	6	0	4.215814	-0.362025	-0.000207
2	6	0	3.183105	-1.270080	-0.000388
3	6	0	1.873030	-0.782724	-0.000240
4	7	0	1.596610	0.516276	0.000028
5	6	0	2.614816	1.387828	0.000193
6	6	0	3.934492	1.003815	0.000101
7	8	0	0.862969	-1.653632	-0.000429
8	1	0	5.241057	-0.710095	-0.000297
9	1	0	3.355844	-2.337385	-0.000624
10	1	0	2.336194	2.435158	0.000423
11	1	0	4.721429	1.744415	0.000260
12	1	0	-0.022543	-1.178162	-0.000241
13	6	0	-4.215820	0.362005	0.000336
14	6	0	-3.183121	1.270073	0.000228
15	6	0	-1.873038	0.782734	0.000095
16	7	0	-1.596606	-0.516261	0.000011
17	6	0	-2.614798	-1.387826	0.000106
18	6	0	-3.934481	-1.003832	0.000291
19	8	0	-0.862976	1.653645	-0.000071
20	1	0	-5.241067	0.710061	0.000467
21	1	0	-3.355877	2.337375	0.000257
22	1	0	-2.336162	-2.435152	0.000049
23	1	0	-4.721409	-1.744439	0.000383
24	1	0	0.022555	1.178199	-0.000050

Pyridine Derivative H (dimer)

HF = -725.7400057 hartrees

Zero-point correction= 0.244121 (Hartree/Particle)

Thermal correction to Gibbs Free Energy= 0.199376

Sum of electronic and zero-point Energies= -725.495885

Sum of electronic and thermal Free Energies= -725.540630

Standard orientation:

Center Number	Atomic Number	Atomic Type	Coordinates (Angstroms) X	Y	Z
1	6	0	4.058992	-0.350325	0.001215
2	6	0	2.809500	-0.929164	0.001002
3	7	0	1.660487	-0.235948	-0.000143
4	6	0	1.715680	1.087055	-0.001115
5	6	0	2.930113	1.778362	-0.001079
6	6	0	4.094259	1.049054	0.000071
7	8	0	0.573796	1.782904	-0.002235
8	6	0	5.312884	-1.172300	0.002754
9	1	0	2.702909	-2.008786	0.001746
10	1	0	2.931849	2.859693	-0.001961
11	1	0	5.049142	1.562332	0.000100
12	1	0	-0.216970	1.164897	-0.001662
13	1	0	5.081981	-2.237767	0.001403
14	1	0	5.920583	-0.956643	0.884073
15	1	0	5.923827	-0.955032	-0.875903
16	6	0	-4.058999	0.350309	0.001080
17	6	0	-2.809518	0.929170	0.001064
18	7	0	-1.660496	0.235971	-0.000154
19	6	0	-1.715670	-1.087035	-0.001402
20	6	0	-2.930088	-1.778362	-0.001556
21	6	0	-4.094244	-1.049070	-0.000330
22	8	0	-0.573787	-1.782881	-0.002559
23	6	0	-5.312903	1.172265	0.002663
24	1	0	-2.702947	2.008794	0.002028
25	1	0	-2.931809	-2.859693	-0.002639
26	1	0	-5.049118	-1.562365	-0.000456
27	1	0	0.216960	-1.164854	-0.001821
28	1	0	-5.920823	0.956212	0.883731
29	1	0	-5.923619	0.955373	-0.876246
30	1	0	-5.082016	2.237736	0.001831

Pyridine Derivative I (dimer)

HF = -693.629853 hartrees

Zero-point correction= 0.266898 (Hartree/Particle)

Thermal correction to Gibbs Free Energy= 0.221096

Sum of electronic and zero-point Energies= -693.362955

Sum of electronic and thermal Free Energies= -693.408757

Standard orientation:

Center Number	Atomic Number	Atomic Type	Coordinates (Angstroms) X	Y	Z
1	6	0	1.786359	-0.775085	0.065089
2	6	0	1.461894	-0.092730	-1.110882
3	6	0	1.542817	1.295002	-1.094008
4	6	0	1.938227	1.990959	0.039719
5	6	0	2.265678	1.289729	1.190568
6	6	0	2.191259	-0.093994	1.203835
7	6	0	1.039994	-0.840658	-2.341392
8	8	0	1.718712	-2.138915	0.148243
9	1	0	1.284848	1.838543	-1.995832
10	1	0	1.986797	3.072198	0.023163
11	1	0	2.575650	1.817127	2.084126
12	1	0	2.431962	-0.657883	2.096413
13	1	0	1.836347	-1.499939	-2.698524
14	1	0	0.790816	-0.147638	-3.143667
15	1	0	0.158979	-1.462121	-2.155378
16	1	0	1.338117	-2.504483	-0.656514
17	6	0	-1.786840	-0.774253	-0.065572
18	6	0	-1.461987	-0.092833	1.110830
19	6	0	-1.541953	1.294967	1.094786
20	6	0	-1.936815	1.991872	-0.038547
21	6	0	-2.264677	1.291561	-1.189839
22	6	0	-2.191188	-0.092201	-1.203939
23	6	0	-1.040820	-0.841793	2.340962
24	8	0	-1.720128	-2.138087	-0.149524
25	1	0	-1.283687	1.837790	1.996957
26	1	0	-1.984648	3.073134	-0.021342
27	1	0	-2.574231	1.819710	-2.083097
28	1	0	-2.432187	-0.655389	-2.096878
29	1	0	-0.790963	-0.149416	3.143583
30	1	0	-0.160448	-1.464106	2.154736
31	1	0	-1.837857	-1.500428	2.697765
32	1	0	-1.339863	-2.504337	0.655078

Pyridine Derivative J (dimer)

HF = -725.7165687 hartrees

Zero-point correction= 0.243553 (Hartree/Particle)

Thermal correction to Gibbs Free Energy= 0.198781

Sum of electronic and zero-point Energies= -725.473016

Sum of electronic and thermal Free Energies= -725.517787

Standard orientation:

Center Number	Atomic Number	Atomic Type	Coordinates (Angstroms) X	Y	Z
1	6	0	1.709948	-0.244003	-0.834157
2	6	0	1.699566	0.832250	0.062230
3	7	0	1.728853	0.629728	1.378232
4	6	0	1.753961	-0.619784	1.843388
5	6	0	1.771172	-1.729099	1.020751
6	6	0	1.752637	-1.537166	-0.349927
7	6	0	1.665568	2.239232	-0.446810
8	8	0	1.685148	-0.066142	-2.185061
9	1	0	1.766447	-0.732672	2.921157
10	1	0	1.795304	-2.725023	1.442119
11	1	0	1.759875	-2.370566	-1.041037
12	1	0	0.771016	2.420240	-1.049450
13	1	0	2.534914	2.451577	-1.075567
14	1	0	1.662845	2.938282	0.386489
15	1	0	1.600385	0.867590	-2.403294
16	6	0	-1.715122	-0.301841	0.819122
17	6	0	-1.707295	0.822630	-0.015805
18	7	0	-1.722804	0.692741	-1.341625
19	6	0	-1.730619	-0.528963	-1.875116
20	6	0	-1.742774	-1.682726	-1.115326
21	6	0	-1.738808	-1.566799	0.263591
22	6	0	-1.689706	2.200304	0.569417
23	8	0	-1.708273	-0.199989	2.177954
24	1	0	-1.732871	-0.582452	-2.957579
25	1	0	-1.751745	-2.653670	-1.591929
26	1	0	-1.743230	-2.436740	0.908108
27	1	0	-0.799453	2.356340	1.185500
28	1	0	-2.563733	2.370424	1.204472
29	1	0	-1.689814	2.943206	-0.225018
30	1	0	-1.638448	0.721012	2.448482

Pyridine Derivative K (dimer)

HF = -685.996633 hartrees

Zero-point correction= 0.268847 (Hartree/Particle)

Thermal correction to Gibbs Free Energy= 0.223201

Sum of electronic and zero-point Energies= -685.727786

Sum of electronic and thermal Free Energies= -685.773432

Standard orientation:

Center Number	Atomic Number	Atomic Type	Coordinates (Angstroms) X	Y	Z
1	6	0	4.132845	0.399997	0.398900
2	6	0	2.916887	0.979765	0.086255
3	7	0	1.835244	0.249548	-0.220169
4	6	0	1.920867	-1.083006	-0.224311
5	6	0	3.118904	-1.748281	0.085991
6	6	0	4.222089	-0.990900	0.395961
7	6	0	2.730565	2.466432	0.073869
8	7	0	0.799292	-1.773718	-0.583374
9	1	0	4.987586	1.016393	0.641300
10	1	0	3.155035	-2.829880	0.077397
11	1	0	5.159259	-1.476628	0.640154
12	1	0	3.653528	2.985029	0.329843
13	1	0	1.956733	2.757344	0.787725
14	1	0	2.404446	2.799826	-0.913670
15	1	0	0.772176	-2.748423	-0.333431
16	1	0	-0.085656	-1.273475	-0.501115
17	6	0	-4.132788	-0.399893	0.399140
18	6	0	-2.916948	-0.979745	0.086170
19	7	0	-1.835349	-0.249590	-0.220538
20	6	0	-1.920865	1.082972	-0.224611
21	6	0	-3.118773	1.748326	0.086009
22	6	0	-4.221929	0.991009	0.396250
23	6	0	-2.730754	-2.466425	0.073659
24	7	0	-0.799274	1.773559	-0.583849
25	1	0	-4.987519	-1.016225	0.641740
26	1	0	-3.154837	2.829927	0.077434
27	1	0	-5.159007	1.476797	0.640680
28	1	0	-1.957003	-2.757462	0.787555
29	1	0	-2.404584	-2.799763	-0.913880
30	1	0	-3.653778	-2.984965	0.329529
31	1	0	-0.771973	2.748243	-0.333847
32	1	0	0.085602	1.273170	-0.501605

Pyridine Derivative L (dimer)

HF = -575.2356486 hartrees

Zero-point correction= 0.235277 (Hartree/Particle)

Thermal correction to Gibbs Free Energy= 0.192884

Sum of electronic and zero-point Energies= -575.000372

Sum of electronic and thermal Free Energies= -575.042764

Standard orientation:

Center Number	Atomic Number	Atomic Type	Coordinates (Angstroms) X	Y	Z
1	7	0	-2.217707	0.621007	0.732002
2	6	0	-1.681106	0.830250	-0.474569
3	6	0	-1.310220	-0.223933	-1.305386
4	6	0	-1.500371	-1.524795	-0.879497
5	6	0	-2.050941	-1.742319	0.373557
6	6	0	-2.385752	-0.638825	1.135807
7	6	0	-1.522986	2.251110	-0.919939
8	1	0	-0.870327	-0.015752	-2.272175
9	1	0	-1.213297	-2.356674	-1.510330
10	1	0	-2.210879	-2.740724	0.757431
11	1	0	-2.814276	-0.770460	2.123506
12	1	0	-2.463578	2.616212	-1.341515
13	1	0	-0.755024	2.342428	-1.687713
14	1	0	-1.265740	2.893590	-0.078653
15	7	0	2.218318	0.619488	-0.732085
16	6	0	1.681880	0.829412	0.474436
17	6	0	1.309948	-0.224302	1.305390
18	6	0	1.498890	-1.525402	0.879704
19	6	0	2.049313	-1.743628	-0.373297
20	6	0	2.385188	-0.640565	-1.135700
21	6	0	1.525127	2.250480	0.919625
22	1	0	0.870198	-0.015561	2.272123
23	1	0	1.211006	-2.356917	1.510647
24	1	0	2.208332	-2.742240	-0.757013
25	1	0	2.813629	-0.772749	-2.123361
26	1	0	2.465933	2.614552	1.341620
27	1	0	0.756921	2.342716	1.687047
28	1	0	1.269005	2.893205	0.078184

Pyridine Derivative M (dimer)

HF = -647.0718874 hartrees

Zero-point correction= 0.188920 (Hartree/Particle)

Thermal correction to Gibbs Free Energy= 0.146832

Sum of electronic and zero-point Energies= -646.882968

Sum of electronic and thermal Free Energies= -646.925055

Standard orientation:

Center Number	Atomic Number	Atomic Type	Coordinates (Angstroms) X	Y	Z
1	6	0	-3.248368	1.334241	0.671556
2	6	0	-2.077852	0.693589	0.302449
3	6	0	-2.165350	-0.587877	-0.228099
4	6	0	-3.430720	-1.158660	-0.357786
5	7	0	-4.553631	-0.543686	-0.003975
6	6	0	-4.459351	0.686697	0.503368
7	8	0	-1.100529	-1.312378	-0.628741
8	1	0	-3.219652	2.332754	1.087676
9	1	0	-1.116998	1.177524	0.423531
10	1	0	-3.516447	-2.158875	-0.769275
11	1	0	-5.386935	1.170612	0.784735
12	1	0	-0.238191	-0.822839	-0.491613
13	6	0	2.812570	1.703487	-0.526679
14	6	0	3.860407	0.940433	-0.046687
15	6	0	3.614991	-0.382055	0.292951
16	6	0	2.328789	-0.883350	0.140149
17	7	0	1.327730	-0.140674	-0.323323
18	6	0	1.559737	1.128207	-0.651668
19	8	0	4.639664	-1.137266	0.762913
20	1	0	2.964278	2.738010	-0.802838
21	1	0	4.855446	1.351693	0.066024
22	1	0	2.107149	-1.913434	0.399504
23	1	0	0.716546	1.695454	-1.026486
24	1	0	4.333901	-2.031493	0.949129

Pyridine Derivative N (dimer)

HF = -647.0835497 hartrees
Zero-point correction= 0.189088 (Hartree/Particle)
Thermal correction to Gibbs Free Energy= 0.146406
Sum of electronic and zero-point Energies= -646.894462
Sum of electronic and thermal Free Energies= -646.937144

Standard orientation:

Center Number	Atomic Number	Atomic Type	Coordinates (Angstroms) X	Y	Z
1	6	0	-2.119885	-1.276398	0.513302
2	6	0	-3.442682	-0.913047	0.636397
3	6	0	-3.855059	0.280351	0.052346
4	6	0	-2.918438	1.056083	-0.623425
5	6	0	-1.619130	0.600275	-0.687841
6	7	0	-1.211687	-0.544098	-0.136665
7	8	0	-5.148516	0.633091	0.166328
8	1	0	-1.765327	-2.200501	0.954332
9	1	0	-4.144731	-1.538103	1.170776
10	1	0	-3.201895	1.991123	-1.089026
11	1	0	-0.864720	1.178671	-1.208820
12	1	0	-5.308017	1.469209	-0.285559
13	6	0	4.599815	-0.125386	-0.027004
14	6	0	3.586245	-1.018458	-0.303870
15	6	0	2.263766	-0.597882	-0.166383
16	6	0	2.042937	0.715690	0.249109
17	6	0	3.135999	1.521869	0.498551
18	7	0	4.407571	1.136043	0.371129
19	8	0	1.280225	-1.460691	-0.437662
20	1	0	5.631105	-0.444117	-0.132187
21	1	0	3.808840	-2.028260	-0.622730
22	1	0	1.041081	1.103156	0.375844
23	1	0	2.974080	2.544571	0.821526
24	1	0	0.363309	-1.057147	-0.301824

Pyridine Derivative O (dimer)

HF = -725.7160373 hartrees

Zero-point correction= 0.243620 (Hartree/Particle)

Thermal correction to Gibbs Free Energy= 0.197338

Sum of electronic and zero-point Energies= -725.472417

Sum of electronic and thermal Free Energies= -725.518699

Standard orientation:

Center Number	Atomic Number	Atomic Type	Coordinates (Angstroms) X	Y	Z
1	6	0	4.095584	-0.315932	0.618126
2	6	0	3.876804	-0.136934	-0.743299
3	7	0	2.637518	-0.054568	-1.234791
4	6	0	1.602954	-0.144510	-0.403767
5	6	0	1.737061	-0.318965	0.971049
6	6	0	3.023149	-0.407262	1.485226
7	6	0	5.012627	-0.029821	-1.715612
8	8	0	0.679498	-0.408238	1.809143
9	1	0	5.107342	-0.382743	0.997315
10	1	0	0.614226	-0.078435	-0.844620
11	1	0	3.171333	-0.545376	2.549307
12	1	0	4.986815	0.931566	-2.233241
13	1	0	4.944704	-0.812172	-2.474518
14	1	0	5.974100	-0.122406	-1.211474
15	1	0	-0.186190	-0.259283	1.327159
16	6	0	-3.199707	1.474170	-0.518017
17	6	0	-2.020006	1.247683	0.181487
18	7	0	-1.703113	0.016099	0.588208
19	6	0	-2.510999	-1.005277	0.319682
20	6	0	-3.700992	-0.849673	-0.373249
21	6	0	-4.050207	0.422402	-0.800002
22	6	0	-1.056937	2.347034	0.507319
23	8	0	-4.531375	-1.890936	-0.649182
24	1	0	-3.448365	2.476346	-0.841132
25	1	0	-2.201305	-1.985602	0.667888
26	1	0	-4.973594	0.577280	-1.343837
27	1	0	-0.092804	2.166230	0.026136
28	1	0	-0.882225	2.397390	1.583576
29	1	0	-1.435107	3.310943	0.170460
30	1	0	-4.160747	-2.708547	-0.300278

Pyridine Derivative P (dimer)

HF = -725.73 hartrees

Zero-point correction= 0.244123 (Hartree/Particle)

Thermal correction to Gibbs Free Energy= 0.197382

Sum of electronic and zero-point Energies= -725.485876

Sum of electronic and thermal Free Energies= -725.532618

Standard orientation:

Center Number	Atomic Number	Atomic Type	Coordinates (Angstroms) X	Y	Z
1	7	0	1.378599	-0.931279	-0.173263
2	6	0	1.846184	-0.362454	0.947706
3	6	0	3.046844	0.323984	0.967596
4	6	0	3.784976	0.430615	-0.205028
5	6	0	3.298869	-0.159154	-1.366852
6	6	0	2.093388	-0.821070	-1.292434
7	6	0	1.001567	-0.499794	2.174914
8	8	0	4.948200	1.108643	-0.164192
9	1	0	3.409270	0.776545	1.880802
10	1	0	3.845406	-0.096515	-2.298630
11	1	0	1.673807	-1.288601	-2.175902
12	1	0	0.805082	-1.552917	2.382802
13	1	0	0.036896	-0.009924	2.023499
14	1	0	1.486845	-0.053705	3.041098
15	1	0	5.359609	1.118228	-1.035778
16	7	0	-4.099926	0.890727	-0.086556
17	6	0	-2.815464	1.267970	-0.141891
18	6	0	-1.769599	0.359602	-0.192246
19	6	0	-2.046049	-1.007237	-0.181047
20	6	0	-3.381319	-1.401447	-0.126062
21	6	0	-4.348654	-0.420353	-0.081728
22	6	0	-2.543303	2.741309	-0.147097
23	8	0	-1.093607	-1.946056	-0.221551
24	1	0	-0.748122	0.712277	-0.239923
25	1	0	-3.647863	-2.449855	-0.116748
26	1	0	-5.393888	-0.707845	-0.038134
27	1	0	-2.955517	3.205060	0.751430
28	1	0	-3.023510	3.212139	-1.007375
29	1	0	-1.475101	2.950595	-0.187440
30	1	0	-0.165013	-1.544151	-0.209996

SXIX. ^{1}H and ^{13}C NMR spectra for methylclioquinol

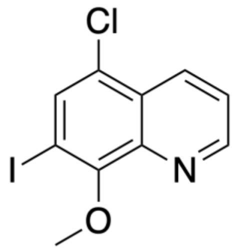

^{1}H NMR (400 MHz, DMSO) δ (ppm): 9.03 (1H, dd, J = 1.60, Ar, 4.16 Hz), 8.55 (1H, dd, Ar, J = 1.60, 8.60 Hz), 8.15 (1H, s, Ar), 7.77 (1H, dd, Ar, J = 4.16, 8.60 Hz), 4.06 (3H, s, OCH3), 3.32 (residual H_2O). ^{13}C NMR (400 MHz, DMSO) δ (ppm): 155.71, 151.07, 141.66, 134.58, 133.15, 126.73, 125.61, 123.38, 91.19, 61.82.

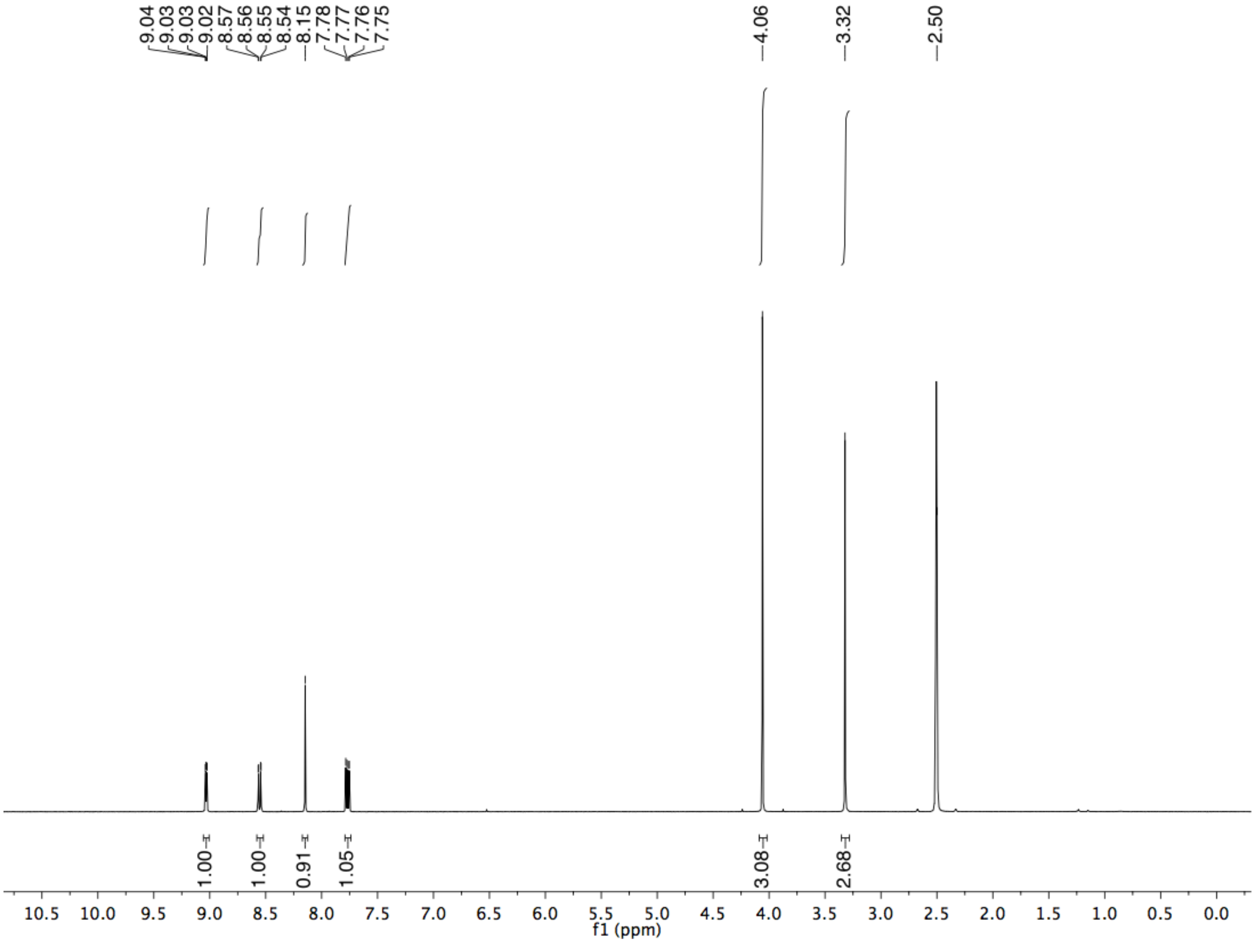

Figure S7. ^{1}H NMR spectrum of methylclioquinol in DMSO-d6.

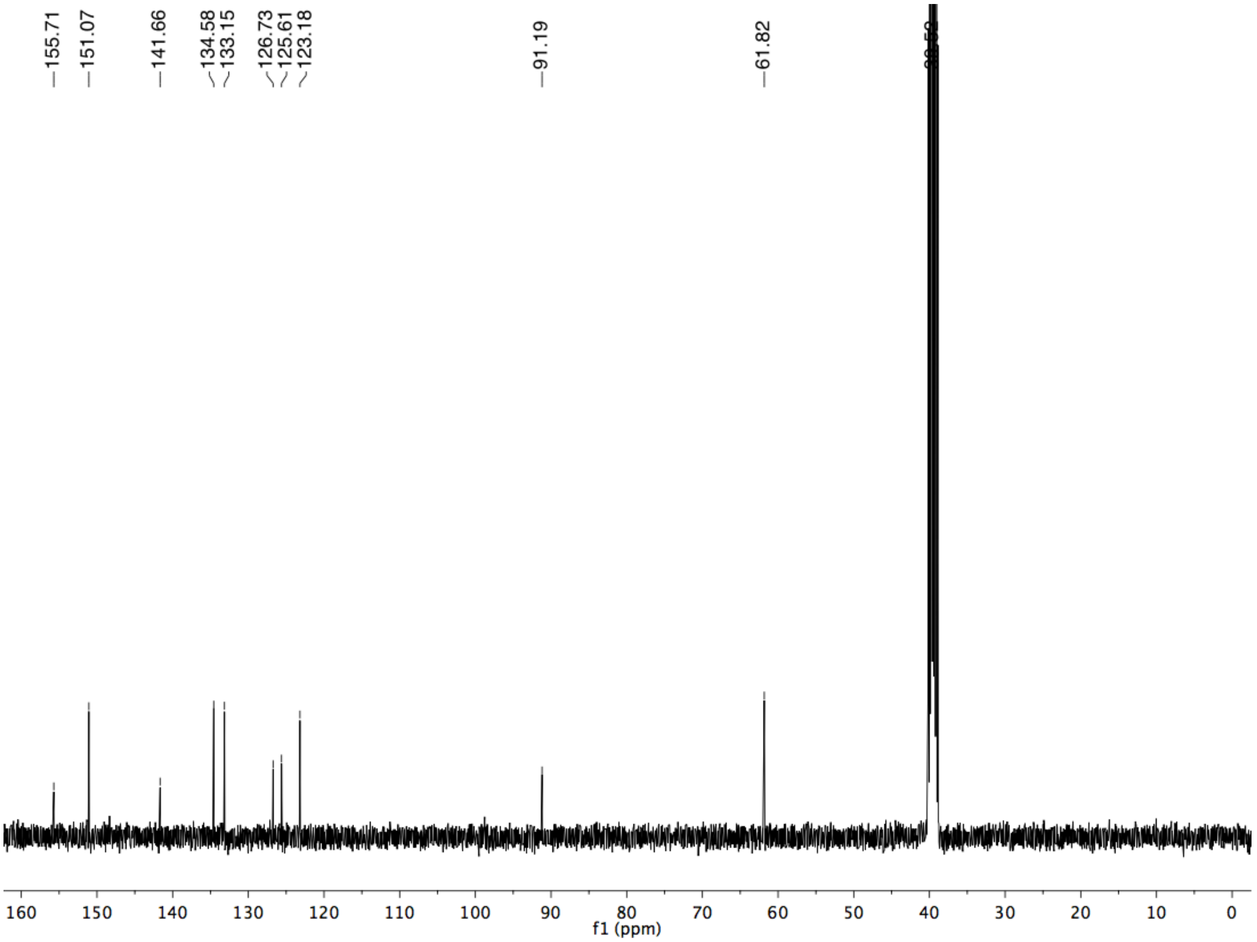

Figure S8. ^{13}C NMR spectrum of methylclioquinol in DMSO-d6.
